# Through-space donor–acceptor homoconjugation strategies for emissive radical species

**DOI:** 10.1039/d6sc00981f

**Published:** 2026-04-17

**Authors:** Ashton R. Davis, Yujie Zhao, Robert N. Sansone, Colleen H. McAloon, Robert G. Griffin, Timothy M. Swager

**Affiliations:** a Department of Chemistry, Massachusetts Institute of Technology Cambridge Massachusetts 02139 USA rgg@mit.edu tswager@mit.edu; b Department of Materials Science and Engineering, Massachusetts Institute of Technology Cambridge Massachusetts 02139 USA

## Abstract

Open shell luminescent organic molecules have been gaining attention in recent years for their ability to access different excited state manifolds compared to their closed shell congeners. However, there has been little work to expand the design of these systems beyond direct conjugation strategies. Herein, we report the synthesis, and optical and magnetic characterization of two new triarylmethyl radical compounds that interact *via* a remote homoconjugation with a donor group through a [2.2.2] bridge. The properties of these bridged radicals are compared to those of a non-bridged species. This study ultimately expands the design strategies for synthesizing emissive radical species.

## Abstract 2

We report the synthesis, and optical and magnetic characterization of two triarylmethyl radical compounds that interact *via* a remote homoconjugation with a donor group through a [2.2.2] bridge, and compare their properties to a non-bridged species.

## Graphical abstract

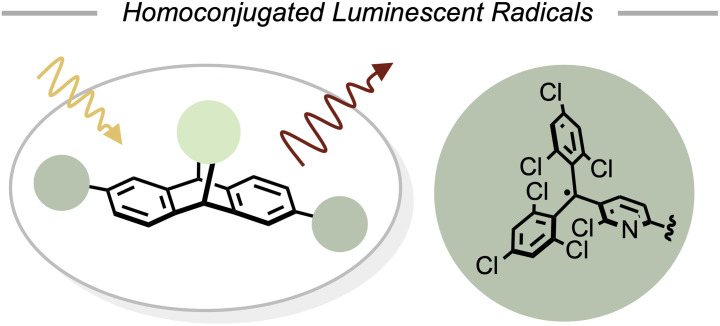

## Introduction

**Fig. 1:**
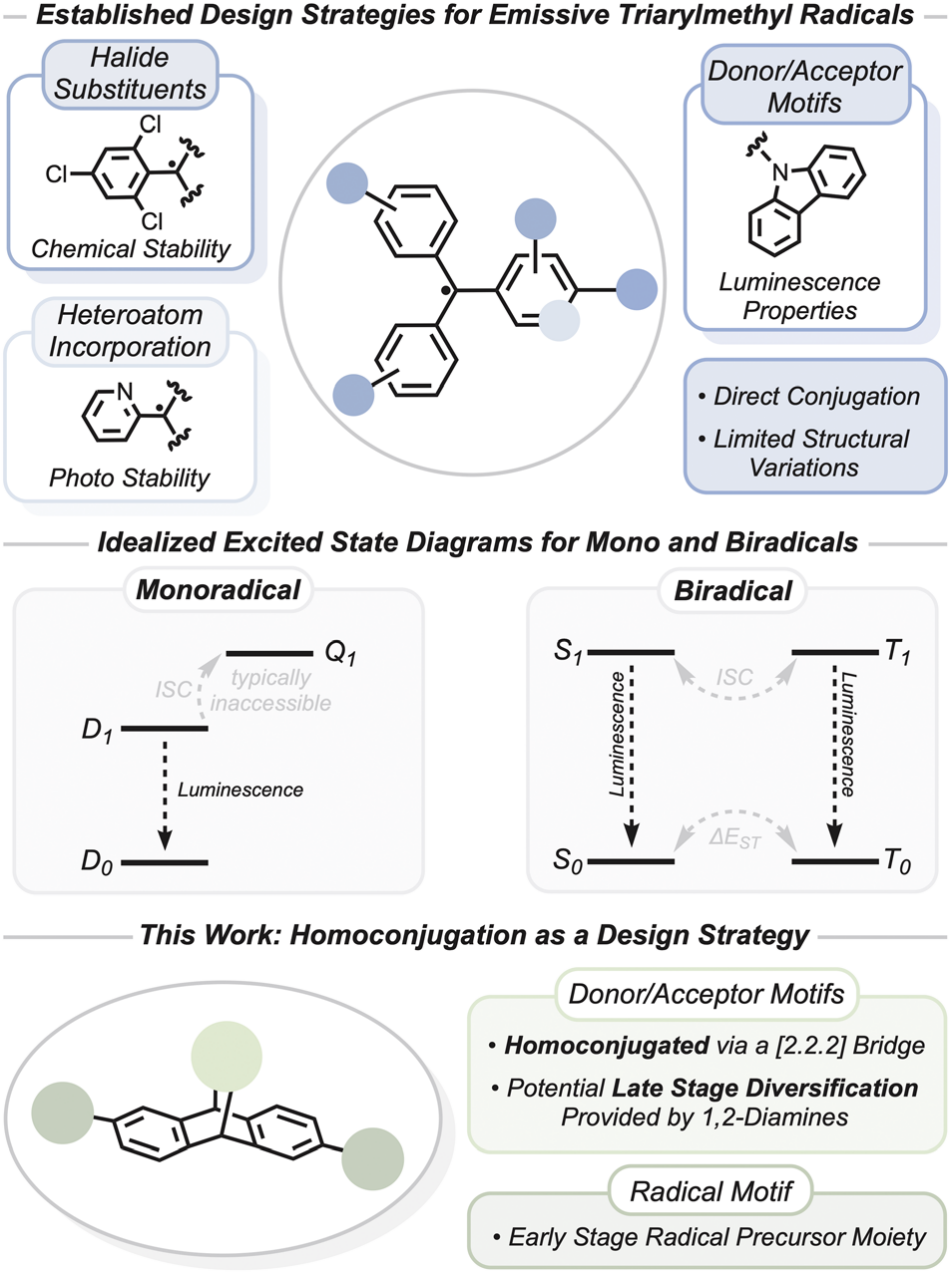
Established methods for synthesizing luminescent triarylmethyl radicals, example excited state diagrams for radical emitters, and an example homoconjugation design strategy.

The vast majority of organic radicals are non-emissive as a result of deactivation pathways afforded by a low energy half-filled orbital. However, there are a growing number of mono- and multi-radical systems that display high quantum yield emissions. These open shell lumophores have been considered for applications in organic light emitting devices as a means to circumvent the limitations of spin statistics resulting from the injection of uncorrelated spins at electrode interfaces.^1,2^ Of particular interest has been the use of triarylmethyl radicals.^3^ Chemical manipulation of these triarylmethyl species *via* the installation of bulky halide protecting groups,^4–8^ pyridyl groups,^9,10^ and electron donor or acceptor moieties (Fig. 1) has overcome their inherent photo and chemical instabilities and has produced a variety of highly stable open shell lumophores with quantum yield emissions near unity.^11–13^

Current strategies to manipulate the ground and excited state properties of triarylmethyl radicals typically make use of direct conjugation to either donor or acceptor groups. Kusamoto *et al.* contributed an informative review on this area.^14^ Typical donor groups for triarylmethyl radicals are N-heterocycles, particularly carbazoles,^15^ and tuning donor strength is an effective means to manipulate luminescent properties.^16–18^ To that end, structural diversity of triarylmethyl radical-based emitters has been relatively limited. We are interested in investigating open-shell lumophores with additional structural diversity that has been effective in tuning their closed-shell congeners and thereby advancing the understanding of these systems.

Homoconjugation has been applied to closed-shell systems to engineer thermally activated delayed fluorescence (TADF) manifolds.^19–21^ This method lowers the excited state singlet–triplet energy gap (Δ*E*_ST_) by minimizing the HOMO–LUMO overlap, and the through-space orbital mixing and spin–orbit coupling promote intersystem crossing (ISC) of electrons from the non-luminescent triplet manifold to the luminescent singlet manifold. Examples of excited state ISC for compounds with ground state doublet configurations (*i.e.* monoradicals) are comparatively rare, as the quartet excited state is generally thought of as energetically inaccessible (Fig. 1).^14^ Molecules with doublet ground states and quartet excited states have been characterized, but in these cases a local excitation occurs in a spatially separated chromophore.^22–25^ In other words, these monoradical quartet excited states are best described as a photoexcited triplet state tethered to a doublet ground state. In comparison, excited state ISC in biradical and biradicaloid††While the terms biradical and diradical are often used interchangeably in the literature, in this study, we use the IUPAC gold book definition of a biradical as an even electron entity with two radical centers which act nearly independently of each other, and biradicaloid when the spins interact significantly. See: https://doi.org/10.1351/goldbook.B00671 for more information. systems (Fig. 1) is common and has been highlighted in recent years as an important design strategy in the synthesis of optically addressable, organic molecular quantum bits (qubits).^26–29^ Both the singlet and triplet excited states can be emissive, and manipulation of these spin states leads to a host of interesting photophysical properties such as magnetoluminescence^30–32^ and spin-dependent emission and absorption.^33,34^ Homoconjugation in a biradical emitter has been used to produce symmetry forbidden charge transfer absorptions in the singlet spin state.^33^ However, homoconjugation as a broader design strategy has not been explored for open shell lumophores.

We describe the optical and magnetic properties of both mono and biradical species which exhibit homoconjugation *via* a [2.2.2] bridging group as well as a non-bridged monoradical for comparison (Fig. 1). We detail the synthesis and ground state characterization of both compounds and offer experimental evidence that the biradical has two, nearly non-interacting, spin centers. Investigations of their excited state properties, guided by calculations, reveal the prospects of homoconjugation in the design of radical lumophores.

## Results and discussion

### Synthesis of iptycenyl radicals

**Fig. 2:**
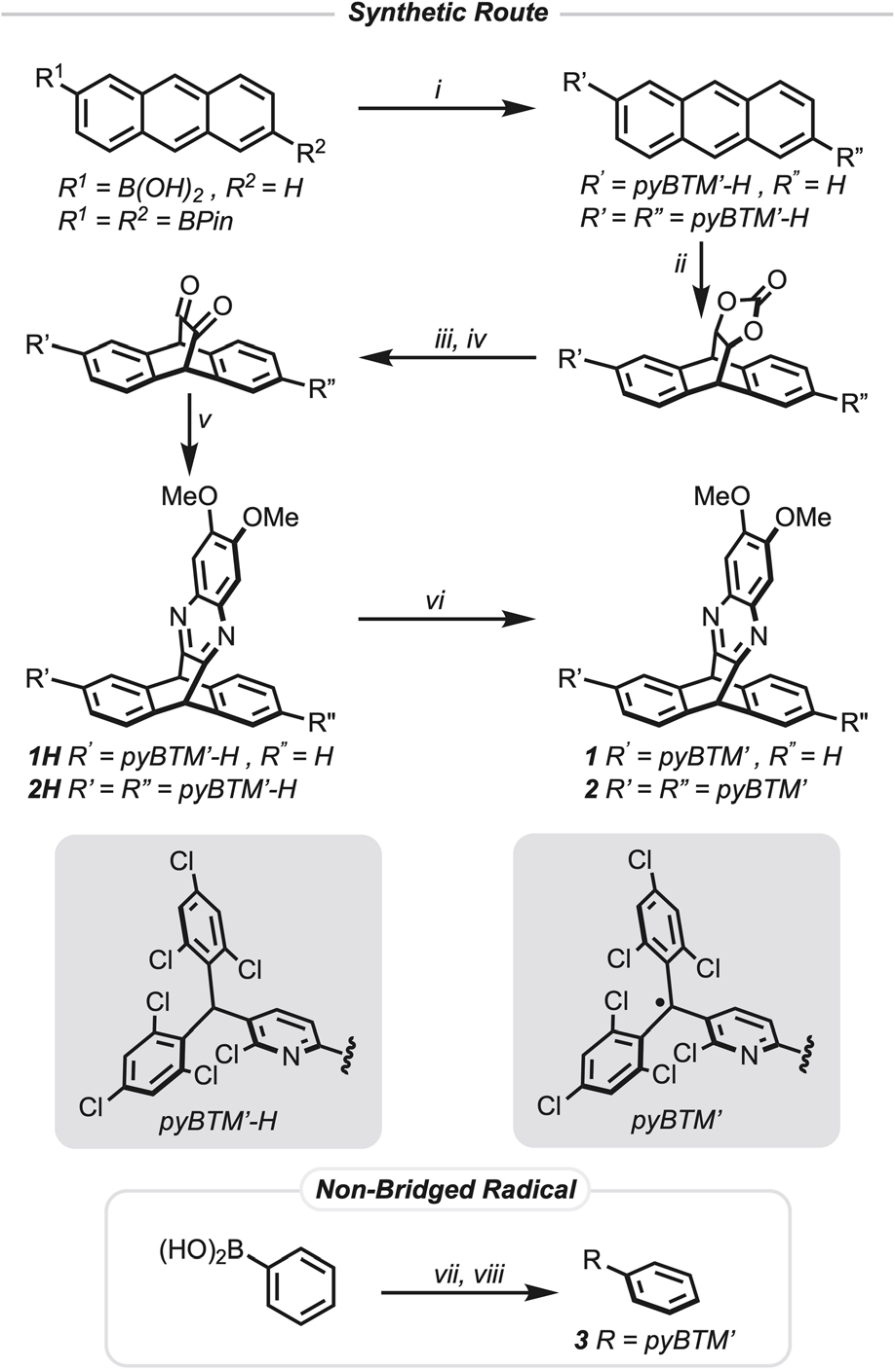
Conditions: (i) pyBTM′-Cl, Pd(PPh_3_)_4_, K_2_CO_3_, PhMe : H_2_O (1 : 1), 110 °C, 16 h; R^1^ ≠ R^2^ 80% yield, R^1^ = R^2^ not isolated. (ii) Vinylene carbonate, *p*-hydroquinone, xylenes, 180 °C, 16 h; R′ ≠ R″ 85% yield, R′ = R″ 95% yield (two steps). (iii) NaOH, THF : H_2_O (5 : 1), 90 °C, 4 h; R′ ≠ R″ 95% yield, R′ = R″ 85% yield. (iv) (CF_3_CO)_2_O, DCM : DMSO (6 : 1), −78 °C, 1 h, then *N*,*N*-diisopropylethylamine, −78 °C → 25 °C, 1 h; R′ ≠ R″ 95% yield, R′ = R″ 96% yield. (v) 1,2-Diamino-4,5-dimethoxybenzene, EtOH : AcOH (19 : 1), 100 °C, 1 h; 1H 54% yield, 2H 54% yield. (vi) Tetrabutylammonium hydroxide, THF, 25 °C, 1.5 h, then silver hexafluoroantimonate, MeCN, 25 °C, 1 h; 1 26% yield, 2 26% yield. (vii) pyBTM′-Cl, Pd(PPh_3_)_4_, K_2_CO_3_, PhMe : H_2_O (1 : 1), 110 °C, 16 h; 3H 97% yield. (viii) Tetrabutylammonium hydroxide, THF, 25 °C, 1.5 h, then silver hexafluoroantimonate, MeCN, 25 °C, 1 h; 3 71% yield.

To realize homoconjugation, we designed compounds 1 and 2 to contain a [2.2.2] bridging group based on a heterocyclic triptycene scaffold. The synthesis of compound 1 and compound 2 (Fig. 2) closely follows a route previously reported by our laboratory to access donor–acceptor TADF iptycene compounds.^19^ Radicals are considered electron-deficient moieties. We therefore decided to install methoxy groups on the pyrazine ring system to increase the electron density on this nominally electron-deficient group to increase interactions with the radical. Additionally, we chose pyBTM′ as the triarylmethyl radical because the inclusion of nitrogen in the aromatic ring system has been shown to improve the photostability of triarylmethyl radicals.^9,10,13,35,36^

The pyBTM′-H trityl moiety is first installed using a Suzuki–Miyaura cross-coupling reaction with either a mono- or di-borylated anthracene and (2,6-dichloro-3-pyridyl)bis(2,4,6-trichlorophenyl)methane (pyBTM′-Cl). Then a Diels–Alder reaction between 1,3-dioxol-2-one and either of the anthracenyl compounds generates the [2.2.2] bicyclic intermediate which is purified by silica gel column chromatography. Hydrolysis of the carbonate followed by Swern oxidation provides the diketone which is then condensed with the diamine to form the pyrazine containing triptycenes 1H and 2H. Purification of the intermediates in this last sequence by silica gel column chromatography was not feasible as the silica catalyzed a formal retro-Diels–Alder reaction giving the substituted anthracene as the only isolated product. Crude reaction products after the initial Diels–Alder reaction were carried forward, including to the subsequent radical formation, without the need for purification *via* chromatography. Additionally, reducing light exposure provided higher purity intermediates. Formation of the radical precursor 3H of the non-bridged species was achieved *via* a Suzuki–Miyaura cross-coupling reaction with phenyl boronic acid and pyBTM′-Cl. All new compounds were fully characterized with 1D and 2D nuclear magnetic resonance (NMR) experiments (Fig. S77–S134) as well as high-resolution mass spectrometry (Fig. S1–S8).

Formation of the final radical species 1, 2, and 3 from 1H, 2H, and 3H, respectively, was achieved by deprotonating the trityl proton with tetrabutylammonium hydroxide to produce deep purple anionic intermediates that are then oxidized with excess silver hexafluoroantimonate. The final radical species 1, 2, and 3 were all stable under column chromatography conditions on neutral alumina allowing for their purification as dark yellow solids for subsequent studies. No obvious degradation was observed for either compound in the solid state when stored at room temperature, but they degraded in air saturated toluene solutions with a half-life of 4.2 days for 1 and 6.5 days for 2 (Fig. S53 and S54). To prevent any degradation, the radical compounds were stored in the solid state at −40 °C in air and solutions were stored at −80 °C in air when not in use for experiments. ^1^H-NMR spectra of both 1 (Fig. S102) and 2 (Fig. S128) in deuterated dichloromethane show broad resonances characteristic of paramagnetic species. The observable resonances in the ^1^H-NMR spectra of compound 1 are attributed to the phenyl ring on the triptycene that is not bonded to the trityl radical, the bridgehead protons, and the protons for the pyrazine moiety. In contrast, the ^1^H-NMR spectrum of compound 2 only shows observable resonances for the bridgehead protons and the pyrazine moiety, while 3 was ^1^H-NMR silent (Fig. S134). Matrix-assisted laser desorption ionization time of flight (MALDI-TOF) mass spectrometry further confirmed conversion of compound 1H to 1 (Fig. S2 and S3) and 2H to 2 (Fig. S7 and S8), and direct analysis in real time (DART) mass spectrometry confirmed the conversion of 3H to 3. Thermogravimetric analysis (Fig. S9 and S10) under a nitrogen atmosphere of 1 and 2 gave thermal degradation temperatures at a 5% weight loss of *T*_d,5_ = 291 °C and *T*_d,5_ = 300 °C, respectively, characteristic of the high thermal stability of triptycene compounds. In comparison, a similar thermal analysis of compound 3 (Fig. S11) gave a more depressed thermal degradation temperature at a 5% weight loss of *T*_d,5_ = 227 °C.

### Cyclic voltammetry and spectroelectrochemistry

**Fig. 3:**
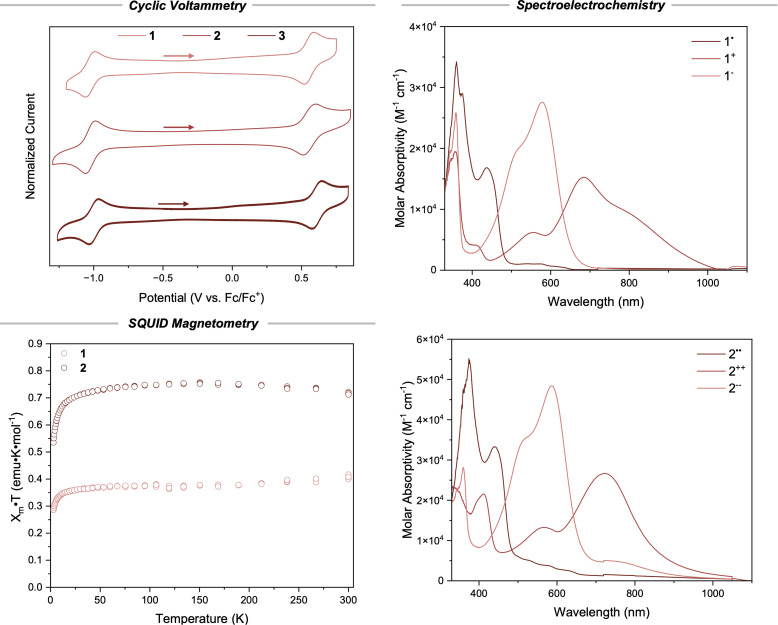
*Cyclic voltammetry*: cyclic voltammogram of 1 (0.1 mM), 2 (0.1 mM), and 3 (0.1 mM) in DCM. Supporting electrolyte [nBu_4_N][PF_6_] (0.1 M), glassy carbon working electrode, platinum mesh counter electrode, Ag/Ag^+^ (3 M aq. KCl) reference electrode. Scan rate: 100 mV s^−1^. *Spectroelectrochemistry*: UV-vis-NIR spectroscopy of *in situ* electrochemically reduced and oxidized 1 (top) and 2 (bottom) in DCM. *Squid magnetometry*: experimental data of the *X*_m_*T vs. T* SQUID measurements for 1 and 2.

To provide further confirmation of the successful formation of monoradical and biradical species, cyclic voltammetry and spectroelectrochemistry were performed in dichloromethane (Fig. 3). Compounds 1, 2, and 3 display amphoteric redox behavior characteristic of organic radicals. Compound 1 has reversible redox waves centered at *E*^ox^^1^ = 0.56 V and *E*^red^^1^ = −1.03 V (Fig. S12). Despite containing two radicals, 2 only exhibited two redox waves at very similar potentials to compound 1 (Fig. S13). At a scan rate of 100 mV s^−1^, the peak-to-peak separation of the oxidation wave for 1 is 65.4 mV and the reduction wave is 69.5 mV, slightly higher than the expected value of 59.2 mV for a fully reversible 1-electron redox event. In contrast, at the same scan rate, the reduction voltammogram of compound 2 displays a separation of 91.9 mV and the oxidation event has a separation of 71.6 mV – both of which are substantially larger than the theoretical value of 29.6 mV for a 2-electron redox event (Table S1). In comparison to 1 and 2, the redox waves for 3 are centered at slightly higher potentials (*E*^ox^^3^ = 0.61 V and *E*^red^^3^ = −1.00 V) indicating a small degree of electronic differences between the bridged and non-bridged species.

To confidently assign the oxidation and reduction waves as 2-electron redox events for 2, we turned to UV-vis-NIR spectroelectrochemistry (Fig. 3). Reduction of compound 1 at a slight overpotential provided the monoanion 1^−^ with a new lambda *λ*^red^_max_ = 597 nm, and oxidation of a fresh solution of 1 provided the monocation 1^+^ (*λ*^ox^_max_ = 684 nm) with absorption tailing into the near infrared region. Applying the same technique to compound 2 yielded qualitatively similar spectra (*λ*^red^_max_ = 598 nm, *λ*^ox^_max_ = 735 nm) indicative of the formation of a dianionic species 2^−−^ and a dicationic species 2^++^, respectively. These results suggest that the oxidation and reduction events observed in the cyclic voltammogram of 2 are indeed 2-electron redox events, or more likely, each observed reduction and oxidation event is two 1-electron events that have very similar redox potentials, indicating weak coupling between the two radical centers in 2.

### SQUID magnetometry

To further validate the quantity of radicals present in each compound and gain insight into the ground state electronic configurations in the crystalline state, we measured the temperature dependence of their magnetic susceptibility with a superconducting quantum interference device (SQUID) (Fig. 3). At temperatures above 50 K, polycrystalline 1 exhibits a roughly constant *χ*_m_*T* close to 0.375 emu K mol^−1^, the theoretical value for an isolated single independent spin. The susceptibility decreases continuously with increasing temperature and no obvious local maximum is observed, indicating that intermolecular coupling between *S* = ½ monoradicals is relatively weak. Two limiting models were considered (1) a one-dimensional (1-D) *S* = ½ Heisenberg chain and (2) pairs of *S* = ½ radicals (dimers). Plots of *χ*_m_*T vs. T* and *χ*_m_*vs. T* both fit reasonably well to the 1-D chain model (Fig. S15) giving a small coupling constant of *J*′/*k* = 0.68 ± 0.08 K. The dimer model gave poorer fits than the 1-D chain for compound 1 (Table S2). Similar results were obtained for polycrystalline 3 (Fig. S17, Table S4) which indicates that both 1 and 3 indeed possess a single *S* = ½ spin.

Polycrystalline 2 exhibits a plateau at around 0.75 emu K mol^−1^ above 50 K as determined based on the *χ*_m_*T vs. T* plot (Fig. 3), which is the theoretical value for two isolated *S* = ½ spins. This is self-consistent with the density of spins in compound 2 being roughly twice that of compounds 1 and 3. Fitting a 1-D *S* = ½ Heisenberg chain to *χ*_m_*T vs. T* and *χ*_m_*vs. T* data provided a value for 2*J*/*k* = 1.12 ± 0.10 K indicating a negligible singlet–triplet energy gap for the ground state with Δ*E*_ST_ ≈ 0.002 kcal mol^−1^ (Fig. S16). This small coupling value agrees well with the cyclic voltammetry studies and indicates that the radical moieties in compound 2 behave more like independent radical centers than a true biradicaloid molecule. In other words, the redox events for compound 2 are not highly coupled and occur through a stepwise 1-electron oxidation (or reduction) scheme. Concurrently, the voltage required to oxidize 2 to 2^+^˙ should not be significantly lower in energy than the voltage required to oxidize 2^+^˙ to 2^++^. Hence, redox events in the CV of compound 2 appear as 2-electron processes instead of two sequential 1-electron processes that would be anticipated for coupled radicals. Consistently, fitting the SQUID data of 2 to the dimer (biradical) model gives slightly inferior results (Table S3). Taken together, the CV, spectroelectrochemistry, and SQUID data confirm 1 and 3 as monoradical species and 2 as a biradical species.

### Continuous wave and pulsed electron paramagnetic resonance (EPR)

Continuous-wave (CW) EPR spectra of biradical 2 do not exhibit clearly resolved splittings that could be assigned to electron–electron coupling (Fig. S18), and the spectra of 2 are qualitatively similar to those of 1 and 3 (Fig. S18). Pulsed EPR experiments were therefore employed to further investigate the relaxation (Fig. S19–S21) and spin–spin interactions in 2 and to compare its behavior directly to the monoradical reference 1.

**Fig. 4:**
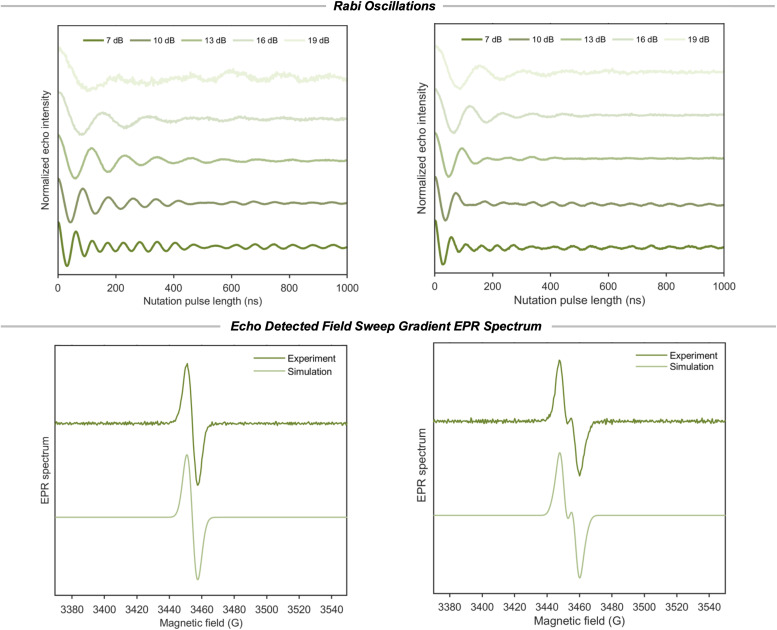
*Rabi oscillations*: nutation frequency at different microwave powers for compound 1 (left) and 2 (right). *Echo detected field sweep gradient EPR spectrum*: experimental and simulated first derivatives of the echo detected field sweep gradient at the X-band and 70 K for compound 1 (left) and 2 (right).

Nutation experiments reveal a clear and reproducible distinction between 1 and 2 (Fig. 4). Under identical microwave power conditions, 2 consistently exhibits a higher nutation frequency than the monoradical reference 1. This observation demonstrates that on the timescale of the excitation pulse, the two unpaired electrons do not behave as fully independent spins but instead exhibit a degree of correlated response to the microwave field (Fig. S22). However, the *υ*_1_/*B*_1_ ratio remains below that expected for a strongly exchange-coupled triplet state, and no distinct half-field (Δ*m*_s_ = 2) transition is observed in CW experiments, indicating that the system does not form a rigid triplet manifold.

Further insight into the nature of the interaction is obtained from echo-detected field sweep (EDFS) measurements (Fig. 4). Compared to the monoradical 1, biradical 2 exhibits a subtle spectral splitting that becomes apparent only in the gradient of the EDFS. This weak feature is difficult to resolve in CW EPR spectra, where saturation effects obscure small electron–electron interactions. The splitting can be reproduced by spectral simulations incorporating an electron–electron dipolar coupling of approximately 17.5 MHz and an isotropic *g* value of 2.0052. We note that relating this coupling to a specific electron–electron separation depends on simplified models and is therefore not uniquely defined.

Double quantum coherence (DQC) experiments on 2 (Fig. S23 and S24) were also performed to further probe the electron–electron interaction.^37^ The DQC analysis using Tikhonov regularization suggests the presence of distance components below approximately 2 nm. However, the distribution does not exhibit well-constrained fits, with the L curve (Fig. S24) showing a less pronounced corner and the major peak appears below 1.5 nm. The limitation of this analysis arises from several factors. First, the inferred electron–electron separation is already close to the lower distance limit for which DQC has been successfully applied previously,^38–40^ wherein a molecule starts to fall into the strong-coupled regime. Secondly, at X-band frequencies, the presence of electron spin echo envelope modulation (ESEEM) contributions from ^1^H and ^35^Cl (Fig. S25) complicates a quantitative distance analysis, because hyperfine coupling with these nuclei makes it challenging to analyze the electron–electron dipolar coupling.

### Steady state optical spectroscopy

**Fig. 5:**
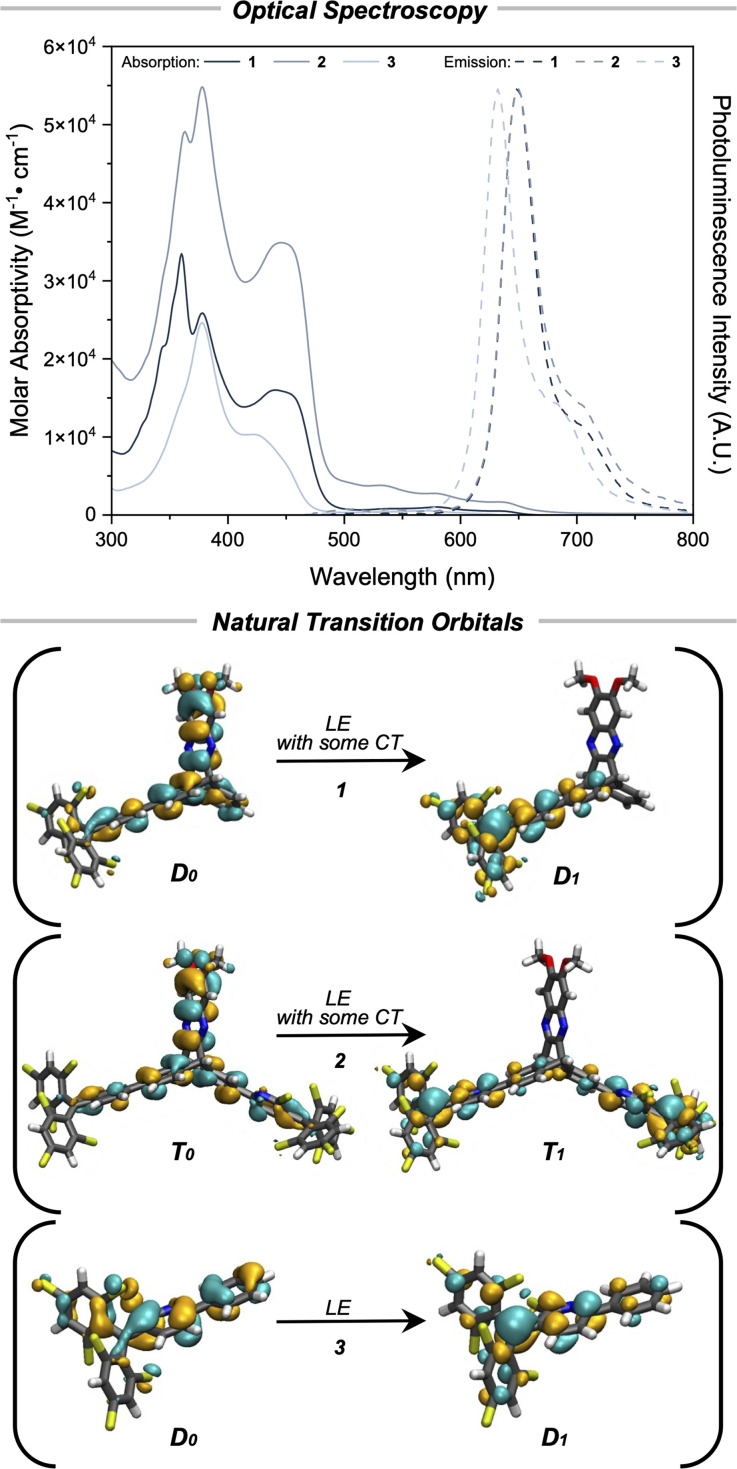
*Optical spectroscopy*: UV-vis spectroscopy of the absorption and emission (*λ*_ex_ = 450 nm) for 1 and 2 and (*λ*_ex_ = 430 nm) for 3 in PhMe. *Natural transition orbitals*: natural transition orbitals for the lowest energy doublet transition (1, top), triplet transition (2, middle), and doublet transition (3, bottom) at the UB3LYP-D3(BJ)/def2-SVP/TDDFT, CPCM(toluene) level of theory. Isosurface = ±0.03 e a_0_^−3^.

Fig. 5 shows the electronic absorption spectra of compounds 1, 2, and 3 in toluene at room temperature. When compared to monoradical 1, the spectrum of biradical 2 is nearly twice as large as that of the monoradical, typical of weakly coupled radical dimers,^33,41–43^ and this further corroborates the treatment of 2 as two independent radicals. There is also a clear difference in the line-shape of the absorption spectra of 3 compared to 1 and 2, indicating an electron difference between the non-bridged and bridged species. No obvious symmetry broken charge-transfer band of the S_0_ → S_1_ transition was observed in the absorption spectra of 2. As explained elsewhere,^33^ to observe a singly occupied molecular orbital (SOMO) to singly unoccupied molecular orbital (SUMO) transition in a biradical, the two SOMOs must have sufficient overlap to create a transition dipole. According to calculations (*vide infra*), the trityl radicals are not sufficiently delocalized onto the aryl rings on the triptycene wings to allow for inter-radical homoconjugation at the sp^3^ bridgehead carbon. In other words, the SOMOs for the singlet state are not sufficiently delocalized for emission from the symmetry broken charge transfer state S_0_ → S_1_ to be observable.

The photoluminescence emission spectra of 1 and 2 (Fig. 5) are essentially identical with a *λ*^em^_max_^1^^,^^2^ = 648 nm for both compounds in toluene. In comparison, there is a hypsochromic shift of 18 nm in the emission spectra of 3 giving *λ*^em^_max_^3^ = 630 nm. This difference further indicates that weak electronic communication is imparted by the inclusion of the [2.2.2] bridging group. The photoluminescence quantum yields, *ϕ*_PL_, for both 1 and 2 are 1.4% in toluene, which suggests minimal nonradiative contributions from the second pyBTM′ group in 2 (Table S5). Photoluminescence lifetimes in air saturated toluene for 1, 2, and 3 were 4.9 ns, 4.8 ns, and 6.4 ns, respectively (Fig. S50–S52). Photoluminescence spectra in solvents of varying polarity show negligible shifts in the peak emission, indicating negligible charge transfer (CT) character in all 3 compounds (Fig. S26–S49).

### Theoretical calculations

Geometries were optimized using density functional theory (DFT) at the UB3LYP-D3BJ/def2-SVP (CPCM, toluene) level of theory, utilizing a broken symmetry (BS) calculation for the singlet state of 2 (Fig. S55, Tables S11–S14). Spin density maps for 1, 2, and 3 (Fig. S56) indicate that the majority of the spin lies on the central trityl carbon atom with delocalization onto the three trityl aryl rings and minimal delocalization to the triptycene (phenyl in the case of 3) aryl rings. Fractional occupation density (FOD) analyses support significant multireference character (Fig. S57, Table S6) as anticipated for trityl radicals. Therefore, complete active space self-consistent field (CASSCF) calculations (Fig. S58 and S59) were performed to identify the singlet–triplet energy gap (Δ*E*_ST_ ≡ 2*J*) of compound 2. An active space of 10 electrons and 10 orbitals (CASSCF(10,10)/def2-SVP) was employed and gave a negligible Δ*E*_ST_ of approximately 0.04 kcal mol^−1^, agreeing with the results from SQUID measurements (*vide supra*).

Time dependent DFT (TD-DFT) results (UB3LYP-D3BJ/def2-SVP, CPCM(toluene)) indicate that the lowest energy transition D_0_ → D_1_ for compound 1 corresponds to excitation of an electron located on the donor moiety to the unoccupied radical orbital (Fig. S60, Table S7). Similar results are observed for the T_0_ → T_1_ state of compound 2 (Fig. S62, Table S9). The symmetry broken charge-transfer state S_0_ → S_1_ was calculated to have a negligible oscillator strength on the order of 10^−2^, explaining the lack of a low-energy, spin-specific absorption band in the UV-vis spectra. Stated differently, the symmetry broken charge-transfer state is effectively disallowed because of low orbital overlap (Fig. S61, Table S8). In comparison to 1 and 2, TD-DFT results for 3 at the same level of theory indicate that the lowest energy transition D_0_ → D_1_ for this compound is an excitation of an electron that is delocalized across all of the aryl rings in the system (Fig. S63, Table S10). Assuming Kasha's rule for the emissive species, electron–hole analysis (Fig. S64–S69) of D_0_ → D_1_ for 1 and T_0_ → T_1_ for 2 of the Franck-Condon state indicates weak charge transfer character with the electron localizing on the trityl moiety and the hole localized on the donor. However, there was also significant hole–electron contribution from the aryl rings on the triptycene wings, indicating a non-negligible amount of locally excited character. Analysis of the natural transition orbitals (NTOs) for 1 and 2 showed significant overlap at this position between the ground and excited state orbitals indicating a significant degree of locally excited character (Fig. 5). In contrast, almost the entirety of both the electron and hole contribution of D_0_ → D_1_ for 3 lies on the trityl aryl rings (Fig. S70 and S71), indicating that this transition contains entirely locally excited character. This conclusion is further supported *via* analysis of the NTOs of 3 (Fig. 5).

Overall, the donor orbitals for 1 and 2 appear to be localized on the pyrazine moiety with significant homoconjugation onto the aryl wings of the triptycene. As a proof of principle, these results indicate that through-space donor–acceptor conjugation strategies can feasibly be applied to emissive radical compounds. Judicious molecular design and improved matching between the donor and acceptor strengths^17^ are anticipated to provide facile access to a new class of high-performance emissive radical species.

## Conclusions

We have synthesized and characterized three new emissive radical species. Cyclic voltammetry, spectroelectrochemistry, and SQUID magnetometry confirm the presence of one *S* = ½ spin in 1 and 3 and two practically independent spins in 2. The results from pulsed EPR studies further corroborate the identity and spin states of these compounds. Optical spectroscopy experiments indicate that the emission event is predominantly locally excited in character for 1 and 2, and theoretical calculations explain this observation as a result of extensive homoconjugation of the donor moiety through a [2.2.2] bridge. Going forward, the design options for luminescent triarylmethyl radicals need to be expanded with other donor or acceptor motifs to further modify luminescent properties of emissive radical species.

## Author contributions

A. R. D. and T. M. S. conceived the idea. A. R. D. performed the synthesis, electrochemistry, magnetometry, steady-state measurements, calculations, and wrote the original draft. Y. Z. and R. G. G. performed and analyzed the EPR measurements. R. N. S. performed degradation studies. C. H. M. performed the lifetime fluorimetry measurements. All authors discussed the results and their interpretation. The manuscript was approved by all authors.

## Conflicts of interest

There are no conflicts to declare.

## Supplementary Material

SC-OLF-D6SC00981F-s001

## Data Availability

The data supporting this article have been included as part of the supplementary information (SI). Supplementary information: synthetic procedures, MALDI-TOF, thermal gravimetric analysis, cyclic voltammetry, SQUID magnetometry, electron paramagnetic resonance, optical spectroscopy, computational details, and nuclear magnetic resonance spectra. Additional references are also included. See DOI: https://doi.org/10.1039/d6sc00981f.
