# Supplementary material for: Through-space donor–acceptor homoconjugation strategies for emissive radical species
Source: Chem Sci. 2026 Apr 17;17(21):10511–8. doi: 10.1039/d6sc00981f (PMC13089764; doi:10.1039/d6sc00981f)
Supplement: SC-017-D6SC00981F-s001 [file SC-017-D6SC00981F-s001.pdf]

Supplementary Information (SI) for Chemical Science.
This journal is © The Royal Society of Chemistry 2026

Supporting Information for

Through Space Donor-Acceptor Homoconjugation Strategies for Emissive Radical Species

Ashton R. Davis,[a] Yujie Zhao,[a] Robert N. Sansone,[a,b] Colleen H. McAloon,[a] Robert G. Griffin, *[a] and Timothy M. Swager*[a]

[a] *Department of Chemistry, Massachusetts Institute of Technology, 77 Massachusetts Avenue, Cambridge, Massachusetts 02139, United States*

[a] *Department of Materials Science and Engineering, Massachusetts Institute of Technology, 77 Massachusetts Avenue, Cambridge, Massachusetts 02139, United States*

*Corresponding Author's Email Address:
R.G.G: rgg@mit.edu
T.M.S.: tswager@mit.edu

Table of Contents

MATERIALS AND METHODS 3

SYNTHETIC PROCEDURES 5

MALDI-TOF 13

THERMAL GRAVIMETRIC ANALYSIS 17

CYCLIC VOLTAMMETRY 19

SQUID MAGNETOMETRY 21

ELECTRON PARAMAGNETIC RESONANCE 25

OPTICAL SPECTROSCOPY 32

COMPUTATIONAL DETAILS 39

NUCLEAR MAGNETIC RESONANCE SPECTRA 62

MATERIALS AND METHODS

Unless otherwise noted, all reactions were carried out under argon atmosphere using standard Schlenk or glovebox techniques. Anhydrous acetonitrile was prepared using a solvent purification system via the method of Grubbs.[1] Tetrahydrofuran was distilled from the ketyl radical under argon, degassed via four freeze, pump, thaw cycles, and stored in the glovebox over activated 4 Å molecular sieves until needed. 2,6-bis(4,4,5,5-tetramethyl-1,3,2-dioxaborolan-2-yl)anthracene, [2] (2,6-dichloro-3-pyridyl)bis(2,4,6-trichlorophenyl)methane (pyBTM'-Cl), [3] and 1,2-diamino-4,5-dimethoxybenzene [4] were prepared according to previously reported procedures. All other reagents and solvents were purchased from commercial suppliers (Sigma Aldrich, Ambeed, VWR, Oakwood, TCI America, ThermoFisher, Strem) and used without further purification. Flash chromatography was performed using a CombiFlash® Rf+ system with RediSep® Rf Silica gel columns (230–400 mesh) using a proper eluent system or was carried out manually on neutral alumina with a Brockmann Grade of I (Oakwood) or II (Alfa Aesar) as specified.

^1H and ^{13}C and spectra were recorded on a Bruker Avance Neo spectrometer operating at 500 MHz or a Bruker Avance III HD 400 MHz. ^1H and ^{13}C spectra were referenced using residual solvent as an internal reference ($CHCl_3$: δ 7.26 ppm and δ 77.4 ppm, respectively | CH_2Cl_2 δ 5.32 ppm for ^1H spectra). Peaks and their apparent multiplicities are reported as (s = singlet, d = doublet, t = triplet, q = quartet, m = multiplet or unresolved, coupling constant(s) in Hz, integration).

High-resolution mass spectrometry (HRMS) was performed at the Mass Spectrometry Laboratory within the MIT Department of Chemistry Instrument Facilities using a JEOL S4 AccuTOF 4G LC-plus equipped with a DART source or a matrix assisted laser desorption/ionization time-of-flight (MALDI-TOF) spectrometer. MALDI-TOF mass spectra were collected using trans-2-[3-(4-tert-butylphenyl)-2-methyl-2-propenylidene]malononitrile, 1,8,9-trihydroxyanthracene, or 2,5-dihydroxybenzoic acid as the matrix and were referenced to an internal standard.

Cyclic voltammetry (CV) was conducted on a BioLogic SP-150 potentiostat. The experiments were carried out under an argon atmosphere in degassed and anhydrous methylene chloride solution containing $^nBu_4N[PF_6]$ (0.1M). The setup consisted of a StonyLab® 3mm glassy carbon disk working electrode, a platinum mesh as the counter electrode, and a silver wire immersed in aqueous 3 M KCl solution. The CV data have been referenced to the external standard Fc/Fc^+ (ferrocene/ferrocenium) redox couple. Spectroelectrochemistry experiments were conducted on a BioLogic SP-150 potentiostat and a Cary 7000-UMS UV-Vis-NIR spectrometer under air. Experiments were performed in a Pine Research spectroelectrochemical cuvette with a fitted platinum honeycomb electrode card and a silver wire immersed in a 0.01 M $AgNO_3$ / 0.1 M $^nBu_4N[PF_6]$ solution in acetonitrile as the reference electrode. The same solutions used for CV studies were used for the spectroelectrochemical ones. An oxidative potential of 0.65 V and a reductive potential of -1.1 V (both vs. Fc/Fc^+), slight overpotentials compared to the center of the redox event in CV, were applied for 5 minutes before the spectra was taken. Fresh samples were taken from the CV solutions for oxidative or reductive measurements.

---

[1] Pangborn, A. B.; Giardello, M. A.; Grubbs, R. H.; Rosen, R. K.; Timmers, F. J. Safe and Convenient Procedure for Solvent Purification. *Organometallics* **1996**, *15* (5), 1518-1520.

[2] Takaki, Y.; Yoza, K.; Kobayashi, K. Fourfold C–H Borylation of Anthracene: 1,3,5,7-Tetraborylanthracene and Its Application to 1,3,5,7-Tetraarylanthracenes. *Chem. Lett.* **2017**, *46* (5), 655-658.

[3] Hattori, Y.; Kimura, S.; Kusamoto, T.; Maeda, H.; Nishihara, H. Cation-responsive turn-on fluorescence and absence of heavy atom effects of pyridyl-substituted triarylmethyl radicals. *Chem. Commun.* **2018**, *54* (6), 615-618.

[4] Pike, Jay D.; Rosa, Dell T.; Coucouvanis, D. Lipophilic Metal−Salicylideneimine−Crown Ether Hybrids — Ditopic Carriers in the Facilitated Transport of Amphiphilic Molecules Across Bulk Liquid Membranes. *Eur. J. Inorg. Chem.* **2001**, *2001* (3), 761-777.

Thermogravimetric analysis (TGA) of **1** and **2** was conducted using a Discovery TGA 5500 from TA Instruments under nitrogen atmosphere. The measurement was carried out using a heating ramp speed of 15 °C per minute up to 800 °C. The reported degradation temperatures $T_{d,5}$ refers to the temperature at which the sample loses 5% of its initial mass.

Magnetic studies were carried out using a Quantum Design Inc® Magnetic Properties Measurement System (MPMS-3) at an applied magnetic field H = 10000 Oe and T = 3 - 300 K in vibrating sample mode. Samples were loaded into polycarbonate powder sample holders mounted on a brass sample holder as described in detail in the dedicated SQUID section of the supporting information. Samples were cooled to 3 K at zero field before applying the field of 10000 Oe and starting the measurement in warming mode. The measurement of magnetic moment at a given temperature was repeated in triplicate. The procedure for background correction and formulas/models used for fitting the data is also described in the dedicated SQUID section of the supporting information.

X-band EPR experiments were carried out at 0.34T on a Bruker ElexSys E580 X-band spectrometer. Samples were loaded into 4 mm quartz EPR tube and positioned in an EN4118X-MD5 resonator for both continuous wave (CW) and pulsed measurements. Temperature control was achieved using a ColdEdge Stinger 4K system combined with an Oxford CF935 flow cryostat and an Oxford IT503 temperature controller. CW and pulsed EPR measurements were performed on **1** and **2** dissolved in toluene at spin concentrations of approximately 100 μM. CW EPR experiments were performed at room temperature to resolve hyperfine couplings. At low temperature, echo-detected field-sweep (EDFS) measurements were preferred, since the CW spectra were easily saturated and fine shoulders and splittings in the lineshape could no longer be reliably resolved. Additional pulsed EPR experiments were carried out to relaxation parameters and nutation frequencies. Pulsed experiments are described in greater detail in the electron paramagnetic resonance section of this document.

UV-Vis absorption spectra were recorded on a Cary 5000 spectrophotometer and corrected for background signal with a solvent filled quartz cuvette. Fluorescence measurements were performed using a Horiba Quanta-ϕ fluorescence spectrophotometer, using right angle detection, and corrected for background using a solvent filled quartz cuvette. All samples were held in 1 cm square quartz cuvettes. Photoluminescent quantum yield, ϕ_{PL}, measurements were performed on a Quantaurus-QY absolute PL quantum yield spectrometer C11347 with a 450 nm excitation wavelength. Fluorescence lifetime data was collected using an Edinburgh Instruments FS5 Spectrofluorometer. A suspension of Ludox (colloidal silica) in deionized water was used to measure the instrument internal response factor (IRF). Lifetime fitting was performed using the Fluorophore software with χ^2 values between 0.8-1.5 with the smallest number of components being used for each fit.

Computational details are described in the computation section of the supporting information.

SYNTHETIC PROCEDURES

Synthesis of 6-(anthracen-2-yl)-3-(bis(2,4,6-trichlorophenyl)methyl)-2-chloropyridine

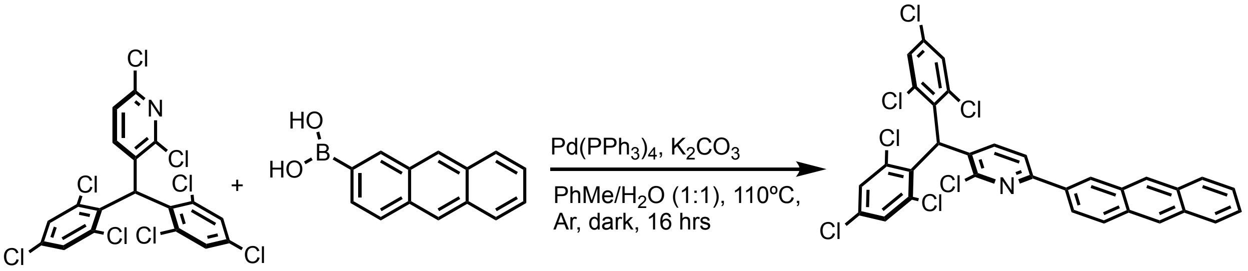

A 50 mL Schlenk flask was charged with anthracen-2-ylboronic acid (0.44 g, 1.98 mmol, 1 eq.), (2,6-dichloro-3-pyridyl)bis(2,4,6-trichlorophenyl)methane (1.25 g, 2.40 mmol, 1.2 eq.), K_2CO_3 (1.65 g, 11.9 mmol, 6 eq.), 12 mL of toluene, 12 mL of water, and a magnetic stir bar. The mixture was then degassed by bubbling argon through the suspension for 5 minutes, and then $Pd(PPh_3)_4$ (230 mg, 0.20 mmol, 0.1 eq.) was added. The flask was sealed and further degassed via three freeze, pump, thaw cycles. After the final thaw, the flask was protected from light with aluminum foil and heated to 110 ºC with stirring for 16 hours in an oil bath. Once the reaction was complete, the flask was removed from the oil bath and allowed to cool to room temperature. Then 40 mL of saturated aqueous NH_4Cl was added and the organic layer was extracted with DCM (3 x 30 mL). The organic layers were collected, dried with Na_2SO_4, and the solvent was removed via rotary evaporation. The residue was triturated with hexanes and the solid was filtered and dried to give 1.05 g (80%) of a pale-yellow powder.

^{1}H NMR (500 MHz, $CDCl_3$) δ 8.72 (s, 1H), 8.54 (s, 1H), 8.44 (s, 1H), 8.14 (d, *J* = 9.0 Hz, 1H), 8.10 (d, *J* = 8.9 Hz, 1H), 8.05 – 8.00 (m, 2H), 7.78 (d, *J* = 8.0 Hz, 1H), 7.52 – 7.46 (m, 2H), 7.38 (s, 4H), 7.30 (d, *J* = 8.0 Hz, 1H), 6.67 (s, 1H).
^{13}C{^{1}H} NMR (126 MHz, $CDCl_3$) δ 156.6, 151.5, 140.3, 137.5, 134.2, 134.0, 133.5, 132.5, 132.2, 131.9, 131.5, 131.3, 129.7, 129.1, 128.5, 128.4, 127.8, 127.3, 126.3, 126.1, 125.8, 123.8, 119.2, 49.2.
HRMS (AccuTOF-DART+) calculated for $C_{32}H_{17}Cl_7N$ $[M+H]^+$: 659.9175, found 659.9208.

Synthesis of 2-(5-(bis(2,4,6-trichlorophenyl)methyl)-6-chloropyridin-2-yl)-9,10-dihydro-9,10-[4,5]epidioxoloanthracen-13-one

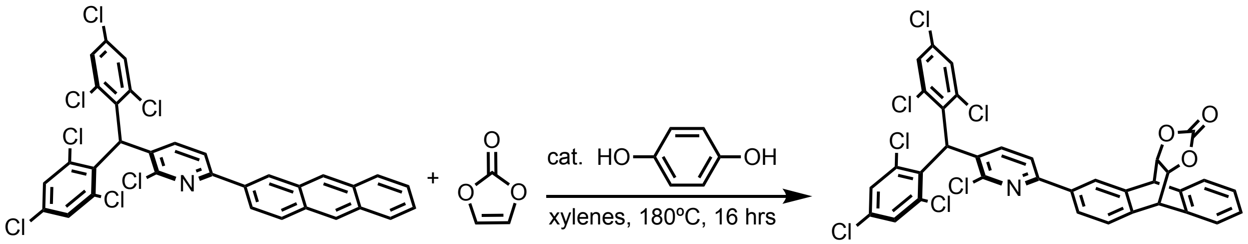

A 15 mL flame-dried pressure vessel was charged with 6-(anthracen-2-yl)-3-(bis(2,4,6-trichlorophenyl)methyl)-2-chloropyridine (0.50 g, 0.75 mmol, 1 eq.), vinylene carbonate (2.33 g, 27.0 mmol, 36 eq.), trace hydroquinone (spatula tip), 5 mL of xylenes, and a magnetic stir bar. The vessel was sealed with a rubber stopper and argon was bubble through the suspension with stirring for 10 minutes. Then the rubber stopper was swapped for the cap to the vessel, and the vessel was sealed. The vessel was wrapped with aluminum foil to protect the reaction from light, and it was heated to 180 ºC with stirring for 16 hours. After the reaction was complete, the vessel was allowed to cool to room temperature before the dark brown solution was transferred to a 50 mL round bottom flask. The volatile compounds were removed via short path vacuum distillation. *Technical note:* vacuum should be introduced slowly to avoid bumping, ensuring the boiling flask is of sufficient volume also helps to alleviate this issue. The product was purified by automated flash column chromatography eluting with chloroform on silica gel affording the title compound as a white powder (483 mg, 85%) as a mixture of diastereomers.

^{1}H NMR (500 MHz, $CDCl_3$) δ 8.15 (d, *J* = 7.3 Hz, 1H), 7.84 (dd, *J* = 13.8, 7.8 Hz, 1H), 7.55 (dd, *J* = 16.1, 8.1 Hz, 1H), 7.47 (d, *J* = 7.8 Hz, 1H), 7.42 – 7.38 (m, 2H), 7.35 (d, *J* = 3.2 Hz, 4H), 7.25 – 7.19 (m, 4H), 6.62 (d, *J* = 3.9 Hz, 1H), 4.97 – 4.87 (m, 2H), 4.82 (s, 1H), 4.76 (s, 1H).

^{13}C{^{1}H} NMR (126 MHz, $CDCl_3$) δ 156.1, 155.8, 154.2, 154.1, 151.4, 151.3, 140.4, 140.2, 139.4, 138.7, 138.1, 137.6, 137.4, 137.4, 137.1, 136.7, 136.1, 135.9, 134.2, 134.1, 134.1, 133.4, 133.2, 131.7, 131.4, 129.7, 129.6, 128.1, 128.1, 128.0, 128.0, 127.1, 126.8, 126.8, 126.2, 126.1, 125.9, 125.8, 125.4, 124.5, 119.1, 119.0, 76.4, 76.3, 76.2, 49.1, 49.1, 48.0, 47.7.
HRMS (MALDI-TOF+) calculated for $C_{35}H_{19}Cl_7NO_3$ $[M+H]^+$: 745.9185, found 745.9199.

Synthesis of 2-(5-(bis(2,4,6-trichlorophenyl)methyl)-6-chloropyridin-2-yl)-9,10-dihydro-9,10-ethanoanthracene-11,12-diol

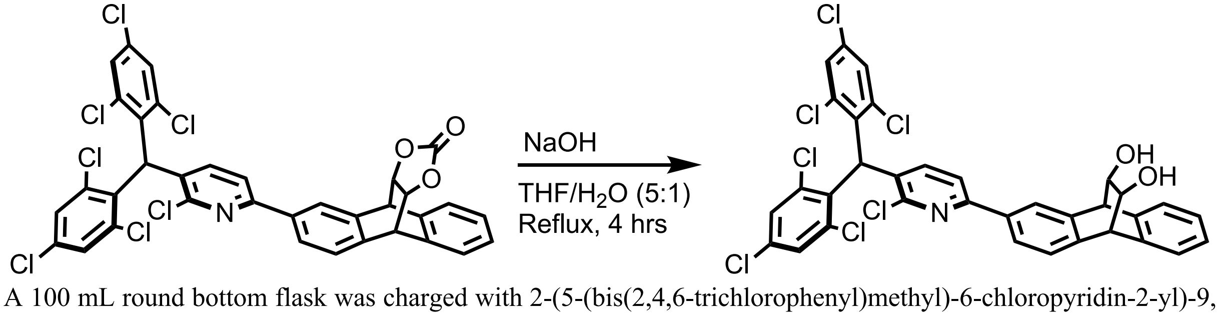

A 100 mL round bottom flask was charged with 2-(5-(bis(2,4,6-trichlorophenyl)methyl)-6-chloropyridin-2-yl)-9,10-dihydro-9,10-[4,5]epidioxoloanthracen-13-one (470 mg, 0.63 mmol), 47 mL tetrahydrofuran, 10 mL of 4 M aqueous NaOH, and a magnetic stir bar. The mixture was refluxed for 4 hours at 90 ºC with protection from light before cooling to room temperature and pouring into 50 mL H_2O. The water was then extracted with DCM (2 x 50 mL). The organic layers were combined and dried with Na_2SO_4. The solvent was decanted, and the solvent was removed via rotary evaporation to give the title compound as an off-white solid (410 mg, 91%) as a mixture of diastereomers which was used without further purification.

^{1}H NMR (500 MHz, $CDCl_3$) δ 8.06 (d, J = 18.9 Hz, 1H), 7.78 (dd, J = 28.4, 7.6 Hz, 1H), 7.54 (dd, J = 10.9, 8.2 Hz, 1H), 7.47 – 7.33 (m, 6H), 7.33 – 7.28 (m, 1H), 7.21 (m, 2H), 7.16 (dd, J = 5.2, 3.3 Hz, 1H), 6.62 (s, 1H), 4.55 – 4.42 (m, 2H), 4.08 (s, 2H), 2.46 – 2.34 (m, 1H), 2.31 (dd, J = 16.0, 5.9 Hz, 1H).
^{13}C{^{1}H} NMR (126 MHz, $CDCl_3$) δ 156.7, 156.6, 151.3, 151.2, 141.9, 140.9, 140.2, 139.8, 139.6, 138.5, 138.3, 137.4, 136.0, 135.9, 134.1, 133.4, 131.1, 131.0, 129.6, 127.0, 127.0, 126.7, 126.7, 125.5, 125.4, 125.3, 125.2, 125.0, 124.9, 123.6, 119.0, 118.9, 68.3, 68.2, 68.2, 68.1, 51.6, 51.6, 51.4, 51.4, 49.1.
HRMS (AccuTOF-DART+) calculated for $C_{34}H_{21}Cl_7NO_2$ $[M+H]^+$: 719.9387, found 719.9332.

Synthesis of (9R,10S)-2-(5-(bis(2,4,6-trichlorophenyl)methyl)-6-chloropyridin-2-yl)-9,10-dihydro-9,10-ethanoanthracene-11,12-dione

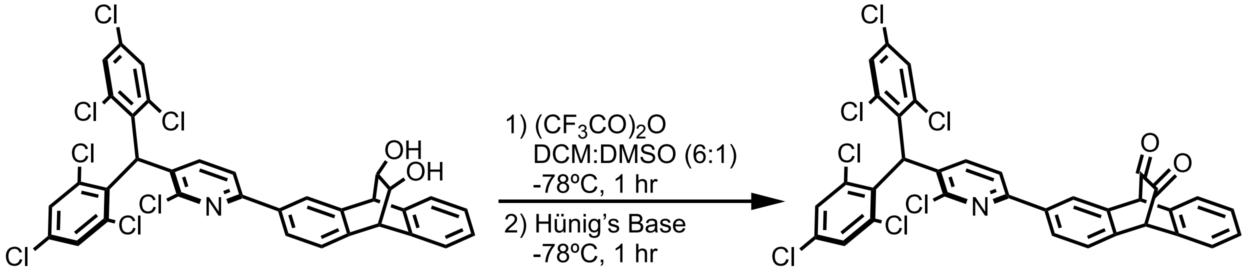

A flamed dried 100 mL round bottom flask was charged with 24 mL anhydrous DCM, 4 mL anhydrous DMSO, and a magnetic stir bar. The flask was sealed with a rubber stopper, cooled to -78 ºC (dry ice, isopropanol), and the contents were degassed with bubbling argon for 10 minutes. Afterwards, the argon line was removed, and the flask was protected from light with aluminum foil. Then, 4 mL of trifluoroacetic anhydride was added via syringe, and the reaction was stirred at -78 ºC for 10 minutes. 2-(5-(bis(2,4,6-trichlorophenyl)methyl)-6-chloropyridin-2-yl)-9,10-dihydro-9,10-ethanoanthracene-11,12-diol (400mg, 0.55 mmol) was dissolved in 4 mL of anhydrous DCM and 4 mL of anhydrous DMSO and was added dropwise to the reaction vessel via syringe. The reaction was allowed to stir at -78 ºC for 1 hour. Then 4 mL of N,N-diisopropylethylamine was added dropwise and the reaction was stirred at -78 ºC for an additional hour before being allowed to warm up to room temperature. Once at room temperature, the light protection was removed and 10 mL of 1 M aqueous HCl was added. The contents of the flask were transferred to a separatory funnel. The aqueous layer was extracted with 50 mL of DCM, and the organic layer was separated. The aqueous layer was back extracted with an additional 25 mL of DCM, and the organic layers were combined and

washed further with water (100 mL) and brine (100 mL) to remove DMSO. The organic layer was dried with Na_2SO_4, decanted, and volatiles were removed via rotary evaporation to give the compound (378 mg, 95%) as a bright yellow solid which was used without further purification.

^{1}H NMR (500 MHz, $CDCl_3$) δ 8.21 (s, 1H), 7.98 (d, *J* = 7.9 Hz, 1H), 7.57 (dd, *J* = 7.9, 4.2 Hz, 2H), 7.50 – 7.47 (m, 2H), 7.39 (dd, *J* = 5.4, 3.2 Hz, 2H), 7.35 (s, 4H), 7.25 (d, *J* = 4.2 Hz, 1H), 6.63 (s, 1H), 5.11 (s, 1H), 5.05 (s, 1H).
^{13}C{^{1}H} NMR (126 MHz, $CDCl_3$) δ 183.6, 155.3, 151.5, 140.5, 138.7, 137.4, 136.4, 135.9, 134.8, 134.6, 134.3, 133.2, 132.1, 129.7, 129.7, 128.0, 126.8, 126.8, 126.5, 125.1, 119.1, 60.2, 60.0, 49.1.
HRMS (AccuTOF-DART+) calculated for $C_{34}H_{17}Cl_7NO_2$ $[M+H]^+$: 715.9074, found 715.9044.

Synthesis of 1H

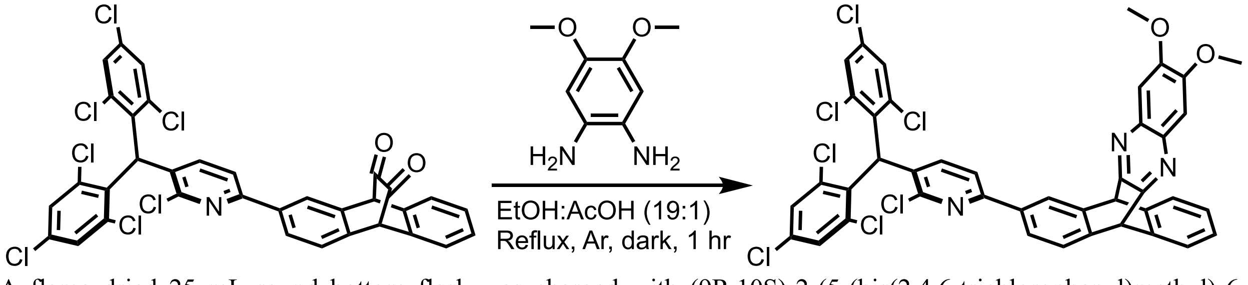

A flame dried 25 mL round bottom flask was charged with (9R,10S)-2-(5-(bis(2,4,6-trichlorophenyl)methyl)-6-chloropyridin-2-yl)-9,10-dihydro-9,10-ethanoanthracene-11,12-dione (200 mg, 0.28 mmol, 1 eq.), 1,2-diamino-4,5-dimethoxybenzene (51.5 mg, 0.30 mmol, 1.1 eq.), 15 mL of a 19:1 mixture of absolute ethanol and acetic acid, and a magnetic stir bar. This suspension was degassed with bubbling argon for 5 minutes before a reflux condenser was attached and the apparatus was protected from light with aluminum foil. The vessel was then refluxed (100 ºC) under argon for 1 hour before cooling to room temperature. The precipitate was filtered, washed with methanol, and dried *in vacuo* to give the compound (128 mg, 54%) as a light-yellow powder.

^{1}H NMR (500 MHz, $CDCl_3$) δ 8.24 (s, 1H), 7.74 (dd, *J* = 7.8, 1.4 Hz, 1H), 7.60 (d, *J* = 7.8 Hz, 1H), 7.57 – 7.49 (m, 3H), 7.34 (s, 4H), 7.26 (s, 2H), 7.19 (d, *J* = 8.0 Hz, 1H), 7.12 (dd, *J* = 5.4, 3.2 Hz, 2H), 6.60 (s, 1H), 5.70 (s, 1H), 5.63 (s, 1H), 3.99 (s, 6H).
^{13}C{^{1}H} NMR (126 MHz, $CDCl_3$) δ 156.2, 155.5, 155.3, 152.1, 151.3, 144.3, 143.6, 142.4, 142.2, 140.2, 137.4, 135.8, 135.8, 135.7, 134.1, 133.3, 131.3, 129.6, 126.8, 126.7, 125.3, 125.3, 125.1, 125.0, 123.6, 118.9, 107.1, 107.0, 56.4, 56.4, 55.5, 55.2, 49.1.
HRMS (MALDI-TOF+) calculated for $C_{42}H_{25}Cl_7N_3O_2$ $[M+H]^+$: 847.9766, found 847.9706.

Synthesis of 1

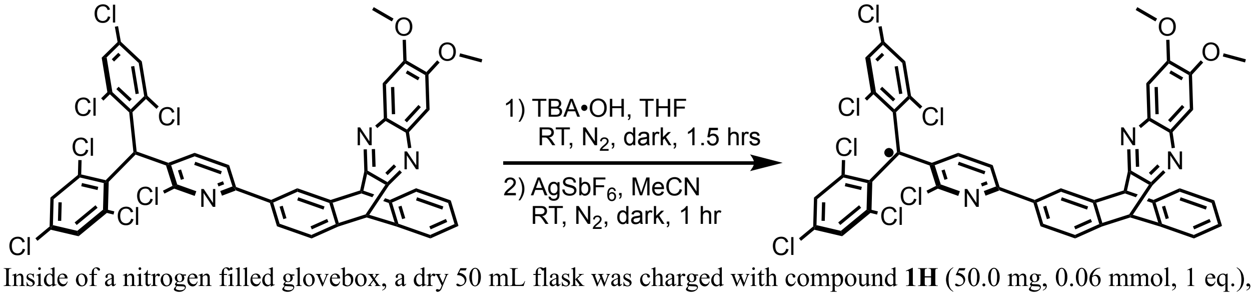

Inside of a nitrogen filled glovebox, a dry 50 mL flask was charged with compound **1H** (50.0 mg, 0.06 mmol, 1 eq.), 20 mL anhydrous THF, and a magnetic stir bar. The vessel was protected from light and tetrabutylammonium hydroxide (1 M in MeOH, 0.19 mL, 0.19 mmol, 3.2 eq.) was added dropwise. The reaction was then allowed to stir for 1.5 hours at room temperature. Silver hexafluoroantimonate (95.0 mg, 0.28 mmol, 4.75 eq.) was dissolved in 25 mL anhydrous MeCN, and this solution was added dropwise to the now deep purple THF solution. The reaction was stirred for an additional hour at room temperature before being removed from the glovebox. The solution was filtered through Celite® (diatomaceous earth) before the solvent was removed via rotary evaporation. Purification by flash chromatography on neutral alumina (Brockmann grade II) eluting with 1:2 hexane:chloroform (R_f = 0.2) afforded **1** as a dark yellow powder (13 mg, 26%).

^{1}H NMR (500 MHz, CD_2Cl_2, 25ºC) δ 8.12 – 7.90 (br, 1H), 7.71 (br, 1H), 7.49 (br, 1H), 7.38 (br, 1H), 7.19 (br, 1H), 6.48 (br, 1H), 4.02 (s, 3H), 3.98 (s, 3H).
HRMS (MALDI-TOF+) calculated for $C_{42}H_{23}Cl_7N_3O_2$ $[M]^+$: 845.9610, found 845.9585.

Synthesis of 2,6-bis(5-(bis(2,4,6-trichlorophenyl)methyl)-6-chloropyridin-2-yl)anthracene

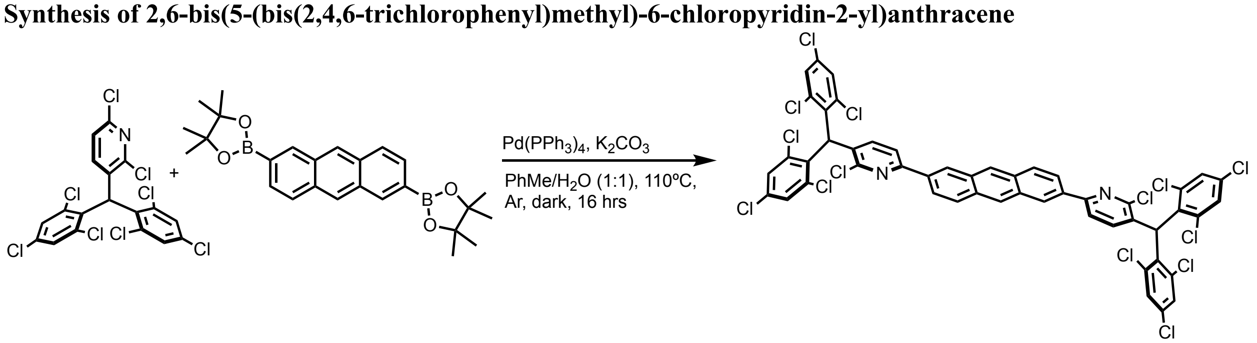

A 50 mL Schlenk flask was charged with 2,6-bis(4,4,5,5-tetramethyl-1,3,2-dioxaborolan-2-yl)anthracene (0.30 g, 0.70 mmol, 1 eq.), (2,6-dichloro-3-pyridyl)bis(2,4,6-trichlorophenyl)methane (0.91 g, 1.74 mmol, 2.5 eq.), K_2CO_3 (1.20 g, 8.68 mmol, 12.5 eq.), 8.6 mL of toluene, 8.6 mL of water, and a magnetic stir bar. The mixture was then degassed by bubbling argon through the suspension for 5 minutes, and then $Pd(PPh_3)_4$ (241 mg, 0.21 mmol, 0.3 eq.) was added. The flask was sealed and further degassed via three freeze, pump, thaw cycles. After the final thaw, the flask was protected from light with aluminum foil and heated to 110 ºC with stirring for 16 hours in an oil bath. Once the reaction was complete, the flask was removed from the oil bath and allowed to cool to room temperature. Then 40 mL of saturated aqueous NH_4Cl was added and the organic layer was extracted with DCM (3 x 30 mL). The organic layers were collected, dried with Na_2SO_4, and the solvent was removed via rotary evaporation. The residue was triturated with hexanes and the solid was filtered and dried to give 1.2 g of a pale-yellow powder that was used without further purification.

^{1}H NMR (400 MHz, $CDCl_3$) δ 8.72 (s, 2H), 8.54 (s, 2H), 8.13 (q, *J* = 9.0 Hz, 4H), 7.78 (d, *J* = 8.1 Hz, 2H), 7.38 (s, 8H), 7.30 (d, *J* = 8.0 Hz, 2H), 6.68 (s, 2H).
^{13}C{^{1}H} NMR (101 MHz, $CDCl_3$) δ 156.4, 151.5, 140.3, 137.5, 134.5, 134.2, 133.4, 132.3, 132.3, 131.4, 129.7, 129.2, 127.7, 127.3, 124.1, 119.3, 49.2.
HRMS (MALDI-TOF+) calculated for $C_{50}H_{23}Cl_{14}N_2$ $[M+H]^+$: 1140.7501, found 1140.7482.

Synthesis of 2,6-bis(5-(bis(2,4,6-trichlorophenyl)methyl)-6-chloropyridin-2-yl)-9,10-dihydro-9,10-[4,5]epidioxoloanthracen-13-one

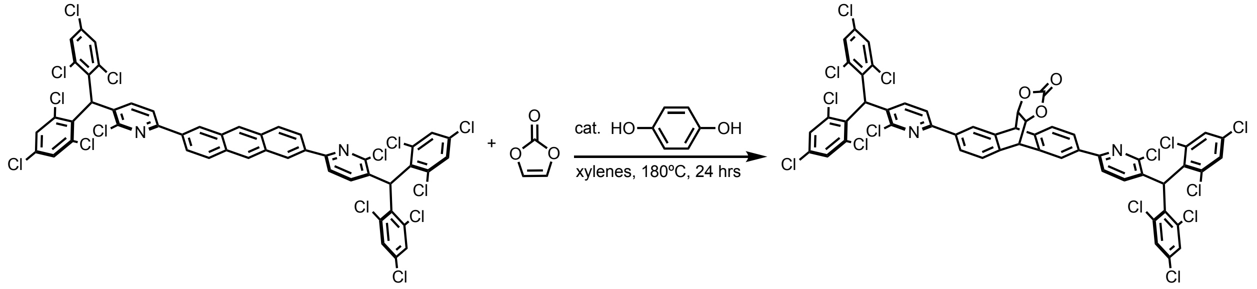

A 15 mL flame-dried pressure vessel was charged with 2,6-bis(5-(bis(2,4,6-trichlorophenyl)methyl)-6-chloropyridin-2-yl)anthracene (0.50 g, 0.44 mmol, 1 eq.), vinylene carbonate (1.35 g, 15.7 mmol, 36 eq.), trace hydroquinone (spatula tip), 5 mL of xylenes, and a magnetic stir bar. The vessel was sealed with a rubber stopper and argon was bubble through the suspension with stirring for 10 minutes. Then the rubber stopper was swapped for the cap to the vessel, and the vessel was sealed. The vessel was wrapped with aluminum foil to protect the reaction from light, and it was heated to 180 ºC with stirring for 16 hours. After the reaction was complete, the vessel was allowed to cool to room temperature before the dark brown solution was transferred to a 50 mL round bottom flask. The volatile compounds were removed via short path vacuum distillation. *Technical note:* vacuum should be introduced slowly to avoid bumping, ensuring the boiling flask is of sufficient volume also helps to alleviate this issue. The product was

purified by automated flash column chromatography eluting with chloroform on silica gel affording the title compound as an off-white powder (340 mg, 95% over two steps) as a mixture of diastereomers.

^{1}H NMR (500 MHz, $CDCl_3$) δ 8.17 (d, *J* = 8.0 Hz, 2H), 7.84 (dd, *J* = 13.5, 7.9 Hz, 2H), 7.56 (dd, *J* = 15.1, 8.0 Hz, 2H), 7.48 (d, *J* = 7.7 Hz, 2H), 7.35 (s, 8H), 7.24 – 7.17 (m, 2H), 6.62 (s, 2H), 4.95 (s, 2H), 4.88 (s, 2H).
^{13}C{^{1}H} NMR (126 MHz, $CDCl_3$) δ 156.0, 155.8, 154.1, 151.5, 151.3, 140.4, 140.3, 139.1, 138.3, 137.9, 137.4, 137.3, 136.9, 136.9, 134.3, 134.2, 133.4, 133.3, 133.2, 133.2, 131.8, 131.5, 129.7, 127.2, 126.5, 126.4, 126.3, 125.6, 124.6, 119.1, 119.0, 76.2, 76.2, 49.1, 49.1, 47.9.
HRMS (MALDI-TOF+) calculated for $C_{53}H_{25}Cl_{14}N_2O_3$ $[M+H]^+$: 1226.7588, found 1226.7588.

Synthesis of 2,6-bis(5-(bis(2,4,6-trichlorophenyl)methyl)-6-chloropyridin-2-yl)-9,10-dihydro-9,10-ethanoanthracene-11,12-diol

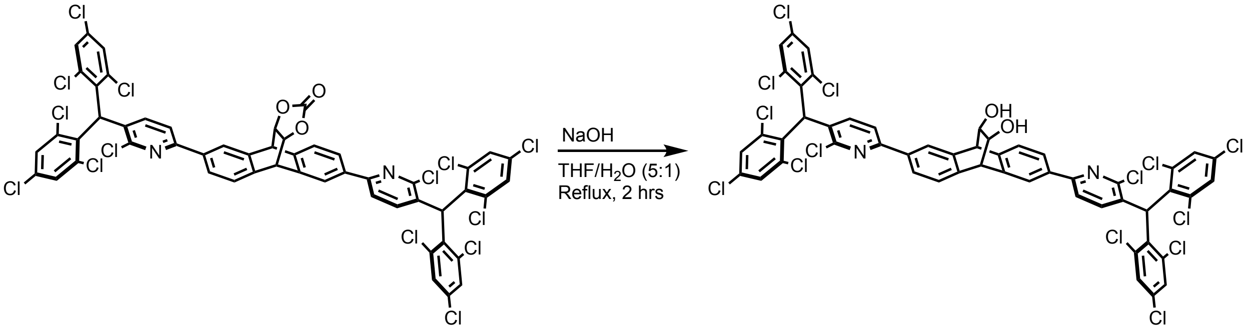

A 100 mL round bottom flask was charged with 2,6-bis(5-(bis(2,4,6-trichlorophenyl)methyl)-6-chloropyridin-2-yl)-9,10-dihydro-9,10-[4,5]epidioxoloanthracen-13-one (300 mg, 0.24 mmol), 30 mL tetrahydrofuran, 6 mL of 4 M aqueous NaOH, and a magnetic stir bar. The mixture was refluxed for 2 hours at 90 ºC with protection from light before cooling to room temperature and pouring into 30 mL H_2O. The water was then extracted with DCM (2 x 50 mL). The organic layers were combined and dried with Na_2SO_4. The solvent was decanted, and the solvent was removed via rotary evaporation to give the compound as an off-white solid (250 mg, 85%) as a mixture of diastereomers which was used without further purification.
^{1}H NMR (500 MHz, $CDCl_3$) δ 8.10 (d, *J* = 27.7 Hz, 2H), 7.80 (dd, *J* = 35.2, 7.7 Hz, 2H), 7.55 (dd, *J* = 14.9, 8.1 Hz, 2H), 7.44 (dd, *J* = 32.1, 7.6 Hz, 2H), 7.35 (s, 8H), 7.21 (d, *J* = 8.0 Hz, 2H), 6.61 (s, 2H), 4.58 (d, *J* = 5.6 Hz, 2H), 4.13 (d, *J* = 10.1 Hz, 2H), 2.38 – 2.16 (br, 2H).
^{13}C{^{1}H} NMR (126 MHz, $CDCl_3$) δ 156.6, 156.5, 151.3, 151.3, 141.6, 140.5, 140.5, 140.3, 140.3, 139.1, 137.4, 136.2, 136.1, 134.2, 133.4, 131.2, 131.1, 129.7, 127.1, 125.6, 125.6, 125.4, 125.3, 123.7, 119.0, 119.0, 68.2, 68.2, 51.5, 49.1.
HRMS (MALDI-TOF+) calculated for $C_{52}H_{27}Cl_{14}N_2O_2$ $[M+H]^+$: 1200.7712, found 1200.7814.

Synthesis of (9R,10R)-2,6-bis(5-(bis(2,4,6-trichlorophenyl)methyl)-6-chloropyridin-2-yl)-9,10-dihydro-9,10-ethanoanthracene-11,12-dione

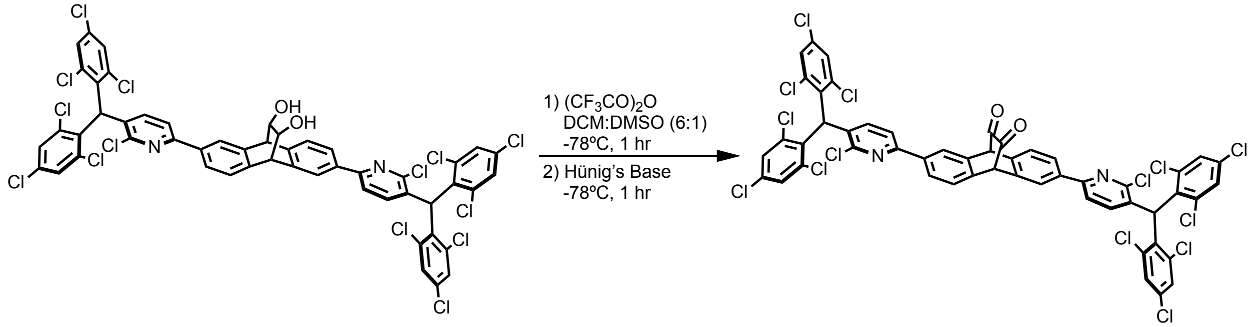

A flamed dried 100 mL round bottom flask was charged with 18 mL anhydrous DCM, 3 mL anhydrous DMSO, and a magnetic stir bar. The flask was sealed with a rubber stopper, cooled to -78 ºC (dry ice, isopropanol), and the contents were degassed with bubbling argon for 10 minutes. Afterwards, the argon line was removed, and the flask was protected from light with aluminum foil. Then, 3 mL of trifluoroacetic anhydride was added via syringe, and the reaction was stirred at -78 ºC for 10 minutes. 2,6-bis(5-(bis(2,4,6-trichlorophenyl)methyl)-6-chloropyridin-2-yl)-9,10-

dihydro-9,10-ethanoanthracene-11,12-diol (300mg, 0.25 mmol) was dissolved in 3 mL of anhydrous DCM and 3 mL of anhydrous DMSO and was added dropwise to the reaction vessel via syringe. The reaction was allowed to stir at -78 ºC for 1 hour. Then 3 mL of N,N-diisopropylethylamine was added dropwise and the reaction was stirred at -78 ºC for an additional hour before being allowed to warm up to room temperature. Once at room temperature, the light protection was removed and 10 mL of 1 M aqueous HCl was added. The contents of the flask were transferred to a separatory funnel. The aqueous layer was extracted with 50 mL of DCM, and the organic layer was separated. The aqueous layer was back extracted with an additional 25 mL of DCM, and the organic layers were combined and washed further with water (100 mL) and brine (100 mL) to remove DMSO. The organic layer was dried with Na_2SO_4, decanted, and volatiles were removed via rotary evaporation to give the title compound (285 mg, 96%) as an orange solid which was used without further purification.

^{1}H NMR (500 MHz, $CDCl_3$) δ 8.24 (s, 2H), 7.98 (d, *J* = 8.0 Hz, 2H), 7.57 (dd, *J* = 7.9, 3.3 Hz, 4H), 7.35 (s, 8H), 7.25 (d, *J* = 3.3 Hz, 2H), 6.62 (s, 2H), 5.16 (s, 2H).
^{13}C{^{1}H} NMR (126 MHz, $CDCl_3$) δ 183.3, 155.3, 151.6, 140.5, 138.8, 137.4, 136.2, 135.6, 134.3, 133.2, 132.2, 129.7, 128.1, 126.9, 125.2, 119.1, 60.1, 49.1.
HRMS: A suitable HRMS was not obtained for this compound. The predominate species observed was 2,6-bis(5-(bis(2,4,6-trichlorophenyl)methyl)-6-chloropyridin-2-yl)anthracene, the product of a formal retro Diels-Alder with extrusion of CO. This reaction type has been well established for similar compounds in the literature.[5]

Synthesis of 2H

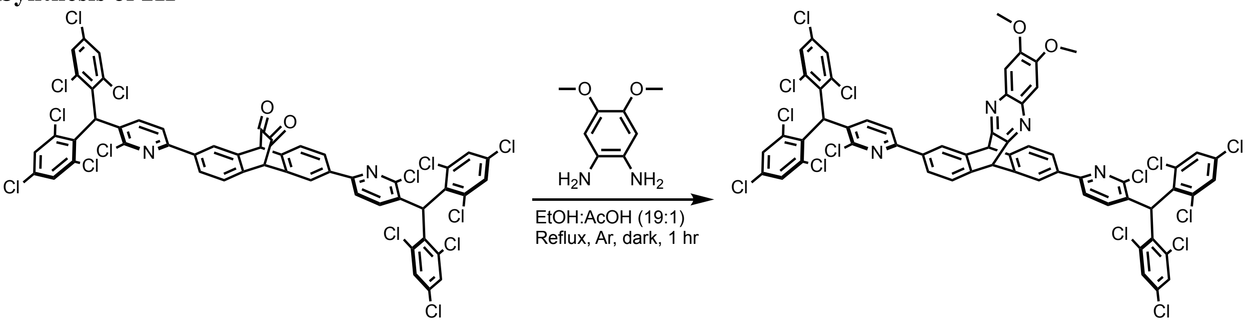

A flame dried 25 mL round bottom flask was charged with (9R,10R)-2,6-bis(5-(bis(2,4,6-trichlorophenyl)methyl)-6-chloropyridin-2-yl)-9,10-dihydro-9,10-ethanoanthracene-11,12-dione (153 mg, 0.28 mmol, 1 eq.), 1,2-diamino-4,5-dimethoxybenzene (23.5 mg, 0 mmol, 1.1 eq.), 15 mL of a 19:1 mixture of absolute ethanol and acetic acid, and a magnetic stir bar. This suspension was degassed with bubbling argon for 5 minutes before a reflux condenser was attached and the apparatus was protected from light with aluminum foil. The vessel was then refluxed (100 ºC) under argon for 1 hour before cooling to room temperature. The precipitate was filtered, washed with methanol, and dried *in vacuo* to give **2H** (93 mg, 54%) as a yellow powder. *Technical note:* Occasionally it was found that this reaction does not yield completely pure product, mostly dependent on how finely divided the starting material is. In these instances, it was found that pure compound could be obtained boiling impure powder in acetone and cooling to -10ºC and then collecting the solid. As mentioned in the manuscript, attempts at column chromatography on silica or neutral alumina caused a retro Diels-Alder reaction to occur and only the 2,6-bis(5-(bis(2,4,6-trichlorophenyl)methyl)-6-chloropyridin-2-yl)anthracene was isolated in those cases. However, carrying impure material forward was not found to have a substantial effect on the radical formation reaction.

^{1}H NMR (500 MHz, $CDCl_3$) δ 8.27 (s, 2H), 7.73 (d, *J* = 7.8 Hz, 2H), 7.60 (d, *J* = 7.8 Hz, 2H), 7.51 (d, *J* = 8.1 Hz, 2H), 7.33 (s, 8H), 7.25 (s, 2H), 7.19 (d, *J* = 8.1 Hz, 2H), 6.60 (s, 2H), 5.74 (s, 2H), 3.99 (s, 6H).
^{13}C{^{1}H} NMR (126 MHz, $CDCl_3$) δ 156.1, 155.0, 152.1, 151.3, 144.1, 143.2, 140.2, 137.39, 135.9, 135.9, 134.1, 134.1, 133.3, 131.3, 129.6, 125.4, 125.4, 123.7, 119.0, 107.0, 56.4, 55.3, 49.1, 31.1.
HRMS (MALDI-TOF+) calculated for $C_{60}H_{31}Cl_{14}N_4O_2$ $[M+H]^+$: 1328.8081, found 1326.8141.

---

[5] Jia, J.; Yamaguchi, Y.; Ueda, T.; Yamada, H.; Kakiuchi, K.; Morimoto, T. Rhodium (I)-Catalyzed [2+ 2+ 1]-Carbonylative Cycloaddition of Diynes with Anthracene α-Diketone as the Source of CO. *Synlett* **2022**, *33* (19), 1948-1952.

Synthesis of 2

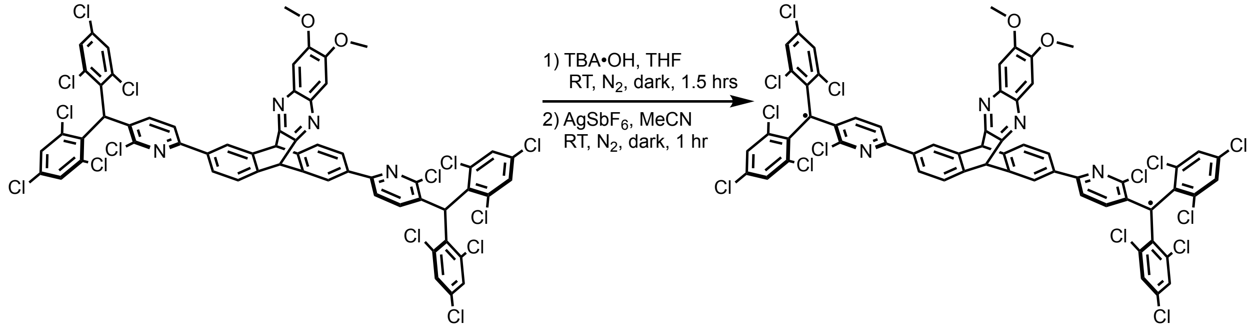

Inside of a nitrogen filled glovebox, a dry 50 mL flask was charged with **2H** (50.0 mg, 0.04 mmol, 1 eq.), 20 mL anhydrous THF, and a magnetic stir bar. The vessel was protected from light and tetrabutyl ammonium hydroxide (1 M in MeOH, 0.24 mL, 0.24 mmol, 6.4 eq.) was added dropwise. The reaction was then allowed to stir for 1.5 hours at room temperature. Silver hexafluoroantimonate (121 mg, 0.35 mmol, 9.5 eq.) was dissolved in 25 mL anhydrous MeCN, and this solution was added dropwise to the now deep purple THF solution. The reaction was stirred for an additional hour at room temperature before being removed from the glovebox. The solution was filtered through Celite® (diatomaceous earth) before the solvent was removed via rotary evaporation. Purification by flash chromatography (2 times) on neutral alumina (Brockmann grade I) eluting with 1:1 hexane:chloroform (R_f = 0.2) afforded the **2** as a dark yellow powder (19.9 mg, 26%).

^{1}H NMR (500 MHz, CD_2Cl_2, 25ºC) δ 7.41 (br, 2H), 5.55 – 5.52 (br, 2H), 4.09 (s, 6H).

HRMS (MALDI-TOF+) calculated for $C_{60}H_{29}Cl_{14}N_4O_2$ $[M+H]^+$: 1326.7924, found 1326.7983.

Synthesis of 3H

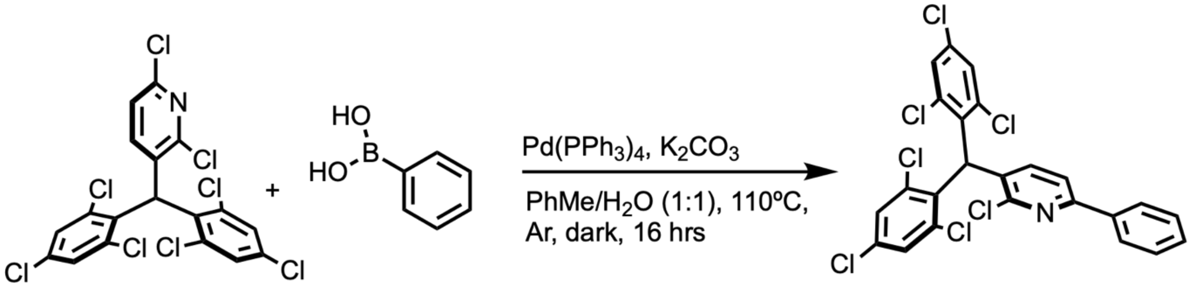

A 50 mL Schlenk flask was charged with phenylboronic acid (81.4 mg, 0.67 mmol, 1 eq.), (2,6-dichloro-3-pyridyl)bis(2,4,6-trichlorophenyl)methane (400 mg, 0.77 mmol, 1.15 eq.), K_2CO_3 (636 mg, 4.6 mmol, 6 eq.), 4.6 mL of toluene, 4.6 mL of water, and a magnetic stir bar. The mixture was then degassed by bubbling argon through the suspension for 5 minutes, and then $Pd(PPh_3)_4$ (88 mg, 0.077 mmol, 0.12 eq.) was added. The flask was heated to 110 ºC with stirring for 16 hours in an oil bath. Once the reaction was complete, the flask was removed from the oil bath and allowed to cool to room temperature. Then 20 mL of saturated aqueous NH_4Cl was added and the organic layer was extracted with DCM (3 x 20 mL). The organic layers were collected, dried with Na_2SO_4, and the solvent was removed via rotary evaporation. The product was purified by automated flash column chromatography eluting with 2:1 hexane:DCM on silica gel affording compound **3H** as a white powder (365 mg, 97%).

^{1}H NMR (500 MHz, $CDCl_3$) δ 8.03 (d, J = 6.9 Hz, 2H), 7.59 (d, J = 8.0 Hz, 1H), 7.48 – 7.43 (m, 3H), 7.36 (s, 4H), 7.23 (d, J = 8.0 Hz, 1H), 6.63 (s, 1H).

^{13}C NMR (126 MHz, $CDCl_3$) δ 156.7, 151.3, 140.2, 137.4, 137.3, 134.1, 134.0, 131.2, 129.9, 129.7, 129.0, 127.0, 118.9, 49.1.

HRMS (AccuTOF-DART+) calculated for $C_{24}H_{13}Cl_7N$ $[M+H]^+$: 559.8862, found 559.8870.

Synthesis of 3

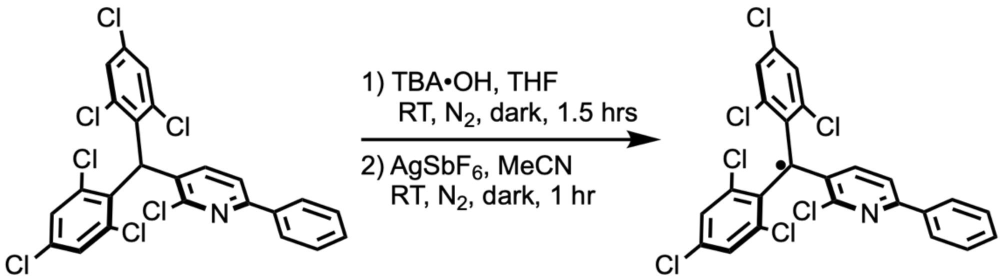

Inside of a nitrogen filled glovebox, a dry 50 mL flask was charged with compound **3H** (100 mg, 0.18 mmol, 1 eq.), 20 mL anhydrous THF, and a magnetic stir bar. The vessel was protected from light and tetrabutylammonium hydroxide (1 M in MeOH, 0.56 mL, 0.56 mmol, 3.2 eq.) was added dropwise. The reaction was then allowed to stir for 1.5 hours at room temperature. Silver hexafluoroantimonate (290 mg, 0.84 mmol, 4.75 eq.) was dissolved in 25 mL anhydrous MeCN, and this solution was added dropwise to the now deep purple THF solution. The reaction was stirred for an additional hour at room temperature before being removed from the glovebox. The solution was filtered through Celite® (diatomaceous earth) before the solvent was removed via rotary evaporation. Purification by flash chromatography on neutral alumina (Brockmann grade II) eluting with 2:1 hexane:DCM ($R_f = 0.6$) afforded **3** as a red powder (71 mg, 71%).

^{1}H NMR (500 MHz, CD_2Cl_2, 25ºC) Species is NMR silent, no resonances were observed.

HRMS (AccuTOF-DART+) calculated for $C_{24}H_{11}Cl_7N$ $[M]^+$: 557.8706, found 557.8741.

MALDI-TOF

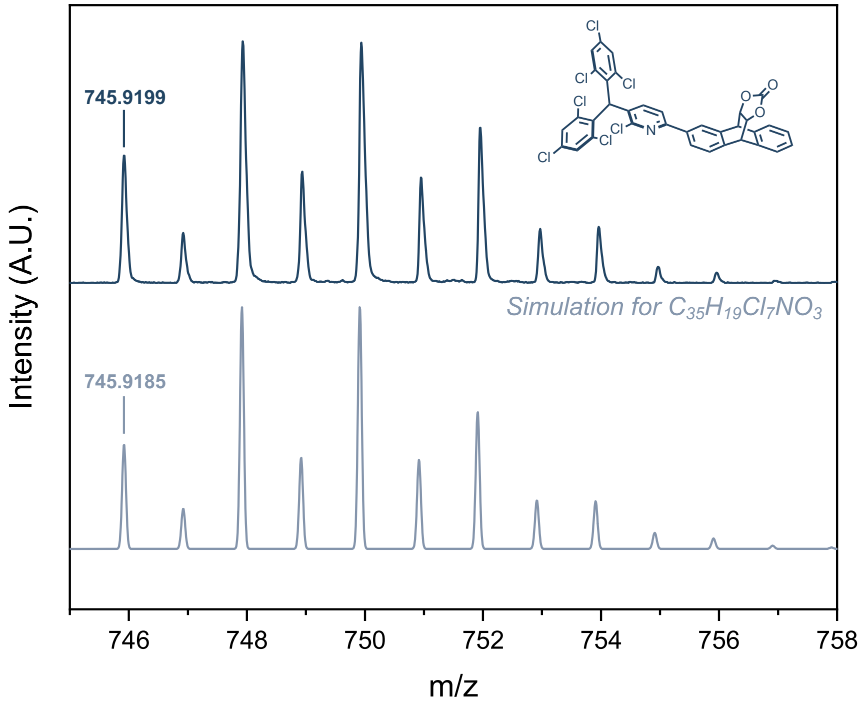

Figure S1: Experimental and simulated MALDI-TOF-MS of 2-(5-(bis(2,4,6-trichlorophenyl)methyl)-6-chloropyridin-2-yl)-9,10-dihydro-9,10-[4,5]epidioxoloanthracen-13-one.

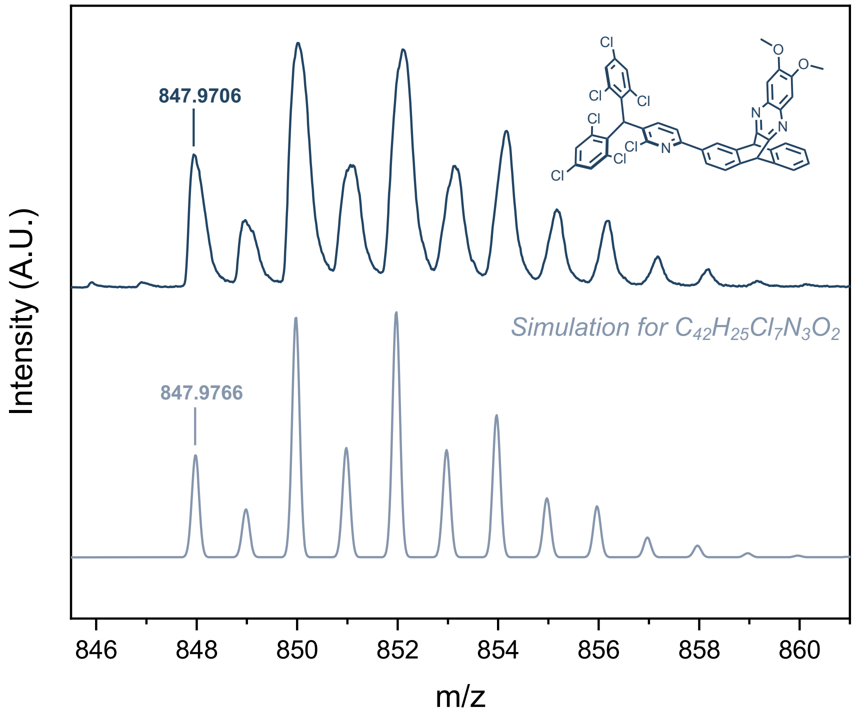

Figure S2: Experimental and simulated MALDI-TOF-MS of compound **1H**.

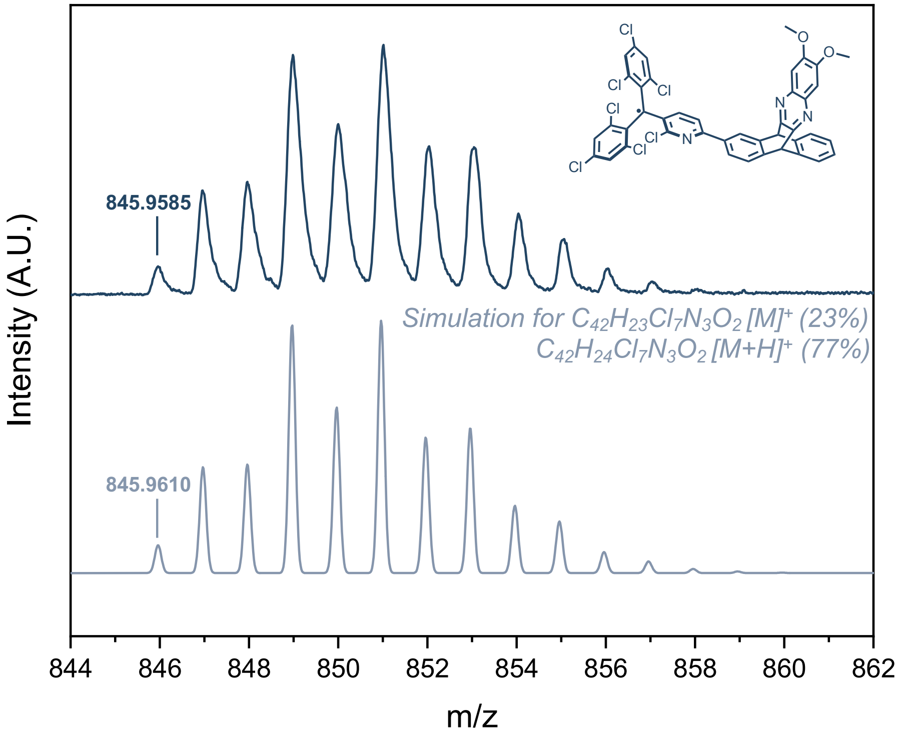

Figure S3: Experimental and simulated MALDI-TOF-MS of compound **1**. The pattern is best simulated as a mixture of the M^{+} and the $M^{+}H$ ions.

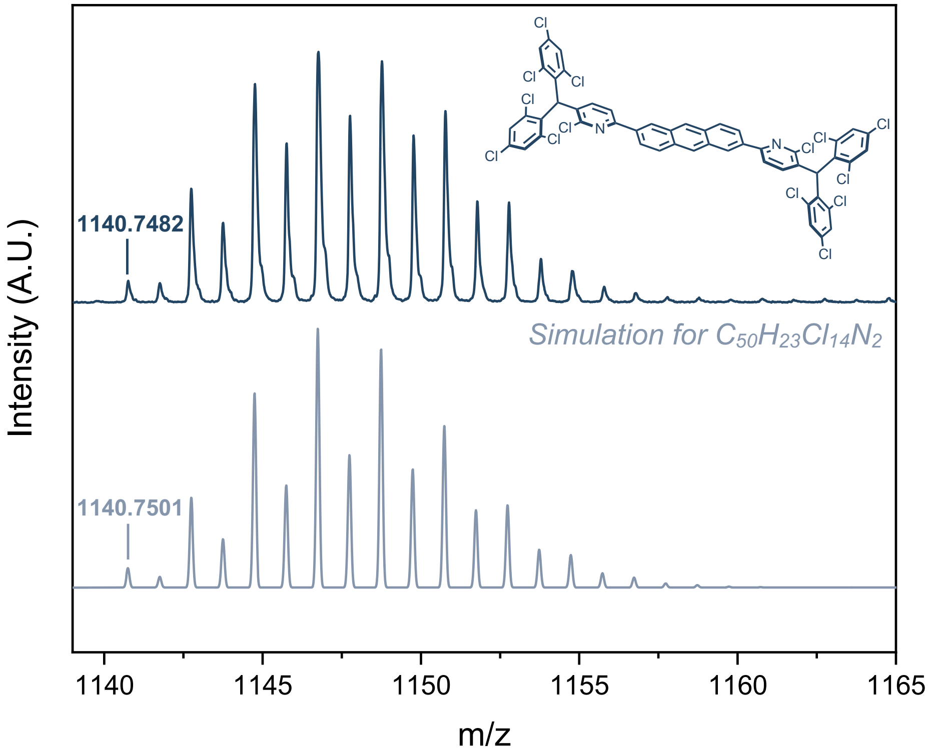

Figure S4: Experimental and simulated MALDI-TOF-MS of 2,6-bis(5-(bis(2,4,6-trichlorophenyl)methyl)-6-chloropyridin-2-yl)anthracene.

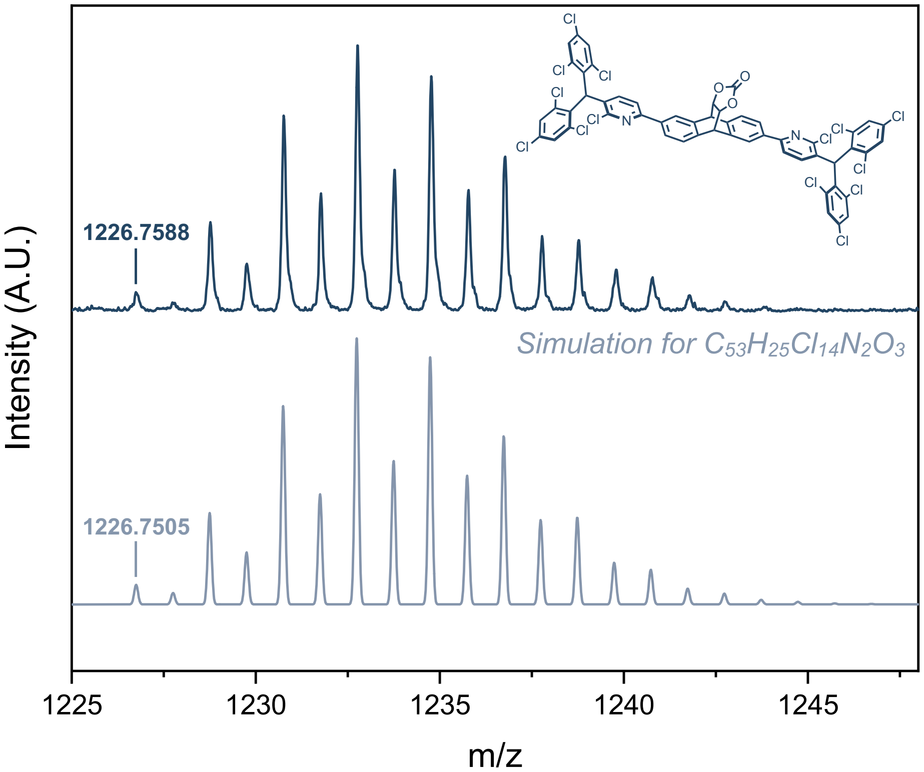

Figure S5: Experimental and simulated MALDI-TOF-MS of 2,6-bis(5-(bis(2,4,6-trichlorophenyl)methyl)-6-chloropyridin-2-yl)-9,10-dihydro-9,10-[4,5]epidioxoloanthracen-13-one.

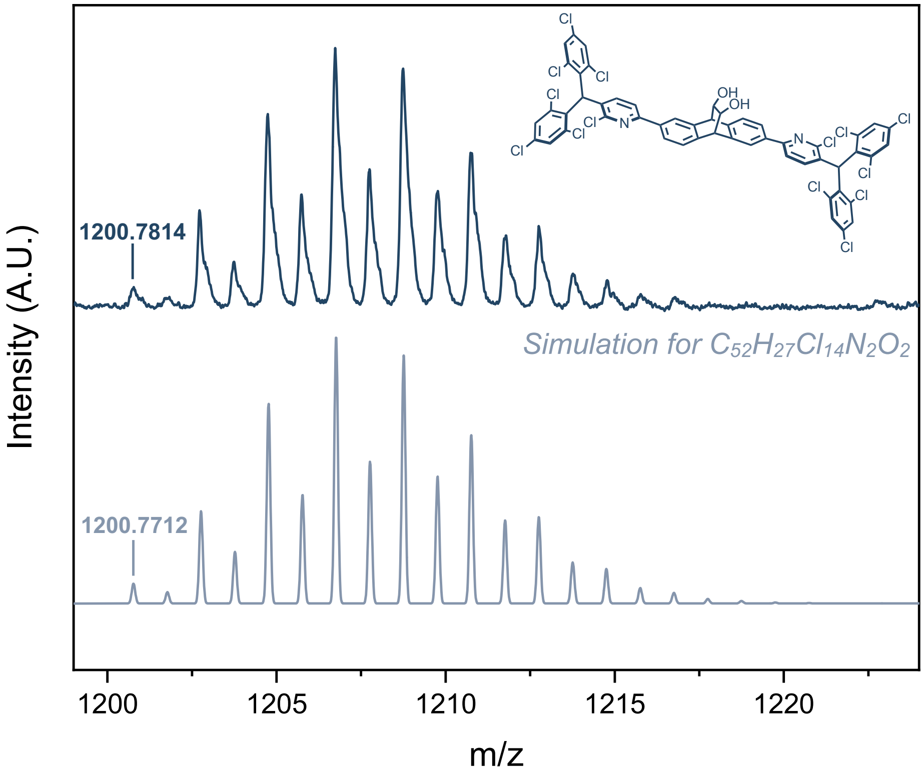

Figure S6: Experimental and simulated MALDI-TOF-MS of compound 2,6-bis(5-(bis(2,4,6-trichlorophenyl)methyl)-6-chloropyridin-2-yl)-9,10-dihydro-9,10-ethanoanthracene-11,12-diol.

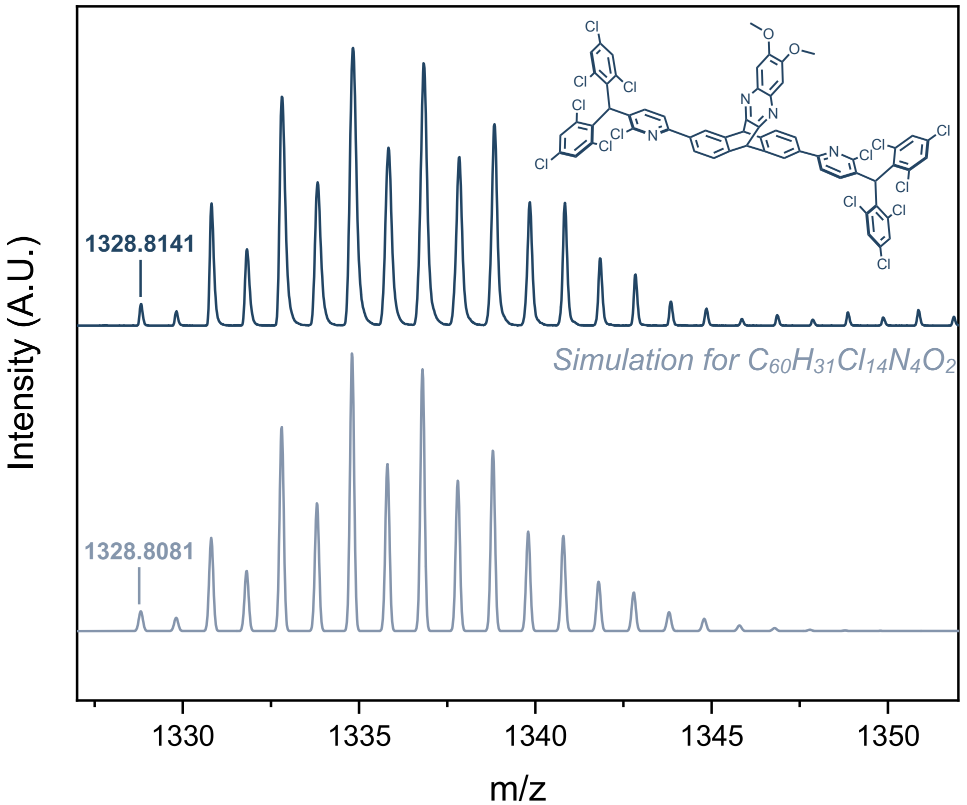

Figure S7: Experimental and simulated MALDI-TOF-MS of compound **2H**.

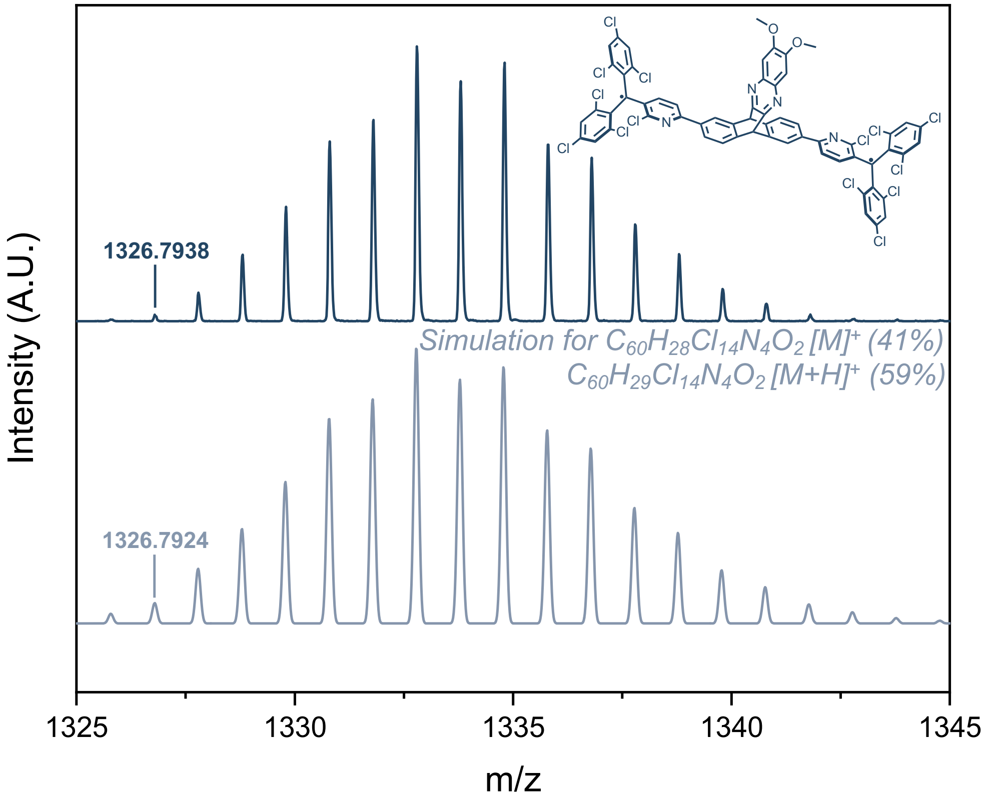

Figure S8: Experimental and simulated MALDI-TOF-MS of compound **2**. The pattern is best simulated as a mixture of the $[M]^+$ and the $[M+H]^+$ ions.

Thermal Gravimetric Analysis

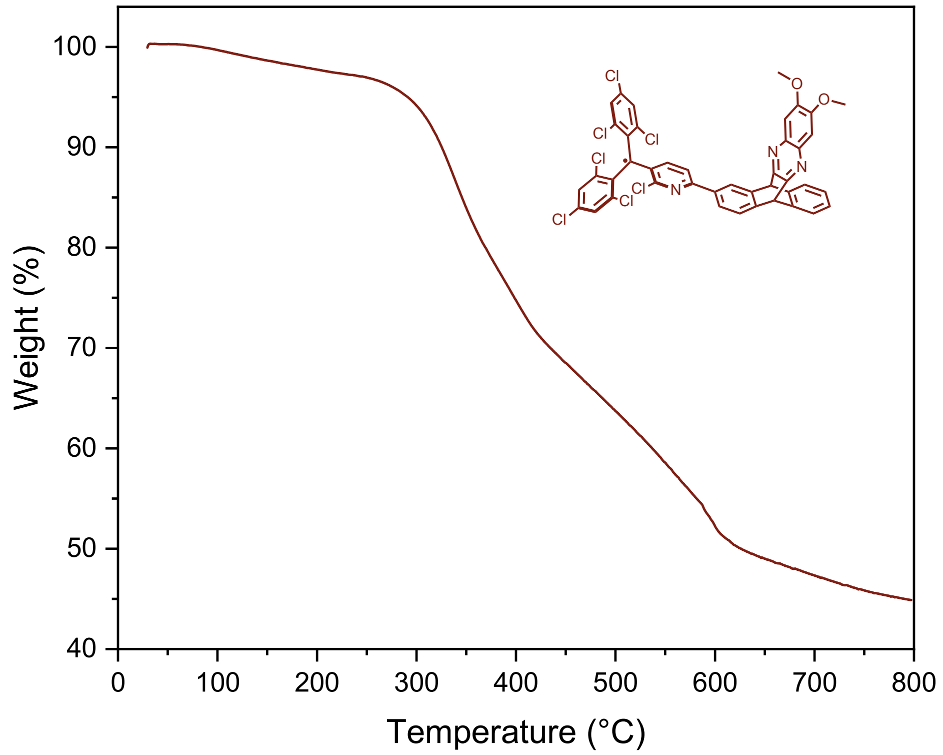

Figure S9: TGA curve of compound **1**. $T_{d,5}$ = 291 ºC

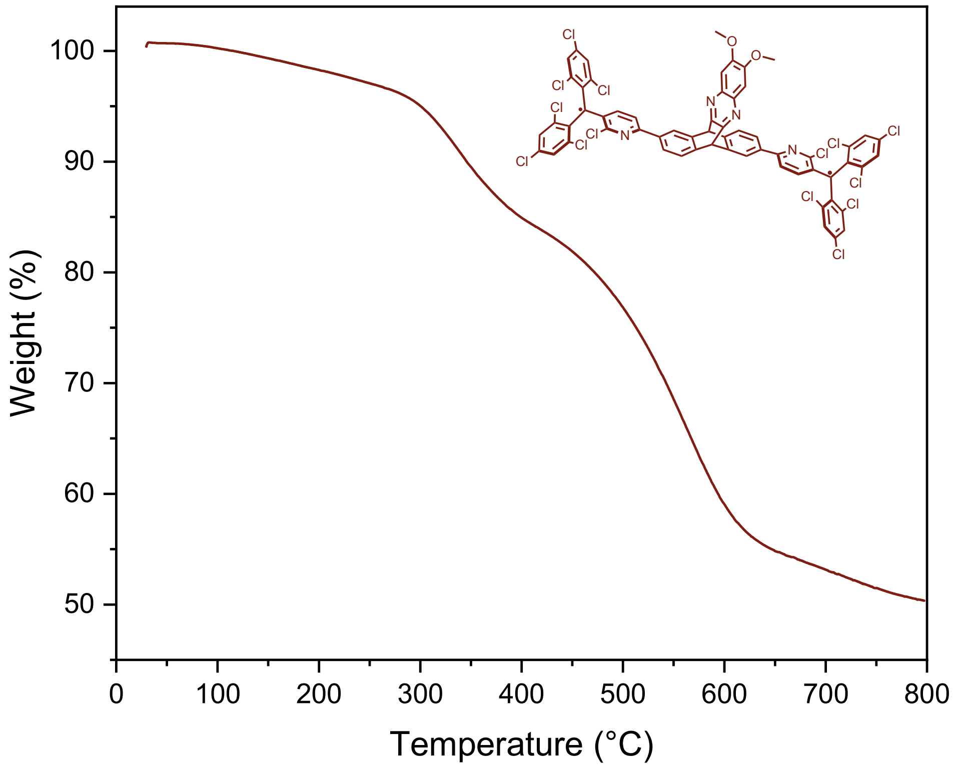

Figure S10: TGA curve of compound **2**. $T_{d,5}$ = 300 ºC

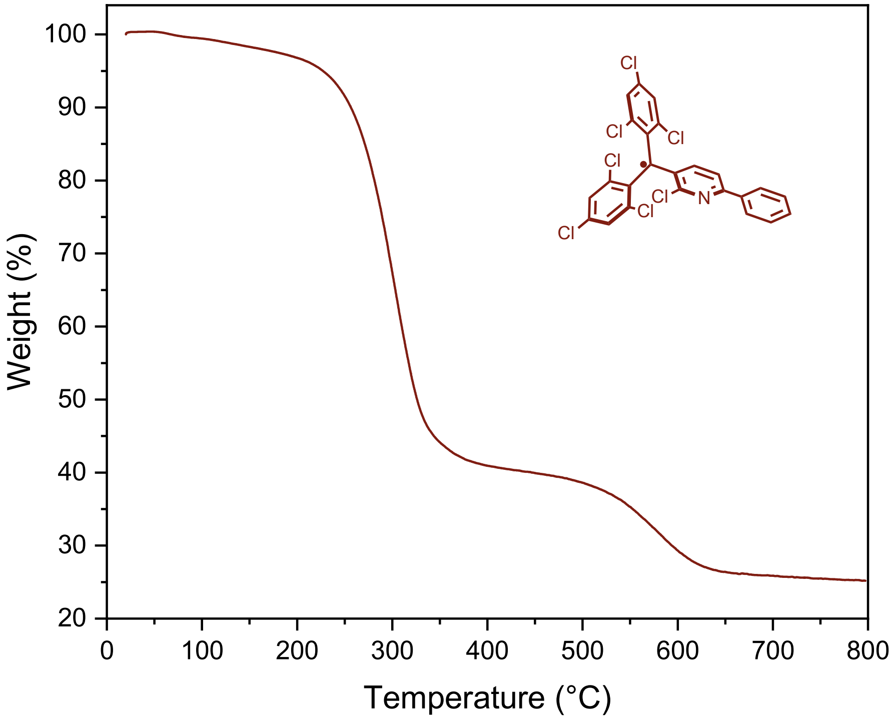

Figure S11: TGA curve of compound **3**. $T_{d,5}$ = 227 ºC

Cyclic Voltammetry

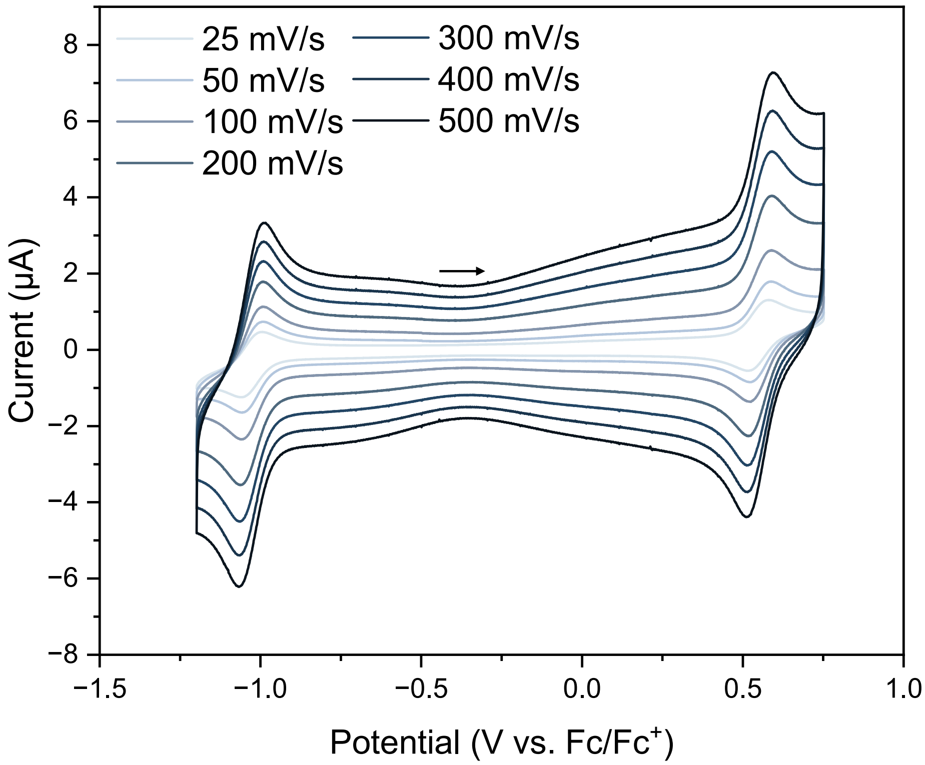

Figure S12: Cyclic voltammogram of compound **1** (0.1 mM) in DCM. Supporting electrolyte TBA•PF_6 (0.1 M), glassy carbon working electrode, platinum mesh counter electrode, Ag/Ag^+ (3 M aq. KCl) reference electrode.

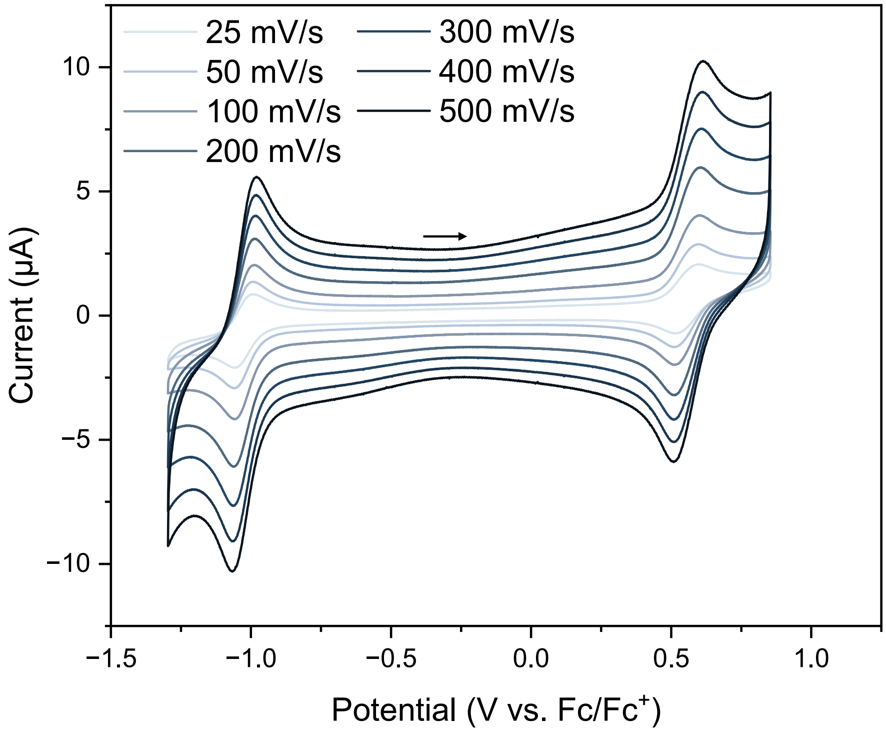

Figure S13: Cyclic voltammogram of compound **2** (0.1 mM) in DCM. Supporting electrolyte TBA•PF_6 (0.1 M), glassy carbon working electrode, platinum mesh counter electrode, Ag/Ag^+ (3 M aq. KCl) reference electrode.

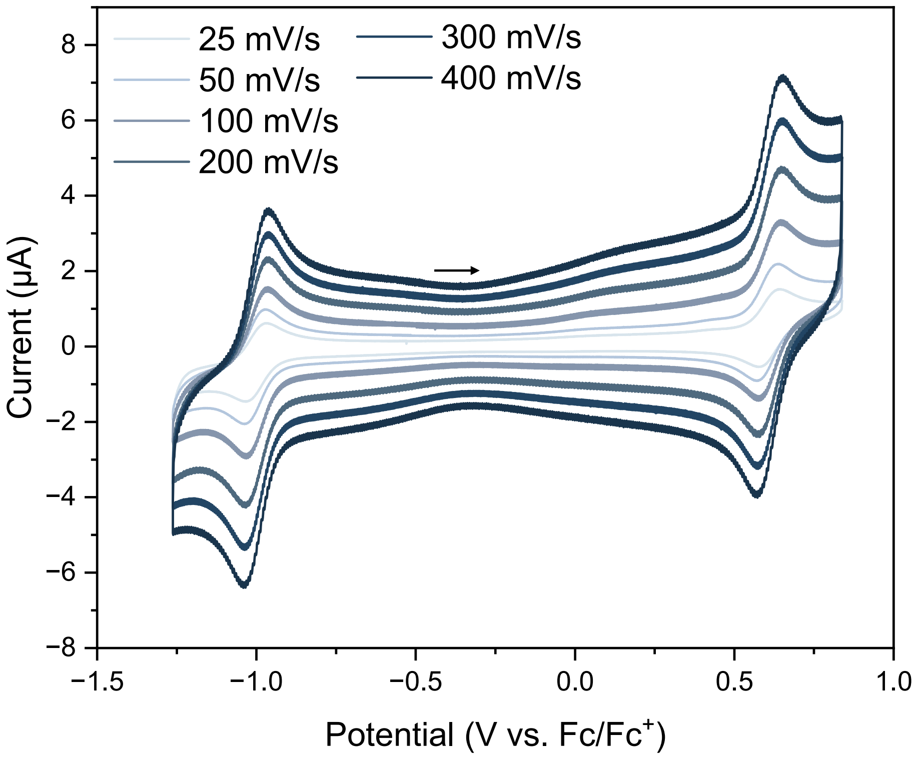

Figure S14: Cyclic voltammogram of compound **3** (0.1 mM) in DCM. Supporting electrolyte TBA•PF_6 (0.1 M), glassy carbon working electrode, platinum mesh counter electrode, Ag/Ag^+ (3 M aq. KCl) reference electrode.

Table S1: Summary of cyclic voltammetry data for compounds **1**, **2**, and **3**. Peak to peak to peak separation is calculated using a scan rate of 100 mV/s.

Compound	Redox Potential	I_{PA}-I_{PC}	Assignment
**1**	E^{ox1} = 0.56 V	65.4 mV	1 e^-
**1**	E^{red1} = -1.03 V	69.5 mV	1 e^-
**2**	E^{ox2} = 0.56 V	71.6 mV	2 e^-
**2**	E^{red2} = -1.02 V	91.9 mV	2 e^-
**3**	E^{ox3} = 0.61V	74.7 mV	1 e^-
**3**	E^{red3} = -1.00V	61.5 mV	1 e^-

SQUID MAGNETOMETRY

The procedure for analyzing SQUID data closely follows that reported by Rajca.[9] For compounds **1** and **2**, initial SQUID runs involved samples of 4.0 mg and 4.9 mg, respectively. For the background SQUID runs a major portion of the sample was removed from the holder, to leave 2.0 mg and 1.0 mg of compound **1** and **2**, respectively. Following these runs, magnetization for the background, M_{bck}, was point-by-point subtracted from magnetization for the sample, M_{sample}, to provide corrected magnetization, M_{cr}, accounting for diamagnetism of sample holder. Thus, M_{cr} was 2.0 mg and 3.9 mg for compounds **1** and **2,** respectively. Subsequently, the M_{cr} were converted to corresponding molar magnetic susceptibilities for compounds **1** and **2**:

$$\chi_{cor} = \frac{M_{cor}}{H * n_{molsample}}$$

where H is the applied magnetic field in Oe and $n_{molsample}$ is the number of moles of the sample for **1** and **2**. Final molar magnetic susceptibility, χ, was obtained by subtraction of Pascal constant derived correction[6] P:

$$\chi = \chi_{cor} - P$$

where P = -0.0004824 emu/mol and P = -0.0007502 emu/mol for compounds **1** and **2** respectively. Consitutive corrections were applied for compound **3** which gave P = -0.0001623 emu/mol. This procedure only gave satisfactory χ for the monoradicals **1** and **3**. For biradical **2**, χ_{cor} was used directly.

Selected models for numerical fitting of magnetic data

All numerical fitting was done in Origin software using a nonlinear curve fit following the Levenberg Marquardt iteration algorithm. Compound **2** was also fitted to S = 1 limiting models for dimers and 1-D chains as described by Rajca,[7] however, the fits were generally worse. This suggests that compound **2** behaves more like independent S=1/2 than S=1, albeit with an overall higher density of spins than compound **1**. Best fits in tables S1 and S2 were identified as those having the highest adjusted R^2 value and the most sensical parameters (i.e. N closest to 1, indicating a relatively pure compound).

One-dimensional antiferromagnetic S = ½ chain model.[8]

This model is used as described by Rajca[9] and employs the Heisenberg Hamiltonian model without the usual factor of 2 for compound **1** or **3** and with the usual factor of 2 for compound **2**. It is used to fit χT *vs* T and χ *vs* T data, in order to obtain the intra-chain antiferromagnetic exchange coupling constant, J'/k, in Kelvin. Diamagnetic impurities and inaccuracies in the mass balance are accounted for by the mass factor, N; free S = ½ radicals, i.e., crystal defects, are accounted with parameter N_{imp}. There is also a possibility of adding mean-field parameter θ, to account for relatively weak inter-molecular interactions (compared to intra-dimer J'/k) between radical molecules; in this case N_{imp} is set to 0. This model accounts for paramagnetic saturation and was applied as a three parameter fit.

$$\chi T = \left\{2 * 1.506 * N * \left[\frac{T}{T-\theta}\right] * \left[\frac{Num}{Den}\right]\right\} + Monorad$$

[6] Kahn, O. *Molecular magnetism*, Wiley-VCH, New York, 1993, Table 1.1.

[7] Wang, W.; Chen, C.; Shu, C.; Rajca, S.; Wang, X.; Rajca, A. S = 1 Tetraazacyclophane Diradical Dication with Robust Stability: A Case of Low-Temperature One-Dimensional Antiferromagnetic Chain. *J. Am. Chem. Soc.* **2018**, *140* (25), 7820-7826.

[8] Kahn, O. *Molecular magnetism*, Wiley-VCH, New York, 1993, Ch. 11, p. 272, Eq. 11.1.5.

[9] Yang, Z.; Pink, M.; Nowik-Boltyk, E. M.; Lu, S.; Junghoefer, T.; Rajca, S.; Stoll, S.; Casu, M. B.; Rajca, A. Thermally Ultrarobust S = 1/2 Tetrazolinyl Radicals: Synthesis, Electronic Structure, Magnetism, and Nanoneedle Assemblies on Silicon Surface. *J. Am. Chem. Soc.* **2023**, *145* (24), 13335-13346.

$$Num = 0.25 + (0.074975 * x) + (0.75235 * x^2)$$

$$Den = 1 + (0.9931 * x) + (0.172135 * x^2) + (0.75825 * x^3)$$

$$x = -\frac{J'}{T}$$

$$Monorad = \frac{\left\{T * 1.118 * N_{imp} * \left\{\frac{\sinh(a)}{[2 + 2 * \cosh(a)]}\right\}\right\}}{H}$$

$$a = 1.345 * \left(\frac{H}{T - \theta}\right)$$

S = ½ dimer model (diradical)[10]

This model is employing Heisenberg Hamiltonian model without the usual factor of 2 for compound **1** or **3** and with the usual factor of 2 for compound **2**. It is used to fit χT *vs* T and χ *vs* T data, in order to obtain the singlet-triplet energy gap (2J/k in Kelvin) in the dimer (compound **2**) or in other words, intra-dimer exchange coupling constant, J'/k, in Kelvin (for compound **1**). Where needed, impurities and inaccuracies in the mass balance are accounted for by the mass factor, N; relatively weak inter-molecular interactions (compared to 2J/k) between diradical molecules are described by mean-field parameter θ. This model accounts for paramagnetic saturation. For compounds **1** and **2**, this formula is applied as a two-parameter fit with θ set to 0 or a three-parameter fit.

$$\chi T = \frac{2 * T * 1.118 * N * \frac{\sinh(a)}{1 + 2\cosh(a) + \exp\left(\frac{\frac{-2J}{k}}{T}\right)}}{H}$$

$$a = 1.345\ \left(\frac{H}{T - \theta}\right)$$

Table S2: Fitting parameters for compound **1**

χT or χ	Fit	J'/k	N	N_{imp}	θ	Adj. R^2
χT	1D-Chain	0.68 ± 0.08	1.015 ± 0.003	0	-1.83 ± 0.12	0.9444
	1D-Chain	-8.16 ± 0.76	0.290 ± 0.015	0.727 ± 0.015	0	0.9438
	Dimer	-0.71 ± 6.95	1.010 ± 0.002	NA	-1.44 ± 3.56	0.9357
	Dimer	-1.37 ± 0.04	1.000 ± 0.002	NA	0	0.9013
χ	1D-Chain	0.19 ± 0.02	0.993 ± 0.001	0	-1.01 ± 0.02	1.000
	1D-Chain	-0.41 ± 0.02	3.223 ± 0.141	-2.236 ± 0.142	0	1.000
	Dimer	0.55 ± 0.99	0.998 ± 8.634	NA	-1.38 ± 0.52	0.9999
	Dimer	-1.21 ± 0.01	0.960 ± 0.004	NA	0	0.9999

[10] Rajca, A. Organic diradicals and polyradicals: from spin coupling to magnetism?. *Chem. Rev.* **1994**, *94*, 871–893.

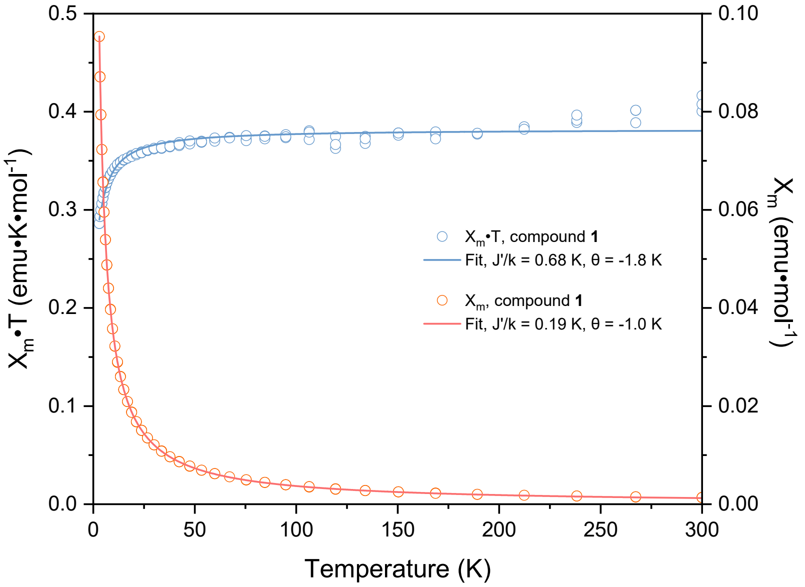

Figure S15: Fit data for compound **1**.

Table S3: Fitting parameters for compound **2**.

χT or χ	Fit	J/k	N	N_{imp}	θ	Adj. R^2
χT	1D-Chain	0.56 ± 0.05	0.997 ± 0.001	0	-1.88 ± 0.07	0.9872
	1D-Chain	-1.26 ± 0.92	1.404 ± 0.893	-0.829 ± 1.791	0	0.9807
	Diradical	0.79 ± 3.95	0.994 ± 0.002	NA	-1.70 ± 2.05	0.9841
	Diradical	-1.56 ± 0.03	0.981 ± 0.002	NA	0	0.9509
χ	1D-Chain	0.29 ± 0.01	0.982 ± 0.001	0	-1.46 ± 0.02	0.9999
	1D-Chain	-0.31 ± 0.01	5.200 ± 0.179	-8.457 ± 0.357	0	0.9999
	Diradical	0.64 ± 1.17	0.976 ± 0.001	NA	-1.47 ± 0.62	0.9999
	Diradical	-1.23 ± 0.01	0.934 ± 0.002	NA	0	0.9998

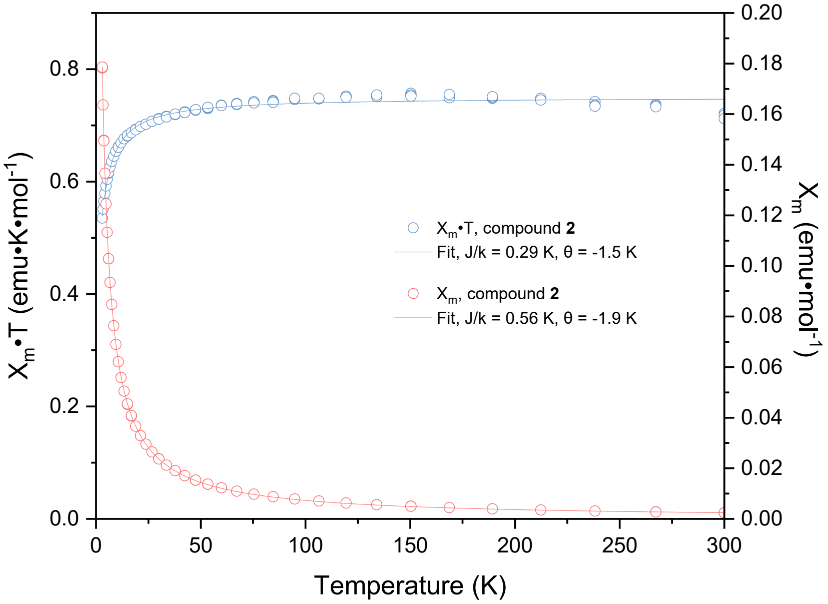

Figure S16: Fit data for compound **2**.

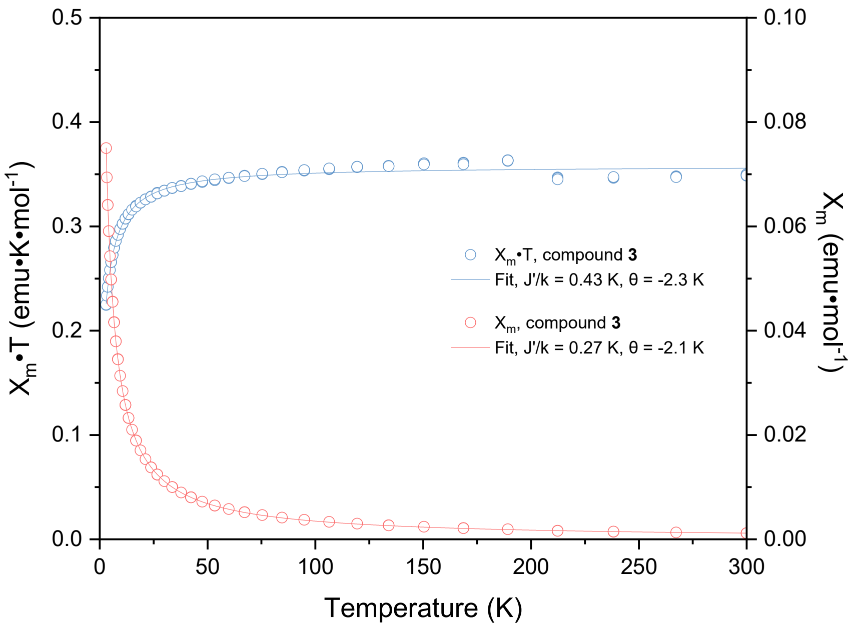

Figure S17: Fit data for compound **3**.

Table S4: Fitting parameters for compound **3**.

χT or χ	Fit	J'/k	N	N_{imp}	θ	Adj. R^2
χT	1D-Chain	0.43 ± 0.08	0.951 ± 0.002	0	-2.33 ± 0.10	0.9913
	1D-Chain	-1.19 ± 0.80	1.871 ± 1.127	-0.930 ± 1.130	0	0.9847
	Diradical	1.04 ± 2.56	0.951 ± 0.002	NA	-2.46 ± 1.36	0.9901
	Diradical	-2.02 ± 0.03	0.930 ± 0.003	NA	0	0.9537
χ	1D-Chain	0.27 ± 0.01	0.943 ± 0.001	0	-2.06 ± 0.01	1.000
	1D-Chain	-0.26 ± 0.01	8.116 ± 0.152	-7.196 ± 0.151	0	0.9999
	Diradical	0.85 ± 0.04	0.941 ± 0.001	NA	-2.25 ± 0.24	0.9999
	Diradical	-1.60 ± 0.02	0.867 ± 0.003	NA	0	0.9994

Electron Paramagnetic Resonance

Easyspin fitting parameters for X-band spectra (derivative of EDFS at 70 K, CW at 293K) for monoradical and diradical:

Diradical (**2**) (70 K)	g_{iso}	2.00522
	Linewidth peak-peak (mT)	0.481
	Electron-electron coupling (MHz)	[1 1 -2]*17.5
Monoradical (**1**) (70 K)	g_{iso}	2.00525
	Linewidth peak-peak (mT)	0.667
Diradical (**2**) (293 K)	g_{iso}	2.00325
	Linewidth peak-peak (mT)	0.6
Monoradical (**1**) (293 K)	g_{iso}	2.00305
	Linewidth peak-peak (mT)	0.667
Phenylradical (**3**) (293 K)	g_{iso}	2.00298
	Linewidth peak-peak (mT)	0.500625

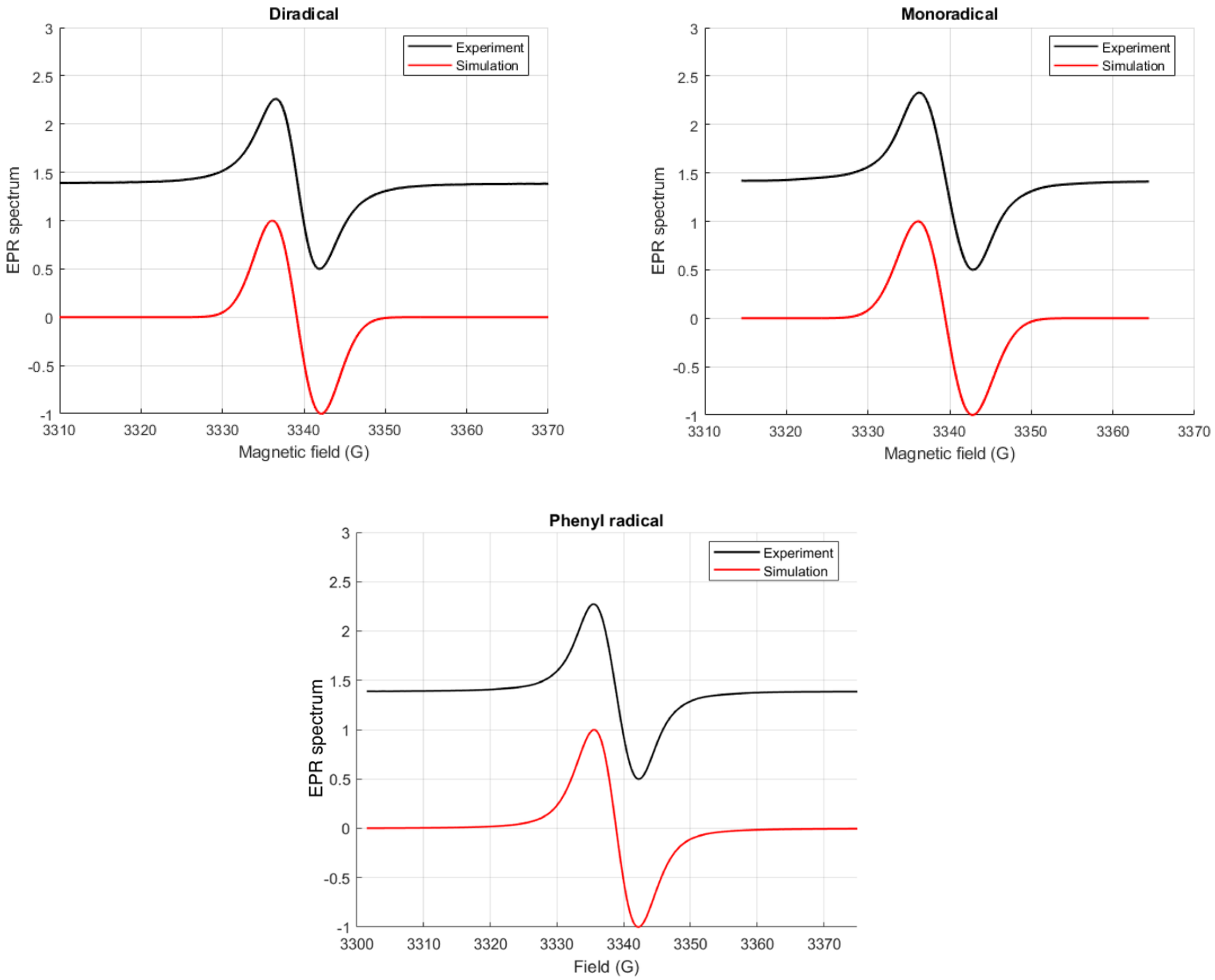

Figure S18: X-band continuous wave EPR spectra at 293 K for compound **1** (top left), **2** (top right), and **3** (bottom).

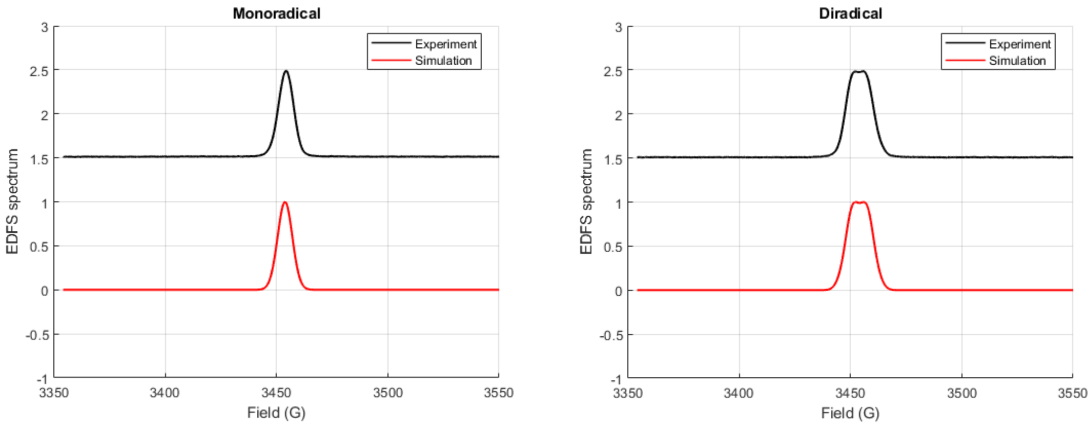

Figure S19: X-band EPR spectra of the gradient of the echo-detected field sweep (EDFS) at 70 K for compound **1** (left) and **2** (right).

Longitudinal relaxation experiment:

Sample	Diradical	Monoradical
Temperature (K)	70	70
Field (G)	3454	3478
Microwave attenuation (dB)	13	13
π/2 pulse length (ns)	20	28
Shot repetition time (ms)	20.4	6.12
Microwave Freq (GHz)	9.6946	9.7626
Video Gain (dB)	51	42

2-pulse echo decay experiment:

Sample	Diradical	Monoradical
Temperature (K)	70	70
Field (G)	3454	3478
Microwave attenuation (dB)	13	13
π/2 pulse length (ns)	20	28
Shot repetition time (ms)	3400	5.1
Microwave Freq (GHz)	9.6941	9.7634
Video Gain (dB)	51	42

Pulsed nutation experiment:

Sample	Diradical	Monoradical
Temperature (K)	70	70
Field (G)	3454	3454
Microwave attenuation (dB)	7, 10, 13, 16, 19	7, 10, 13, 16, 19
Tau (ns)	500	500
Shot repetition time (us)	3332.3	3332.3
Microwave Freq (GHz)	9.6949	9.6949
Video Gain (dB)	45	45

Relaxation experiments:

Spin–lattice relaxation times (T_{1e}) were determined using an inversion recovery pulse sequence. In this experiment, a π pulse was first applied to invert the spin populations, followed by a variable recovery delay τ, during which the magnetization relaxed toward thermal equilibrium. The remaining longitudinal magnetization was then probed using a standard π/2–π echo detection sequence. By incrementing τ and fitting the echo intensity as a function of delay time to an exponential recovery, T_{1e} values were extracted.

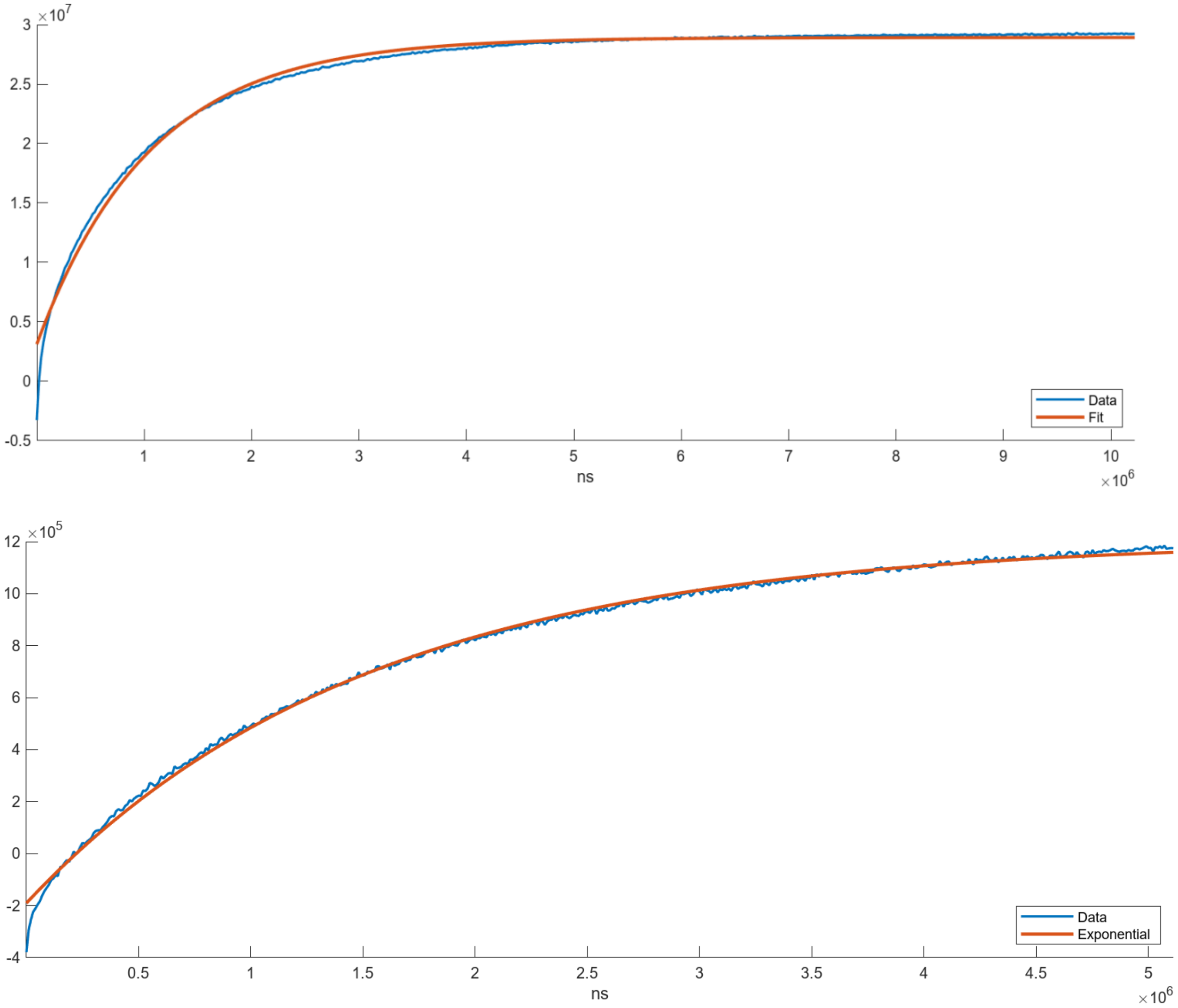

Figure S20: Inversion recovery experiments on the (top) **2** and (bottom) **1**. Fits yield electron spin–lattice relaxation times T_{1e} of 1.1 ms and 1.5 ms, respectively.

Phase memory times (T_m) were measured using a standard two-pulse Hahn echo sequence ($\pi/2 - \tau - \pi - \tau$ – echo). In this experiment, the interpulse delay τ was systematically incremented to monitor the decay of the echo intensity as a function of total evolution time. As τ increases, spin–spin interactions and environmental fluctuations lead to dephasing and a corresponding reduction in the echo amplitude. The resulting echo-decay curves were fit to exponential functions to extract T_{2e} values.

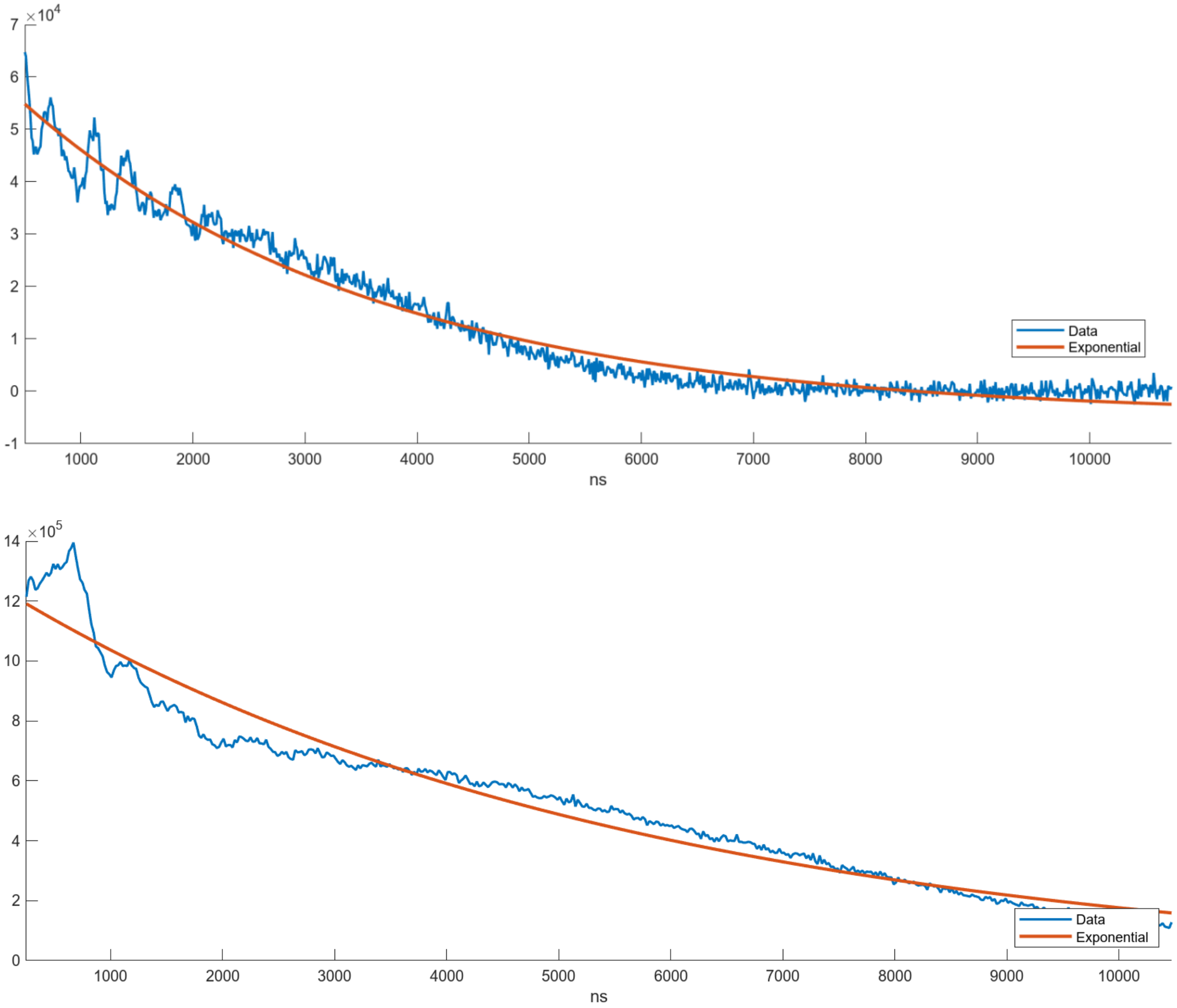

Figure S21: Two-pulse echo delay experiments on the (top) **2** and (bottom) **1**, giving a T_m of 3.2 μs and 5.6 μs, respectively. ESEEM modulations associated with ^{1}H and ^{35}Cl were observed.

Nutation experiment:

Nutation experiments were performed by applying an initial microwave pulse of variable length (t_p) to drive coherent spin rotations, followed by a fixed-delay π/2–π echo sequence for detection. As t_p was incremented, oscillations in the echo intensity arising from Rabi nutation were recorded. The time-domain data were baseline corrected, zero-filled, and Fourier transformed to obtain the corresponding nutation frequency.

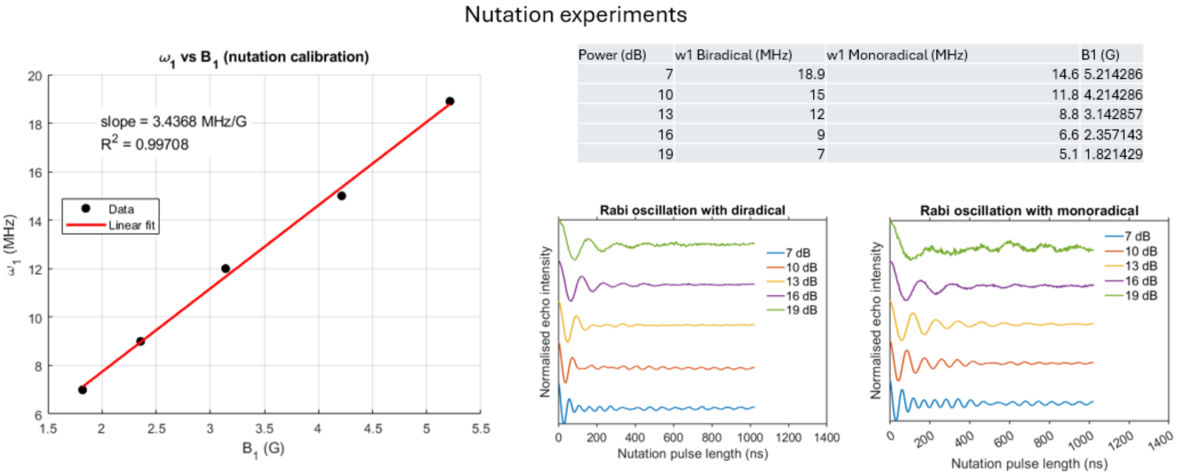

Power (dB)	w1 Biradical (MHz)	w1 Monoradical (MHz)	B1 (G)
7	18.9	14.6	5.214286
10	15	11.8	4.214286
13	12	8.8	3.142857
16	9	6.6	2.357143
19	7	5.1	1.821429

Figure S22: Nutation experiments on **1** and **2** at different microwave power levels, at 70 K.

Double quantum coherence (DQC) experiment

DQC EPR measurement were performed on a Bruker ELEXSYS E580 X-band EPR spectrometer, using the six-pulse sequence, π/2- τ1-π- τ1-π/2-τ3-π-τ3-π/2-τ2-π-τ2-echo. A π/2 pulse of 12 ns and π pulse of 24 ns were utilized for the experiment. No increment was applied to suppress the nuclear modulation due to the lack of SNR. For analysis, a high order polynomial function was used to fit the low frequency nuclear modulations in the time domain trace. This is the subtracted from the original data. The result is then processed using DeerAnalysis 2022. The background removal is chosen according to the best validation.

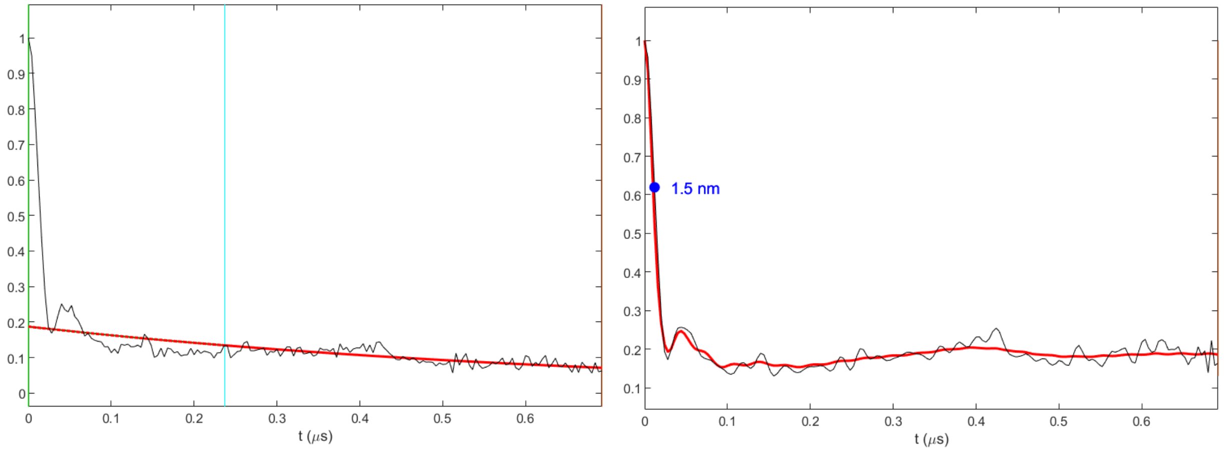

Figure S23: X-band DQC (left) time domain results and (right) fitting after background correction at 70 K for compound **2**.

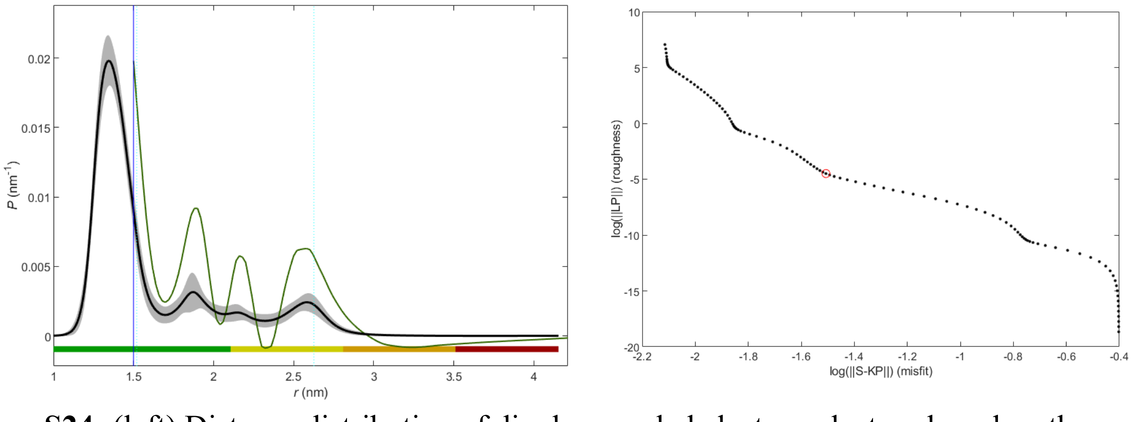

Figure S24: (left) Distance distribution of dipolar coupled electron-electron based on the Tikhonov regularization analysis on the DQC results using the DeerAnalysis 2022 package. Green curve indicates the suppressed distance distribution assuming the peak below 1.5 nm is attributed to 1H ESEEM. (right) L-curve shows a less pronounced corner, indicating a broad distribution.

The ESEEM data is the same as the echo-delay results for Tm measurements. The time domain trace is first background corrected using an exponential decay function. A Hamming window is then applied to suppress the noise. The result is then Fourier-transformed to the frequency domain and the absolute value of the complex spectrum is calculated and plotted as a function of frequency.

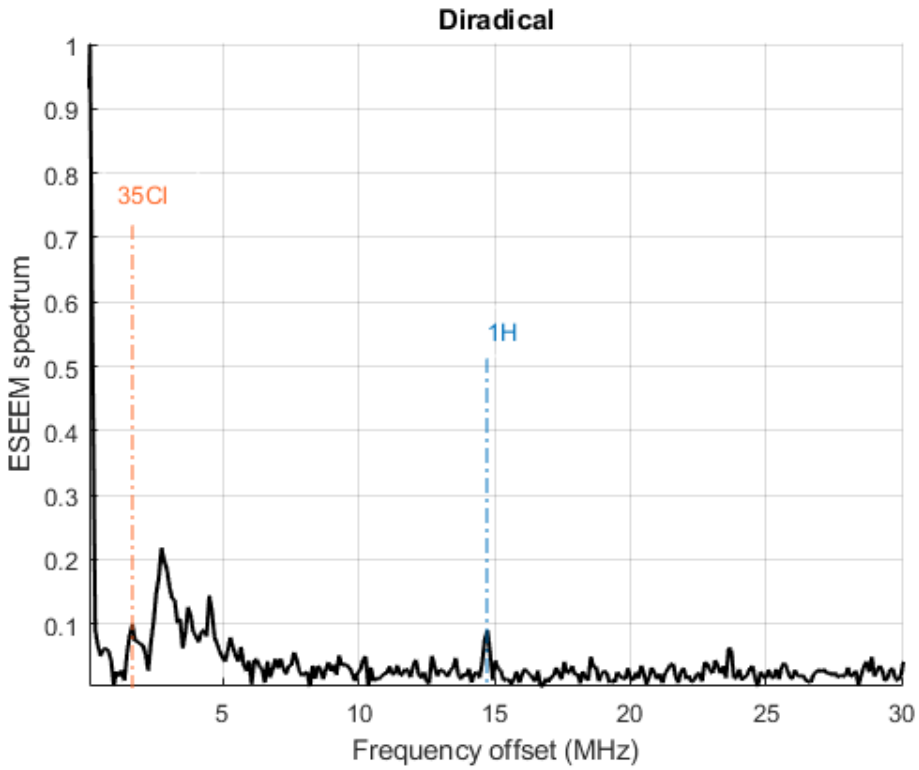

Figure S25: ESEEM spectrum from echo delay experiment for diradical at X-band and 70 K.

OPTICAL SPECTROSCOPY

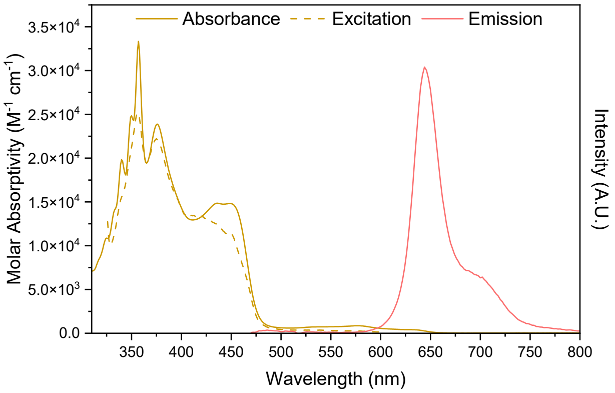

Figure S26: UV-Vis, emission (450 nm), and excitation (648 nm) spectra of Compound **1** in cyclohexane.

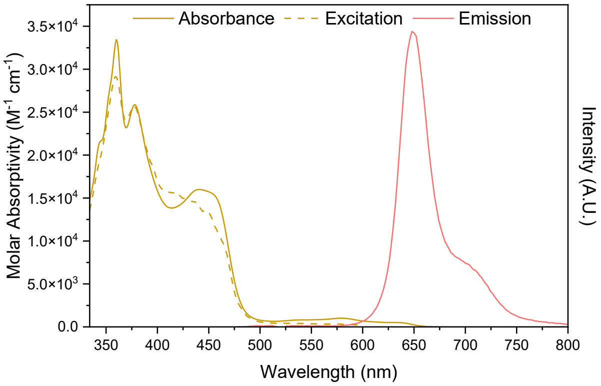

Figure S27: UV-Vis, emission (450 nm), and excitation (648 nm) spectra of Compound **1** in toluene.

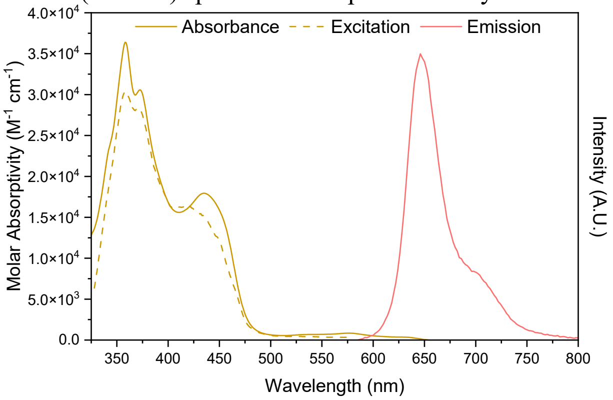

Figure S28: UV-Vis, emission (450 nm), and excitation (648 nm) spectra of Compound **1** in acetone.

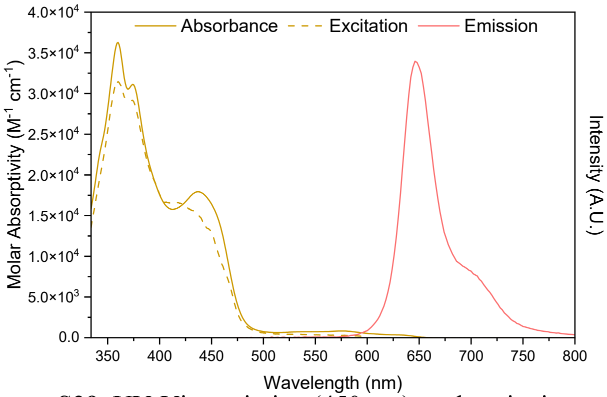

Figure S29: UV-Vis, emission (450 nm), and excitation (648 nm) spectra of Compound **1** in DCM.

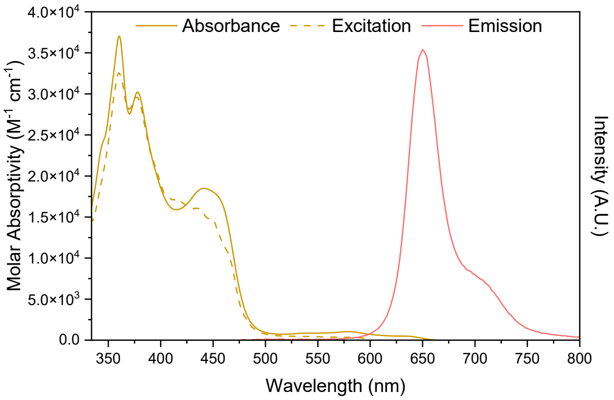

Figure S30: UV-Vis, emission (450 nm), and excitation (648 nm) spectra of Compound **1** in chlorobenzene.

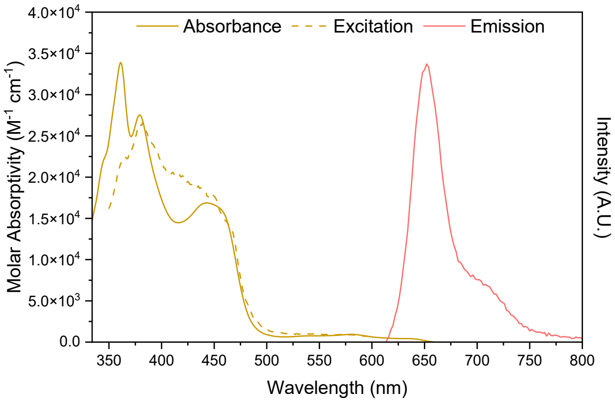

Figure S31: UV-Vis, emission (450 nm), and excitation (648 nm) spectra of Compound **1** in bromobenzene.

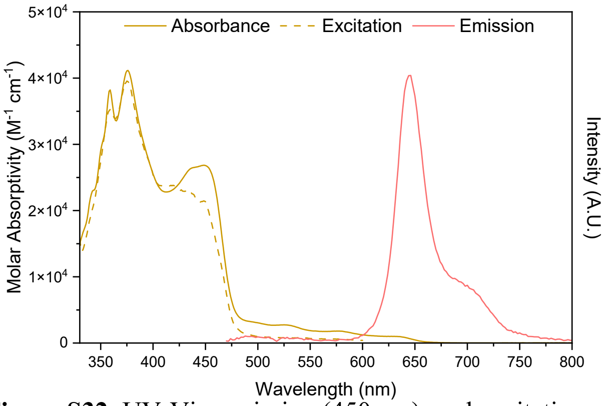

Figure S32: UV-Vis, emission (450 nm), and excitation (648 nm) spectra of Compound **2** in cyclohexane.

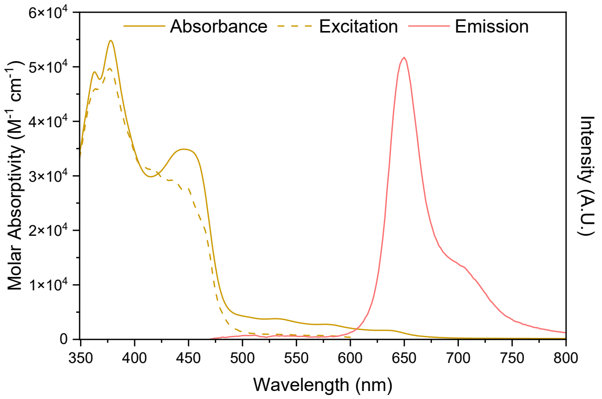

Figure S33: UV-Vis, emission (450 nm), and excitation (648 nm) spectra of Compound **2** in toluene.

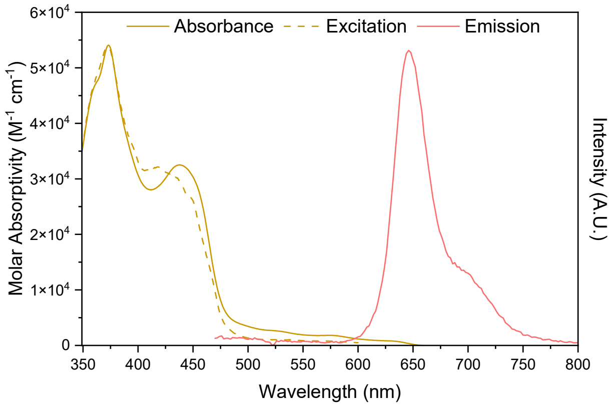

Figure S34: UV-Vis, emission (450 nm), and excitation (648 nm) spectra of Compound **2** in acetone.

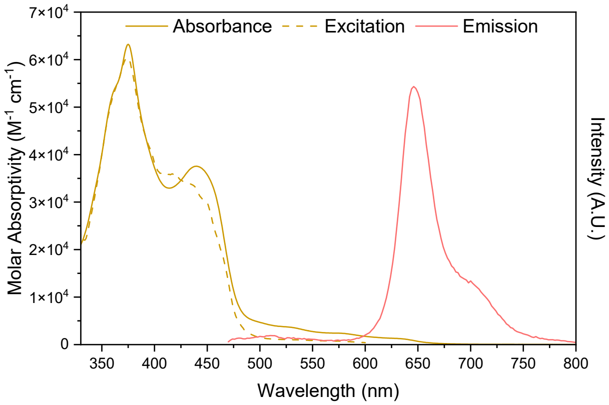

Figure S35: UV-Vis, emission (450 nm), and excitation (648 nm) spectra of Compound **2** in DCM.

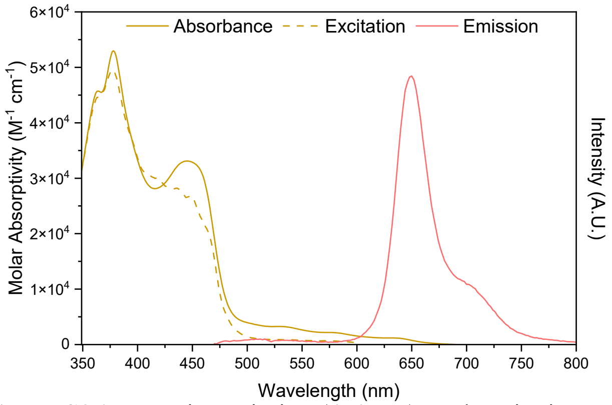

Figure S36: UV-Vis, emission (450 nm), and excitation (648 nm) spectra of Compound **2** in chlorobenzene.

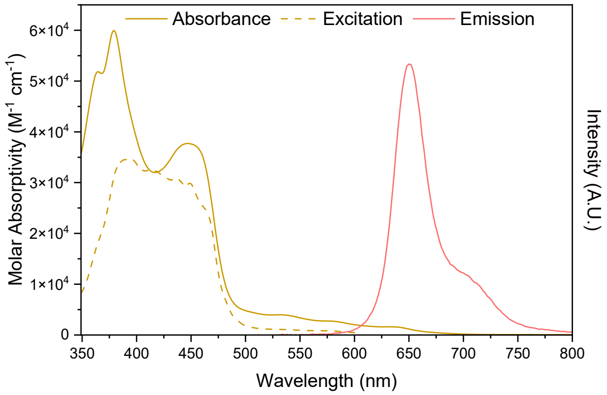

Figure S37: UV-Vis, emission (450 nm), and excitation (648 nm) spectra of Compound **2** in bromobenzene.

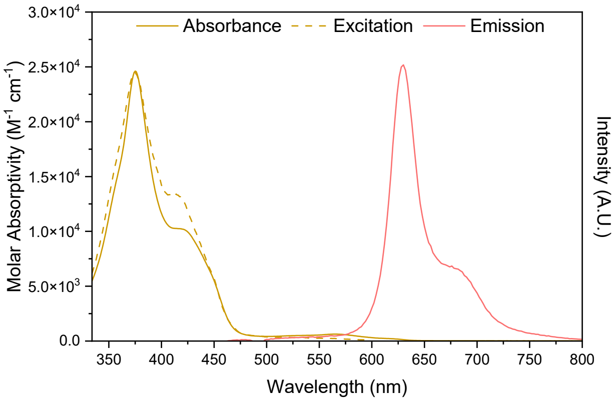

Figure S38: UV-Vis, emission (430 nm), and excitation (630 nm) spectra of Compound **3** in cyclohexane.

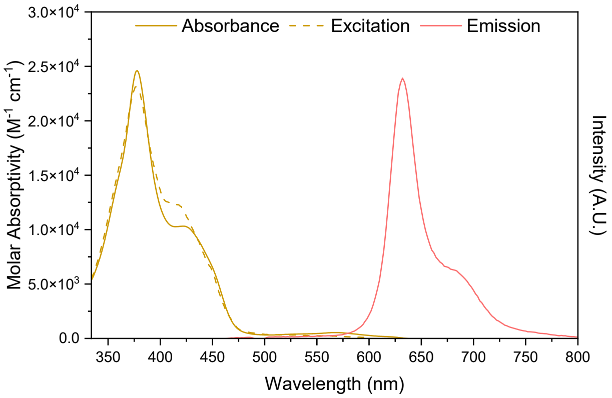

Figure S39: UV-Vis, emission (430 nm), and excitation (630 nm) spectra of Compound **3** in toluene.

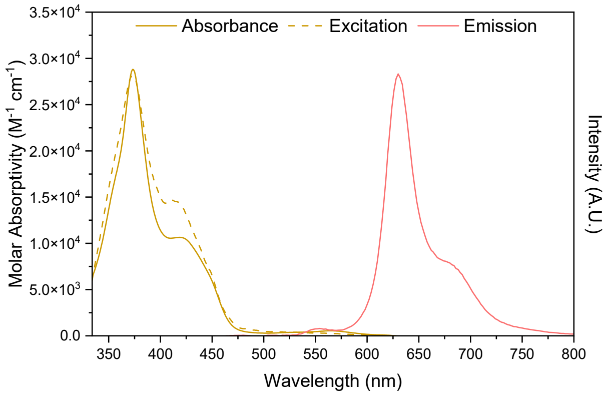

Figure S40: UV-Vis, emission (430 nm), and excitation (630 nm) spectra of Compound **3** in acetone.

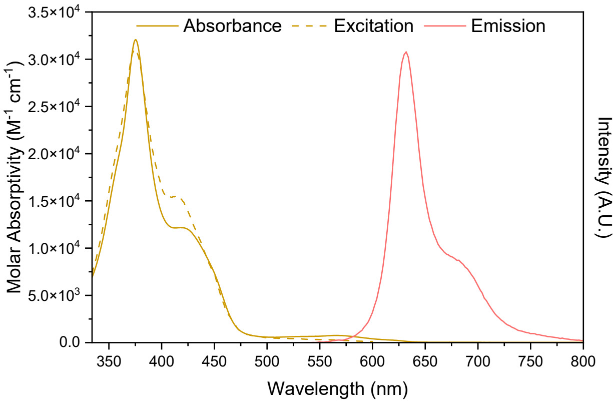

Figure S41: UV-Vis, emission (430 nm), and excitation (630 nm) spectra of Compound **3** in DCM.

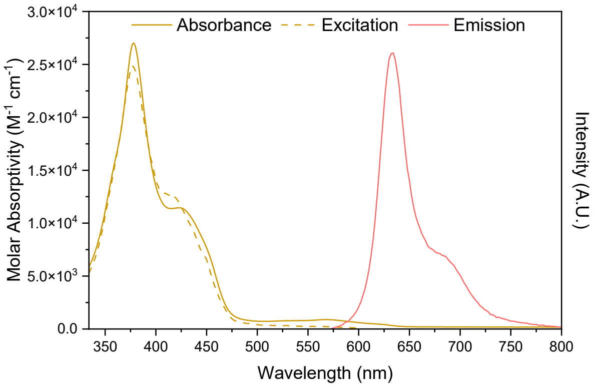

Figure S42: UV-Vis, emission (430 nm), and excitation (630 nm) spectra of Compound **3** in chlorobenzene.

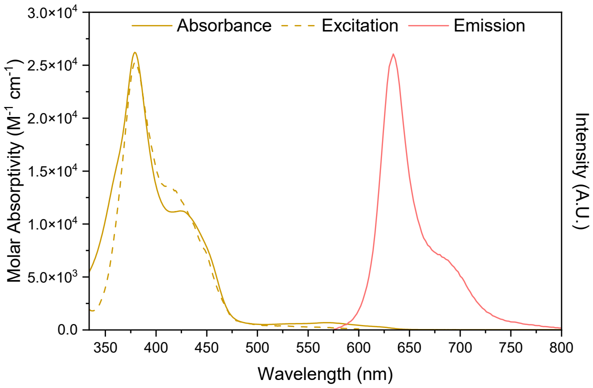

Figure S43: UV-Vis, emission (430 nm), and excitation (630 nm) spectra of Compound **3** in bromobenzene.

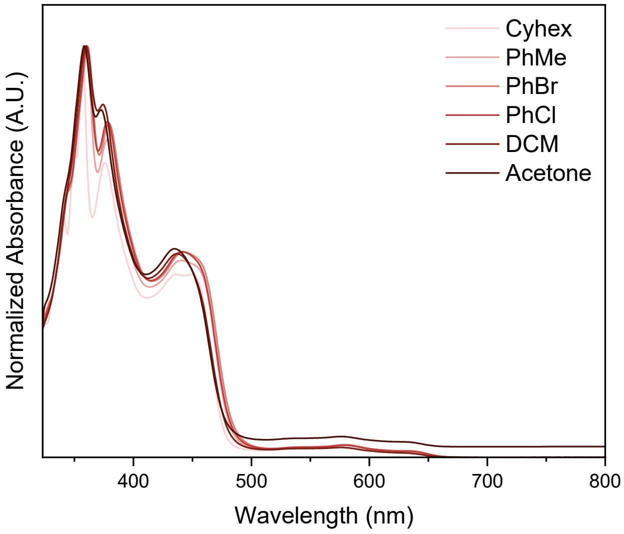

Figure S44: Normalized absorbance of compound **1** in different solvents. Solvents are ordered by increasing polarizability.

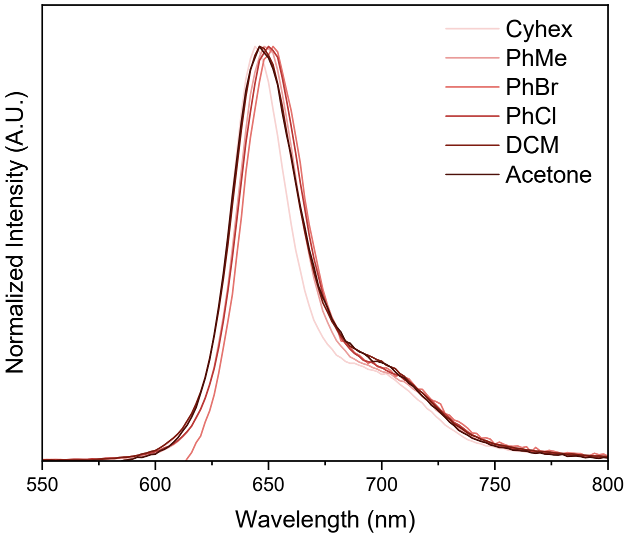

Figure S45: Normalized fluorescence of compound **1** in different solvents. Solvents are ordered by increasing polarizability.

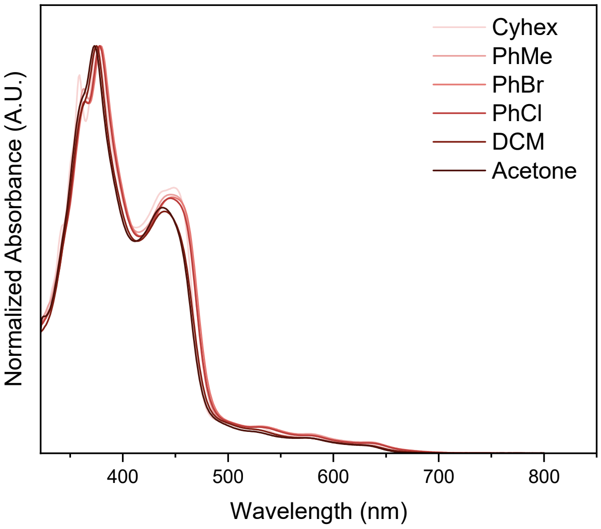

Figure S46: Normalized absorbance of compound **2** in different solvents. Solvents are ordered by increasing polarizability.

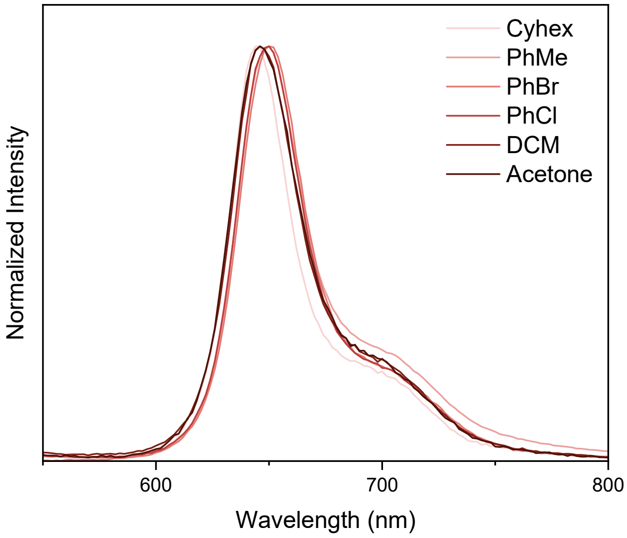

Figure S47: Normalized fluorescence of compound **2** in different solvents. Solvents are ordered by increasing polarizability.

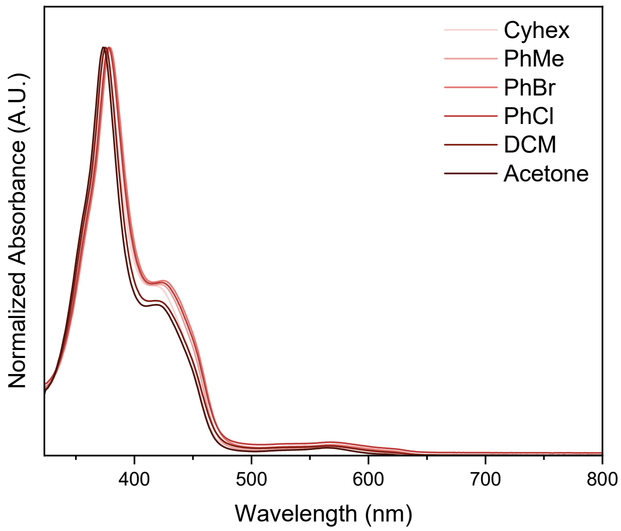

Figure S48: Normalized absorbance of compound **3** in different solvents. Solvents are ordered by increasing polarizability.

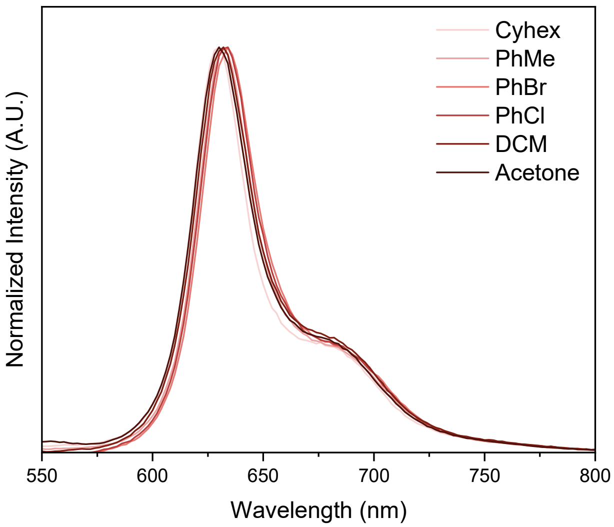

Figure S49: Normalized fluorescence of compound **3** in different solvents. Solvents are ordered by increasing polarizability.

Table S5: Summary of photoluminescent quantum yield (ϕ_{PL}) data for compounds **1** and **2** (450 nm excitation) and (**3**).

Solvent	Compound	ϕ_{PL}	Compound	ϕ_{PL}	Compound	ϕ_{PL}
**Cyclohexane**	**1**	1.1%	**2**	1.1%	**3**	0.7%
**Toluene**	**1**	1.4%	**2**	1.4%	**3**	1.1%
**Bromobenzene**	**1**	1.2%	**2**	1.2%	**3**	1.1%
**Chlorobenzene**	**1**	1.1%	**2**	1.1%	**3**	1.1%
**DCM**	**1**	0.08%	**2**	0.08%	**3**	0.09%
**Acetone**	**1**	0.03%	**2**	0.03%	**3**	0.09%

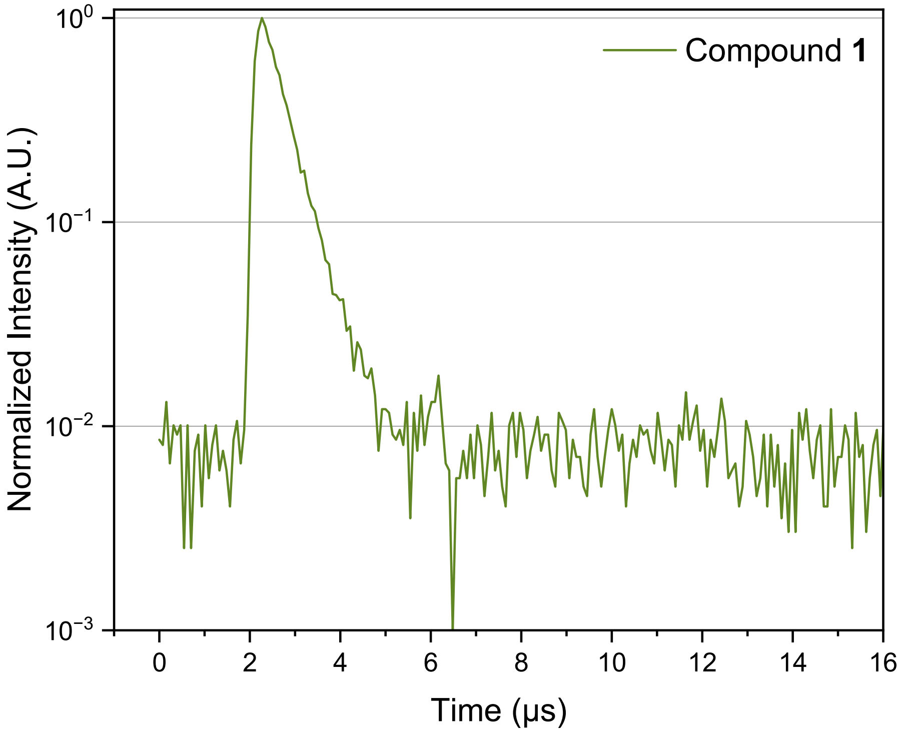

Figure S50: Fluorescence lifetime measurement of compound **1** under argon atmosphere in toluene. τ = 4.9 ns.

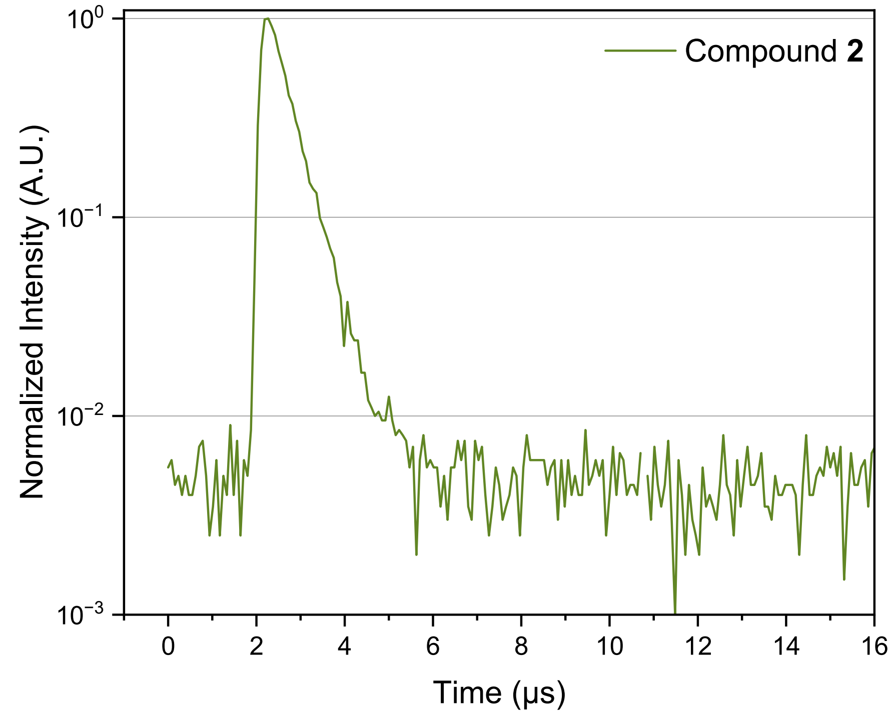

Figure S51: Fluorescence lifetime measurement of compound **2** under argon atmosphere in toluene. τ = 4.8 ns.

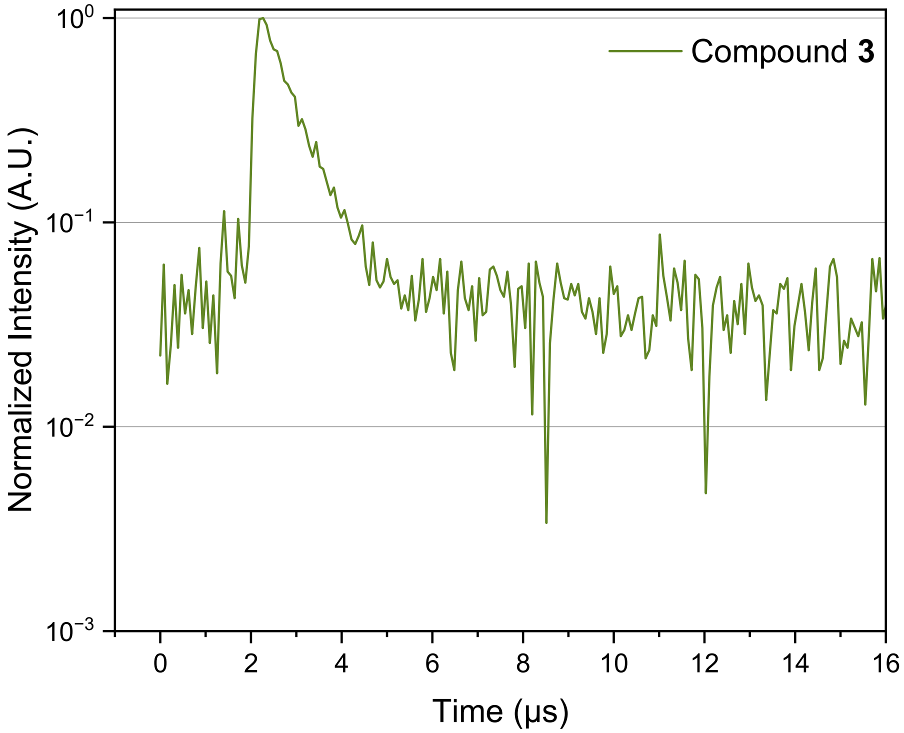

Figure S52: Fluorescence lifetime measurement of compound **3** under argon atmosphere in toluene. τ = 6.4 ns.

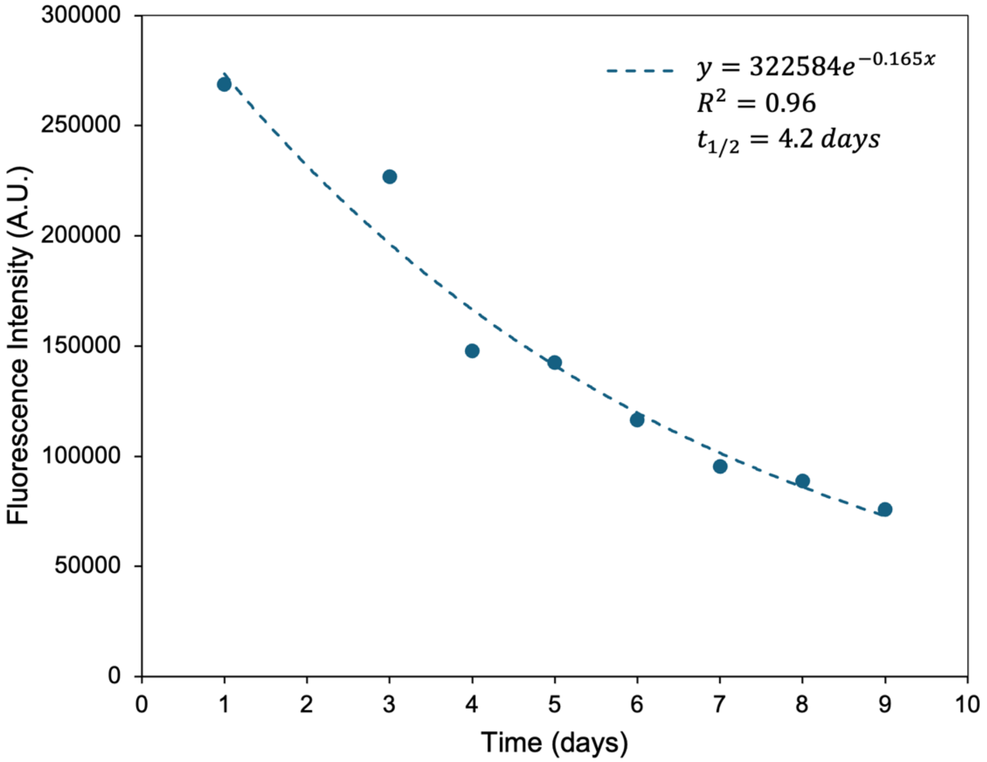

Figure S53: Fluorescence intensity decay of the peak at 648 nm (450 nm excitation) as a function of time for **1**. Half-life was fitted to a first order decay model.

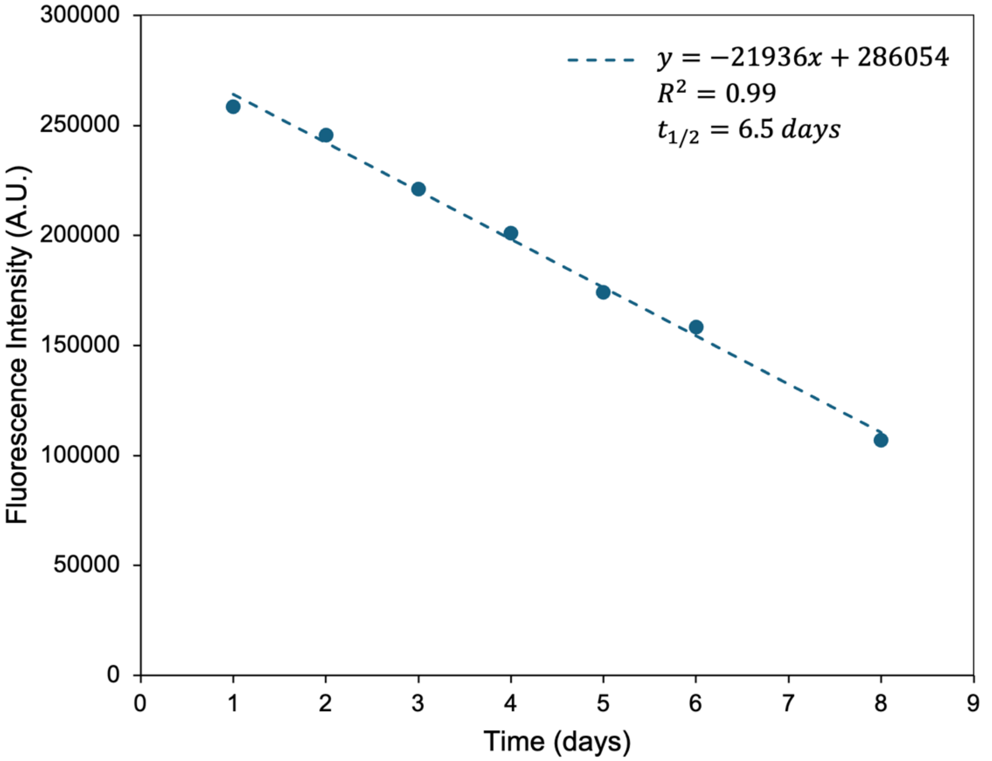

Figure S54: Fluorescence intensity decay 648 nm (450 nm excitation) as a function of time for **2**. Half-life was fitted to a zeroth order decay model.

COMPUTATIONAL DETAILS

All calculations were carried out using the ORCA 5.0.3 program[11] unless otherwise noted. Geometry optimizations were performed with density functional theory (DFT) at the (BS-)UB3LYP-D3(BJ)[12,13]/def2-SVP[14,15] level of theory using CPCM solvation[16,17]. Geometries were optimized for doublet (compounds **1** and **3**), open shell singlet (OSS), and triplet states (both for compound **2**). Open-shell singlets were modeled with broken-symmetry (BS) DFT. Harmonic vibrational frequency analyses were conducted to ensure that local minima were obtained. We found two geometries for compound **1** and three geometries for compound **2**. For both compound **1** and **2** the lowest energy conformer was found to be the structure with the nitrogen on the pyridyl ring pointed towards the side of the triptycene aromatic ring that has one proton. We used the lowest energy conformation for each structure for subsequent calculations.

Spin density analysis was carried out at the UB3LYP-D3(BJ)/def2-SVP,CPCM(toluene) level of theory (broken symmetry for singlet states). Fractional orbital density (FOD) analysis was carried out at the TPSS/def2-SVP level of theory with an occupational smear temperature of 5000 K. To calculate the singlet-triplet energy gap of **2**, complete active space self-consistent field (CASSCF) calculations were carried out on DFT optimized geometries of the singlet and triplet state. We assumed alternancy symmetry[18] of the radicals when selecting the active space, i.e. the active space should comprise of $4n+2$ for a trityl diradical. An active space of 10 electrons and 10 orbitals was chosen for compound **2** for computational efficiency. Time dependent (TD)-DFT calculations were carried out at the UB3LYP-D3(BJ)/def2-SVP,CPCM(toluene) level of theory with the Tamm-Dancoff approximation (TDA). Only the Franck-Condon state was analyzed. Orbital analysis was performed using VMD.[19,20] Electron-hole analysis[21] was performed using Multiwfn.[22]

---

[11] Neese, F.; Wennmohs, F.; Becker, U.; Riplinger, C. The ORCA quantum chemistry program package. *J. Chem. Phys.* **2020**, *152*, 224108.

[12] Grimme, S.; Ehrlich, S.; Goerigk, L. Effect of the damping function in dispersion corrected density functional theory. *J. Comput. Chem.* **2011**, *32* (7), 1456-1465.

[13] Grimme, S.; Antony, J.; Ehrlich, S.; Krieg, H. A consistent and accurate ab initio parametrization of density functional dispersion correction (DFT-D) for the 94 elements H-Pu. *J. Chem. Phys.* **2010**, *132* (15).

[14] Weigend, F.; Ahlrichs, R. Balanced basis sets of split valence, triple zeta valence and quadruple zeta valence quality for H to Rn: Design and assessment of accuracy. *Physical Chemistry Chemical Physics* **2005**, *7* (18), 3297-3305.

[15] Weigend, F. Accurate Coulomb-fitting basis sets for H to Rn. *Physical Chemistry Chemical Physics* **2006**, *8* (9), 1057-1065.

[16] Barone, V.; Cossi, M. Quantum Calculation of Molecular Energies and Energy Gradients in Solution by a Conductor Solvent Model. *J. Phys. Chem. A* **1998**, *102*, 1995–2001

[17] Garcia-Rates, M.; Neese,F. Effect of the Solute Cavity on the Solvation Energy and its Derivatives within the Framework of the Gaussian Charge Scheme. *J. Comput. Chem*. **2020**, *41*, 922–939.

[18] Poh, Y. R.; Morozov, D.; Kazmierczak, N. P.; Hadt, R. G.; Groenhof, G.; Yuen-Zhou, J., Alternant hydrocarbon diradicals as optically addressable molecular qubits. *J. Am. Chem. Soc.* **2024,** *146*, 15549-15561.

[19] VMD 1.9.4. https://www.ks.uiuc.edu/Research/vmd/

[20] Humphrey, W.; Dalke, A.; Schulten, K., VMD - Visual Molecular Dynamics, *J. Molec. Graphics,* **1996**, *14*, 33–38.

[21] Liu, Z.; Lu, T.; Chen, Q. An sp-hybridized all-carboatomic ring, cyclo[18]carbon: Electronic structure, electronic spectrum, and optical nonlinearity. *Carbon* **2020**, *165*, 461-467.

[22] Lu, T.; Chen, F. Multiwfn: A multifunctional wavefunction analyzer. *J. Comput. Chem.* **2012**, *33* (5), 580-592.

Conformational analysis

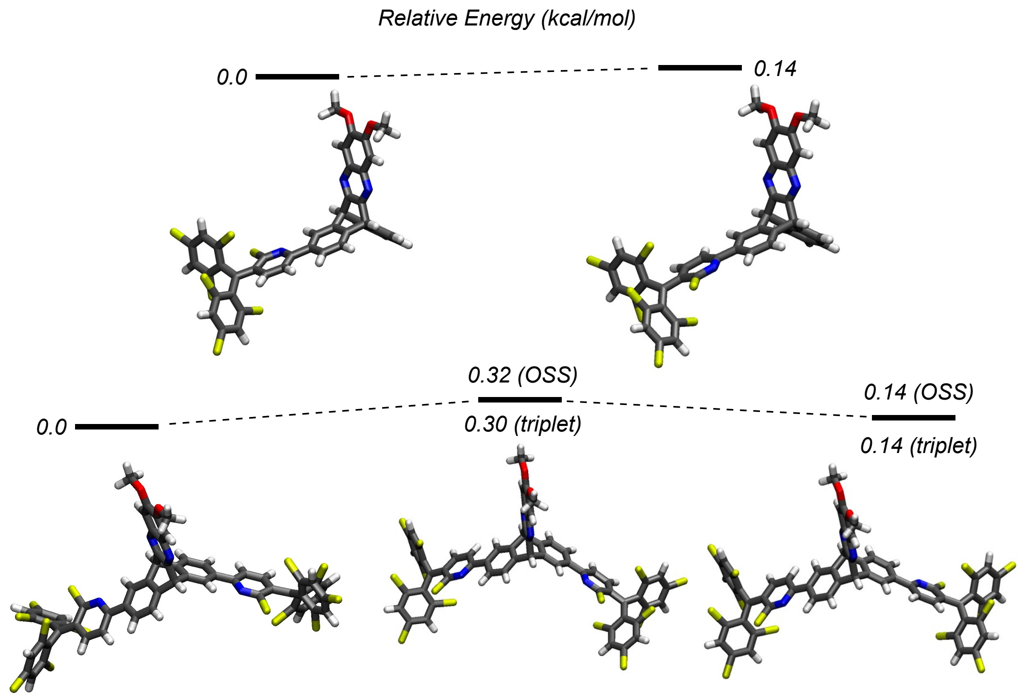

Figure S55: Energy level diagram for various DFT optimized geometries at the (BS-)UB3LYP-D3(BJ)/def2-SVP level of theory with different orientations of the pyridyl ring. *Top*: Computations are done for the doublet state of compound **1**. *Bottom:* The energies for both the open shell singlet and the triplet states are shown for compound **2**. Images are generated from the optimized geometries of the triplet state.

Spin density plots

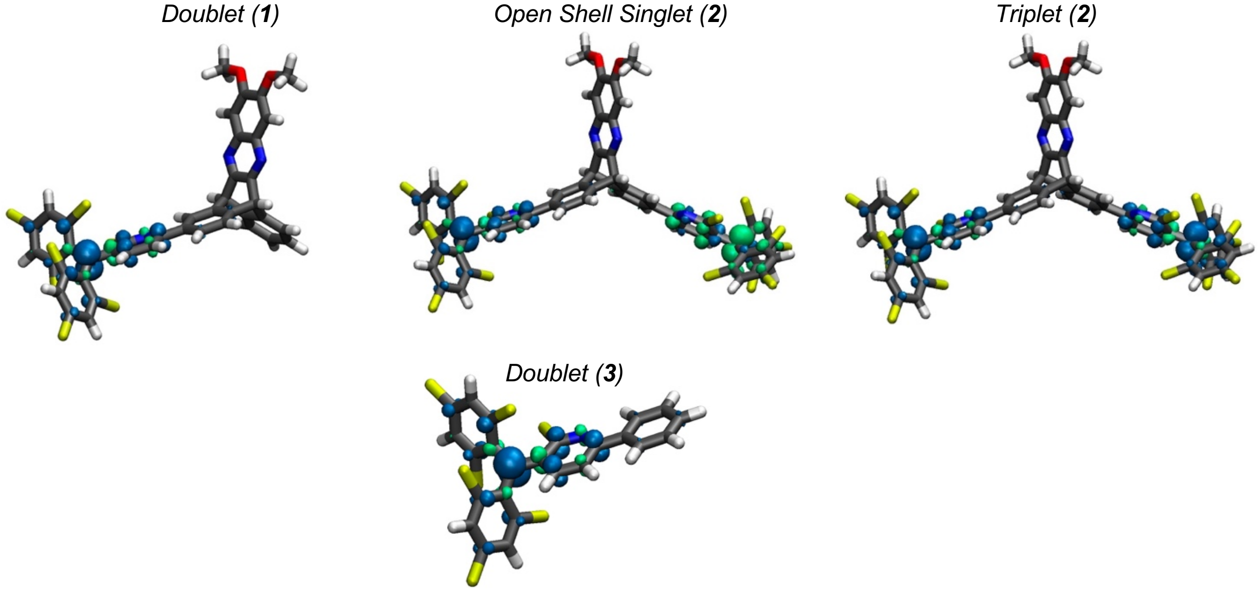

Figure S56: Spin density distribution calculated at the UB3LYP-D3(BJ)/def2-SVP,CPCM(toluene) level of theory (broken symmetry for singlet states) (isosurface = ± 0.005 $e\ a_0^3$)Top row: *Left*: the doublet state of **1**. *Center*: singlet state of **2**. *Right*: triplet state of **2**. Bottom row: the doublet state of **3**.

Fractional occupation number weighted electron density (FOD)

FOD analysis can identify systems with significant static electron correlation. FOD plots show the contribution of 'hot' (strongly correlated) electrons. While TPSS/def2-TZVP is the default method for FOD analysis, in this work TPSS/def2-SVP was employed for computational feasibility. It was shown in the original FOD paper[23] that FOD results are not strongly basis set dependent, which suggests the present results are reliable.

Doublet (**1**) Singlet (OSS) (**2**) Triplet (**2**)

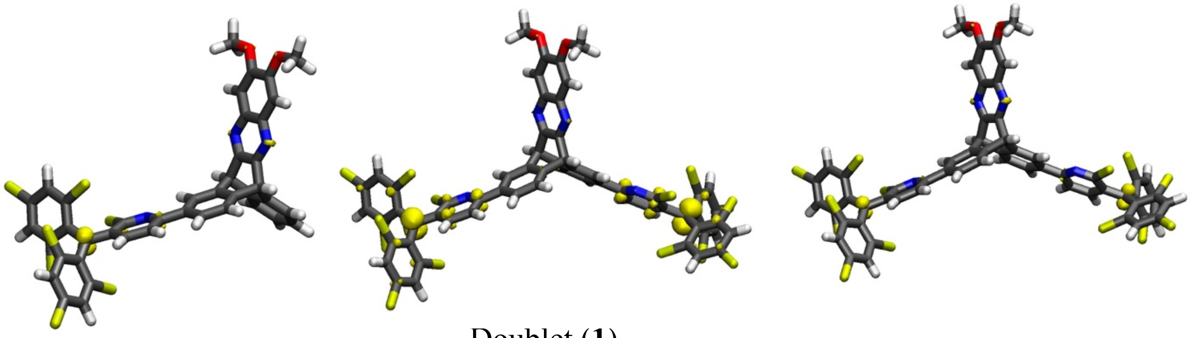

Doublet (**1**)

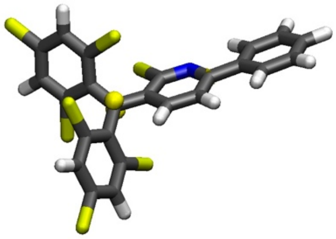

Figure S57: FOD plots for <u>Top:</u> the doublet (left) configuration of compound **1** as well as the singlet (middle) and triplet (right) configurations of compound **2** and Bottom: the doublet configuration of **3** at the TPSS/def2-SVP level of theory with a sear temperature of 5000 K (isosurface = 0.005 $e\ a_0^3$).

The key quantity from FOD analysis is r^{FOD}, the fractional occupation number weighted electron density. Results are presented in Table S5. The large values of r^{FOD} for all considered electronic states of **1** and **2** are consistent with significant strongly correlated electrons, which indicates that multireference methods are most appropriate.

Table S6: FOD results for **1** and **2** calculated at the TPSS/def2-SVP level of theory with a smear temperature of 5000 K.

r^{FOD}	Doublet	Singlet	Triplet
**1**	0.91	--	--
**2**	--	2.55	1.47
**3**	0.55	--	--

---

[23] Grimme, S.; Hansen, A. A practicable real-space measure and visualization of static electron-correlation effects. *Angew. Chem. Int. Ed.* **2015**, *54* (42), 12308-12313.

Complete Active Space Self-Consistent Field (CASSCF) Results

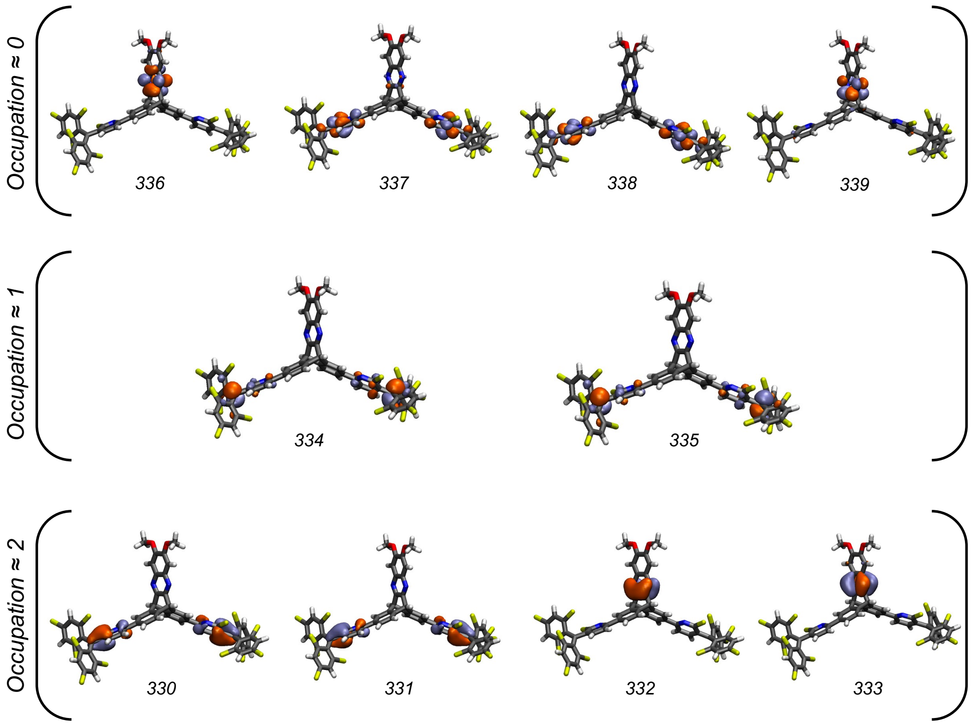

Figure S58: Active space orbitals (isosurface = ± 0.03 $e\ a_0^3$). for **2**, triplet state, calculated at the CASSCF(10,10)/def2-SVP level of theory.

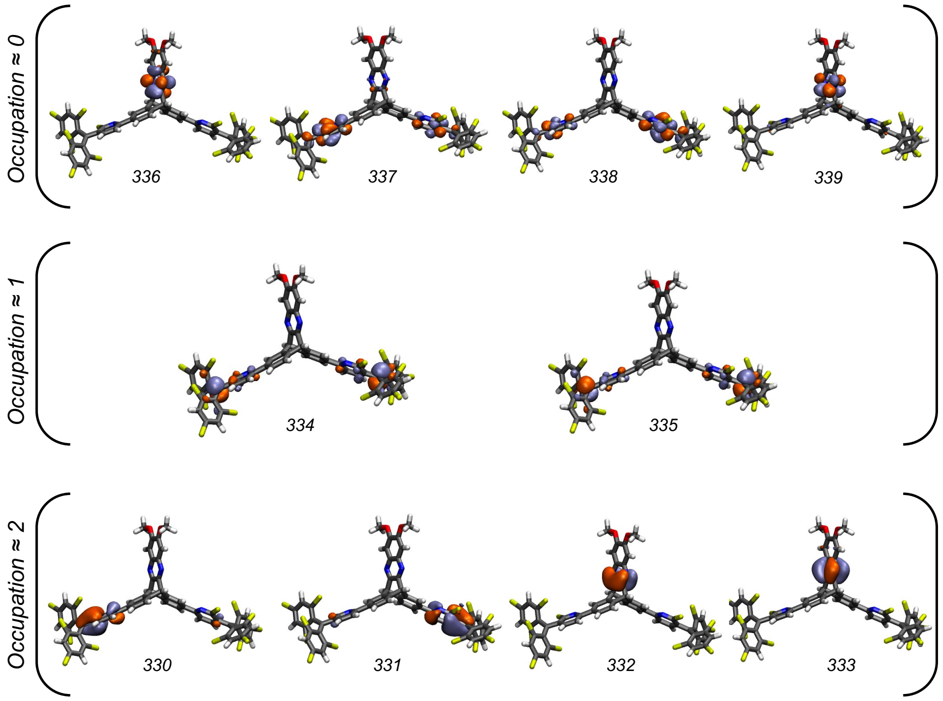

Figure S59: Active space orbitals (isosurface = ± 0.03 $e\ a_0^3$) for **2**, singlet state, calculated at the CASSCF(10,10)/def2-SVP level of theory.

Frontier Molecular Orbitals and Time Dependent – Density Functional Theory (TD-DFT)

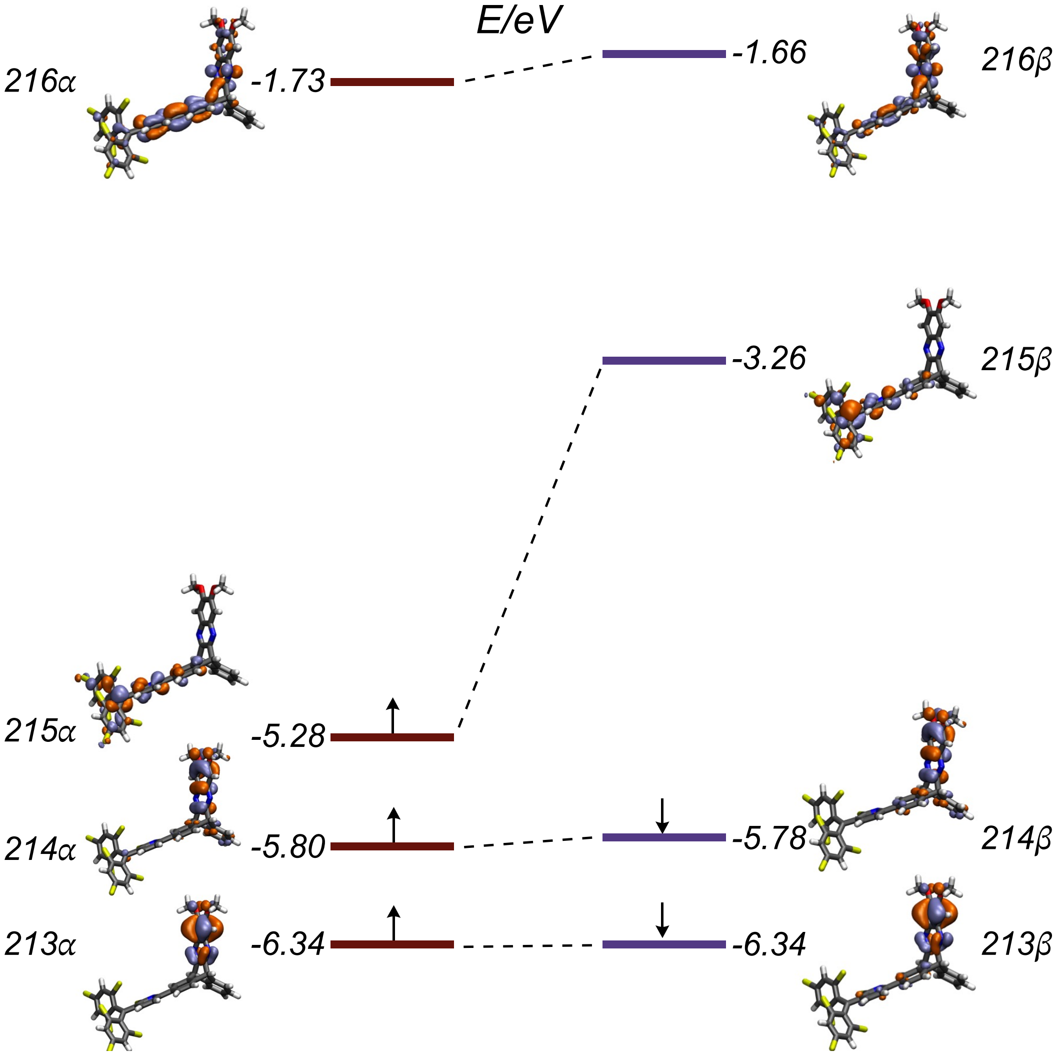

Figure S60: Frontier molecular orbitals (isosurface = ± 0.03 $e\ a_0^3$) for the doublet state of compound **1** calculated at the UB3LYP-D3(BJ) / def2-SVP, CPCM(toluene) level of theory.

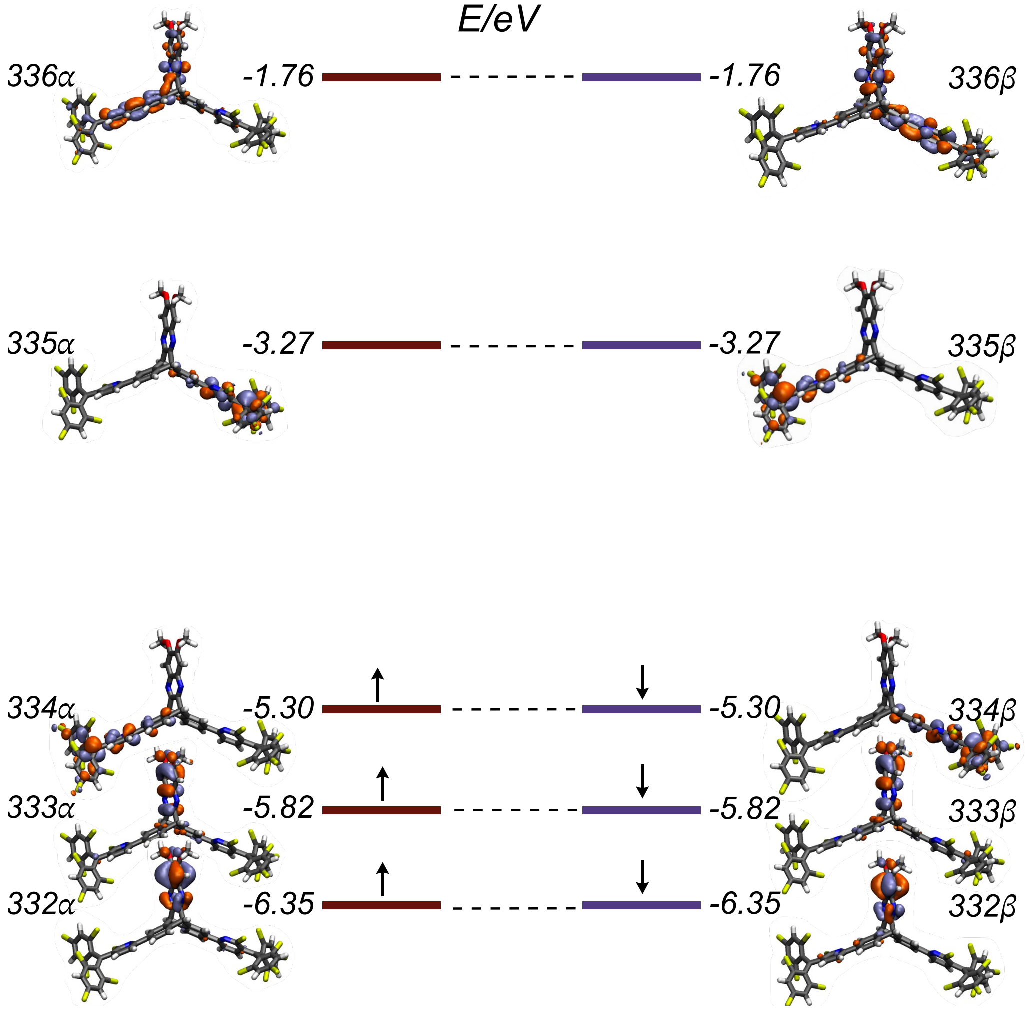

Figure S61: Frontier molecular orbitals (isosurface = ± 0.03 $e\ a_0^3$) for the open shell singlet state of compound **2** calculated at the BS-UB3LYP-D3(BJ) / def2-SVP, CPCM(toluene) level of theory.

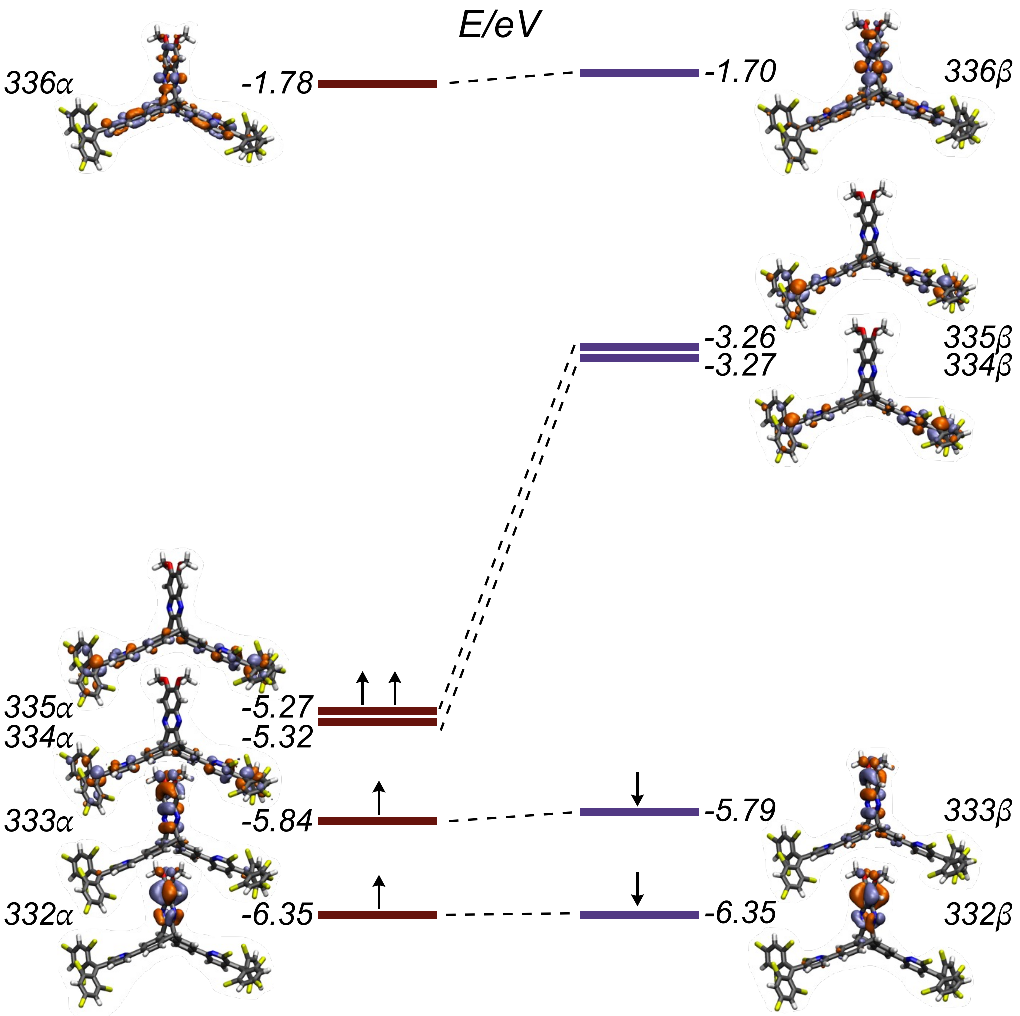

Figure S62: Frontier molecular orbitals (isosurface = ± 0.03 $e\ a_0^3$) for the triplet state of compound **2** calculated at the UB3LYP-D3(BJ) / def2-SVP, CPCM(toluene) level of theory.

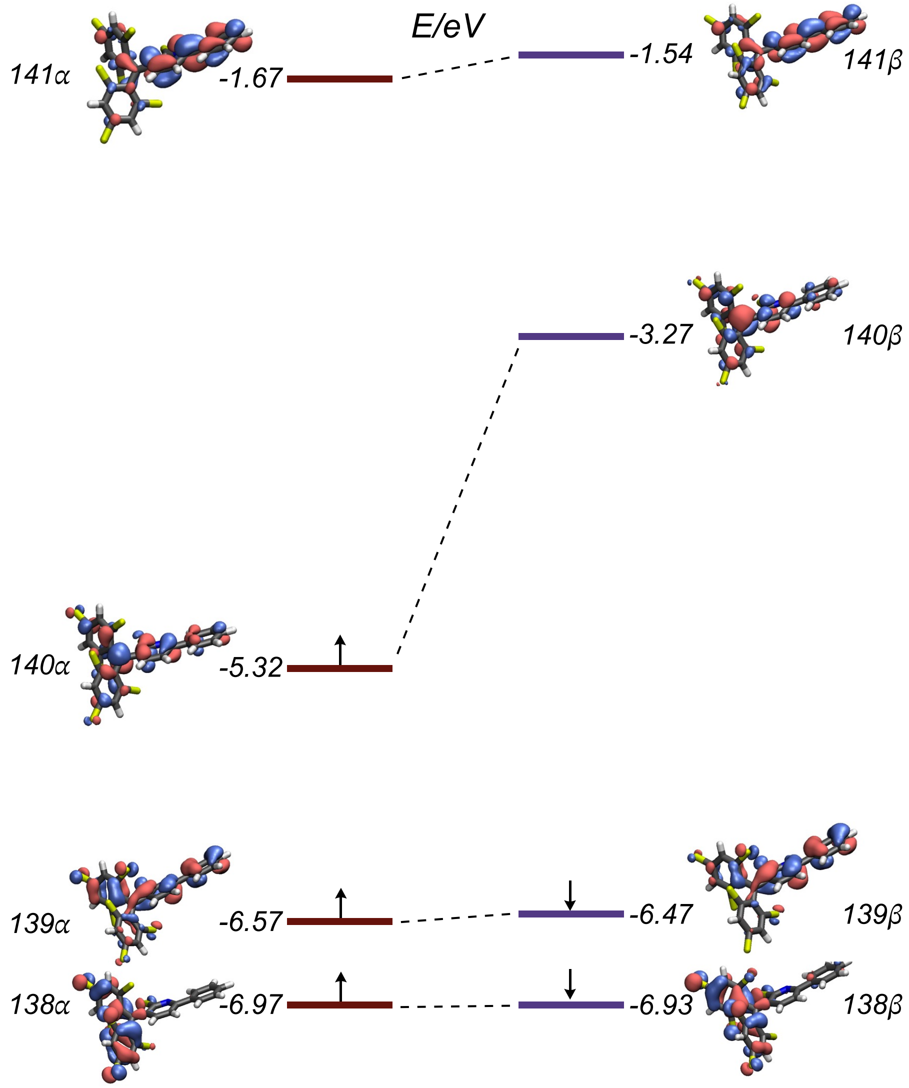

Figure S63: Frontier molecular orbitals (isosurface = ± 0.03 $e\ a_0^3$) for the triplet state of compound **3** calculated at the UB3LYP-D3(BJ) / def2-SVP, CPCM(toluene) level of theory.

Table S7: The first fifty doublet excited states (D_n) of compound **1** calculated at the UB3LYP-D3(BJ) / def2-SVP, CPCM(toluene) level of theory.

D_n	Excitation[a] (Contribution)[b]	Vertical Excitation Wavelength (nm)	f [c]
D_1	214β →215β (0.78)	573	0.1086
D_2	212β →215β (0.43)	513	0.0199
D_3	208β→215β (0.36)	450	0.0547
D_5	215α→216α (0.45)	435	0.4494
D_6	211β→215β (0.70)	432	0.0128
D_7	207β→215β (0.26)	423	0.1294
D_8	214β→216β (0.26)	419	0.1059
D_{12}	215α→218α (0.64)	387	0.2213
D_{15}	215α→219α (0.37)	380	0.0604
D_{17}	215α→219α (0.31)	367	0.0276
D_{18}	215α→217α (0.62)	366	0.0192
D_{19}	203β→215β (0.35)	364	0.0189
D_{21}	215α→220α (0.48)	352	0.0103
D_{23}	202β→215β (0.45)	336	0.0602
D_{24}	214α→216α (0.44)	333	0.2752
D_{28}	201β→215β (0.29)	319	0.0506
D_{29}	214β→217β (0.42)	317	0.0692
D_{31}	201β→215β (0.26)	310	0.0109
D_{42}	211α→216α (0.25)	288	0.0585
D_{45}	213α→216α (0.27)	286	0.0256

[a]Orbitals involved in major excitation [b]The contribution weight of orbitals less than 0.25 are not listed [c]Oscillator strengths for transitions with $f > 0.0100$.

Table S8: The first fifty singlet excited states (S_n) of compound **2** calculated at the UB3LYP-D3(BJ) / def2-SVP, CPCM(toluene) level of theory.

S_n	Excitation[a] (Contribution)[b]	Vertical Excitation Wavelength (nm)	f[c]
S_1	334α→335α (0.91)	682	0.0107
S_3	333α→335α (0.38)	565	0.1683
	333β→335β (0.34)		
S_4	333α→335α (0.35)	561	0.0481
	333β→335β (0.38)		
S_5	331β→335β (0.38)	512	0.0281
S_7	328α→335α (0.33)	450	0.1393
S_{11}	334α→336α (0.27)	438	0.8225
S_{23}	320β→335β (0.25)	398	0.0441
S_{24}	334α→339α (0.54)	387	0.2863
S_{25}	334β→339β (0.44)	387	0.1391
S_{32}	334α→337α (0.39)	371	0.0177
S_{33}	334β→337β (0.35)	371	0.0227
S_{34}	317β→335β (0.67)	367	0.0356
S_{35}	317α→335α (0.40)	367	0.0151
S_{49}	334β→345β (0.43)	343	0.0145

[a]Orbitals involved in major excitation [b]The contribution weight of orbitals less than 0.25 are not listed [c]Oscillator strengths for transitions with $f > 0.0100$.

Table S9: The first fifty triplet excited states (T_n) of compound **2** calculated at the UB3LYP-D3(BJ) / def2-SVP, CPCM(toluene) level of theory.

T_n	Excitation[a] (Contribution)[b]	Vertical Excitation Wavelength (nm)	f[c]
T_1	333β→334β (0.77)	571	0.1820
T_2	333β→335β (0.78)	569	0.0477
T_{11}	335α→337α (0.29)	437	0.8638
T_{12}	335α→336α (0.28)	434	0.1750
T_{31}	334α→336α (0.35)	375	0.0182
	335α→337α (0.25)		
T_{32}	317β→334β (0.26)	367	0.0255
T_{33}	316β→334β (0.26)	367	0.0254
T_{34}	335α→338α (0.34)	366	0.0157

[a]Orbitals involved in major excitation [b]The contribution weight of orbitals less than 0.25 are not listed [c]Oscillator strengths for transitions with $f > 0.0100$.

Table S10: The first fifty doublet excited states (D_n) of compound **3** calculated at the UB3LYP-D3(BJ) / def2-SVP, CPCM(toluene) level of theory.

D_n	Excitation[a] (Contribution)[b]	Vertical Excitation Wavelength (nm)	f[c]
D_1	139β →140β (0.79)	523	0.0745
D_2	138β →140β (0.78)	449	0.0389
D_3	140α→141α (0.38)	430	0.2218
	137β→140β (0.44)		
D_4	140α→141α (0.29)	415	0.3814
	137β→140β (0.48)		
D_6	134β→140β (0.66)	401	0.0260
D_7	140α→142α (0.32)	387	0.1581
	136β→140β (0.43)		
D_8	140α→142α (0.41)	384	0.1174
	136β→140β (0.37)		
D_9	140α→143α (0.63)	375	0.0825
D_{10}	133β→140β (0.82)	368	0.0287
D_{11}	140α→144α (0.38)	356	0.0498
	132β→140β (0.26)		
D_{12}	140α→144α (0.48)	345	0.0112
	132β→140β (0.28)		
D_{17}	131β→140β (0.32)	309	0.0131
D_{19}	130β→140β (0.38)	293	0.0139
D_{31}	139β→141β (0.27)	274	0.2566
D_{38}	139α→142α (0.26)	264	0.0282
	139β→142β (0.35)		
D_{44}	138β→141β (0.31)	256	0.0353
D_{45}	123β→140β (0.57)	256	0.0161
D_{48}	137α→141α (0.31)	252	0.0183

[a]Orbitals involved in major excitation [b]The contribution weight of orbitals less than 0.25 are not listed [c]Oscillator strengths for transitions with $f > 0.0100$.

Electron-Hole Analysis

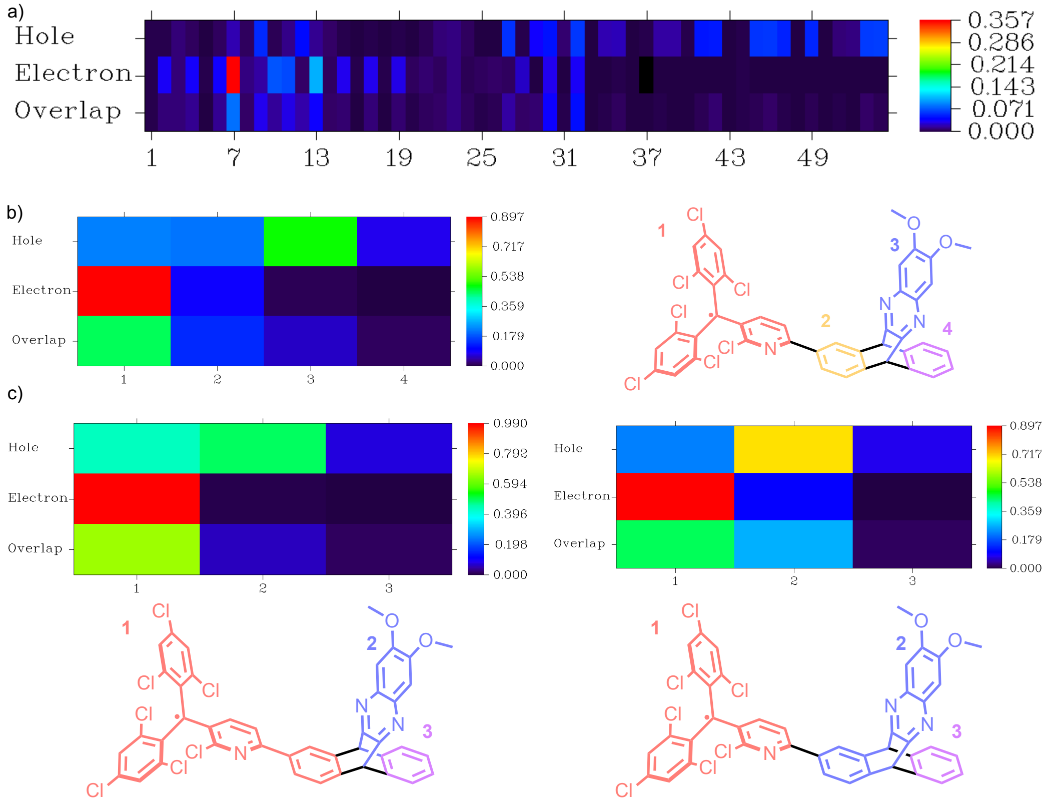

Figure S64: Electron-hole analysis for $D_0 \rightarrow D_1$ for compound **1**. a) Electron-hole heat map for all atoms in compound **1**, hydrogens are omitted. b) Electron-hole heat map (left) with fragment definition (right) where the triptycene aryl fragments are defined separately. c) Electron-hole heat maps (top) with fragment definitions (bottom) where the triptycene aryl group is included with the trityl moiety (left) or the donor moiety (right).

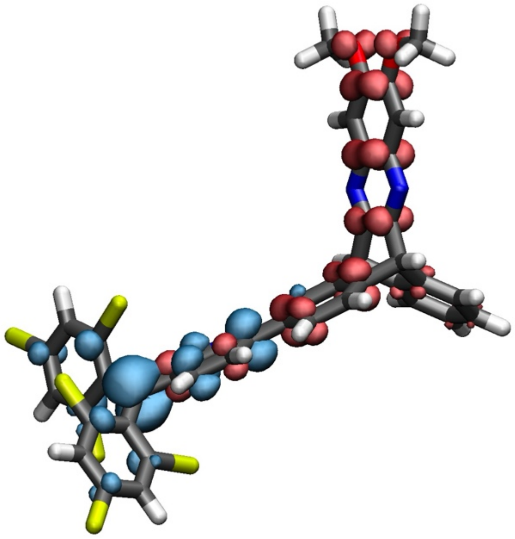

Figure S65: Schematic of orbital contribution to the calculated electron (blue) and hole (red) distribution to the $D_0 \rightarrow D_1$ transition for compound **1** (isosurface = 0.002 $e\ a_0^3$).

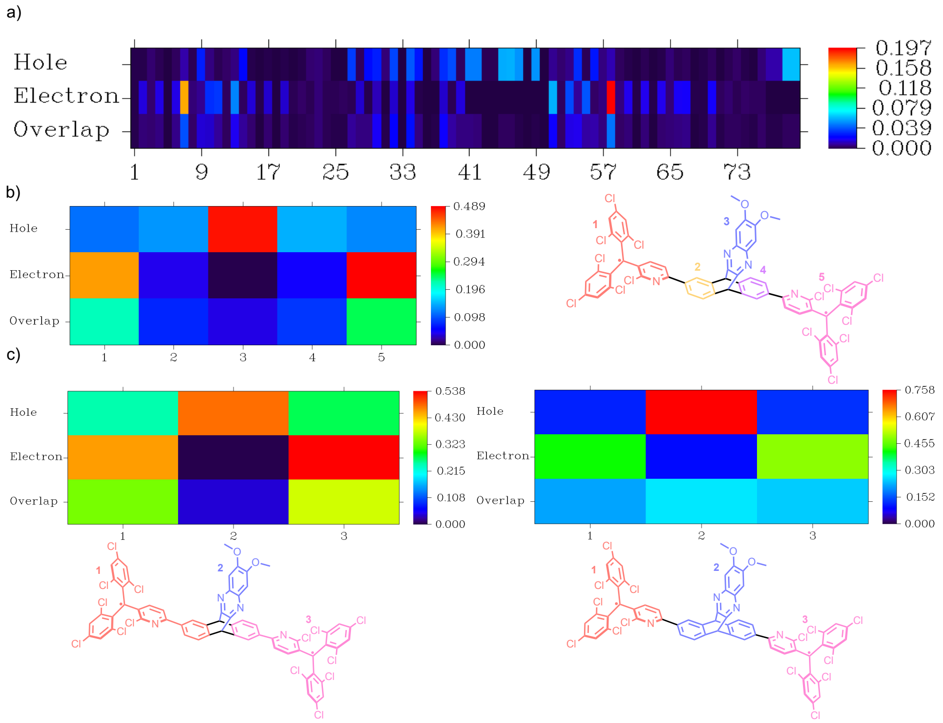

Figure S66: Electron-hole analysis for $T_0 \rightarrow T_1$ for compound **2**, state 1. a) Electron-hole heat map for all atoms in compound **2**, hydrogens are omitted. b) Electron-hole heat map (left) with fragment definition (right) where the triptycene aryl fragments are defined separately. c) Electron-hole heat maps (top) with fragment definitions (bottom) where the triptycene aryl group is included with the trityl moiety (left) or the donor moiety (right).

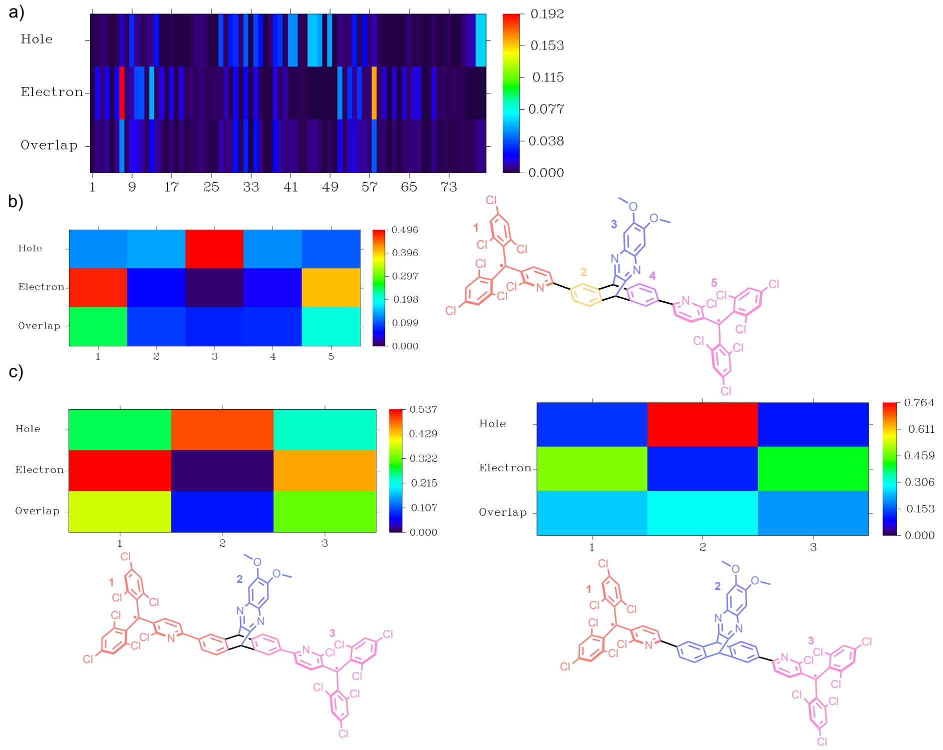

Figure S67: Electron-hole analysis for $T_0 \rightarrow T_1$ for compound **2**, state 2. a) Electron-hole heat map for all atoms in compound **2**, hydrogens are omitted. b) Electron-hole heat map (left) with fragment definition (right) where the triptycene aryl fragments are defined separately. c) Electron-hole heat maps (top) with fragment definitions (bottom) where the triptycene aryl group is included with the trityl moiety (left) or the donor moiety (right).

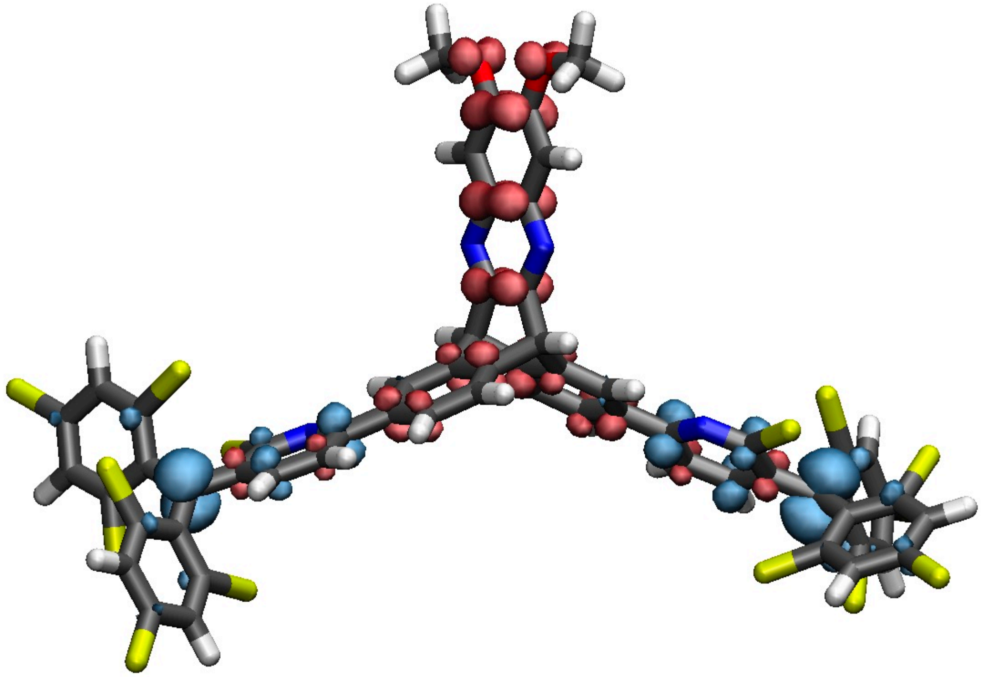

Figure S68: Schematic of orbital contribution to the calculated electron (blue) and hole (red) distribution to the $T_0 \rightarrow T_1$ transition for compound **2,** state 1 (isosurface = 0.002 $e\ a_0^3$).

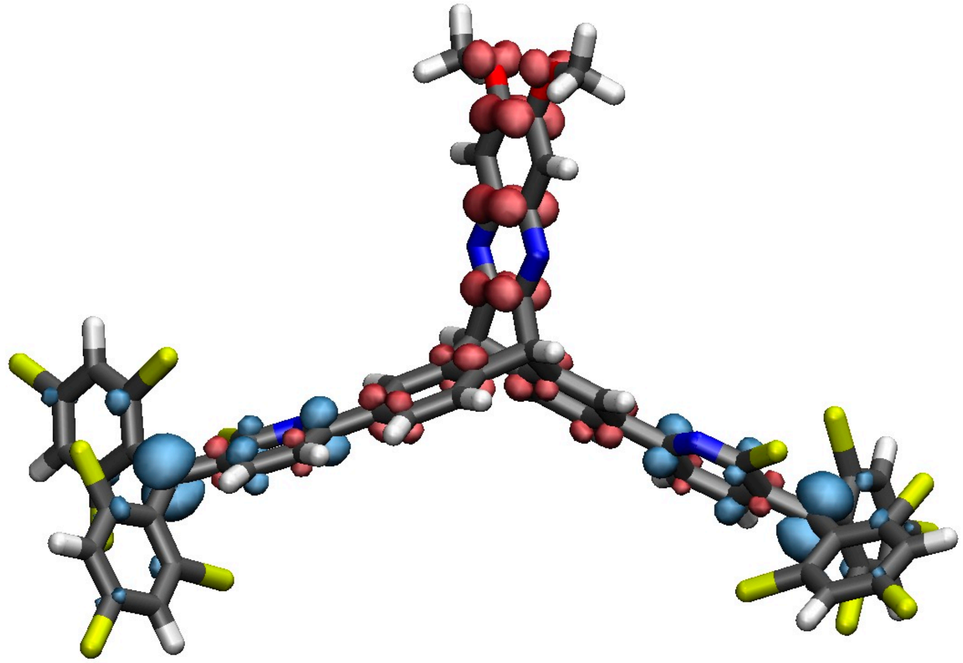

Figure S69: Schematic of orbital contribution to the calculated electron (blue) and hole (red) distribution to the $T_0 \rightarrow T_1$ transition for compound **2,** state 2 (isosurface = 0.002 $e\ a_0^3$).

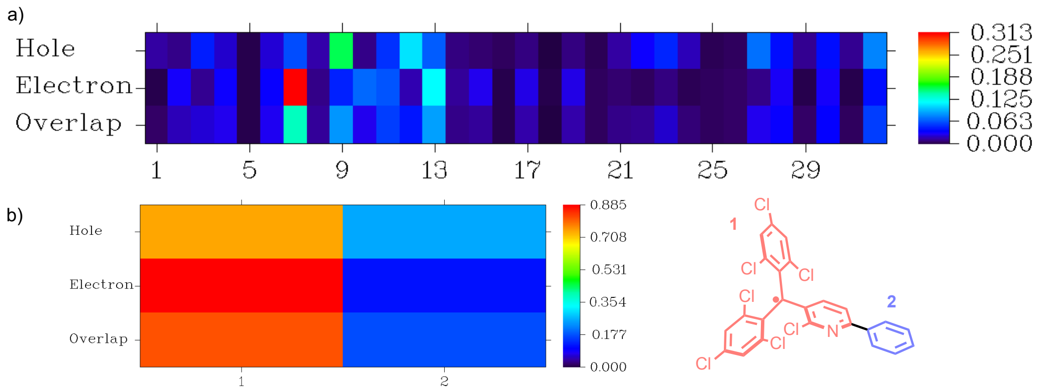

Figure S70: Electron-hole analysis for $D_0 \rightarrow D_1$ for compound **3**. a) Electron-hole heat map for all atoms in compound **1**, hydrogens are omitted. b) Electron-hole heat map (left) with fragment definition (right) where the phenyl fragment is defined separately.

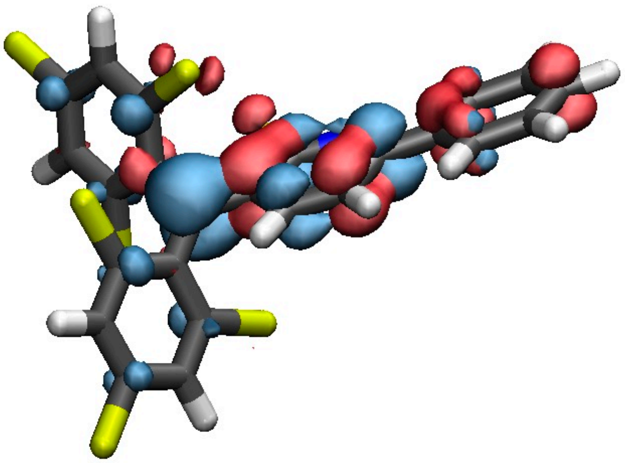

Figure S71: Schematic of orbital contribution to the calculated electron (blue) and hole (red) distribution to the $D_0 \rightarrow D_1$ transition for compound **3** (isosurface = 0.002 $e\ a_0^3$).

Natural Transition Orbitals

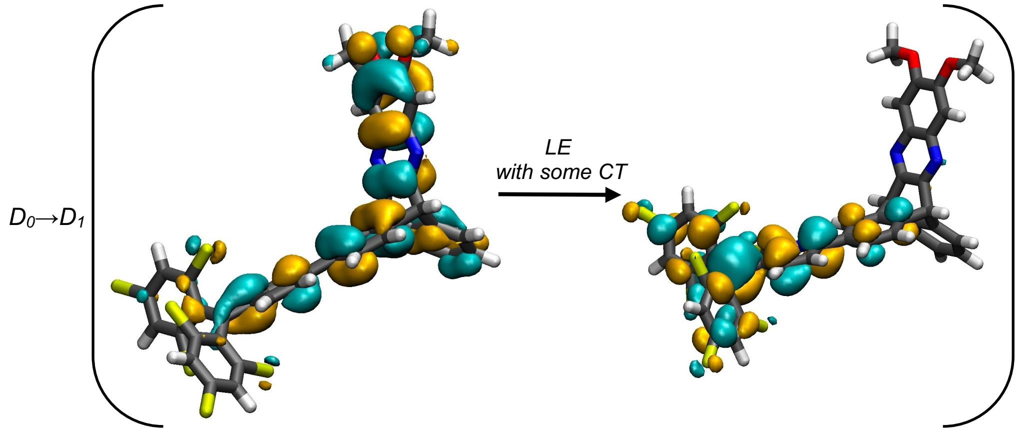

Figure S72: Natural transition orbitals (isosurface = ± 0.03 $e\ a_0^3$) for the lowest energy transition of compound **1** at the UB3LYP-D3(BJ)/def2-SVP/TDDFT, CPCM(toluene) level of theory.

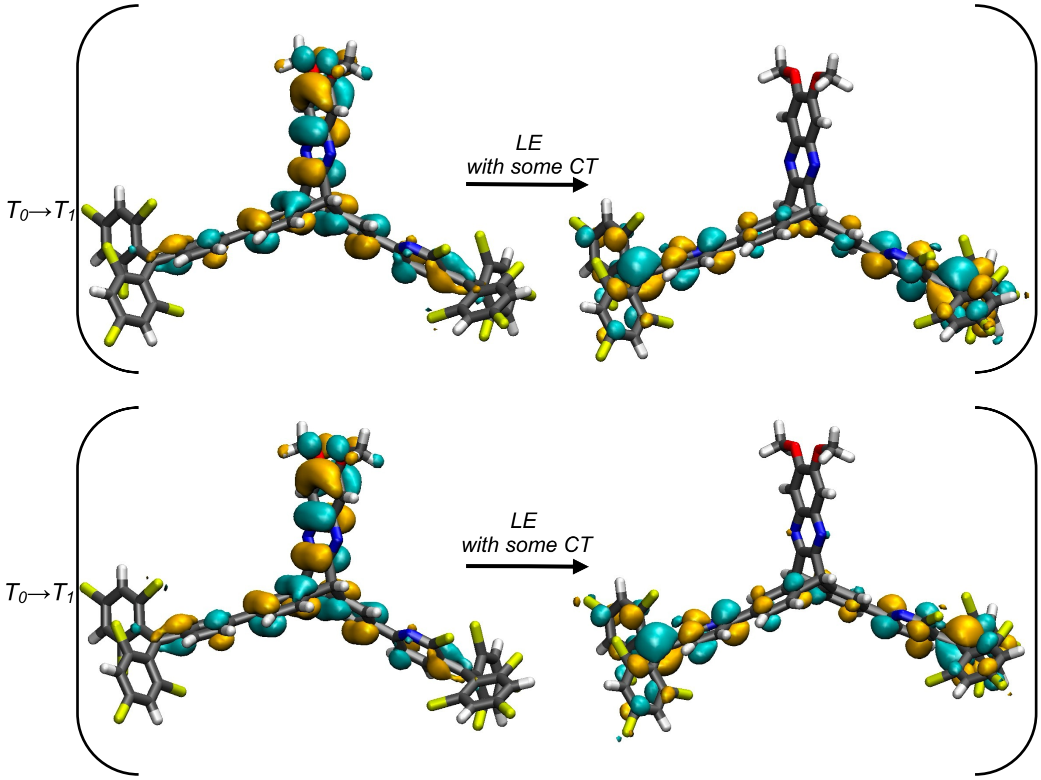

Figure S73: Natural transition orbitals (isosurface = ± 0.03 $e\ a_0^3$) for the lowest energy triplet transition of compound **2** at the UB3LYP-D3(BJ)/def2-SVP/TDDFT, CPCM(toluene) level of theory.

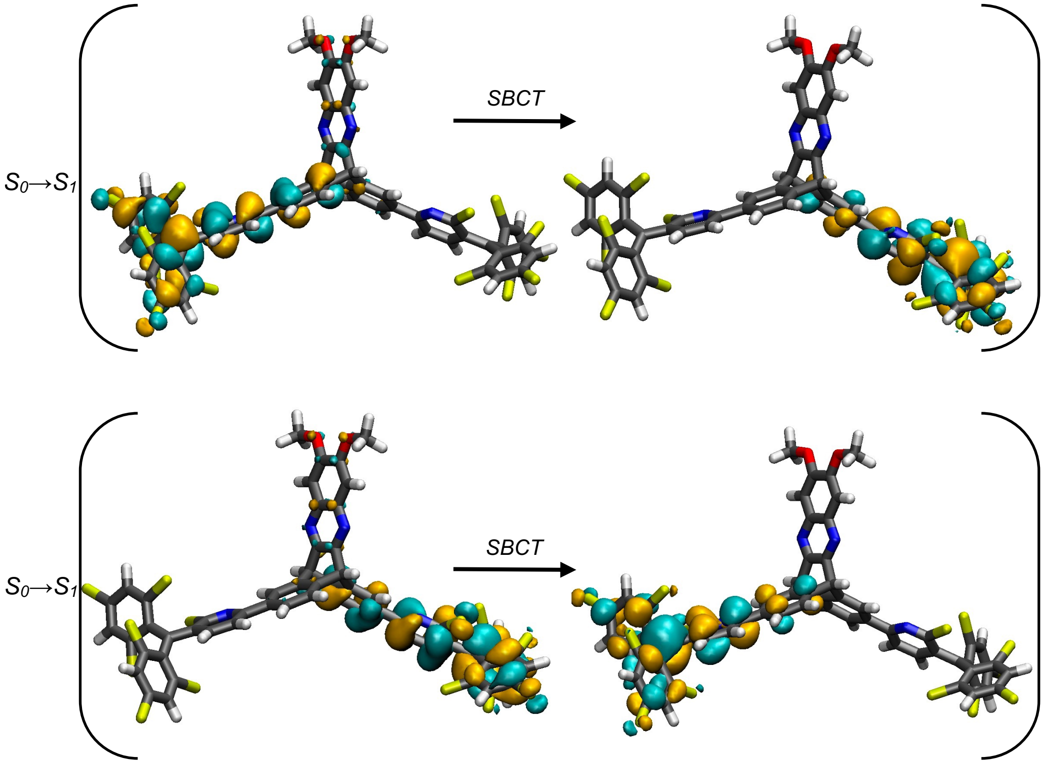

Figure S74: Natural transition orbitals (isosurface = ± 0.03 $e\ a_0^3$) for the lowest energy singlet transition of compound **2** at the UB3LYP-D3(BJ)/def2-SVP/TDDFT, CPCM(toluene) level of theory.

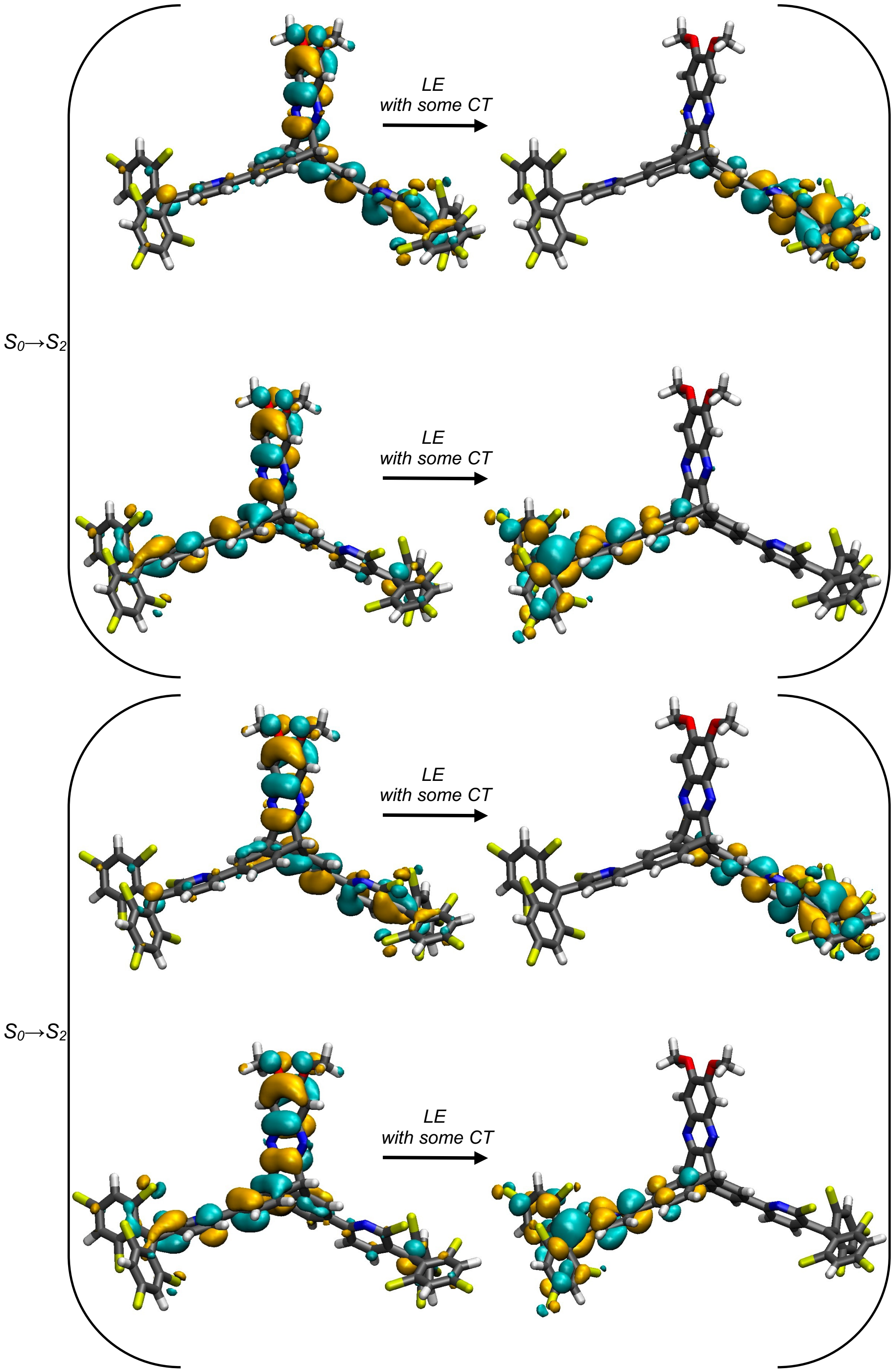

Figure S75: Natural transition orbitals (isosurface = ± 0.03 $e\ a_0^3$) for the second lowest energy singlet transition of compound **2** at the UB3LYP-D3(BJ)/def2-SVP/TDDFT, CPCM(toluene) level of theory.

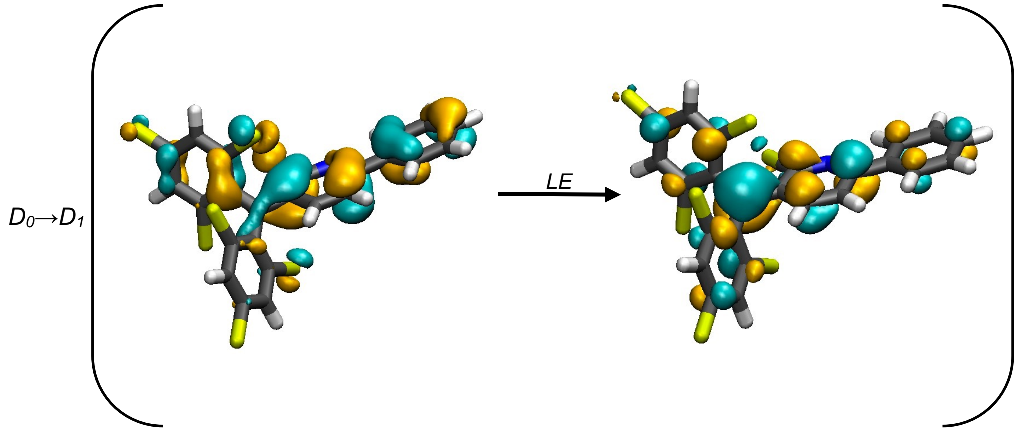

Figure S76: Natural transition orbitals (isosurface = ± 0.03 $e\ a_0^3$) for the lowest energy transition of compound **3** at the UB3LYP-D3(BJ)/def2-SVP/TDDFT, CPCM(toluene) level of theory.

XYZ Coordinates for DFT Optimized Structures at the (BS-)UB3LYP-D3(BJ) / def2-SVP, CPCM(toluene) Level of Theory

Table S11: XYZ coordinates for compound **1**.

Atom	X	Y	Z	Atom	X	Y	Z
**C1**	-5.3570	0.6462	-0.2833	**C40**	6.3961	-4.6574	-2.6137
**C2**	-4.0799	1.0052	0.1447	**C41**	6.4180	-3.4313	-3.5281
**C3**	-2.9946	1.1960	-0.7439	**H42**	4.5014	0.5858	-1.8713
**C4**	-3.2906	1.0027	-2.1172	**H43**	6.4515	-0.6850	-2.6878
**C5**	-4.5546	0.6419	-2.5750	**H44**	1.9739	-2.8771	-2.4111
**C6**	-5.5826	0.4648	-1.6472	**C45**	4.9452	-6.4077	-1.7549
**C7**	-1.6591	1.5890	-0.2744	**C46**	6.0310	-6.9291	-1.0347
**C8**	-1.5295	2.7545	0.6205	**C47**	7.2856	-6.3176	-1.1068
**C9**	-0.4533	0.9261	-0.7106	**C48**	7.4738	-5.1759	-1.9010
**C10**	0.7522	1.6569	-0.8498	**H49**	5.8928	-7.8187	-0.4150
**C11**	-0.3238	-0.4618	-1.0127	**H50**	8.4547	-4.6972	-1.9610
**C12**	1.9184	1.0542	-1.2866	**C51**	4.6335	-4.5543	-4.8036
**C13**	1.9089	-0.3214	-1.5937	**H52**	3.0849	-5.0538	-3.3474
**N14**	0.7749	-1.0311	-1.4294	**C53**	5.9199	-3.9271	-4.8812
**C15**	-2.0917	4.0156	0.3124	**H54**	7.4023	-2.9561	-3.6037
**C16**	-1.9957	5.1166	1.1641	**N55**	4.0298	-5.0558	-5.8500
**C17**	-1.3098	4.9747	2.3698	**N56**	6.5807	-3.8071	-6.0036
**C18**	-0.7261	3.7579	2.7275	**C57**	6.0498	-4.7507	-9.5140
**C19**	-0.8418	2.6760	1.8582	**C58**	4.7561	-5.3871	-9.4348
**H20**	-6.1561	0.4942	0.4413	**O59**	6.5924	-4.7182	-10.7473
**Cl21**	-3.8419	1.1367	1.8710	**C60**	7.8604	-4.1113	-10.9109
**Cl22**	-7.1718	0.0122	-2.2016	**H61**	7.8363	-3.0454	-10.6246
**H23**	-4.7369	0.5158	-3.6414	**H62**	8.1111	-4.1964	-11.9762
**Cl24**	-2.0684	1.2848	-3.3333	**H63**	8.6325	-4.6240	-10.3111
**H25**	0.7418	2.7271	-0.6358	**O64**	4.2812	-5.8565	-10.6053
**Cl26**	-1.6745	-1.5583	-0.7434	**C65**	3.0147	-6.4884	-10.6151
**Cl27**	-0.1736	1.1553	2.3958	**H66**	3.0068	-7.3857	-9.9719
**Cl28**	-2.8975	4.2701	-1.2175	**H67**	2.8228	-6.7846	-11.6544
**H29**	-2.4364	6.0713	0.8797	**H68**	2.2196	-5.8008	-10.2776
**Cl30**	-1.1790	6.3372	3.4490	**H69**	3.9632	-6.8849	-1.7022
**H31**	-0.2048	3.6460	3.6775	**C70**	6.6342	-4.2338	-8.3735
**C32**	3.0936	-1.0558	-2.0873	**C71**	4.1056	-5.4767	-8.2192
**C33**	2.9558	-2.4074	-2.4781	**C72**	4.6950	-4.9507	-7.0397
**C34**	4.0574	-3.1146	-2.9337	**C73**	5.9776	-4.3217	-7.1176
**C35**	4.3621	-0.4529	-2.1737	**H74**	7.6083	-3.7481	-8.3951
**C36**	5.4731	-1.1688	-2.6331	**H75**	3.1298	-5.9497	-8.1215
**C37**	5.3245	-2.5000	-3.0117	**H76**	8.1263	-6.7299	-0.5430
**C38**	4.0687	-4.5735	-3.3884	**H77**	2.8209	1.6522	-1.4058
**C39**	5.1307	-5.2731	-2.5398	**C39**	5.1307	-5.2731	-2.5398

Table S12: XYZ coordinates for the OSS state of compound **2**.

Atom	X	Y	Z	Atom	X	Y	Z
**C1**	-5.2672	0.8414	-0.0125	**N55**	3.7224	-4.9884	-5.7194
**C2**	-4.0213	0.9578	0.6021	**N56**	6.3971	-4.0352	-5.5600
**C3**	-2.8187	1.1385	-0.1220	**C57**	5.9381	-4.2203	-9.1980
**C4**	-2.9600	1.2021	-1.5315	**C58**	4.5805	-4.7060	-9.2800
**C5**	-4.1893	1.0869	-2.1742	**O59**	6.5407	-4.0134	-10.3852
**C6**	-5.3387	0.9041	-1.4035	**C60**	7.8707	-3.5288	-10.3920
**C7**	-1.5157	1.2669	0.5457	**H61**	7.9417	-2.5433	-9.8998
**C8**	-1.3631	2.2473	1.6369	**H62**	8.1608	-3.4313	-11.4461
**C9**	-0.3544	0.5209	0.1237	**H63**	8.5571	-4.2304	-9.8868
**C10**	-0.3617	-0.8038	-0.4046	**O64**	4.1145	-4.8786	-10.5326
**C11**	0.9390	1.0891	0.2260	**C65**	2.7881	-5.3432	-10.7013
**N12**	0.6971	-1.4399	-0.8271	**H66**	2.6512	-6.3425	-10.2523
**C13**	1.9182	-0.8720	-0.7753	**H67**	2.6150	-5.4038	-11.7835
**C14**	2.0631	0.4137	-0.2164	**H68**	2.0582	-4.6474	-10.2523
**C15**	-1.7342	3.6051	1.4948	**C69**	7.6656	-8.3140	-0.5980
**C16**	-1.6226	4.5338	2.5298	**N70**	8.9339	-7.8793	-0.7361
**C17**	-1.1173	4.1114	3.7589	**C71**	9.9338	-8.5368	-0.2126
**C18**	-0.7240	2.7868	3.9590	**C72**	9.8054	-9.7369	0.5470
**C19**	-0.8504	1.8821	2.9075	**C73**	8.4709	-10.1958	0.6718
**H20**	-6.1628	0.6860	0.5877	**C74**	7.4110	-9.5019	0.1160
**Cl21**	-3.9879	0.7869	2.3409	**Cl75**	11.5268	-7.8881	-0.5865
**Cl22**	-6.8858	0.7538	-2.1921	**H76**	8.2831	-11.1050	1.2454
**H23**	-4.2494	1.1527	-3.2598	**C77**	10.8893	-10.4896	1.1328
**Cl24**	-1.5714	1.5031	-2.5480	**C78**	10.7348	-11.9521	1.2494
**Cl25**	-1.8539	-1.7373	-0.4378	**C79**	10.4470	-12.7673	0.1250
**H26**	1.0364	2.0983	0.6302	**C80**	10.3033	-14.1499	0.2114
**Cl27**	-0.4315	0.2200	3.2394	**C81**	10.4512	-14.7660	1.4555
**Cl28**	-2.3048	4.2044	-0.0443	**C82**	10.7356	-14.0193	2.5982
**H29**	-1.9128	5.5716	2.3704	**C83**	10.8694	-12.6355	2.4809
**Cl30**	-0.9759	5.2545	5.0669	**C84**	12.0977	-9.8542	1.6751
**H31**	-0.3437	2.4569	4.9251	**C85**	13.3977	-10.3108	1.3517
**C32**	3.0449	-1.6572	-1.3243	**C86**	12.0422	-8.7594	2.5741
**C33**	2.7801	-2.8957	-1.9518	**C87**	13.1797	-8.1613	3.1085
**C34**	3.8200	-3.6385	-2.4885	**C88**	14.5555	-9.7340	1.8713
**C35**	4.3759	-1.2064	-1.2526	**C89**	14.4341	-8.6574	2.7490
**C36**	5.4246	-1.9592	-1.7924	**Cl90**	13.6159	-11.6019	0.1946
**C37**	5.1497	-3.1752	-2.4119	**H91**	13.0867	-7.3299	3.8061
**C38**	3.6906	-4.9803	-3.2095	**Cl92**	15.8698	-7.9221	3.4096
**C39**	4.6248	-5.9424	-2.4807	**H93**	15.5351	-10.1110	1.5800
**C40**	5.9557	-5.4818	-2.4057	**Cl94**	10.3454	-12.0614	-1.4686
**C41**	6.1582	-4.1171	-3.0645	**H95**	10.0988	-14.7379	-0.6826
**H42**	4.6123	-0.2580	-0.7685	**Cl96**	10.2780	-16.4956	1.5814
**H43**	6.4520	-1.5929	-1.7276	**H97**	10.8359	-14.5002	3.5704
**H44**	1.7495	-3.2474	-2.0071	**Cl98**	11.1514	-11.7440	3.9571

**C45**	4.2853	-7.1741	-1.9280	**H99**	3.2570	-7.5393	-1.9843
**C46**	5.2715	-7.9460	-1.3043	**Cl100**	10.5014	-8.1423	3.1186
**C47**	6.6047	-7.5016	-1.2313	**C101**	6.5188	-4.0032	-7.9634
**C48**	6.9362	-6.2492	-1.7963	**C102**	3.8661	-4.9544	-8.1235
**H49**	4.9891	-8.9131	-0.8865	**C103**	4.4512	-4.7365	-6.8483
**H50**	7.9701	-5.9062	-1.7462	**C104**	5.7963	-4.2547	-6.7677
**C51**	4.3254	-4.7660	-4.5800	**H105**	7.5393	-3.6376	-7.8629
**H52**	2.6587	-5.3399	-3.2865	**H106**	2.8410	-5.3203	-8.1470
**C53**	5.6741	-4.2889	-4.5000	**H107**	6.3951	-9.8711	0.2510
**H54**	7.1918	-3.7570	-3.0212	**H108**	3.0393	0.8924	-0.1496

Table S13: XYZ coordinates for the triplet state of compound **2**.

Atom	X	Y	Z	Atom	X	Y	Z
**C1**	-5.2659	0.8497	-0.0300	**N55**	3.7194	-4.9894	-5.7133
**C2**	-4.0227	0.9636	0.5901	**N56**	6.3941	-4.0363	-5.5554
**C3**	-2.8162	1.1422	-0.1284	**C57**	5.9334	-4.2223	-9.1932
**C4**	-2.9512	1.2054	-1.5386	**C58**	4.5757	-4.7079	-9.2745
**C5**	-4.1778	1.0928	-2.1868	**O59**	6.5355	-4.0161	-10.3807
**C6**	-5.3311	0.9127	-1.4214	**C60**	7.8657	-3.5321	-10.3884
**C7**	-1.5161	1.2691	0.5449	**H61**	7.9374	-2.5464	-9.8966
**C8**	-1.3660	2.2514	1.6347	**H62**	8.1553	-3.4351	-11.4427
**C9**	-0.3541	0.5214	0.1277	**H63**	8.5521	-4.2337	-9.8832
**C10**	-0.3617	-0.8044	-0.3977	**O64**	4.1092	-4.8808	-10.5268
**C11**	0.9393	1.0890	0.2306	**C65**	2.7830	-5.3462	-10.6948
**N12**	0.6969	-1.4418	-0.8184	**H66**	2.6466	-6.3452	-10.2451
**C13**	1.9183	-0.8741	-0.7669	**H67**	2.6096	-5.4076	-11.7770
**C14**	2.0634	0.4124	-0.2099	**H68**	2.0528	-4.6504	-10.2461
**C15**	-1.7343	3.6095	1.4880	**C69**	7.6664	-8.3162	-0.5962
**C16**	-1.6267	4.5400	2.5218	**N70**	8.9343	-7.8803	-0.7342
**C17**	-1.1283	4.1192	3.7542	**C71**	9.9350	-8.5376	-0.2124
**C18**	-0.7375	2.7945	3.9588	**C72**	9.8081	-9.7386	0.5462
**C19**	-0.8597	1.8879	2.9083	**C73**	8.4740	-10.1990	0.6706
**H20**	-6.1646	0.6959	0.5660	**C74**	7.4133	-9.5055	0.1161
**Cl21**	-3.9976	0.7907	2.3290	**Cl75**	11.5273	-7.8871	-0.5864
**Cl22**	-6.8750	0.7661	-2.2169	**H76**	8.2873	-11.1092	1.2431
**H23**	-4.2329	1.1588	-3.2727	**C77**	10.8928	-10.4907	1.1306
**Cl24**	-1.5577	1.5045	-2.5491	**C78**	10.7391	-11.9534	1.2470
**Cl25**	-1.8552	-1.7358	-0.4314	**C79**	10.4490	-12.7681	0.1229
**H26**	1.0368	2.0990	0.6329	**C80**	10.3057	-14.1508	0.2088
**Cl27**	-0.4456	0.2258	3.2456	**C81**	10.4560	-14.7674	1.4524
**Cl28**	-2.2958	4.2063	-0.0554	**C82**	10.7423	-14.0212	2.5949
**H29**	-1.9146	5.5779	2.3589	**C83**	10.8758	-12.6374	2.4780
**Cl30**	-0.9927	5.2643	5.0610	**C84**	12.1023	-9.8551	1.6707
**H31**	-0.3627	2.4659	4.9274	**C85**	13.4019	-10.3092	1.3421
**C32**	3.0448	-1.6597	-1.3161	**C86**	12.0483	-8.7624	2.5723
**C33**	2.7795	-2.8976	-1.9445	**C87**	13.1867	-8.1643	3.1048

**C34**	3.8190	-3.6403	-2.4820	**C88**	14.5606	-9.7321	1.8594
**C35**	4.3760	-1.2095	-1.2439	**C89**	14.4406	-8.6578	2.7401
**C36**	5.4242	-1.9620	-1.7849	**Cl90**	13.6185	-11.5966	0.1805
**C37**	5.1488	-3.1772	-2.4058	**H91**	13.0948	-7.3348	3.8048
**C38**	3.6891	-4.9818	-3.2035	**Cl92**	15.8774	-7.9223	3.3979
**C39**	4.6238	-5.9441	-2.4754	**H93**	15.5398	-10.1071	1.5640
**C40**	5.9548	-5.4836	-2.4014	**Cl94**	10.3438	-12.0612	-1.4701
**C41**	6.1568	-4.1187	-3.0598	**H95**	10.0994	-14.7383	-0.6850
**H42**	4.6128	-0.2618	-0.7586	**Cl96**	10.2830	-16.4971	1.5778
**H43**	6.4518	-1.5961	-1.7201	**H97**	10.8443	-14.5025	3.5668
**H44**	1.7488	-3.2490	-1.9996	**Cl98**	11.1598	-11.7465	3.9543
**C45**	4.2846	-7.1756	-1.9223	**H99**	3.2562	-7.5408	-1.9778
**C46**	5.2713	-7.9477	-1.2995	**Cl100**	10.5086	-8.1493	3.1244
**C47**	6.6046	-7.5036	-1.2279	**C101**	6.5147	-4.0048	-7.9590
**C48**	6.9357	-6.2511	-1.7928	**C102**	3.8618	-4.9558	-8.1176
**H49**	4.9890	-8.9149	-0.8816	**C103**	4.4475	-4.7376	-6.8426
**H50**	7.9696	-5.9082	-1.7437	**C104**	5.7927	-4.2559	-6.7628
**C51**	4.3230	-4.7671	-4.5743	**H105**	7.5353	-3.6394	-7.8590
**H52**	2.6572	-5.3414	-3.2799	**H106**	2.8366	-5.3217	-8.1404
**C53**	5.6718	-4.2900	-4.4950	**H107**	6.3979	-9.8761	0.2506
**H54**	7.1905	-3.7587	-3.0170	**H108**	3.0397	0.8909	-0.1437

Table S14: XYZ coordinates for compound **3**.

Atom	X	Y	Z	Atom	X	Y	Z
**C1**	-5.3437	0.6635	-0.3527	**H23**	-4.6664	0.5664	-3.7009
**C2**	-4.0726	1.0119	0.1011	**Cl24**	-2.0010	1.3217	-3.3393
**C3**	-2.9714	1.2068	-0.7668	**H25**	0.7745	2.7068	-0.6027
**C4**	-3.2442	1.0297	-2.1469	**Cl26**	-1.6714	-1.5582	-0.7253
**C5**	-4.5018	0.6798	-2.6301	**Cl27**	-0.1841	1.1068	2.4063
**C6**	-5.5463	0.4974	-1.7222	**Cl28**	-2.8671	4.2840	-1.1835
**C7**	-1.6417	1.5859	-0.2704	**H29**	-2.4228	6.0504	0.9464
**C8**	-1.5170	2.7372	0.6420	**Cl30**	-1.1909	6.2733	3.5314
**C9**	-0.4359	0.9175	-0.7007	**H31**	-0.2217	3.5773	3.7261
**C10**	0.7745	1.6397	-0.8324	**C32**	3.0868	-1.0744	-2.1248
**C11**	-0.3159	-0.4704	-1.0052	**C33**	3.0041	-2.4668	-2.3243
**C12**	1.9345	1.0313	-1.2800	**C34**	4.0969	-3.1845	-2.8081
**C13**	1.9112	-0.3392	-1.6060	**C35**	4.2983	-0.4244	-2.4314
**N14**	0.7762	-1.0444	-1.4322	**C36**	5.3906	-1.1446	-2.9165
**C15**	-2.0749	4.0040	0.3488	**C37**	5.2962	-2.5274	-3.1059
**C16**	-1.9857	5.0907	1.2192	**H38**	4.3971	0.6541	-2.3005
**C17**	-1.3117	4.9286	2.4291	**H39**	6.3212	-0.6215	-3.1505
**C18**	-0.7329	3.7054	2.7728	**H40**	2.0654	-2.9691	-2.0888
**C19**	-0.8417	2.6378	1.8851	**H41**	2.8455	1.6196	-1.3818
**H20**	-6.1559	0.5076	0.3562	**H42**	4.0134	-4.2645	-2.9536
**Cl21**	-3.8629	1.1243	1.8323	**H43**	6.1531	-3.0898	-3.4853
**Cl22**	-7.1272	0.0574	-2.3093				

NUCLEAR MAGNETIC RESONANCE SPECTRA

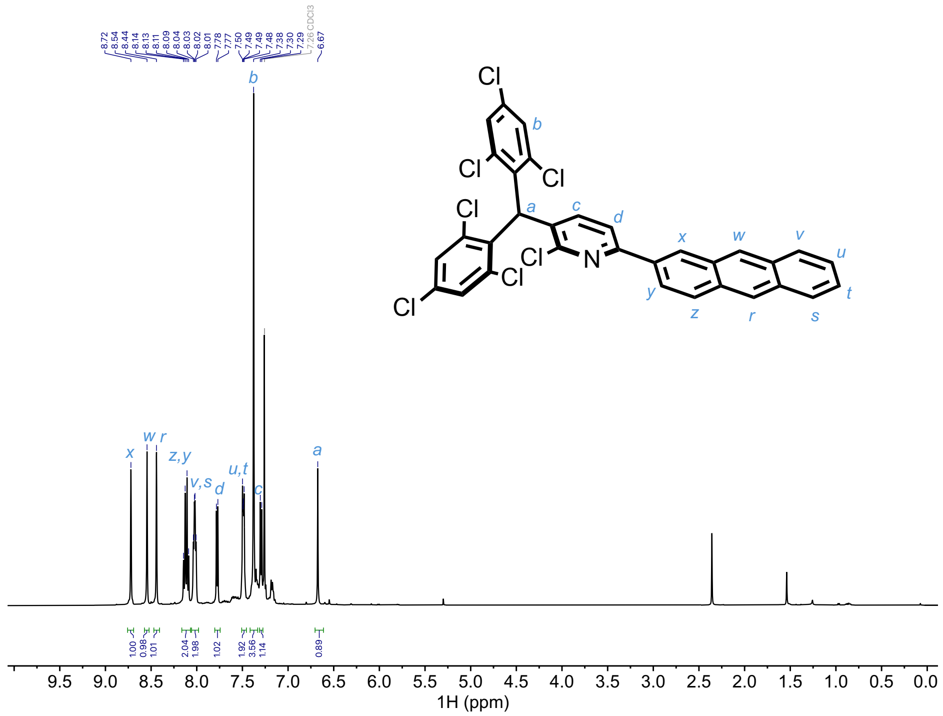

Figure S77: ^{1}H NMR (500 MHz, $CDCl_3$) of 6-(anthracen-2-yl)-3-(bis(2,4,6-trichlorophenyl)methyl)-2-chloropyridine.

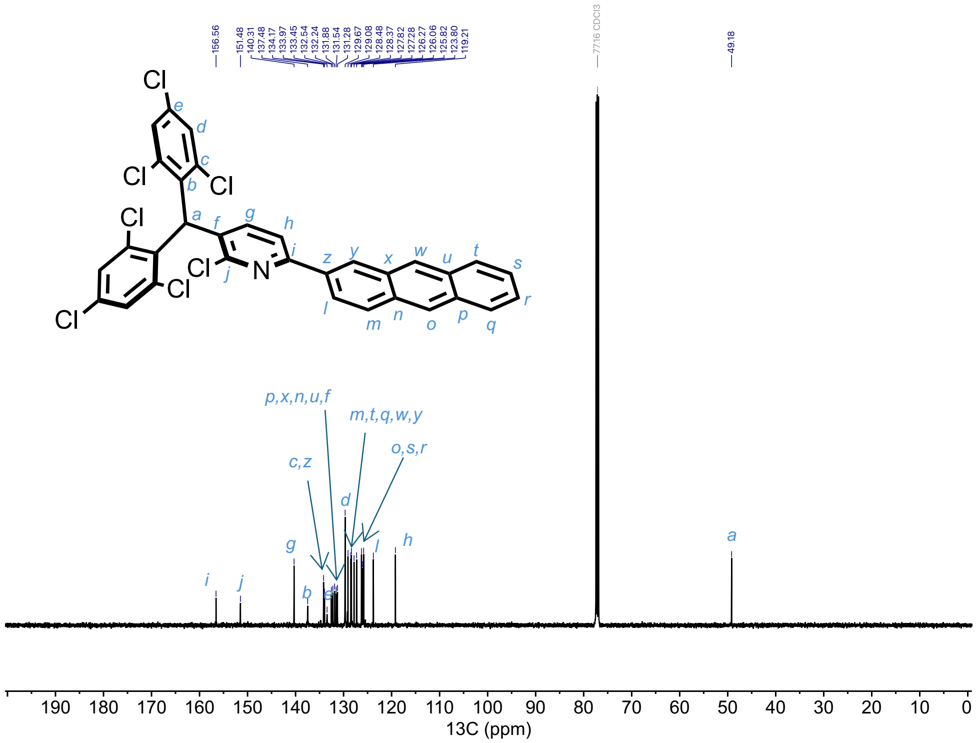

Figure S78: $^{13}C\{^{1}H\}$ NMR (126 MHz, $CDCl_3$) of 6-(anthracen-2-yl)-3-(bis(2,4,6-trichlorophenyl)methyl)-2-chloropyridine.

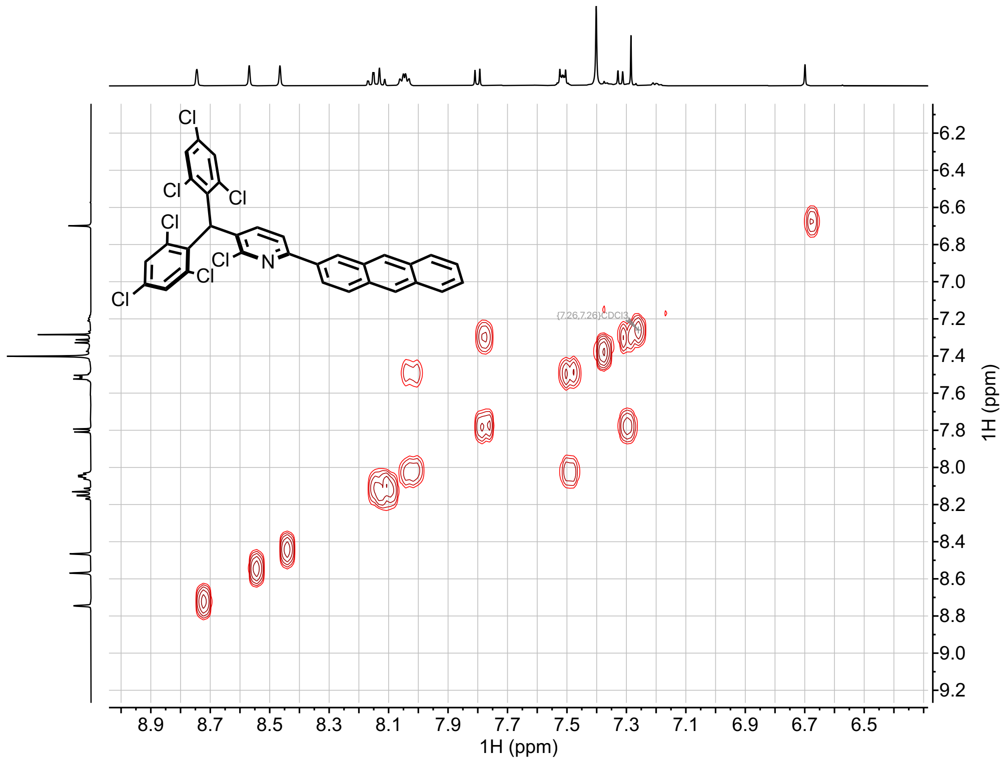

Figure S79: ^{1}H-^{1}H COSY of 6-(anthracen-2-yl)-3-(bis(2,4,6-trichlorophenyl)methyl)-2-chloropyridine in $CDCl_3$.

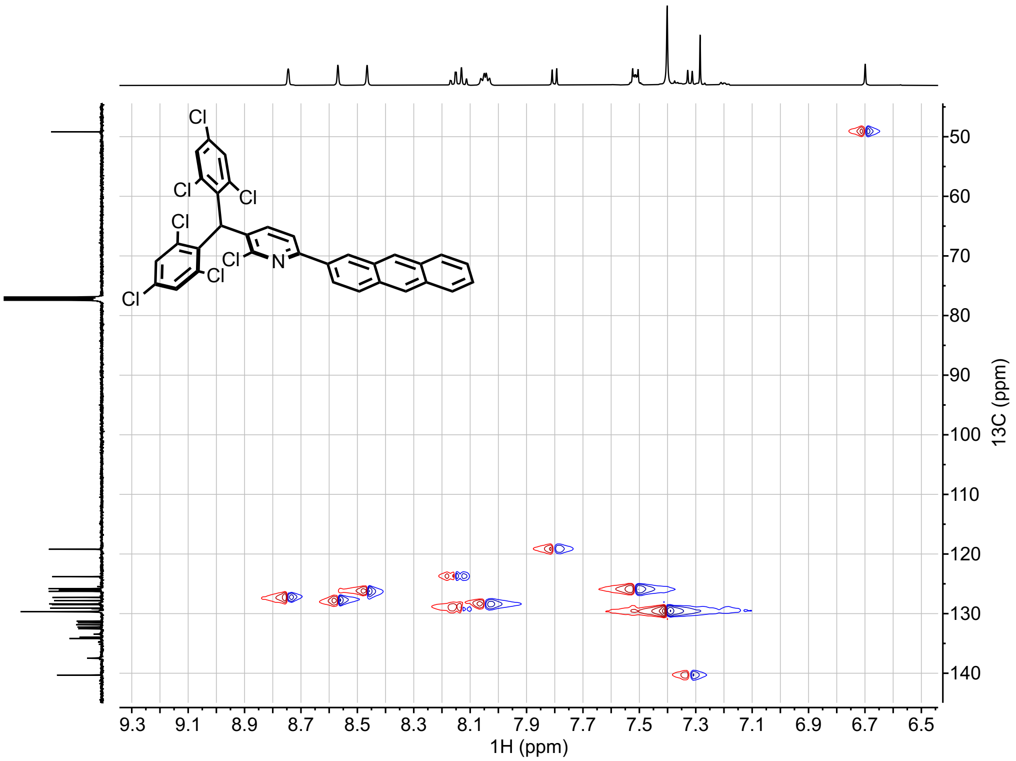

Figure S80: ^{1}H-^{13}C HSQC of 6-(anthracen-2-yl)-3-(bis(2,4,6-trichlorophenyl)methyl)-2-chloropyridine in $CDCl_3$.

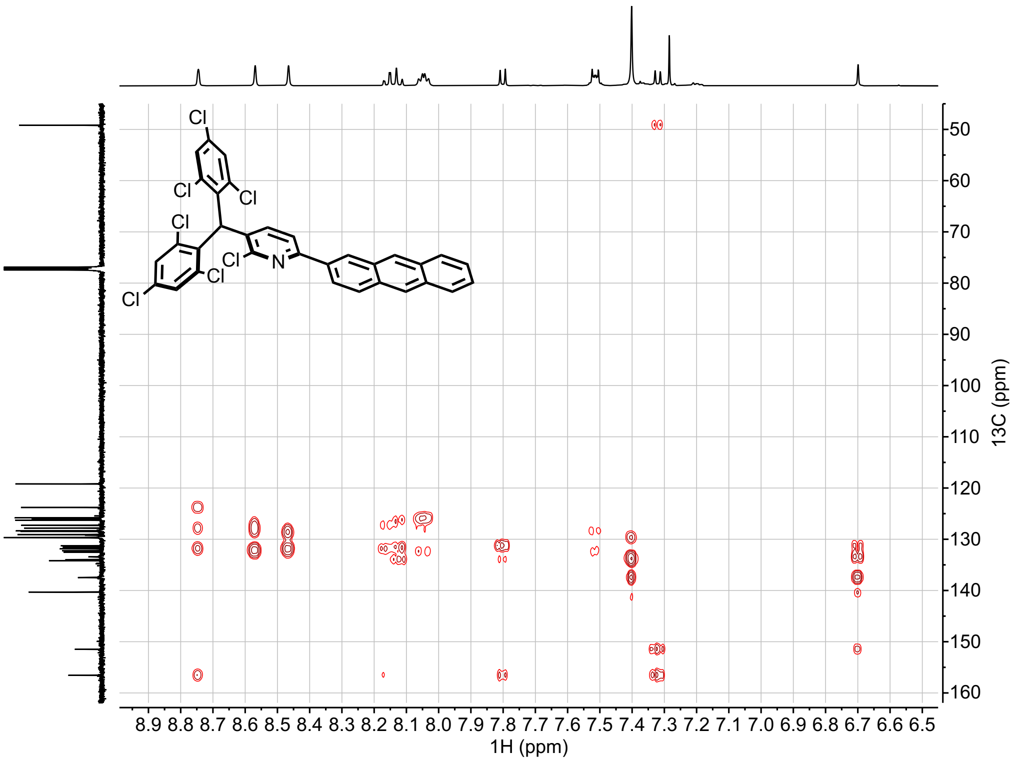

Figure S81: ^{1}H-^{13}C HMBC of 6-(anthracen-2-yl)-3-(bis(2,4,6-trichlorophenyl)methyl)-2-chloropyridine in $CDCl_3$.

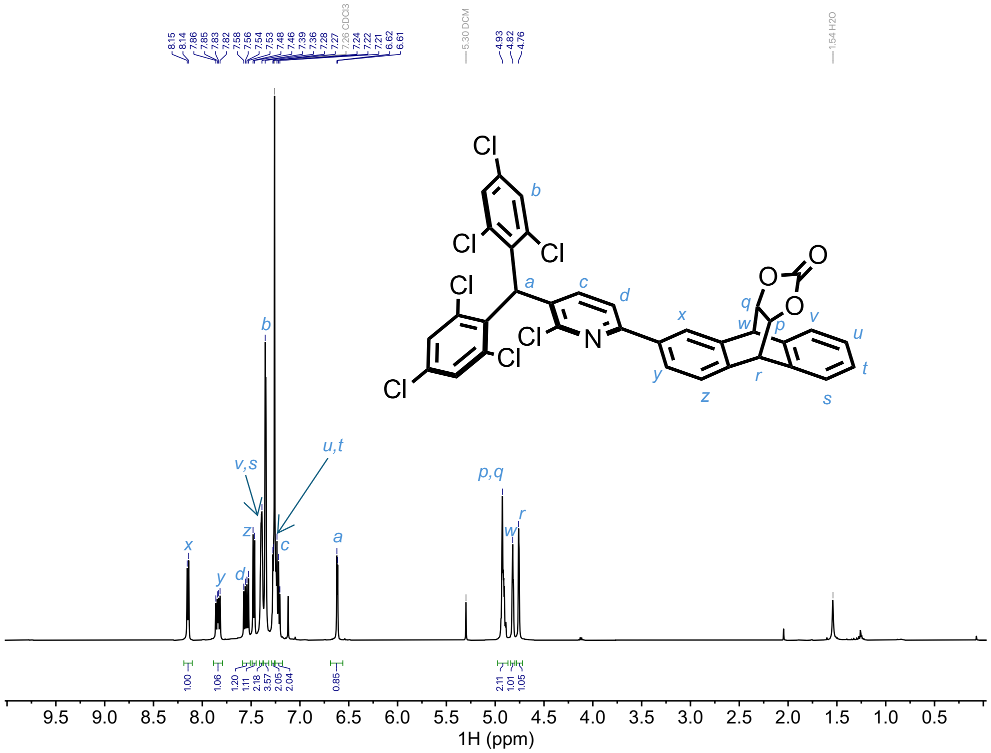

Figure S82: ^{1}H NMR (500 MHz, $CDCl_3$) of 2-(5-(bis(2,4,6-trichlorophenyl)methyl)-6-chloropyridin-2-yl)-9,10-dihydro-9,10-[4,5]epidioxoloanthracen-13-one

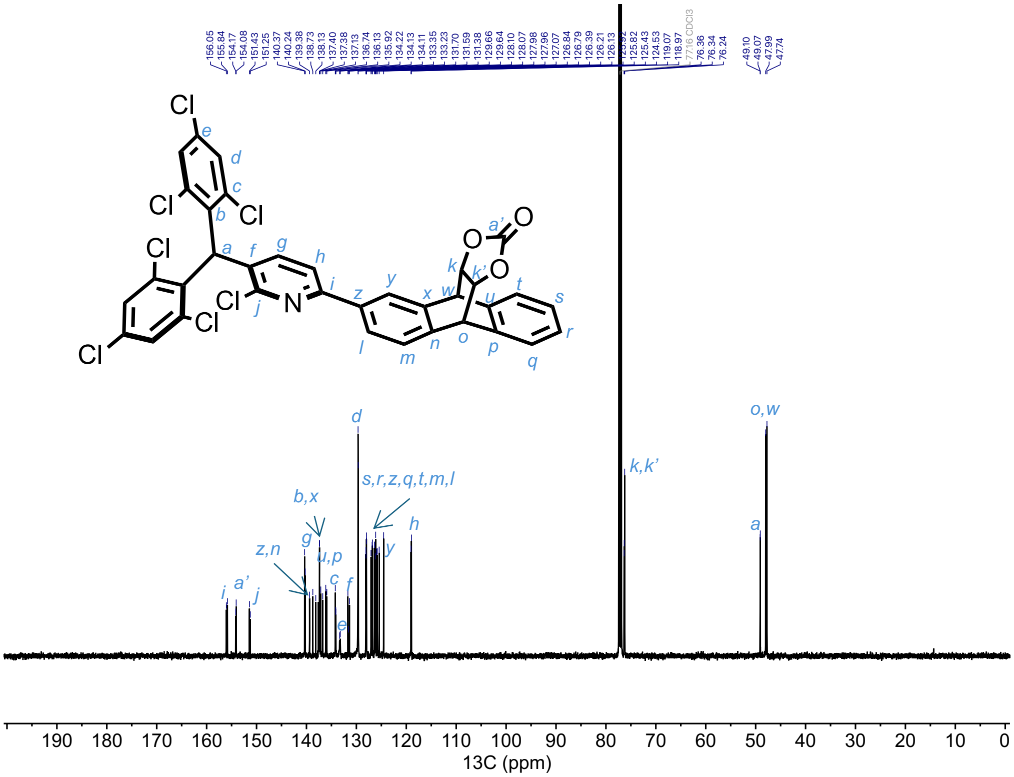

Figure S83: $^{13}C\{^1H\}$ NMR (126 MHz, $CDCl_3$) of 2-(5-(bis(2,4,6-trichlorophenyl)methyl)-6-chloropyridin-2-yl)-9,10-dihydro-9,10-[4,5]epidioxoloanthracen-13-one.

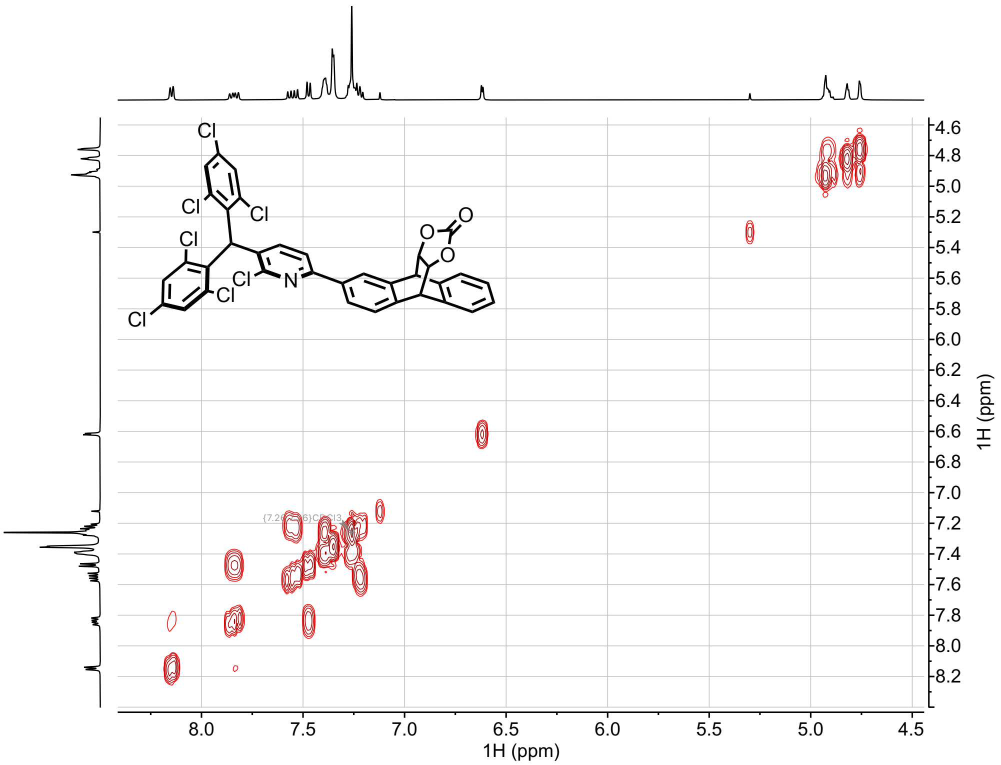

Figure S84: ^1H-^1H COSY of 2-(5-(bis(2,4,6-trichlorophenyl)methyl)-6-chloropyridin-2-yl)-9,10-dihydro-9,10-[4,5]epidioxoloanthracen-13-onein $CDCl_3$.

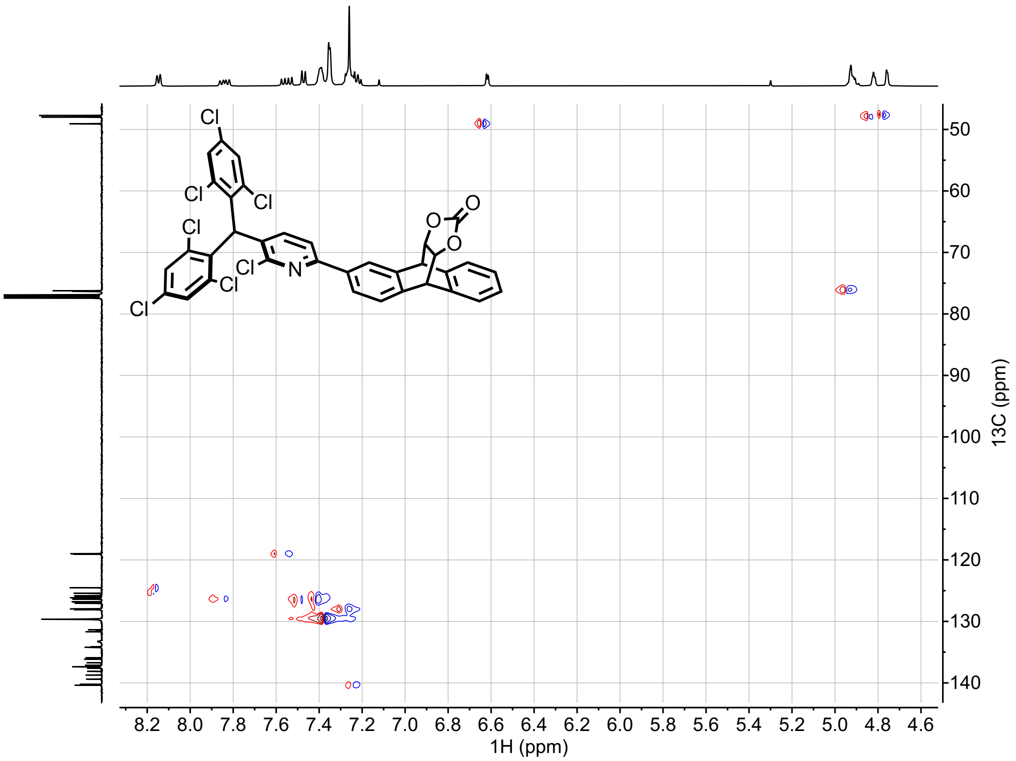

Figure S85: ^{1}H-^{13}C HSQC of 2-(5-(bis(2,4,6-trichlorophenyl)methyl)-6-chloropyridin-2-yl)-9,10-dihydro-9,10-[4,5]epidioxoloanthracen-13-one in $CDCl_3$.

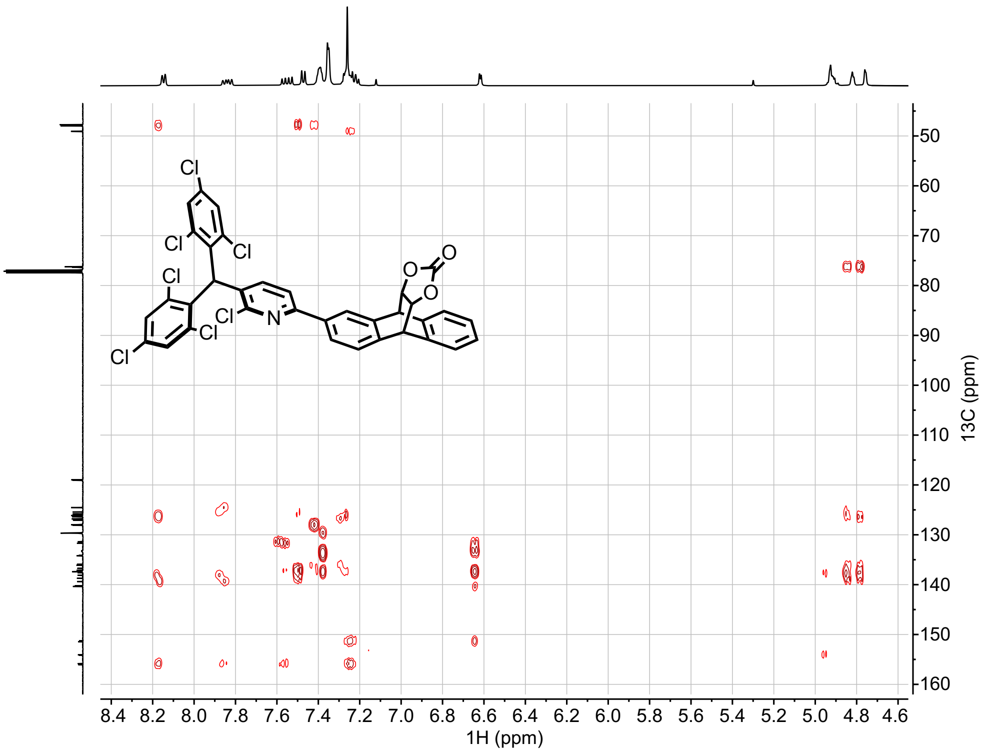

Figure S86: ^{1}H-^{13}C HMBC of 2-(5-(bis(2,4,6-trichlorophenyl)methyl)-6-chloropyridin-2-yl)-9,10-dihydro-9,10-[4,5]epidioxoloanthracen-13-one in $CDCl_3$.

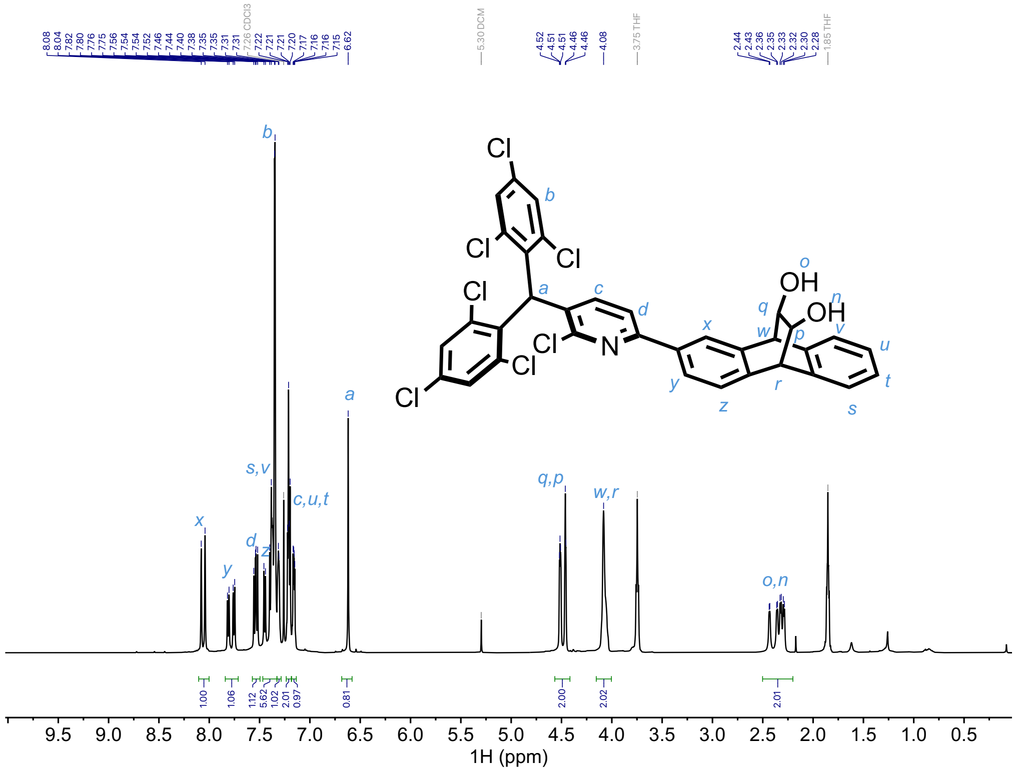

Figure S87: ^{1}H NMR (500 MHz, $CDCl_3$) of 2-(5-(bis(2,4,6-trichlorophenyl)methyl)-6-chloropyridin-2-yl)-9,10-dihydro-9,10-ethanoanthracene-11,12-diol.

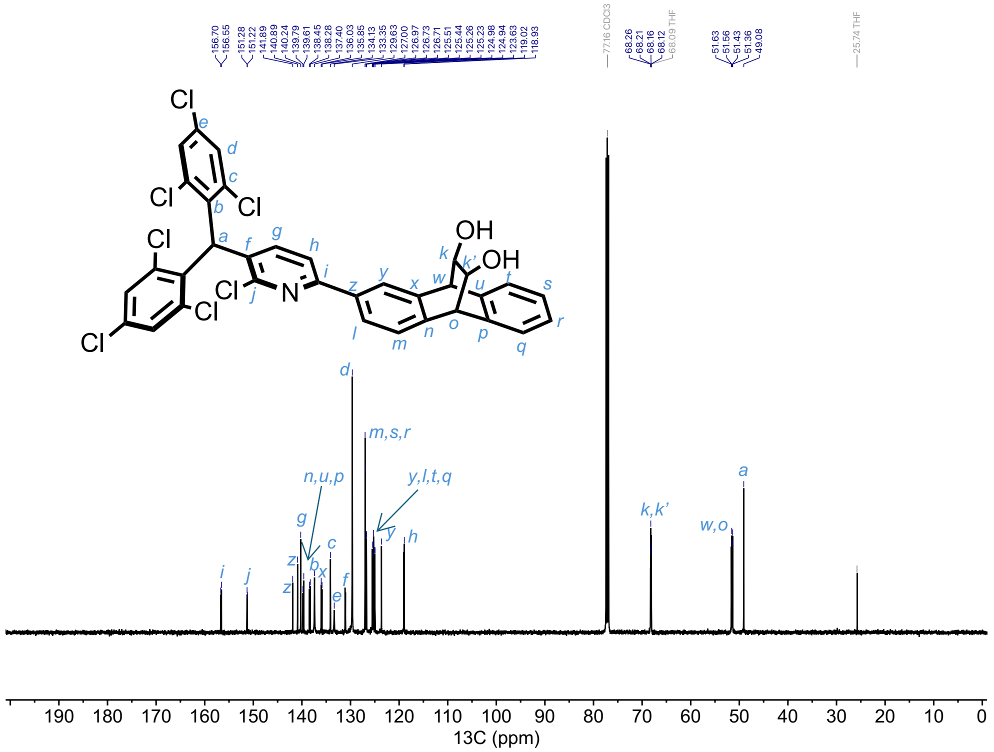

Figure S88: $^{13}C\{^{1}H\}$ NMR (126 MHz, $CDCl_3$) of 2-(5-(bis(2,4,6-trichlorophenyl)methyl)-6-chloropyridin-2-yl)-9,10-dihydro-9,10-ethanoanthracene-11,12-diol.

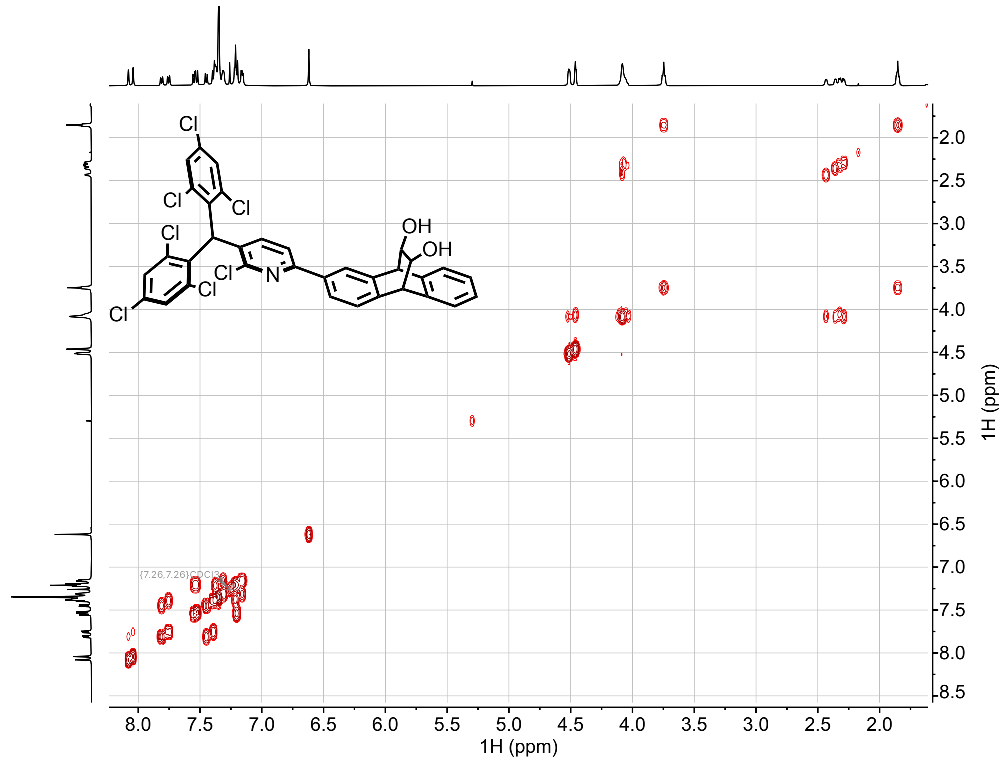

Figure S89: ^{1}H-^{1}H COSY of 2-(5-(bis(2,4,6-trichlorophenyl)methyl)-6-chloropyridin-2-yl)-9,10-dihydro-9,10-ethanoanthracene-11,12-diol in $CDCl_3$.

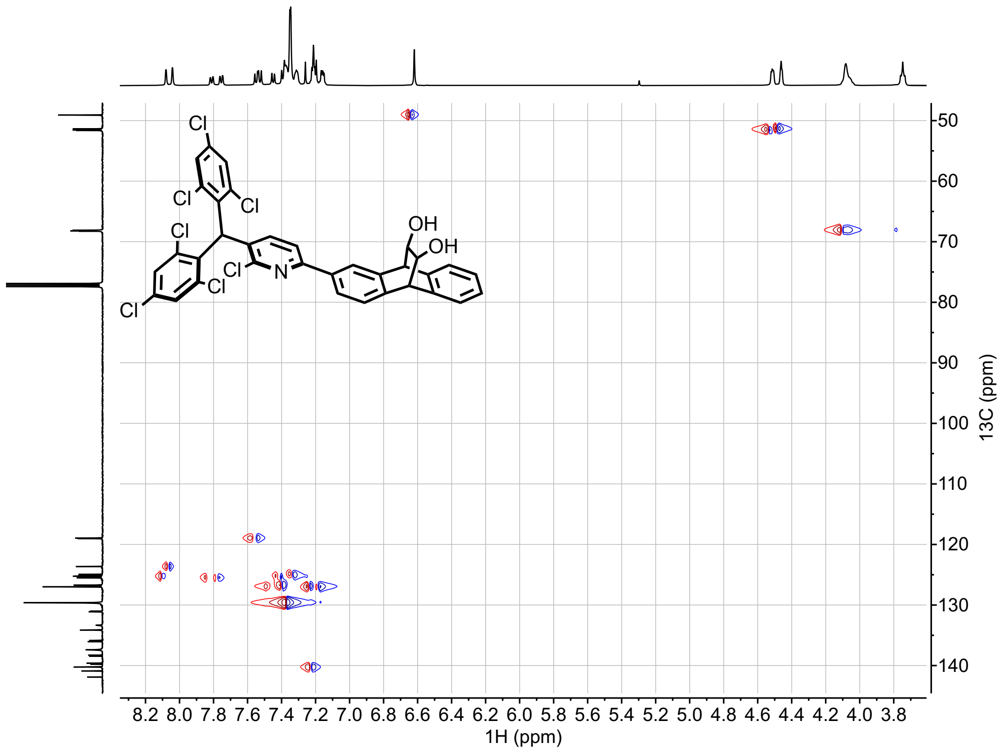

Figure S90: ^{1}H-^{13}C HSQC of 2-(5-(bis(2,4,6-trichlorophenyl)methyl)-6-chloropyridin-2-yl)-9,10-dihydro-9,10-ethanoanthracene-11,12-diol in $CDCl_3$.

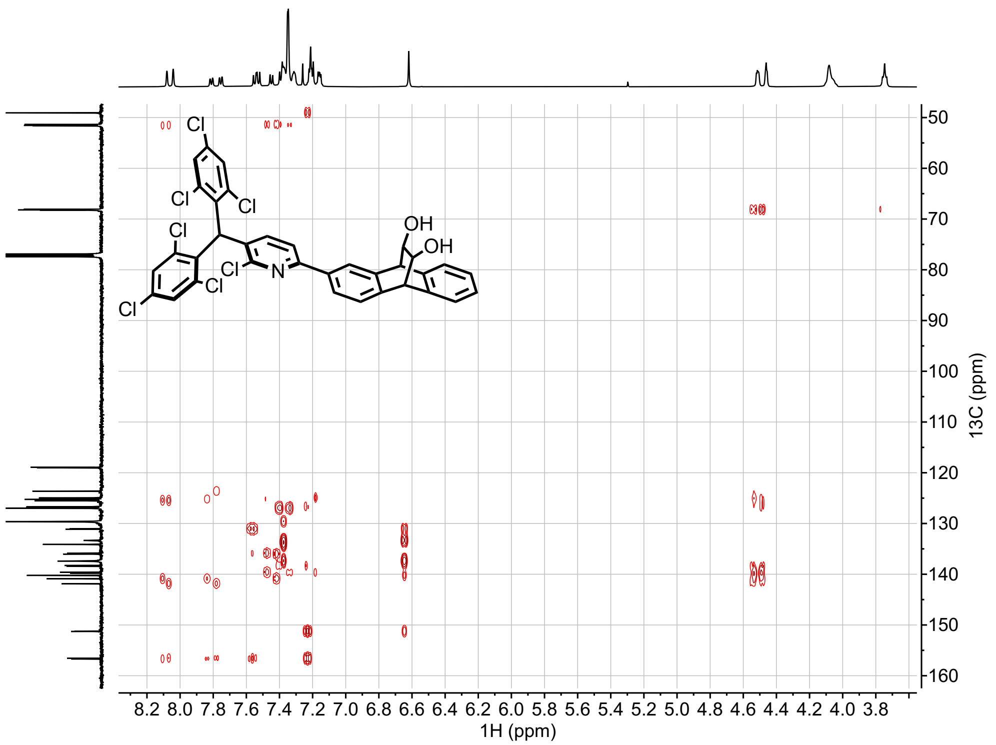

Figure S91: ^{1}H-^{13}C HMBC of 2-(5-(bis(2,4,6-trichlorophenyl)methyl)-6-chloropyridin-2-yl)-9,10-dihydro-9,10-ethanoanthracene-11,12-diol in $CDCl_3$.

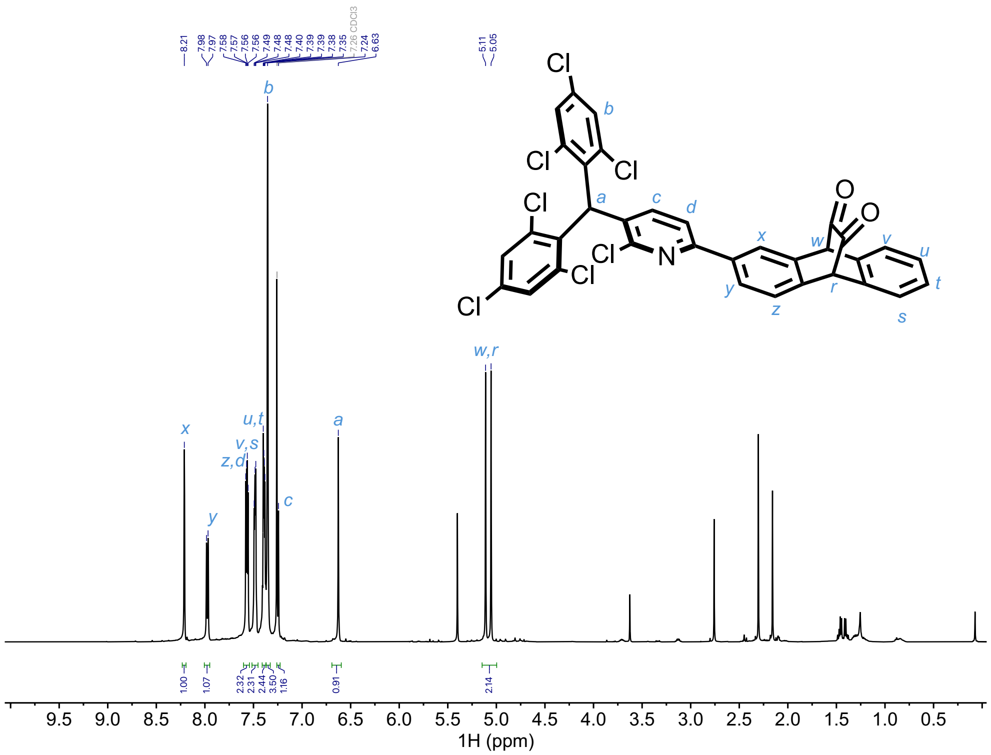

Figure S92: ^{1}H NMR (500 MHz, $CDCl_3$) of (9R,10S)-2-(5-(bis(2,4,6-trichlorophenyl)methyl)-6-chloropyridin-2-yl)-9,10-dihydro-9,10-ethanoanthracene-11,12-dione.

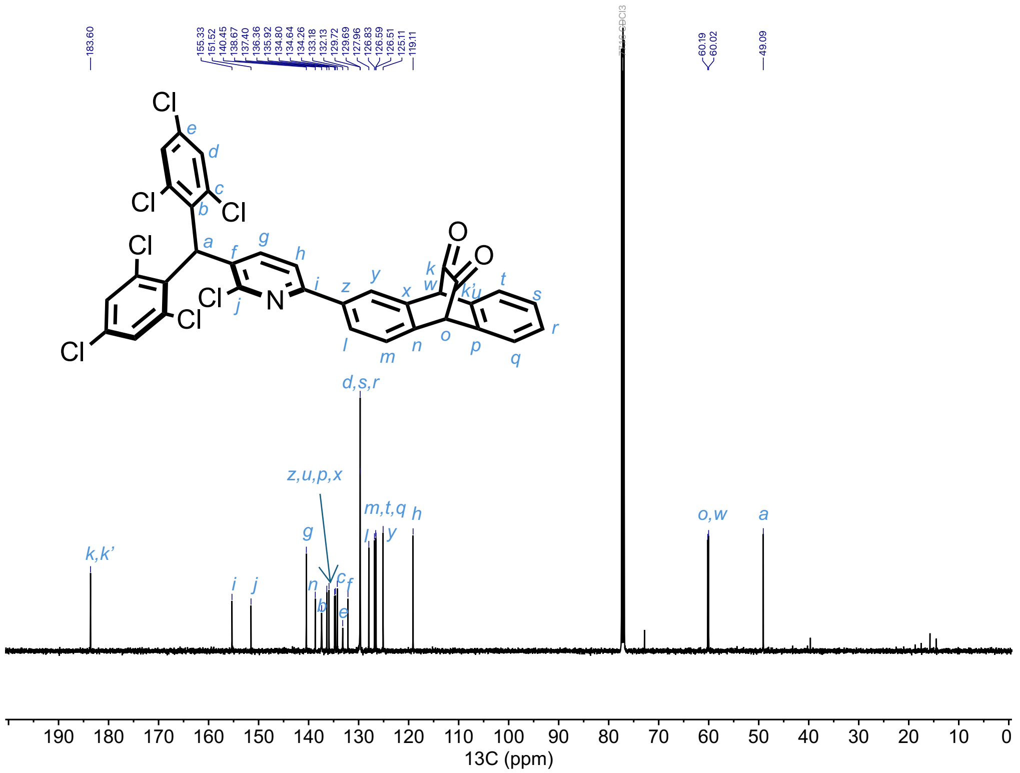

Figure S93: $^{13}C\{^{1}H\}$ NMR (126 MHz, $CDCl_3$) of (9R,10S)-2-(5-(bis(2,4,6-trichlorophenyl)methyl)-6-chloropyridin-2-yl)-9,10-dihydro-9,10-ethanoanthracene-11,12-dione.

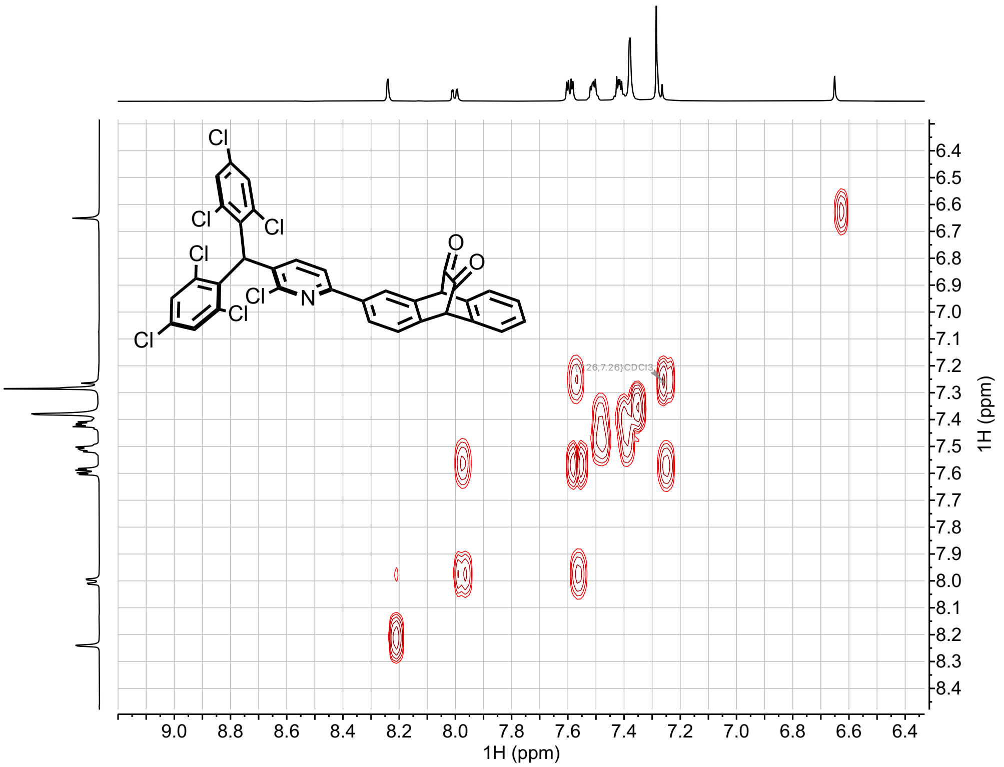

Figure S94: ^{1}H-^{1}H COSY of (9R,10S)-2-(5-(bis(2,4,6-trichlorophenyl)methyl)-6-chloropyridin-2-yl)-9,10-dihydro-9,10-ethanoanthracene-11,12-dione in $CDCl_3$.

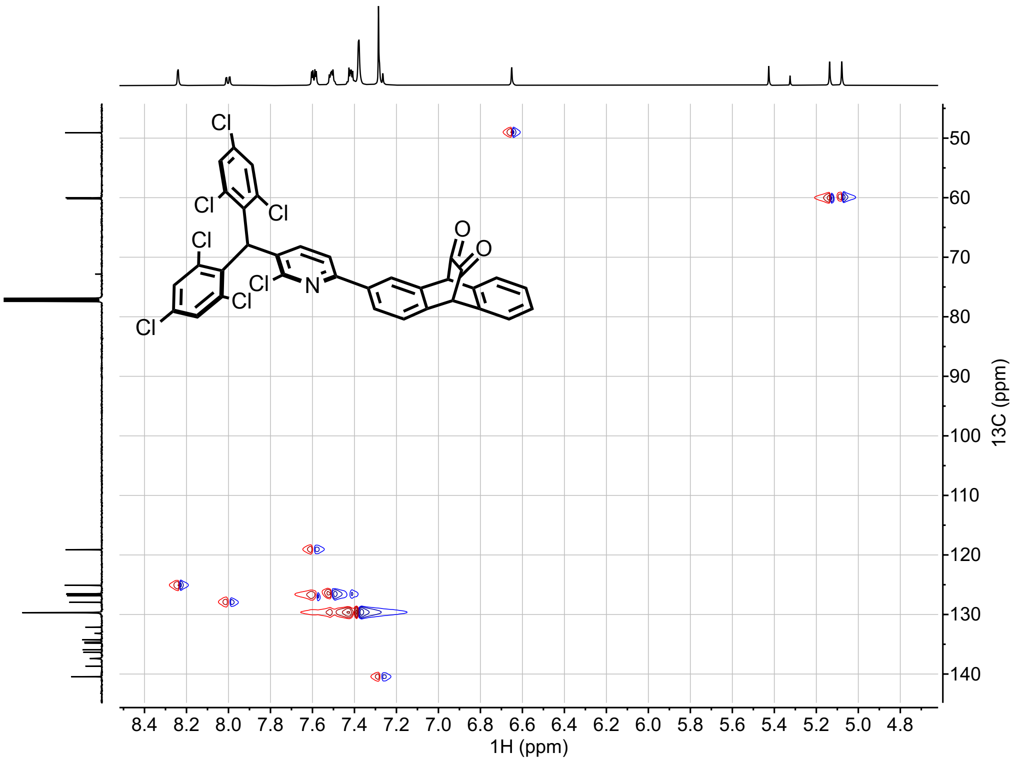

Figure S95: ^{1}H-^{13}C HSQC of (9R,10S)-2-(5-(bis(2,4,6-trichlorophenyl)methyl)-6-chloropyridin-2-yl)-9,10-dihydro-9,10-ethanoanthracene-11,12-dione in $CDCl_3$.

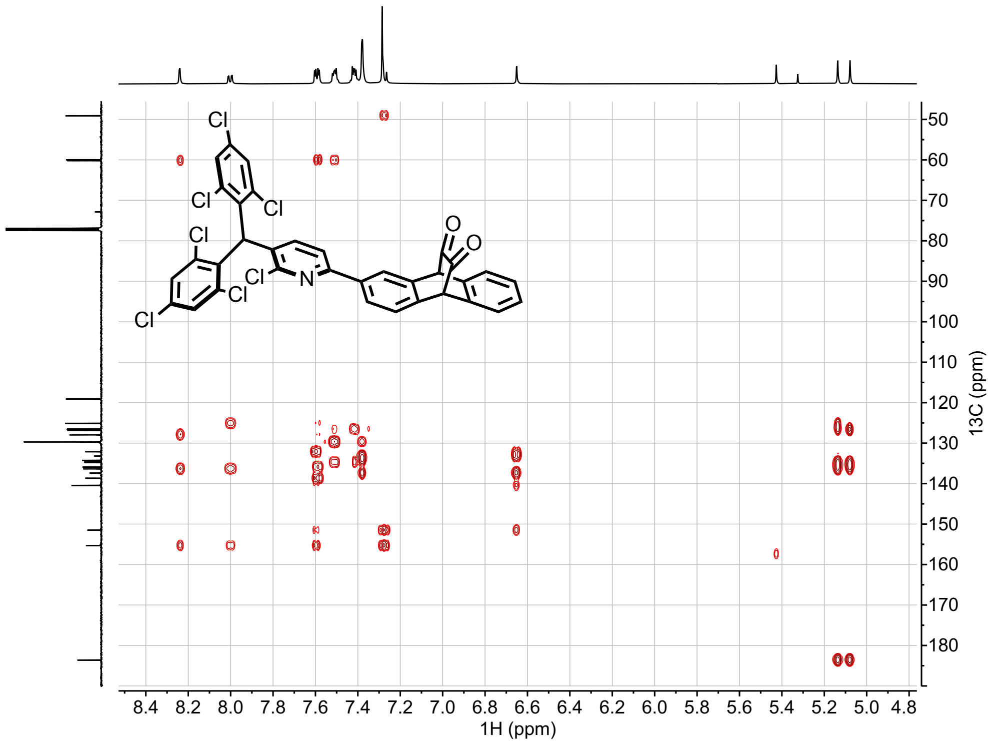

Figure S96: ^{1}H-^{13}C HMBC of (9R,10S)-2-(5-(bis(2,4,6-trichlorophenyl)methyl)-6-chloropyridin-2-yl)-9,10-dihydro-9,10-ethanoanthracene-11,12-dione in $CDCl_3$.

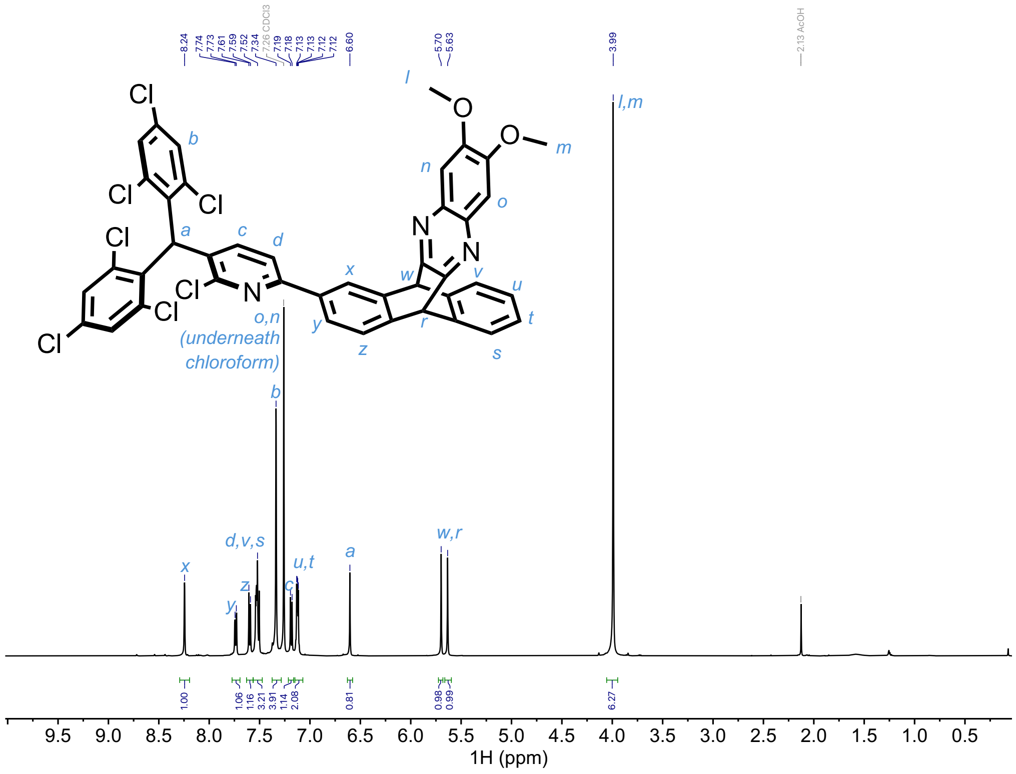

Figure S97: ^{1}H NMR (500 MHz, $CDCl_3$) of compound **1H**.

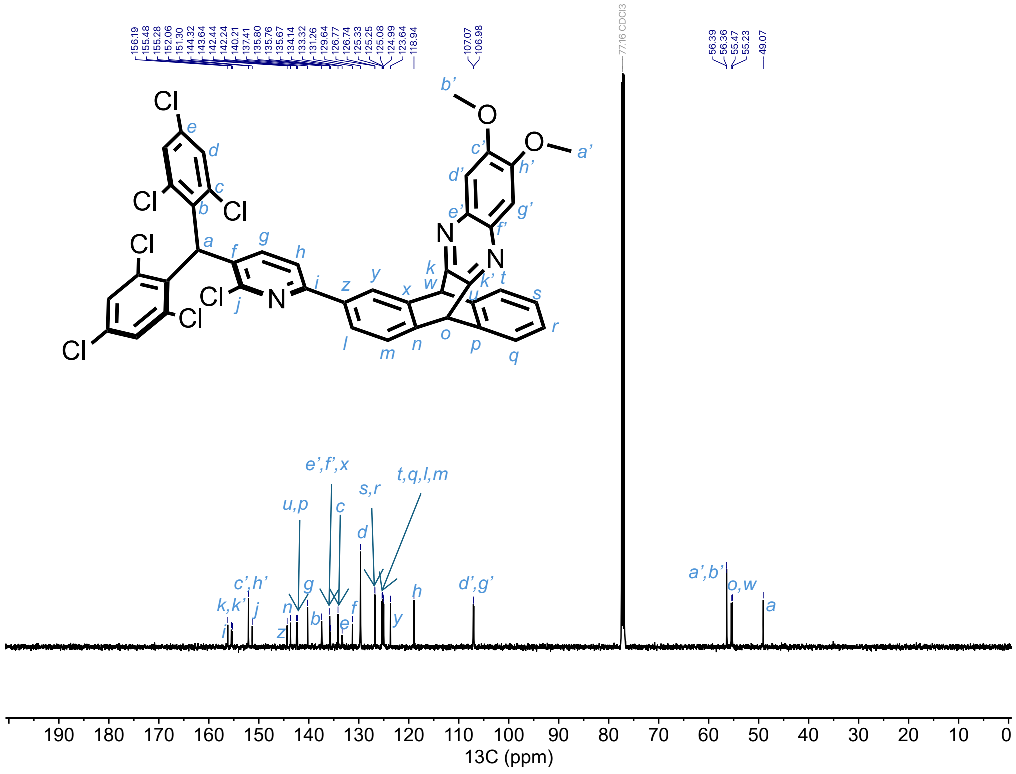

Figure S98: $^{13}C\{^{1}H\}$ NMR (126 MHz, $CDCl_3$) of compound **1H**.

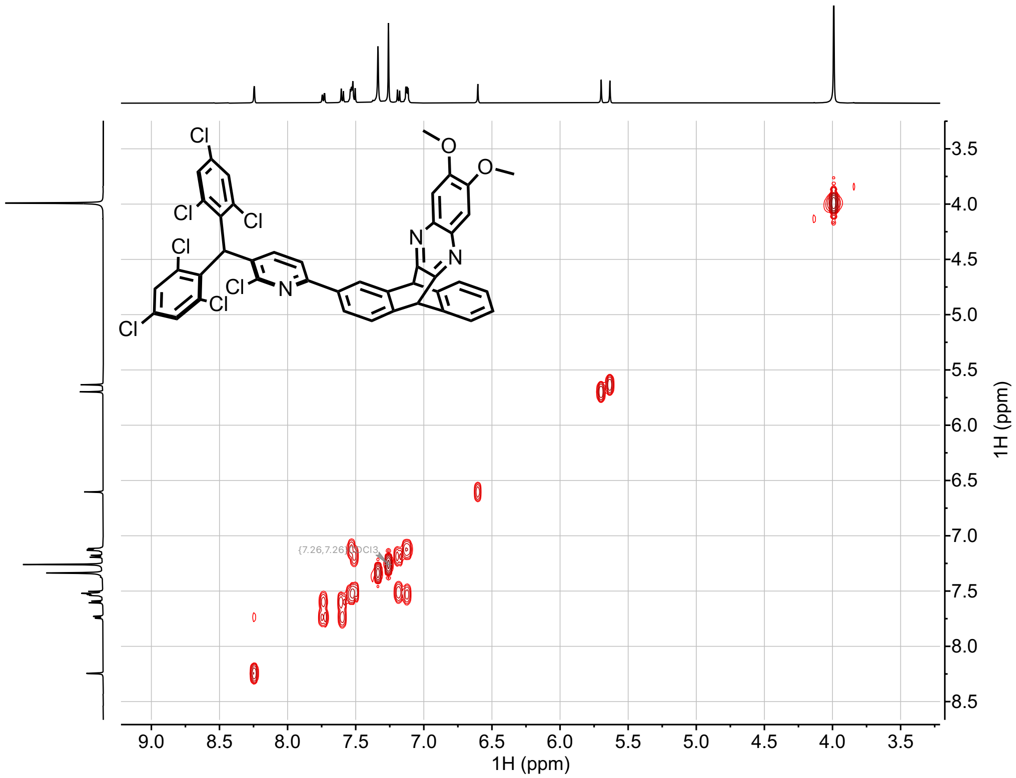

Figure S99: ^{1}H-^{1}H COSY of compound **1H** in $CDCl_3$.

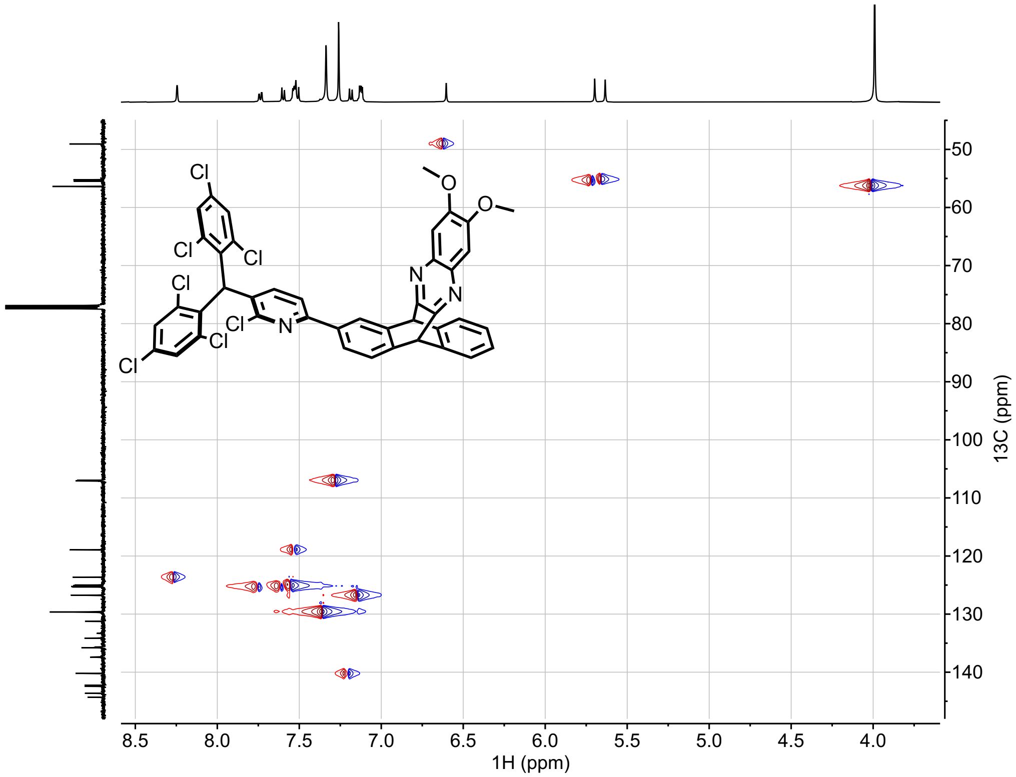

Figure S100: ^{1}H-^{13}C HSQC of compound **1H** in $CDCl_3$.

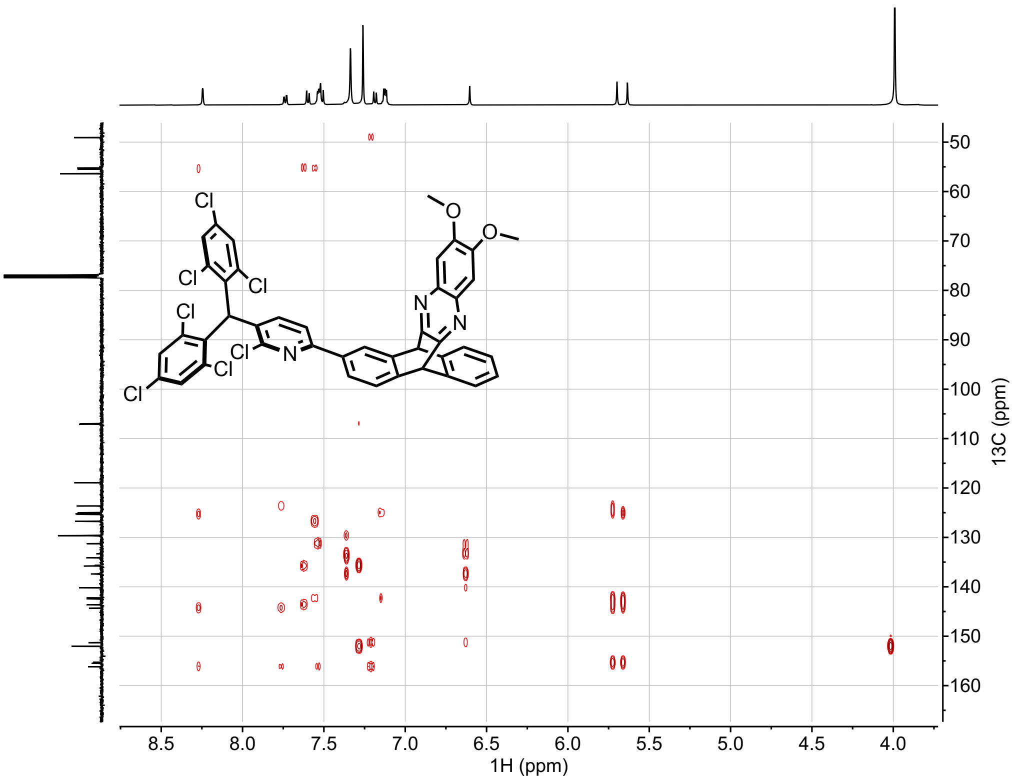

Figure S101: ^{1}H-^{13}C HMBC of compound **1H** in $CDCl_3$.

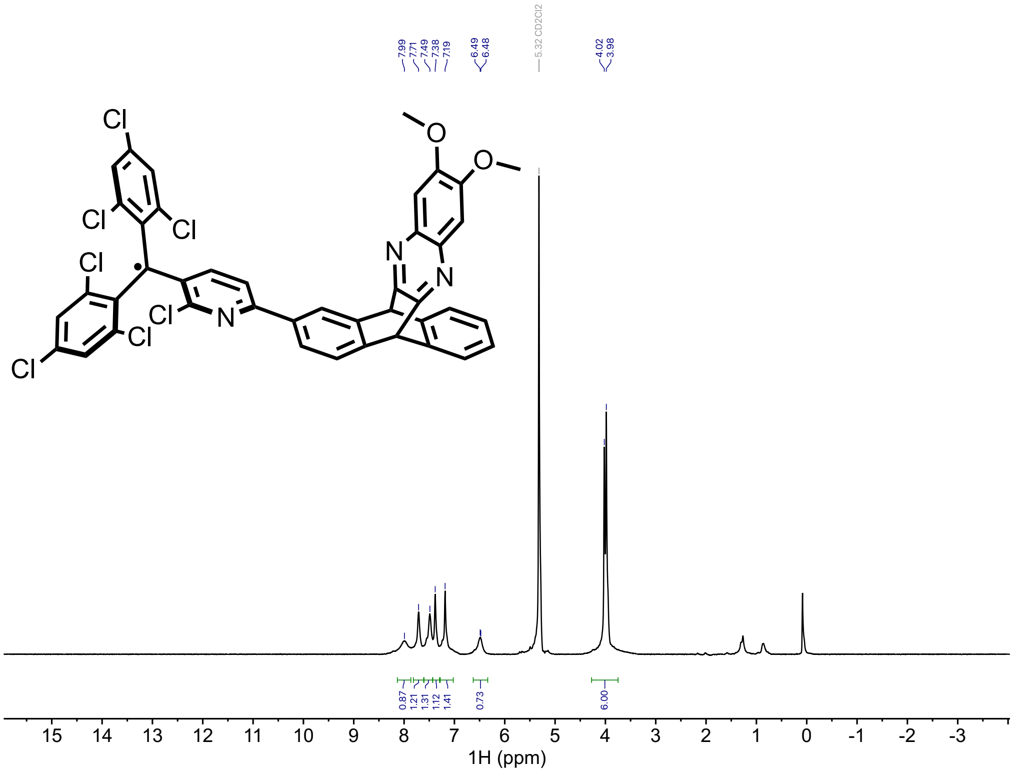

Figure S102: ^{1}H NMR (500 MHz, CD_2Cl_2) of compound **1** at room temperature.

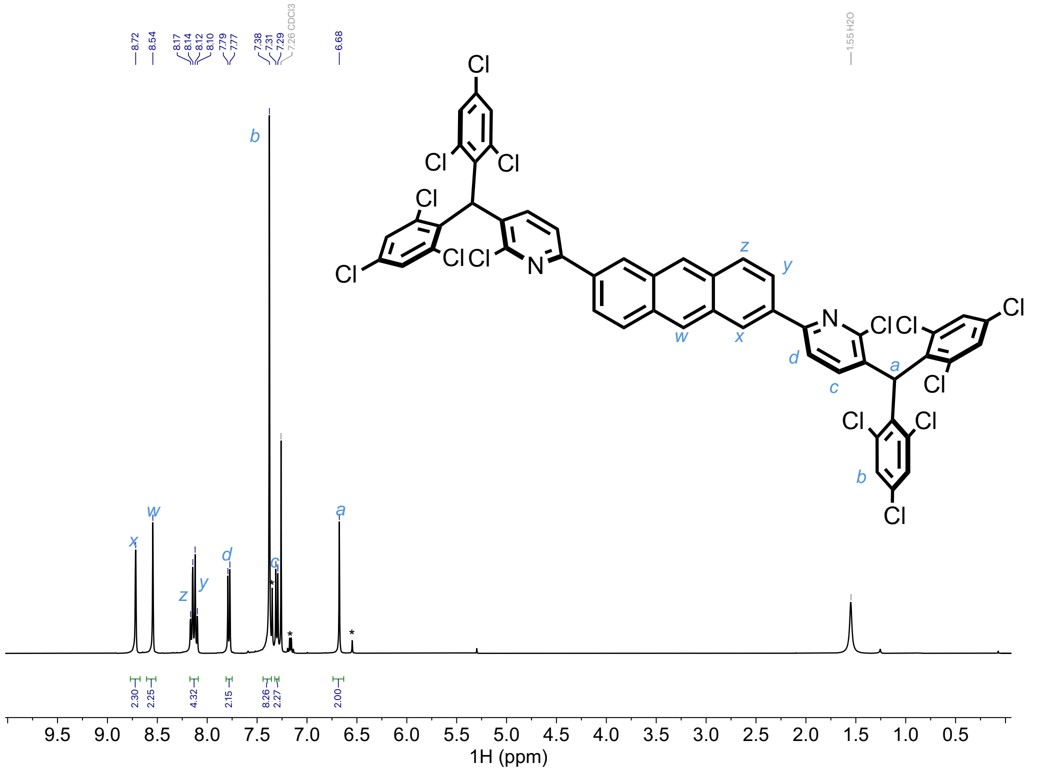

Figure S103: ^{1}H NMR (400 MHz, $CDCl_3$) of 2,6-bis(5-(bis(2,4,6-trichlorophenyl)methyl)-6-chloropyridin-2-yl)anthracene. *indicates signals attributed to residual pyTTM starting material.

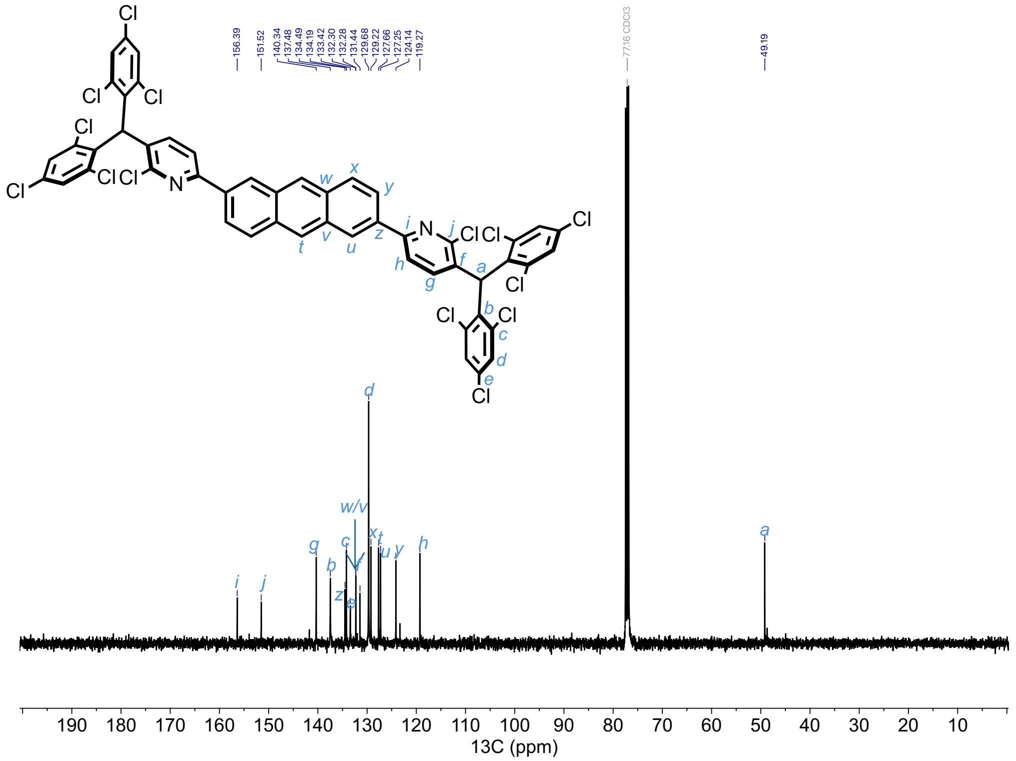

Figure S104: $^{13}C\{^{1}H\}$ NMR (101 MHz, $CDCl_3$) of 2,6-bis(5-(bis(2,4,6-trichlorophenyl)methyl)-6-chloropyridin-2-yl)anthracene.

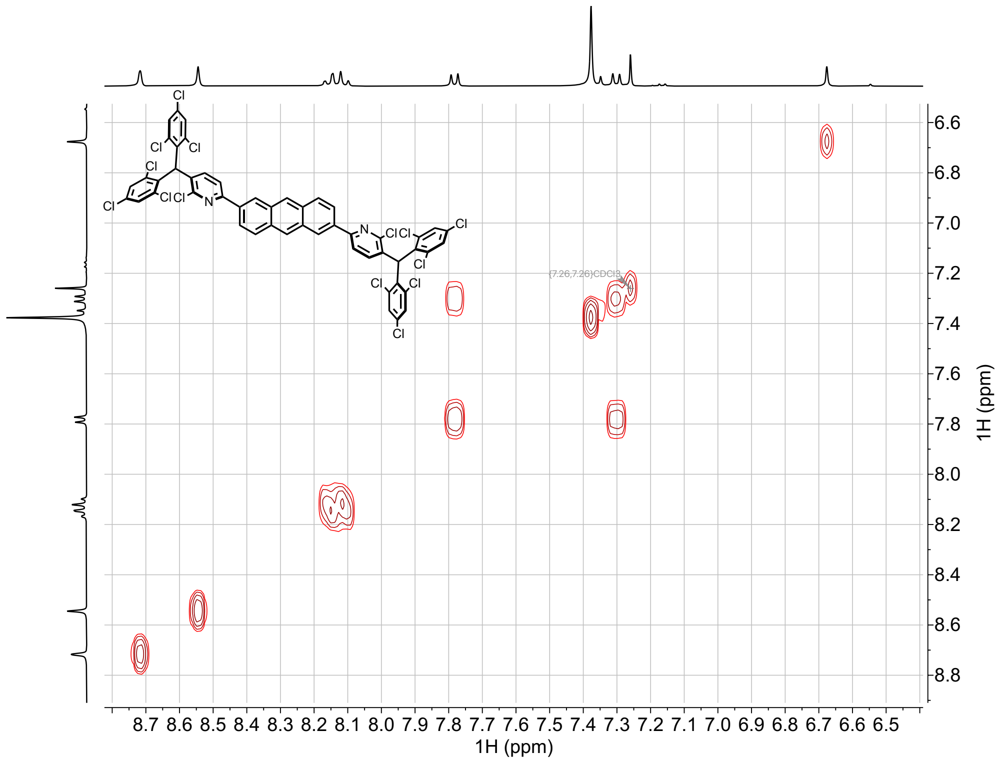

Figure S105: ^1H-^1H COSY of 2,6-bis(5-(bis(2,4,6-trichlorophenyl)methyl)-6-chloropyridin-2-yl)anthracene in $CDCl_3$.

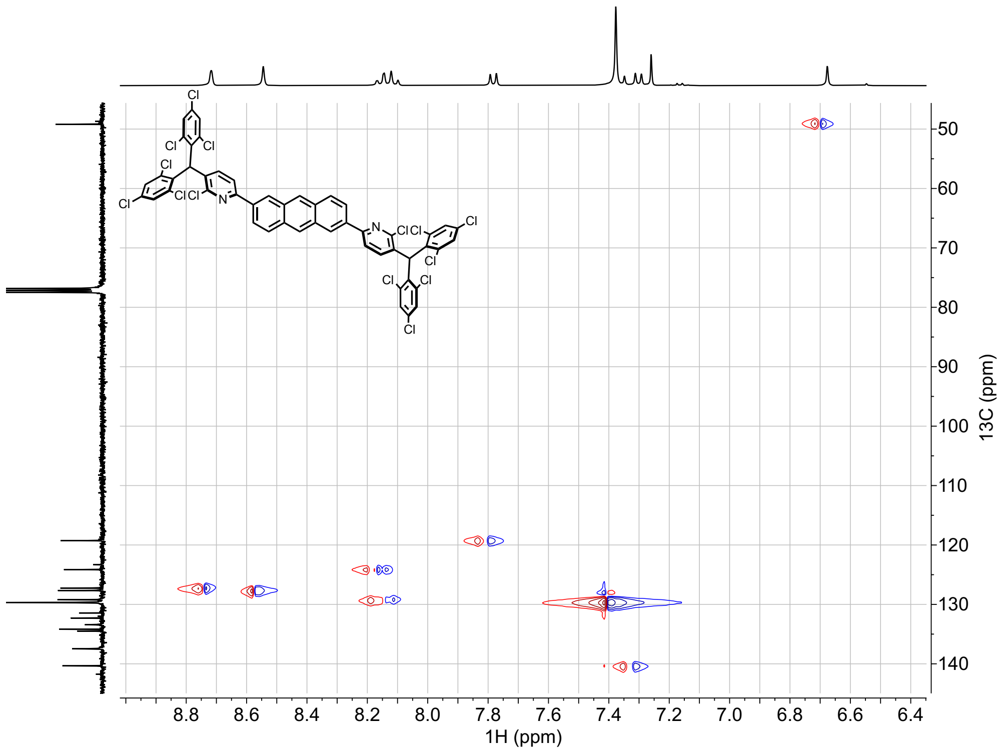

Figure S106: ^1H-^{13}C HSQC of 2,6-bis(5-(bis(2,4,6-trichlorophenyl)methyl)-6-chloropyridin-2-yl)anthracene in $CDCl_3$.

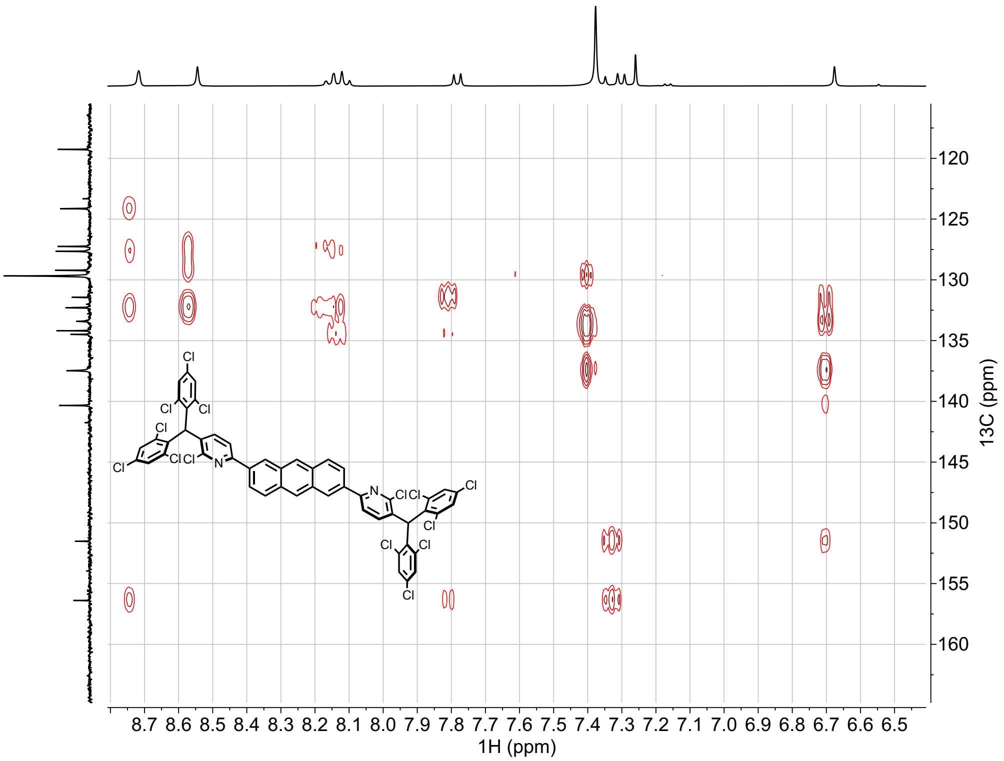

Figure S107: ^{1}H-^{13}C HMBC of 2,6-bis(5-(bis(2,4,6-trichlorophenyl)methyl)-6-chloropyridin-2-yl)anthracene in $CDCl_3$.

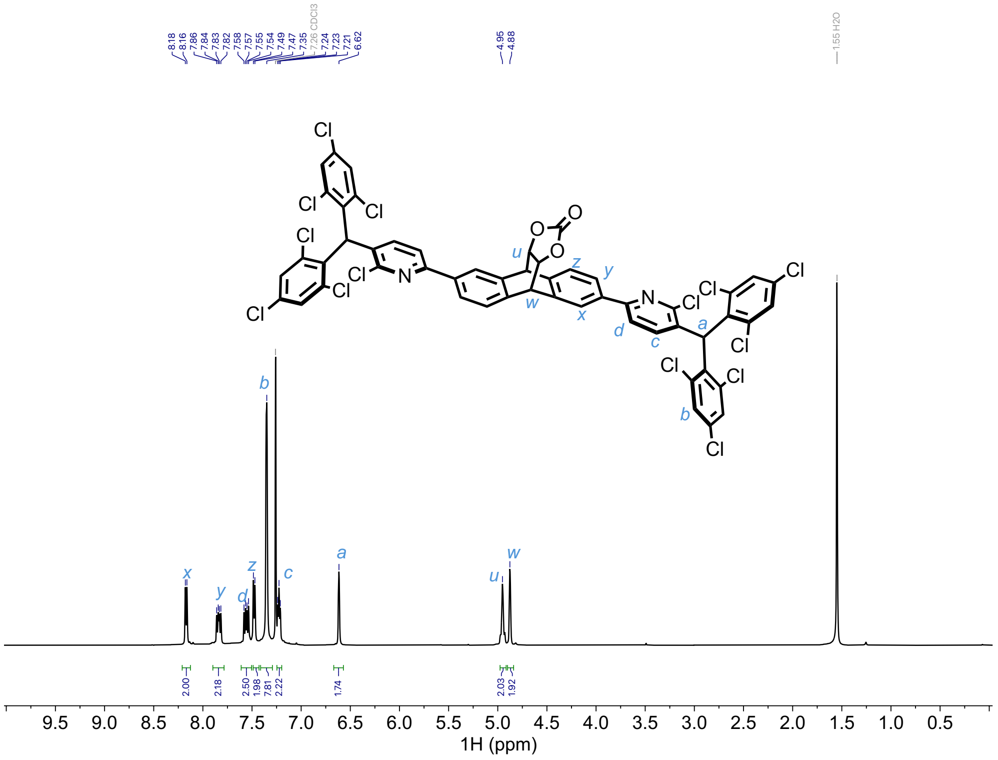

Figure S108: ^{1}H NMR (500 MHz, $CDCl_3$) of 2,6-bis(5-(bis(2,4,6-trichlorophenyl)methyl)-6-chloropyridin-2-yl)-9,10-dihydro-9,10-[4,5]epidioxoloanthracen-13-one.

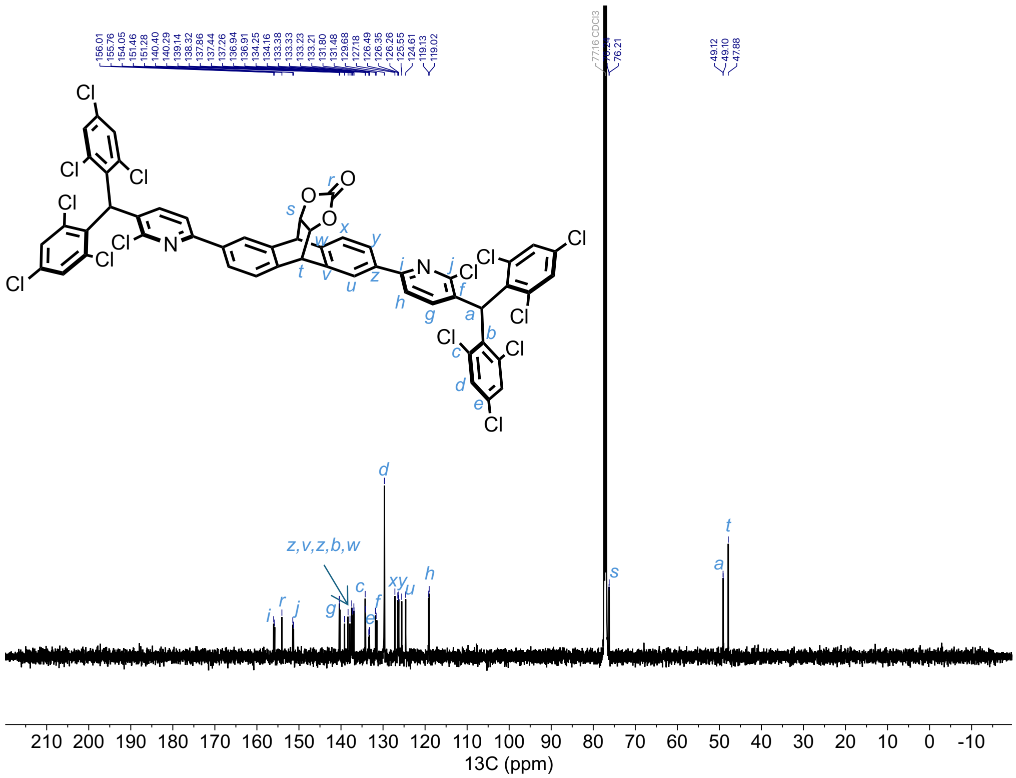

Figure S109: $^{13}C\{^{1}H\}$ NMR (126 MHz, $CDCl_3$) of 2,6-bis(5-(bis(2,4,6-trichlorophenyl)methyl)-6-chloropyridin-2-yl)-9,10-dihydro-9,10-[4,5]epidioxoloanthracen-13-one.

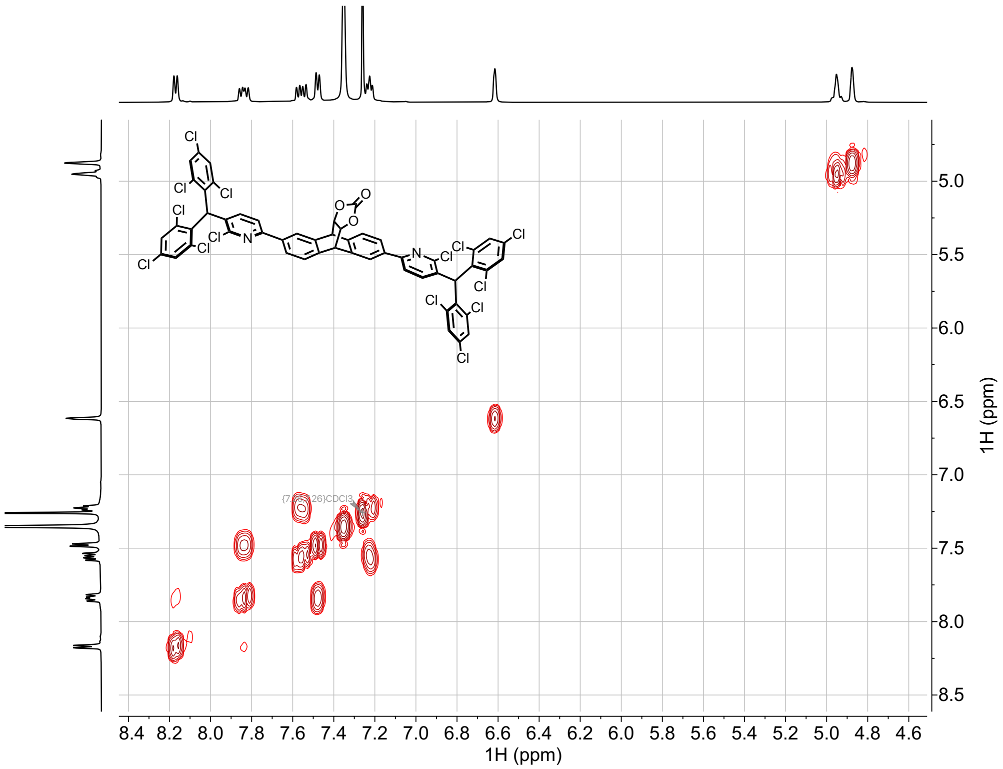

Figure S110: ^{1}H-^{1}H COSY of 2,6-bis(5-(bis(2,4,6-trichlorophenyl)methyl)-6-chloropyridin-2-yl)-9,10-dihydro-9,10-[4,5]epidioxoloanthracen-13-one in $CDCl_3$.

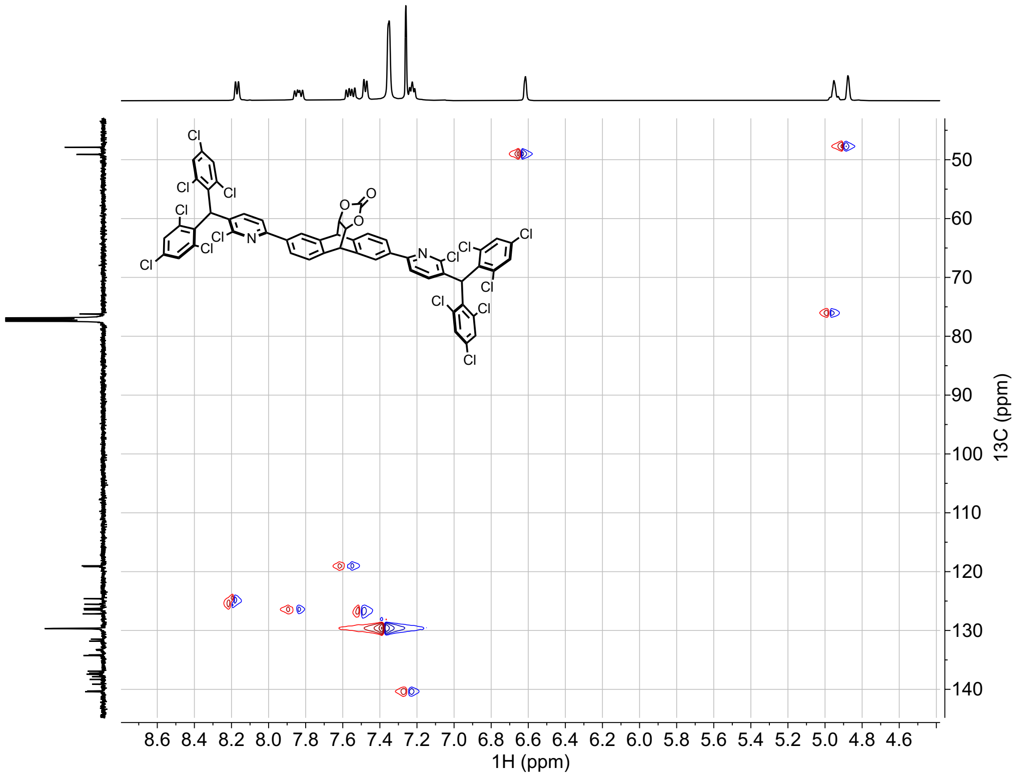

Figure S111: ^{1}H-^{13}C HSQC of 2,6-bis(5-(bis(2,4,6-trichlorophenyl)methyl)-6-chloropyridin-2-yl)-9,10-dihydro-9,10-[4,5]epidioxoloanthracen-13-one in $CDCl_3$.

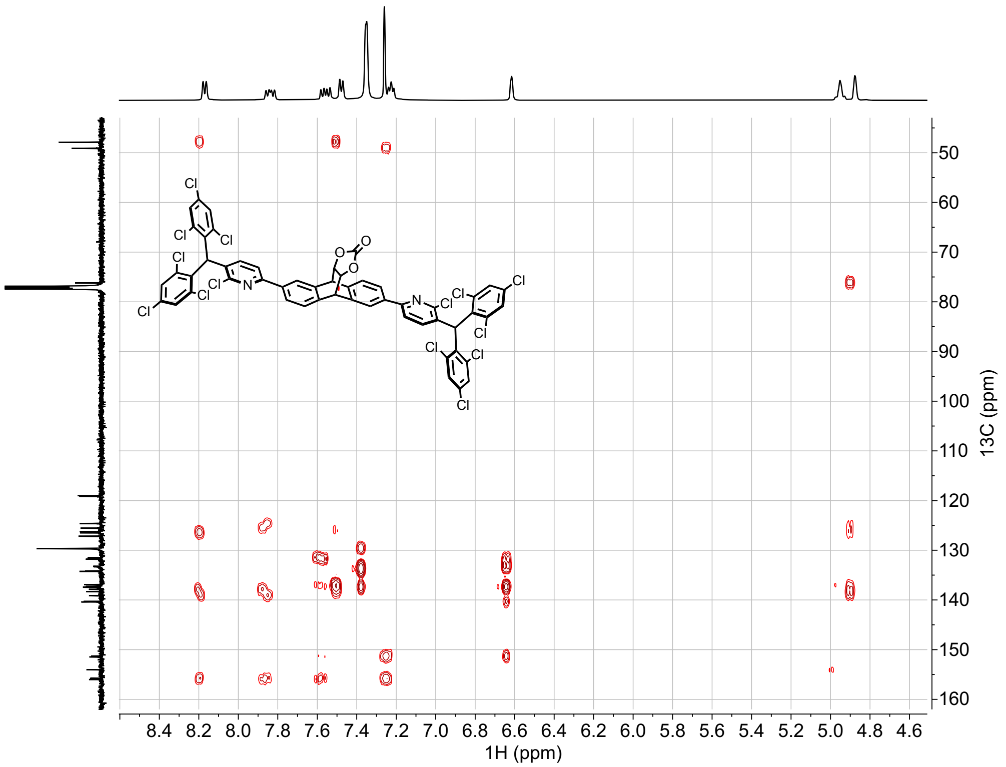

Figure S112: ^{1}H-^{13}C HMBC of 2,6-bis(5-(bis(2,4,6-trichlorophenyl)methyl)-6-chloropyridin-2-yl)-9,10-dihydro-9,10-[4,5]epidioxoloanthracen-13-one in $CDCl_3$.

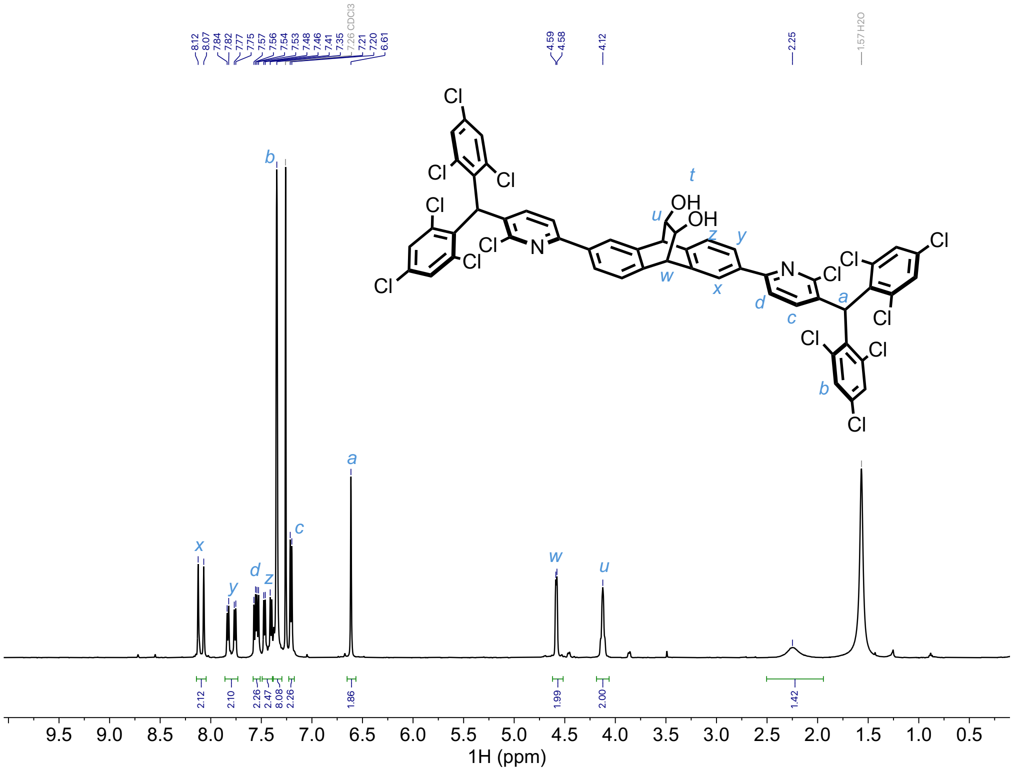

Figure S1113: ^{1}H NMR (500 MHz, $CDCl_3$) of 2,6-bis(5-(bis(2,4,6-trichlorophenyl)methyl)-6-chloropyridin-2-yl)-9,10-dihydro-9,10-ethanoanthracene-11,12-diol.

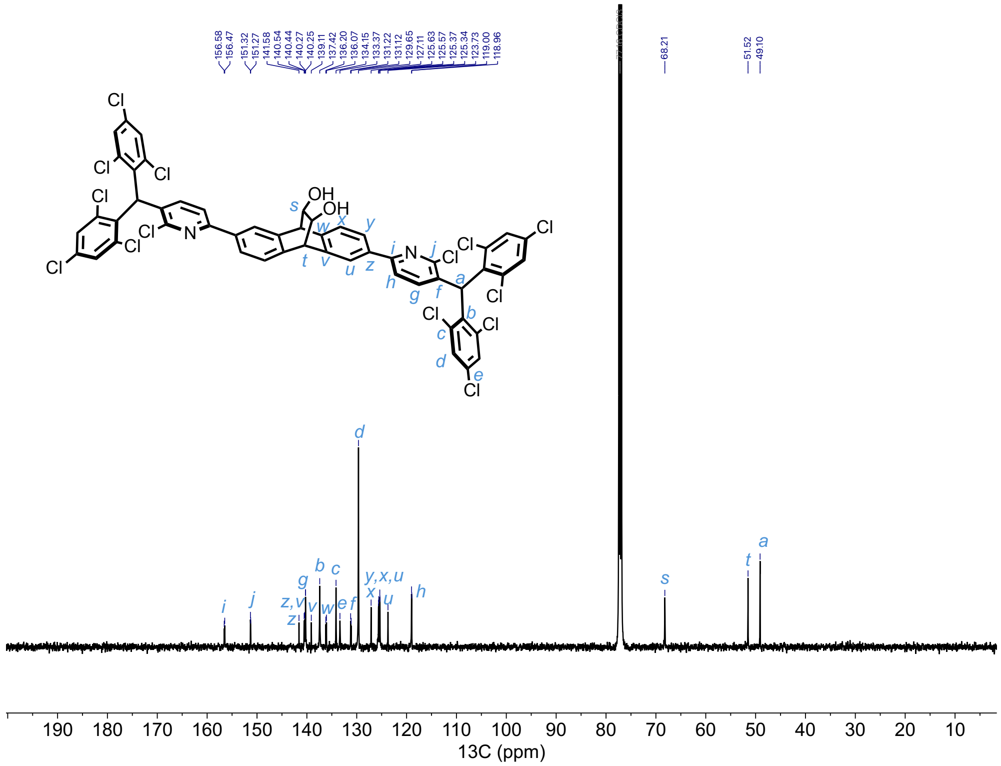

Figure S114: $^{13}C\{^{1}H\}$ NMR (126 MHz, $CDCl_3$) of 2,6-bis(5-(bis(2,4,6-trichlorophenyl)methyl)-6-chloropyridin-2-yl)-9,10-dihydro-9,10-ethanoanthracene-11,12-diol.

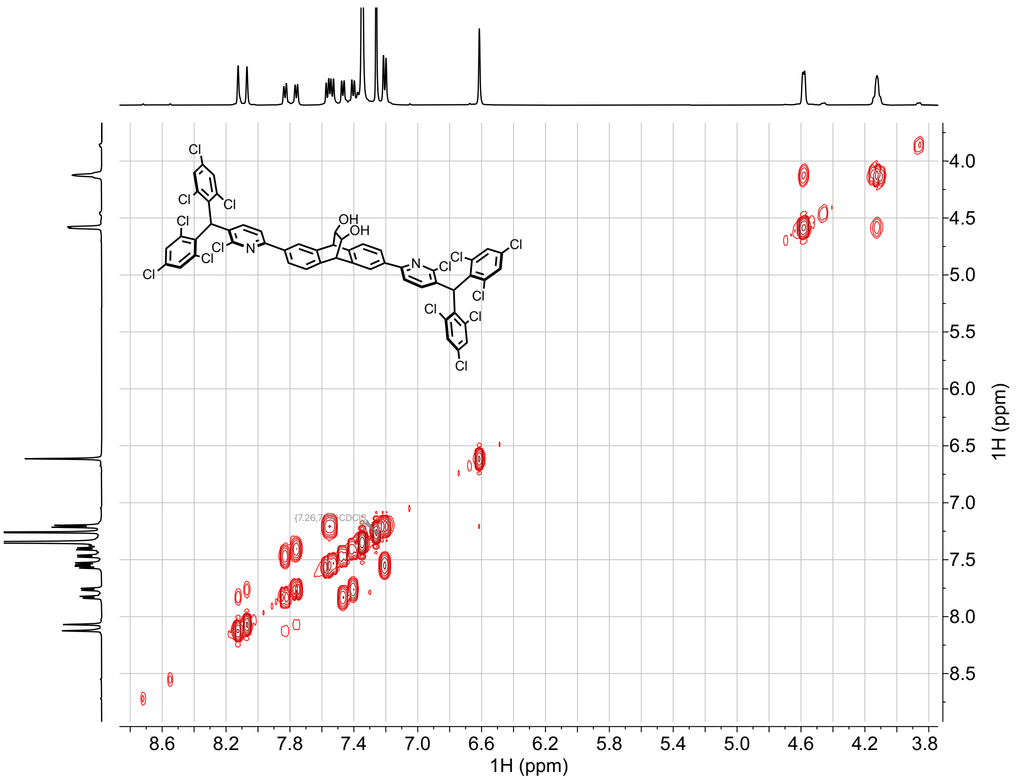

Figure S115: ^{1}H-^{1}H COSY of 2,6-bis(5-(bis(2,4,6-trichlorophenyl)methyl)-6-chloropyridin-2-yl)-9,10-dihydro-9,10-ethanoanthracene-11,12-diol in $CDCl_3$.

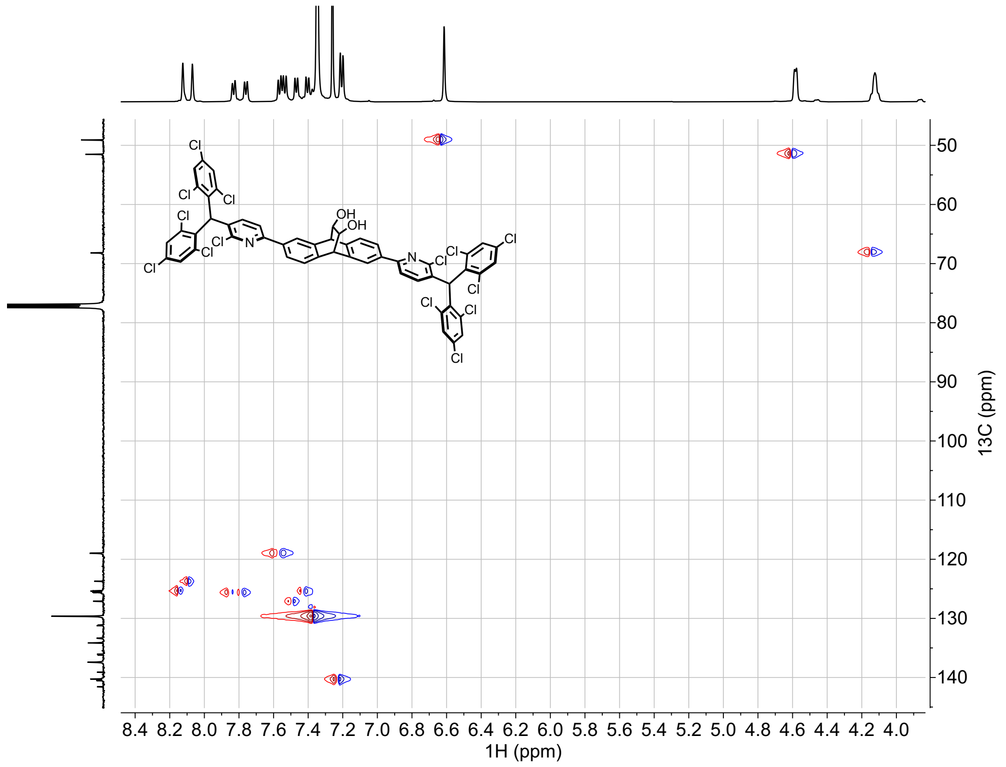

Figure S116: ^{1}H-^{13}C HSQC of 2,6-bis(5-(bis(2,4,6-trichlorophenyl)methyl)-6-chloropyridin-2-yl)-9,10-dihydro-9,10-ethanoanthracene-11,12-diol in $CDCl_3$.

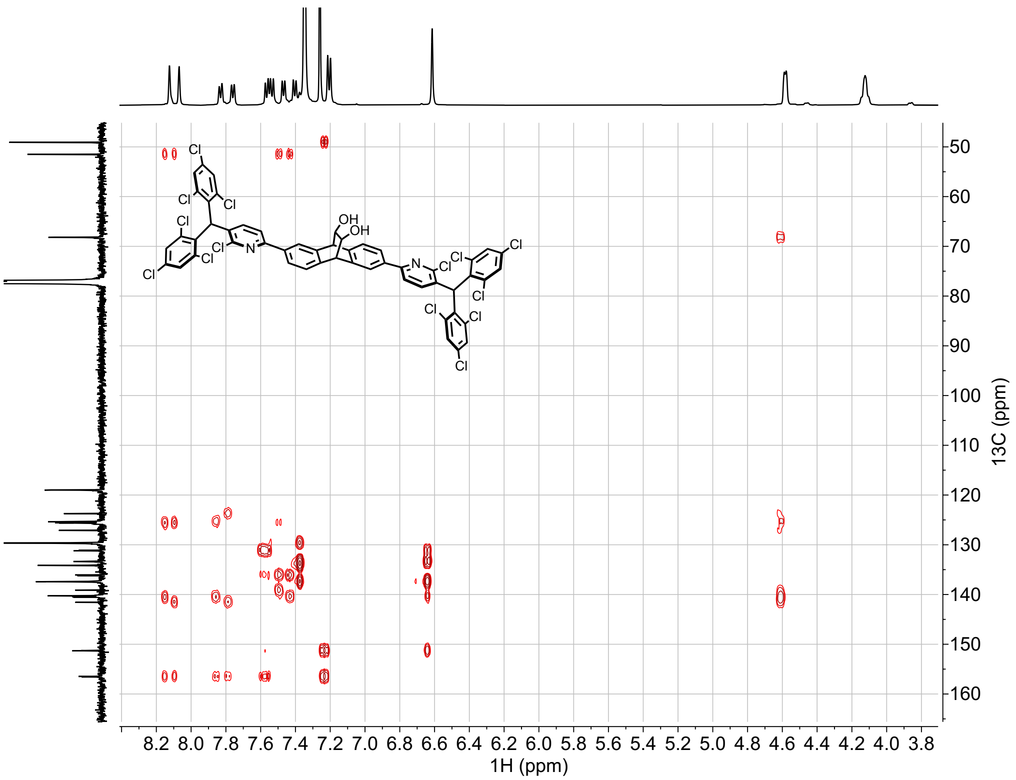

Figure S117: ^{1}H-^{13}C HMBC of 2,6-bis(5-(bis(2,4,6-trichlorophenyl)methyl)-6-chloropyridin-2-yl)-9,10-dihydro-9,10-ethanoanthracene-11,12-diol compound in $CDCl_3$.

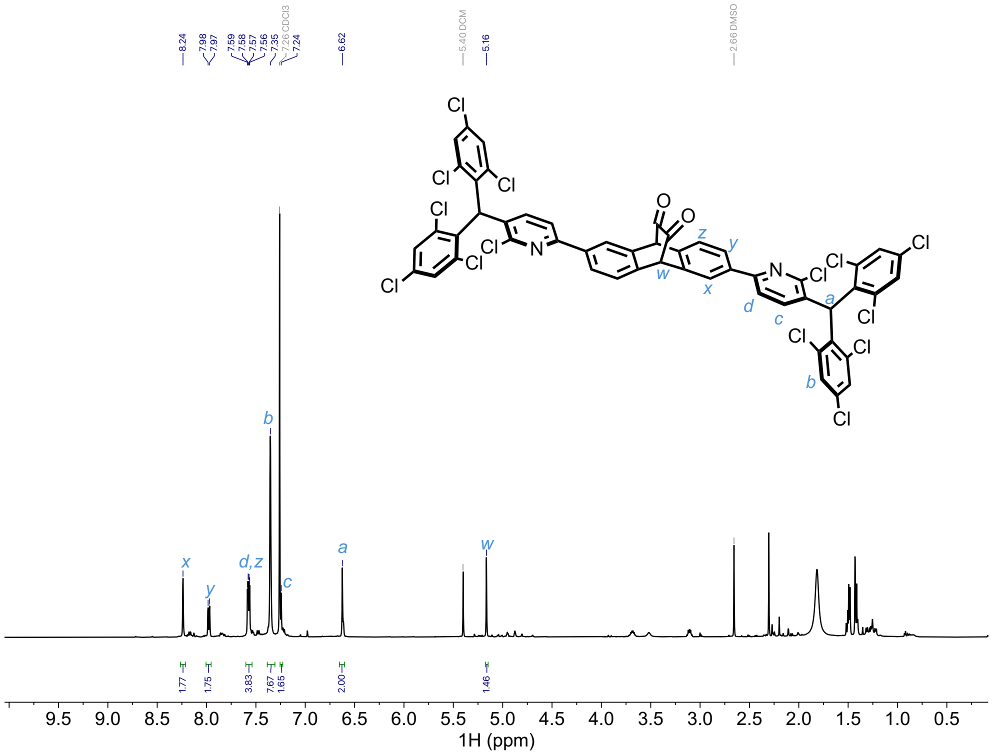

Figure S118: ^{1}H NMR (500 MHz, $CDCl_3$) of (9R,10R)-2,6-bis(5-(bis(2,4,6-trichlorophenyl)methyl)-6-chloropyridin-2-yl)-9,10-dihydro-9,10-ethanoanthracene-11,12-dione.

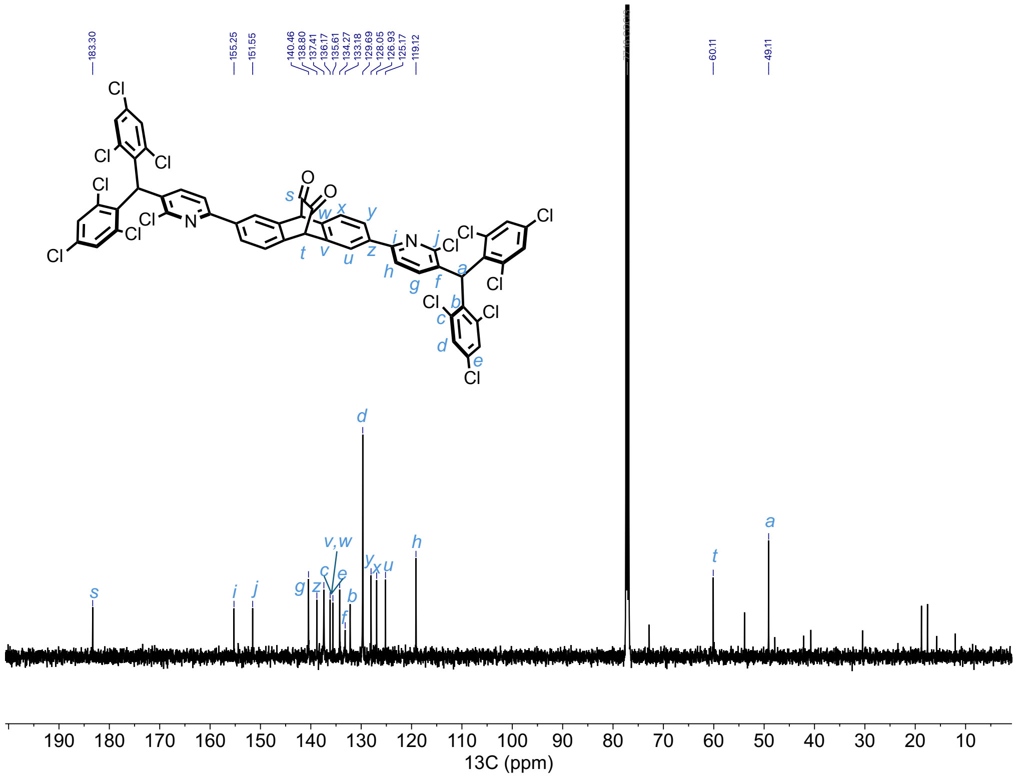

Figure S119: $^{13}C\{^{1}H\}$ NMR (126 MHz, $CDCl_3$) of (9R,10R)-2,6-bis(5-(bis(2,4,6-trichlorophenyl)methyl)-6-chloropyridin-2-yl)-9,10-dihydro-9,10-ethanoanthracene-11,12-dione.

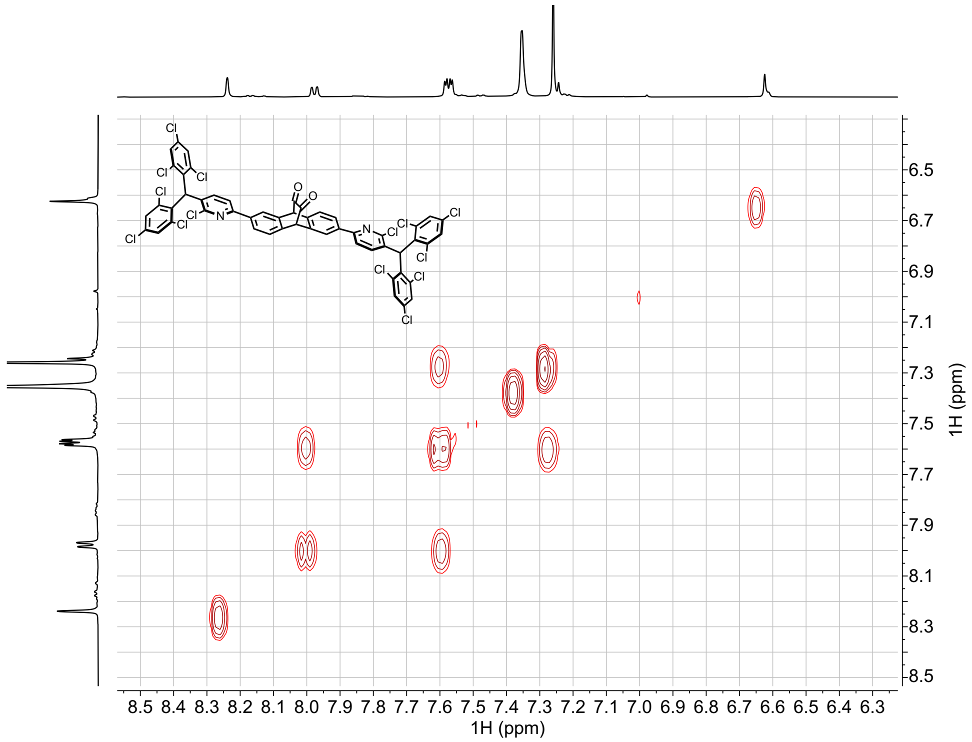

Figure S120: ^{1}H-^{1}H COSY of (9R,10R)-2,6-bis(5-(bis(2,4,6-trichlorophenyl)methyl)-6-chloropyridin-2-yl)-9,10-dihydro-9,10-ethanoanthracene-11,12-dione in $CDCl_3$.

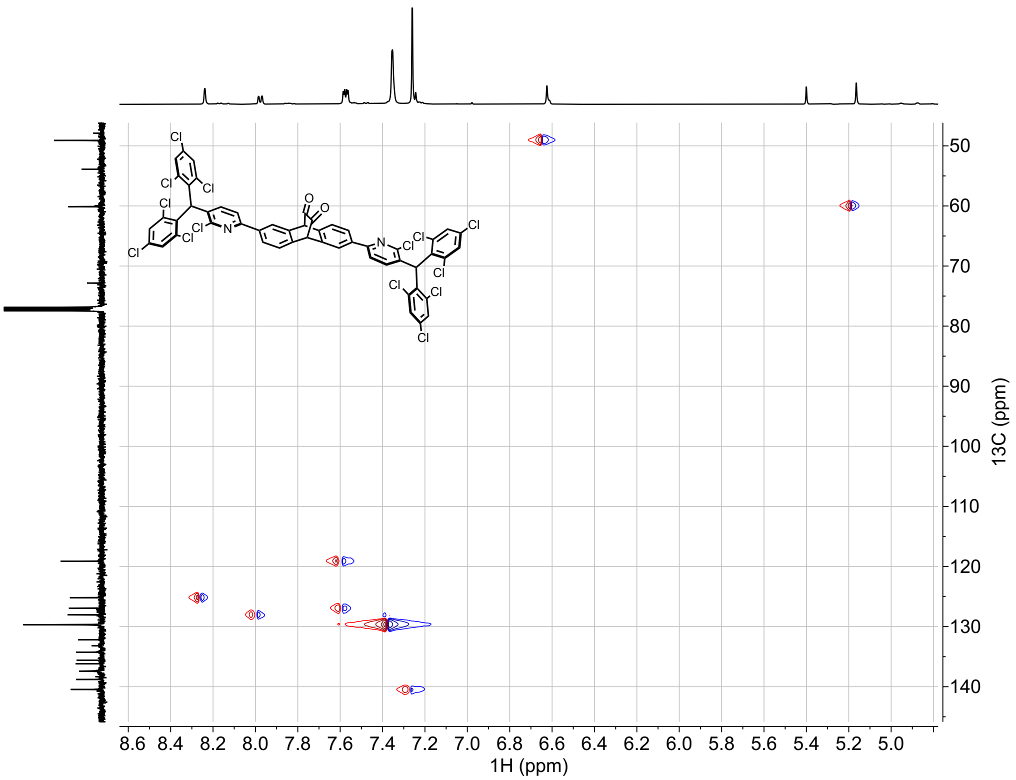

Figure S121: ^{1}H-^{13}C HSQC of (9R,10R)-2,6-bis(5-(bis(2,4,6-trichlorophenyl)methyl)-6-chloropyridin-2-yl)-9,10-dihydro-9,10-ethanoanthracene-11,12-dione in $CDCl_3$.

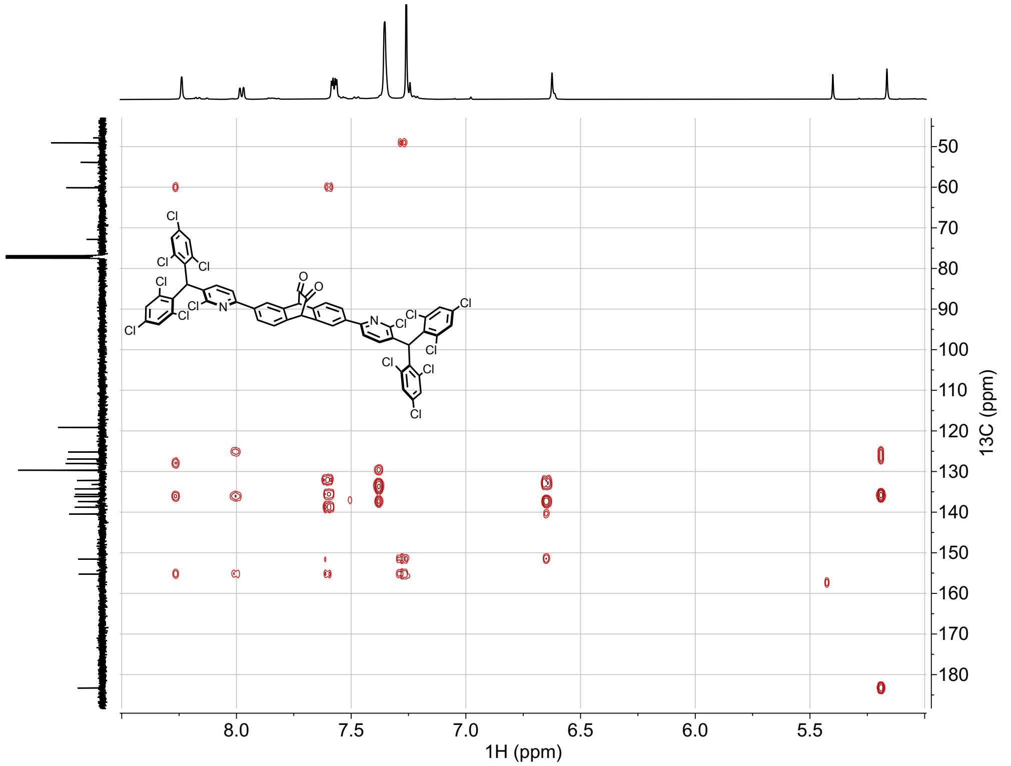

Figure S122: ^{1}H-^{13}C HMBC of (9R,10R)-2,6-bis(5-(bis(2,4,6-trichlorophenyl)methyl)-6-chloropyridin-2-yl)-9,10-dihydro-9,10-ethanoanthracene-11,12-dione in $CDCl_3$.

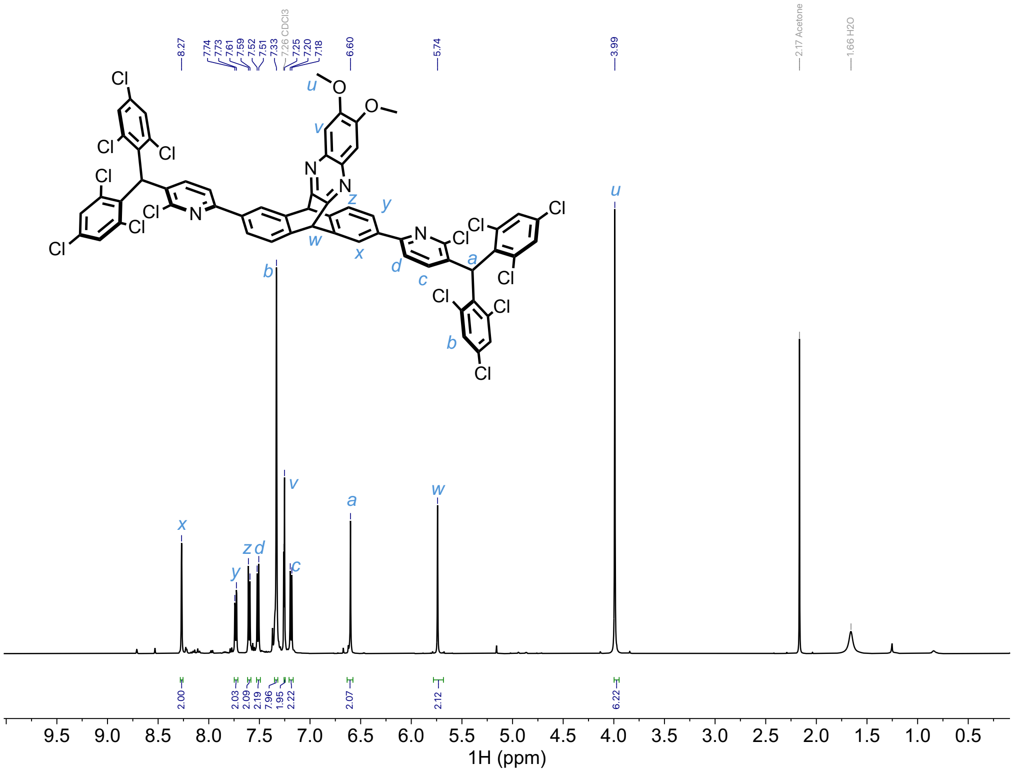

Figure S123: ^{1}H NMR (500 MHz, $CDCl_3$) of compound **2H**.

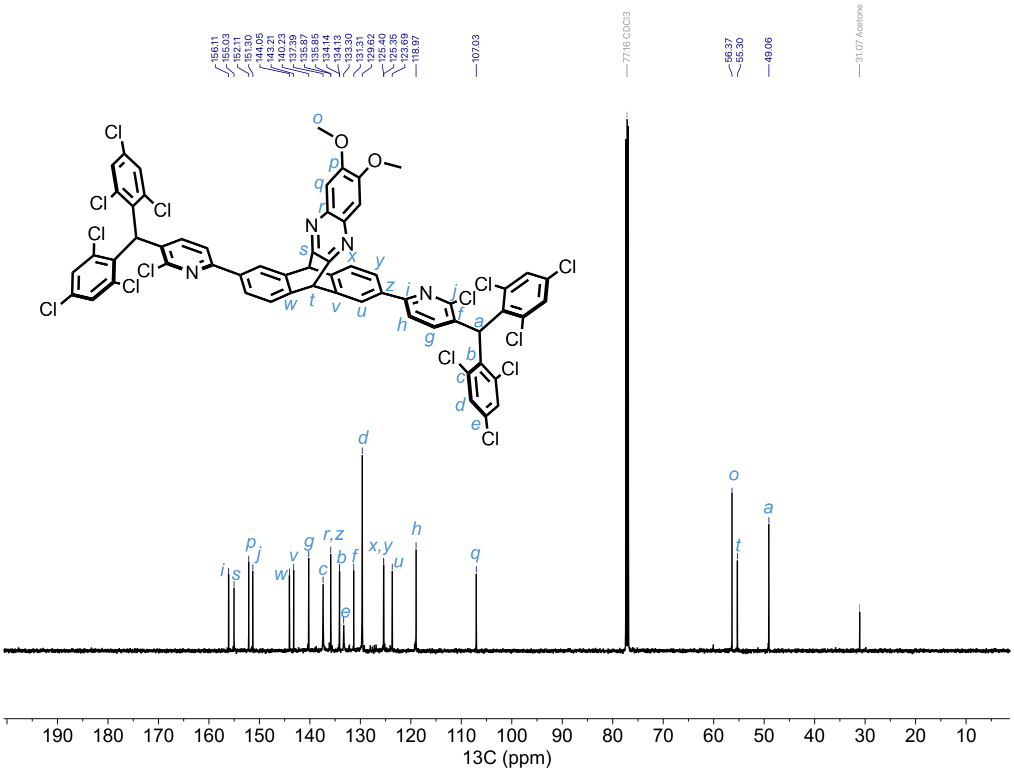

Figure S124: $^{13}C\{^{1}H\}$ NMR (126 MHz, $CDCl_3$) of compound **2H**.

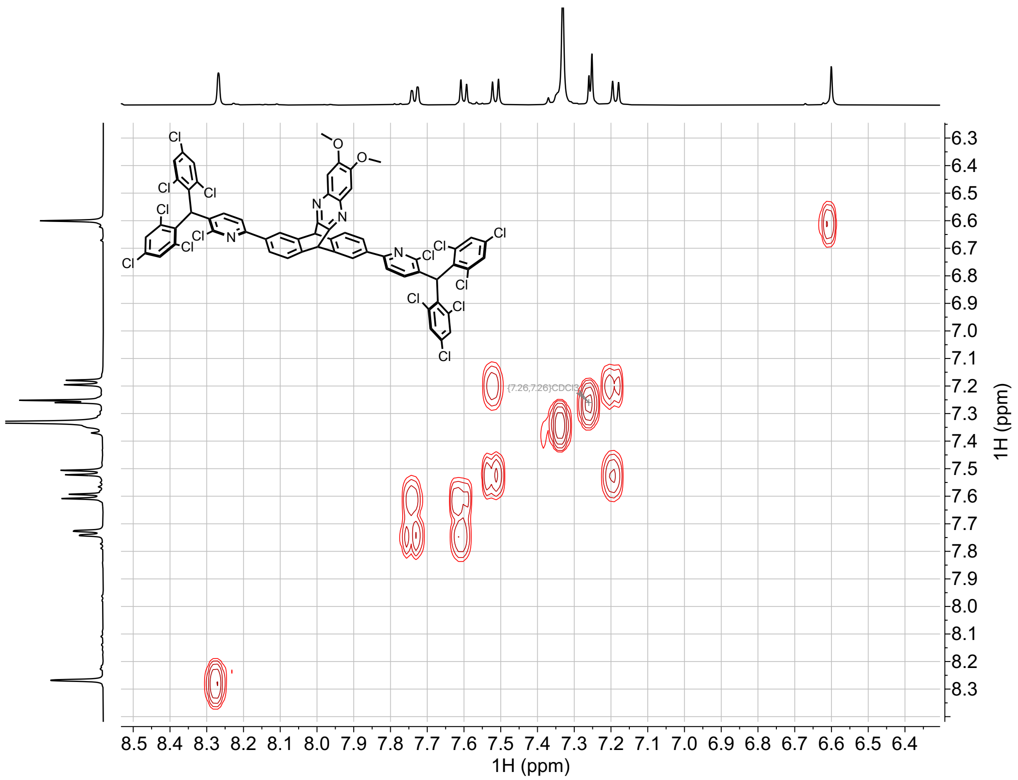

Figure S125: ^{1}H-^{1}H COSY of compound **2H** in $CDCl_3$.

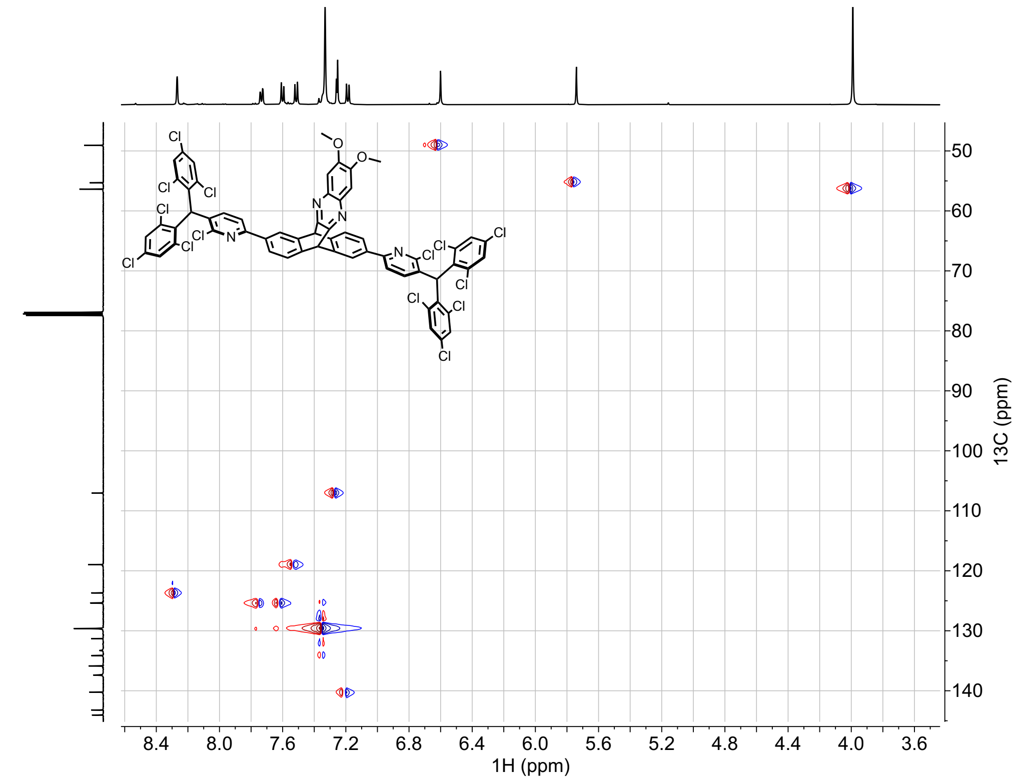

Figure S126: ^{1}H-^{13}C HSQC of compound **2H** in $CDCl_3$.

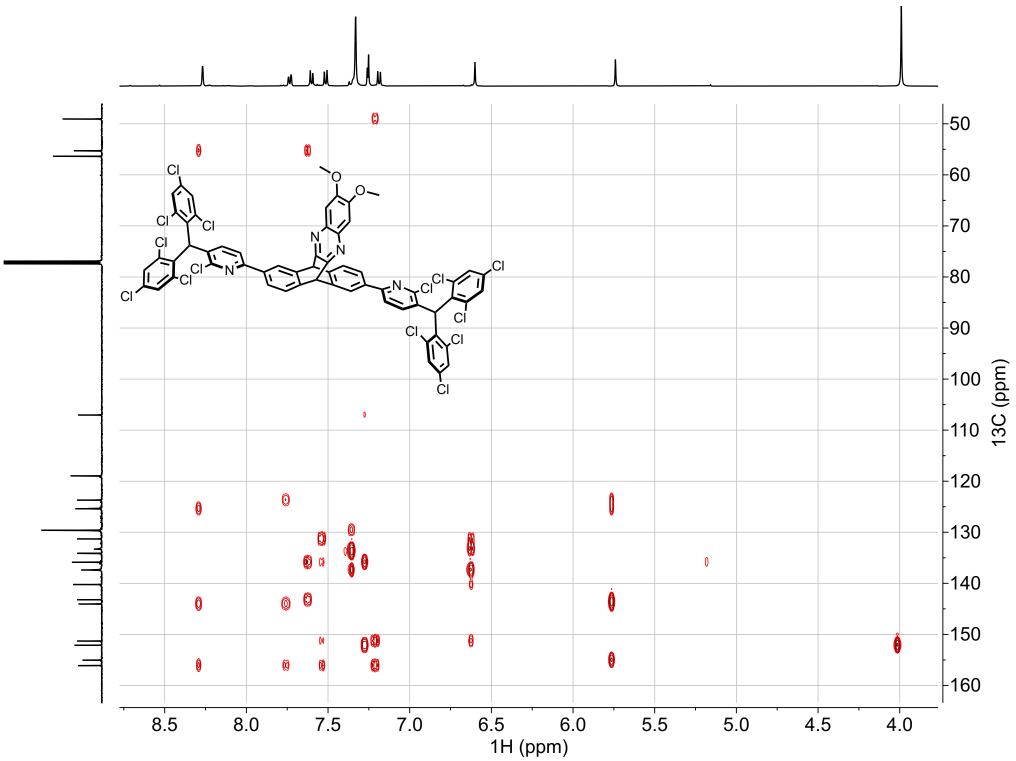

Figure S127: ^{1}H-^{13}C HMBC of compound **2H** in $CDCl_3$.

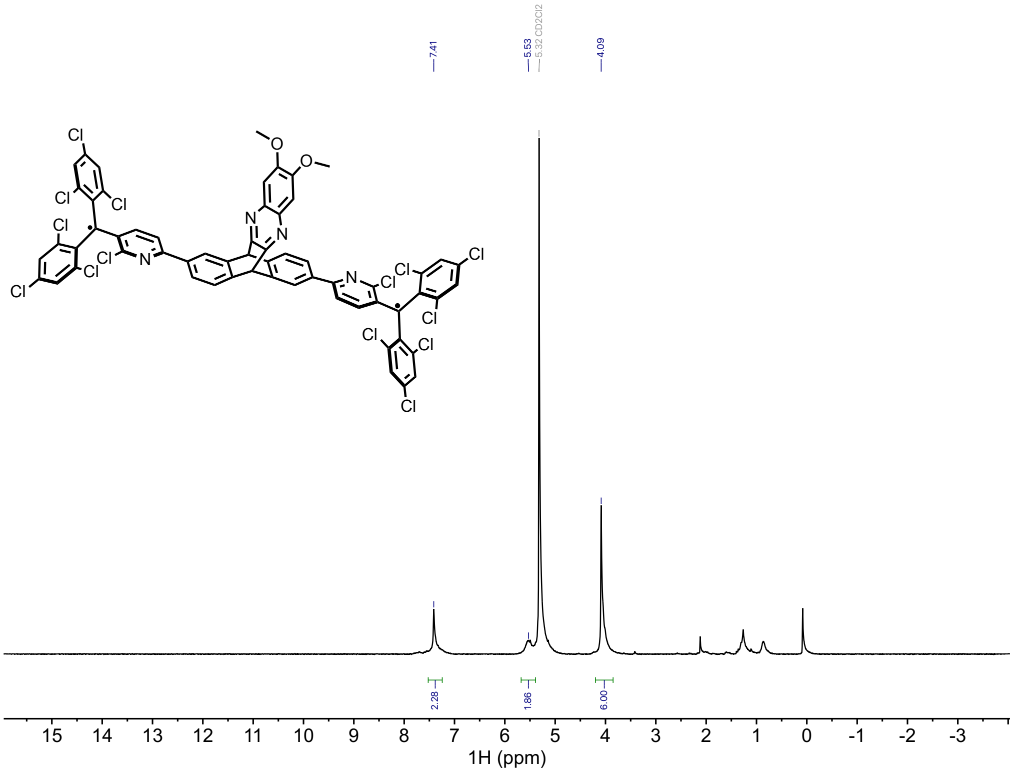

Figure S128: ^{1}H NMR (500 MHz, CD_2Cl_2) of compound **2** at room temperature.

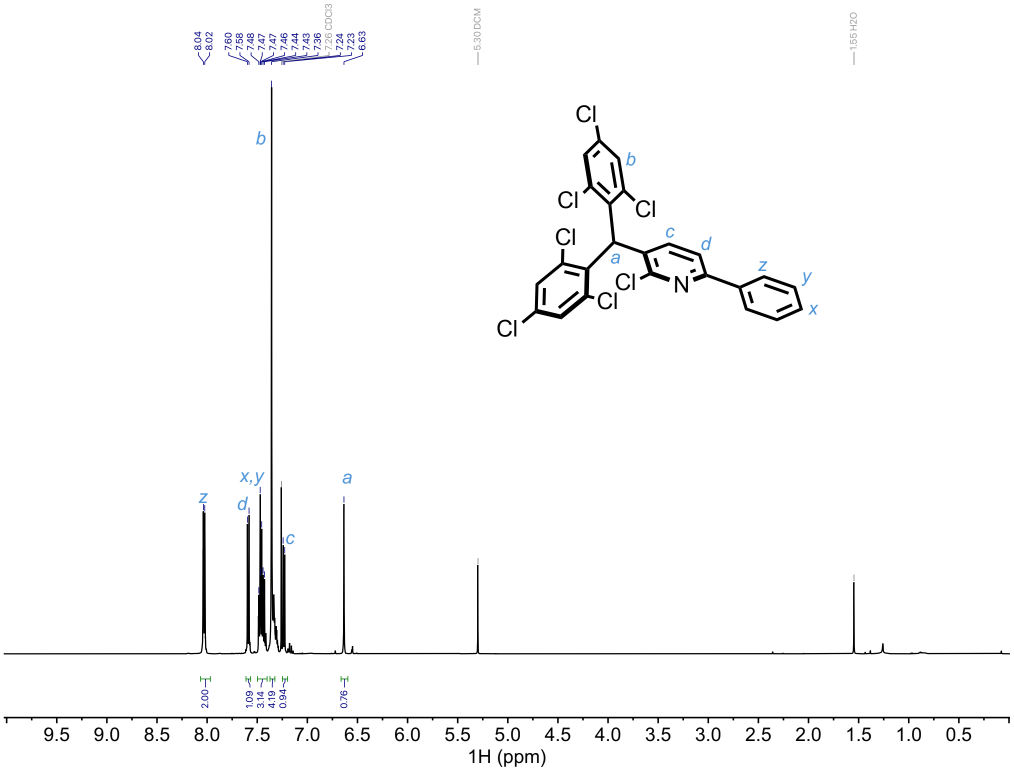

Figure S129: ^{1}H NMR (500 MHz, $CDCl_3$) of compound **3H**.

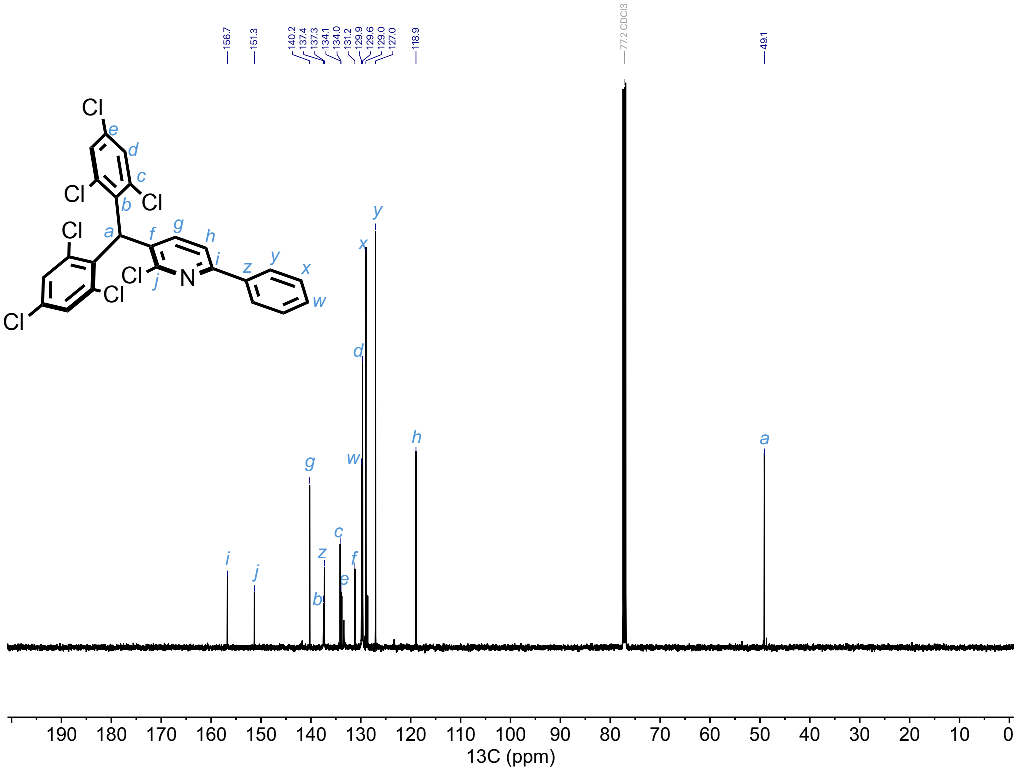

Figure S130: $^{13}C\{^{1}H\}$ NMR (126 MHz, $CDCl_3$) of compound **3H**.

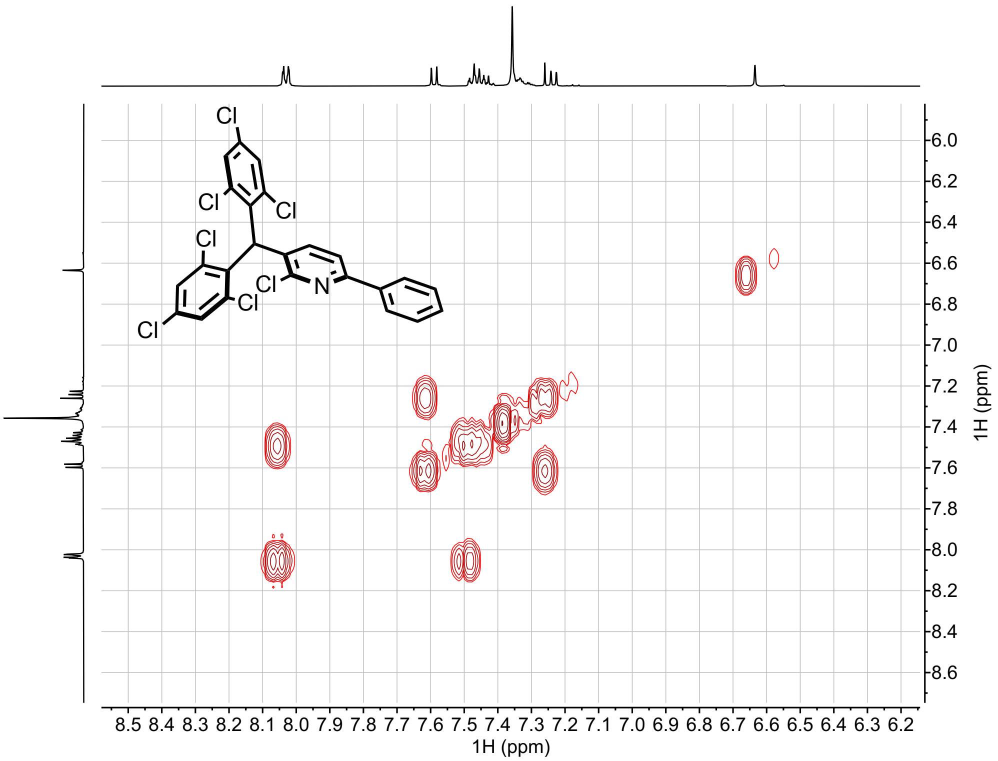

Figure S131: ^{1}H-^{1}H COSY of compound **3H** in $CDCl_3$.

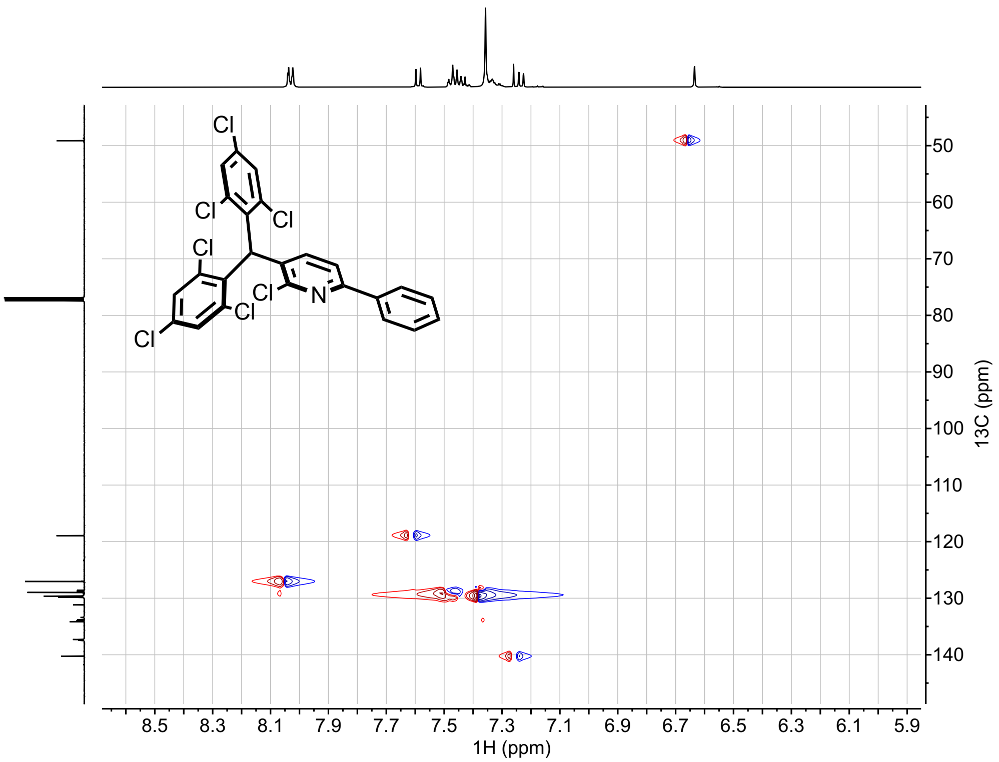

Figure S132: ^{1}H-^{13}C HSQC of compound **3H** in $CDCl_3$.

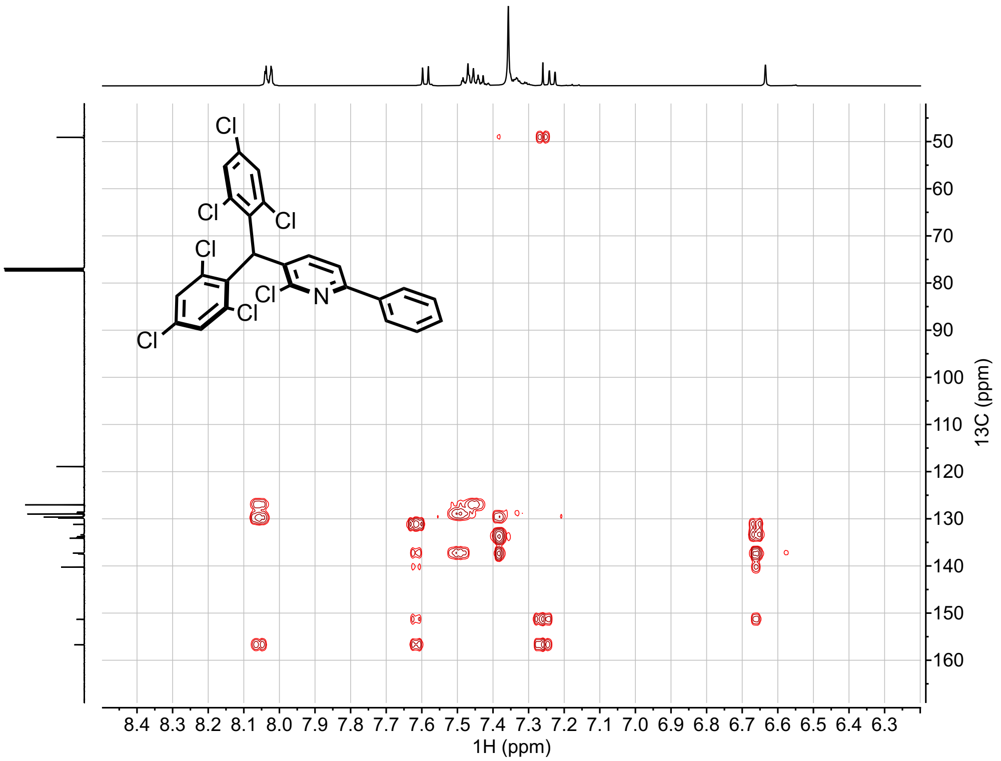

Figure S133: ^{1}H-^{13}C HMBC of compound **3H** in $CDCl_3$.

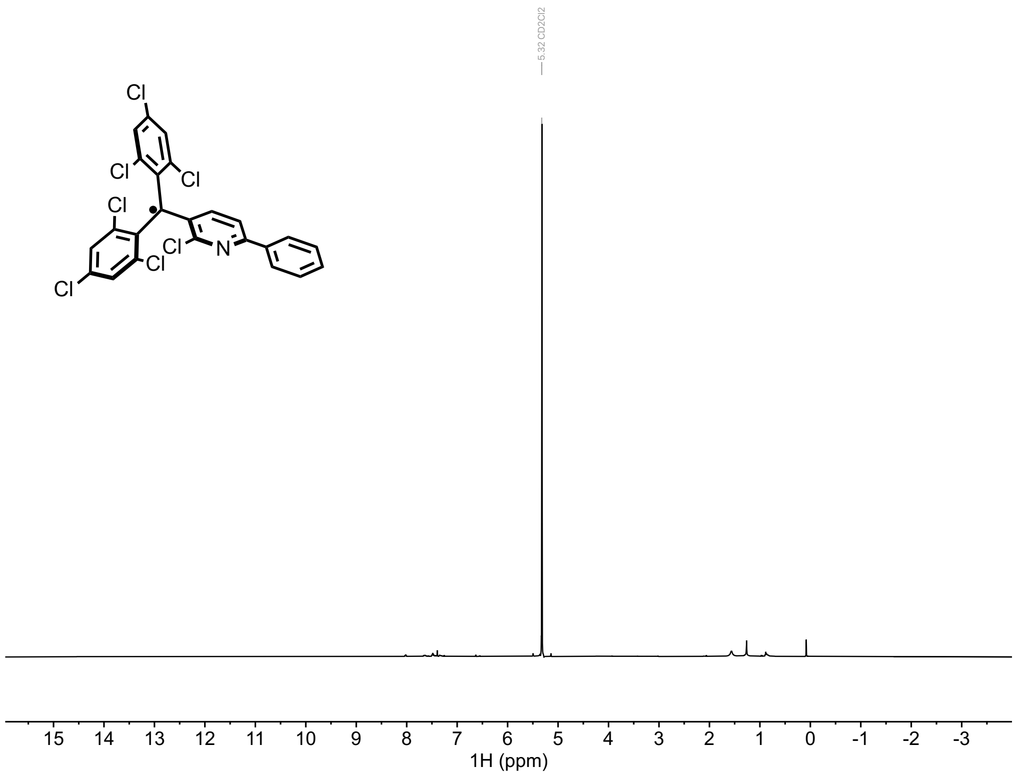

Figure S134: ^{1}H NMR (500 MHz, CD_2Cl_2) of compound **3** at room temperature.
